# Shuttle HAT for mild alkene transfer hydrofunctionalization

**DOI:** 10.1038/s41467-024-53281-7

**Published:** 2024-10-30

**Authors:** Tanner C. Jankins, Philip M. Blank, Andrea Brugnetti, Philip Boehm, Françoise A. Aouane, Bill Morandi

**Affiliations:** Laboratorium für Organische Chemie, ETH Zürich, 8093 Zürich, Switzerland

**Keywords:** Synthetic chemistry methodology, Homogeneous catalysis, Synthetic chemistry methodology

## Abstract

Hydrogen atom transfer (HAT) from a metal-hydride is a reliable and powerful method for functionalizing unsaturated C–C bonds in organic synthesis. Cobalt hydrides (Co–H) have garnered significant attention in this field, where the weak Co–H bonds are most commonly generated in a catalytic fashion through a mixture of stoichiometric amounts of peroxide oxidant and silane reductant. Here we show that the reverse process of HAT to an alkene, i.e. hydrogen atom abstraction of a C–H adjacent to a radical, can be leveraged to generate catalytically active Co–H species in an application of shuttle catalysis coined shuttle HAT. This method obviates the need for stoichiometric reductant/oxidant mixtures thereby greatly simplifying the generation of Co–H. To demonstrate the generality of this shuttle HAT platform, five different reaction manifolds are shown, and the reaction can easily be scaled up to 100 mmol.

## Abstract (web summary)

Hydrogen atom transfer (HAT) from a metal-hydride is a reliable and powerful method for functionalizing unsaturated C–C bonds in organic synthesis. Herein, the authors show that reverse process of HAT to an alkene can be leveraged to generate catalytically active Co–H species in an application of shuttle catalysis coined shuttle HAT.

## Introduction

**Fig. 1:**
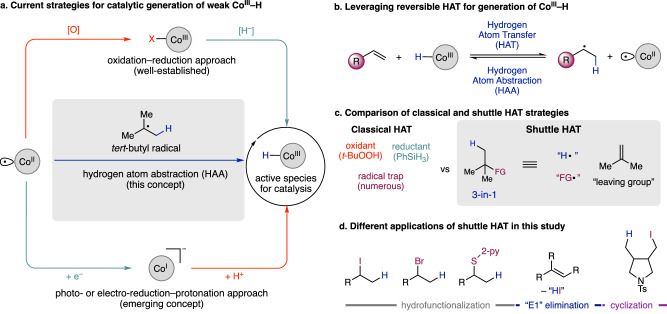
Overview of previous MHAT methods and this study. **a** Different approaches to generate unstable Co(III)–H from Co(II) sources. The most common or classical approach is shown on the top path where Co(II) is first oxidized to Co(III), then the hydride is introduced through transmetallation. The bottom path describes a more recent concept where the Co(III)–H is formed from reduction of Co(II) to Co(I) followed by protonation. The middle path describes this work where the Co(III)–H is formed directly from Co(II) by abstracting a hydrogen atom from a C–H bond adjacent to a radical. **b** The underlying reversibility of HAT and HAA that the shuttle HAT concept is based on. **c** A comparison of classical methods for HAT hydrofunctionalization of alkenes compared to the shuttle HAT strategy (**d**) The different synthetic applications explored in using this approach towards catalytic Co(III)–H.

Carbon-centered radicals are versatile reactive intermediates which are widely used to produce polymers^1^, pharmaceuticals^2^, and other fine chemicals. In the context of complex molecule synthesis and drug discovery there is an increasing demand for C(*sp*^*3*^)-rich architectures which are often most readily accessible through radical based reactions^3,4^. To accelerate the discovery and synthesis of C(*sp*^*3*^)-rich molecules, a general methodology that can install readily diversifiable functional handles from simple olefin building blocks would be an ideal tool to access an array of different products. Currently, hydrogen atom transfer (HAT) from a metal-hydride to an alkene is among the most reliable methods for alkyl radical generation and can be used in a myriad of transformations^5–11^. However, the weak metal hydrides which undergo HAT have limited approaches to form under catalytic conditions (Fig. 1a). In these approaches, the cobalt(II) must be oxidized or reduced first before the hydride can be formed, and cannot be formed directly from cobalt(II). Initial pioneering work utilized a silane reductant and peroxide oxidant mixture where first, single-electron oxidation of the catalyst forms a M–X bond which is followed by transmetalation from the silane (Fig. 1a, top). The use of both oxidant and silane reductant in the same pot produces large amounts of unnecessary waste, is challenging to employ in large scale applications, and is less than ideal from a catalysis perspective. To overcome the problem of using the oxidant/silane mixture, photo- and electroreductive protocols have recently been developed by several groups as an emerging alternative to the classical oxidative conditions^12–18^. In these reactions, the catalytic metal-hydride is formed through reduction of a Co(II) center followed by protonation to form the Co(III)–H (Fig. 1a, bottom). While advantageous over the classical oxidative conditions as it avoids the use of silanes and oxidants, the reductive approach still requires acidic and reducing conditions to generate the Co–H from Co(II) sources (Fig. 1a, bottom). Norton and Chirik^19–21^ have also shown that metal hydrides that undergo HAT can be generated with H_2,_ but these approaches are limited to hydrogenation and alkene isomerization and feature much stronger M–H species (BDE > 50 kcal/mol). While attractive in those contexts, they may not be applicable in hydrofunctionalization due to the fast rate of hydrogen atom transfer from M–H to alkyl radicals to produce the hydrogenated product^22^. Therefore, there is a clear demand for conceptually distinct strategies to access these catalytic manifolds under milder reaction conditions, ideally using neither reducing or oxidizing and pH neutral conditions to enable a broad substrate scope and allow the introduction of highly reactive functional handles that are crucial to synthetic endeavors (Fig. 1a, middle).

Due to our lab’s interest in reversible catalysis^23–28^, we envisioned that a single-electron variation of the shuttle catalysis framework could form catalytically active Co–H by using the reverse process of hydrogen atom transfer to an alkene (Fig. 1b). In this way the Co–H would be formed directly from abstraction of a C–H bond adjacent to a radical^29–34^. Combining reversible Co–H formation with another reversible process could produce two interwoven catalytic cycles that allow for the net transfer of a hydrogen atom and functional group from one molecule (the donor) to another molecule containing an alkene (the acceptor). Crucially, the design of this shuttle HAT platform would not only be limited to a single functional group donor, but could be amenable to incorporating numerous different functional groups (Fig. 1d).

## Results

**Fig. 2:**
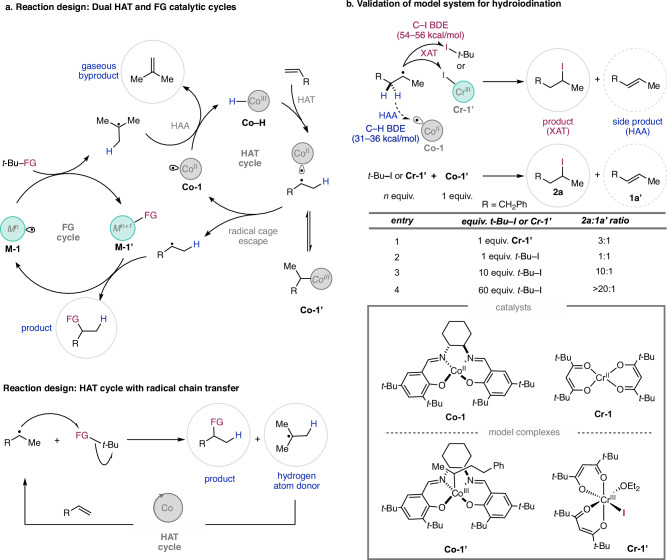
Reaction design and validation. **a** Reaction design for dual catalytic pathways (top). The reaction starts with cleavage of a single electron cleavage of a FG by a metal catalyst to generate a *tert*-butyl radical. A Co(II) species (**Co-1**) then abstracts a hydrogen atom to form isobutene and a Co–H. The **Co–H** undergoes HAT with an alkene to first form a radical cage pair followed by radical cage escape and trapping of the alkyl radical with the oxidized FG transfer catalyst **M-1’** to generate the product and turn over the catalyst. **b** Model complexes of catalytically relevant species and their use in stoichiometric reactions to demonstrate the feasibility of the proposed mechanisms. The table shows how using model complex **Cr-1’** or *t-*BuI can successfully form the product with decreasing amounts of side product as the amount of the *t-*BuI is increased. For synthesis of the model complexes, see Supplementary Information Section 4.

Although there are numerous potential functional groups which could be applied in this methodology, we chose to initially focus on a hydroiodination reaction because of the synthetic versatility of alkyl iodides, and to demonstrate the mildness of our protocol by forming highly reactive C–I bonds under catalytic conditions. Indeed, alkyl iodides have historically been challenging to access using catalytic HAT protocols^17,35–37^. In this sense, the shuttle catalysis framework is advantageous over traditional approaches for forming highly reactive products, such as alkyl iodides^38–40^, because the catalyst is designed to reversibly react and reform the product, while traditional approaches are prone to irreversible degradation of the product by the catalyst or reagents. To intertwine with the reversible HAT cycle, we derived inspiration for a reversible halogen atom transfer (XAT) cycle from atom transfer radical polymerization (ATRP) where a halogen is reversibly cleaved and introduced in the propagation step of living polymerization (Fig. 2a, FG cycle)^1^.

The use of cobalt salen complexes for reversible HAT in alkene isomerization and 1,5-HAT^29,34^, prompted us to focus on this ligand scaffold for our initial investigations. After considering numerous potential XAT catalysts, we chose to investigate the Cr(II/III) redox couple because of the high chemoselectivity for reversibly cleaving C(*sp*^*3*^)–I bonds^41^ and the excellent functional group tolerance of chromium in these oxidation states^42^. We preferred Cr over the more commonly used Cu ATRP catalysts because Cu is more prone than Cr to undergo undesired side reactivity such as coupling reactions^43–45^.

**Fig. 3:**
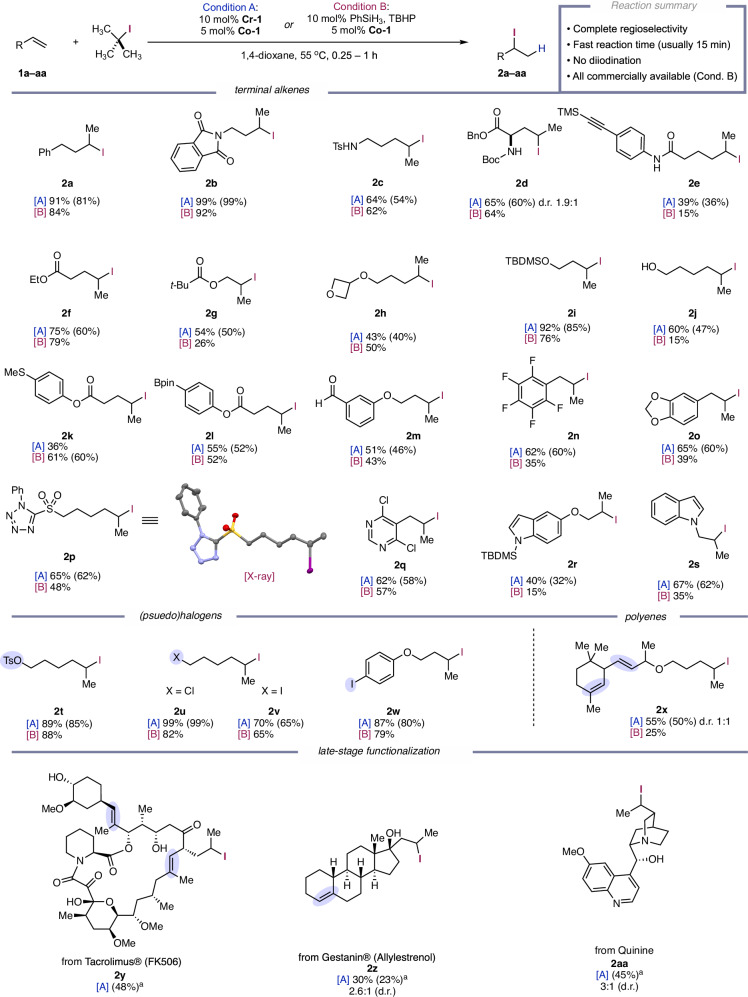
Substrate scope. The reaction was run using 5 mol% of **Co−1** and 10 mol% of **Cr-1** (Condition A) or 10 mol% TBHP/PhSiH_3_ (condition B) with 1 equivalent of alkene and 3 equivalents of *t-*Bu–I in 1,4-dioxane ([0.1 M] Condition A; [1 M] condition B) in a sealed tube under argon. The data given reflects conditions A, [A], and conditions B, [B]. The yield is given as [A] ^1^H NMR yield (isolated yields are in parenthesis). In most cases, incomplete conversion of alkenyl starting material accounts for the remaining mass balance for lower yielding substrates. In more sterically hindered substrates, 1-position alkene isomerization is often also observed in low amounts, but hydrogenated alkene could never be detected. Abbreviations: TBDMS *tert*-butyl dimethylsilane, Bpin boronic pinacol ester, TMS trimethylsilane. ^*a*^ The reaction conditions were modified using 10 mol% of **Co-1**, and 20 mol% of **Cr-1**. Normal catalyst loading does not provide full conversion of the starting material which cannot be separated from the product, so high loadings of catalyst were required.

A concise set of experiments was initially designed to test the feasibility of the proposed reaction (Fig. 2b). It was unclear whether XAT to form the product (**2a**) could outcompete hydrogen atom abstraction (HAA) to form side products (**1a’**) considering that HAA is an inner sphere process while XAT is an intermolecular process, and that the C–H bond dissociation energy (BDE) is significantly lower than the C–I BDE of *t*-BuI (31–36 kcal/mol^33^ vs. 54 kcal/mol^46^). In a XAT vs. HAA competition experiment, a potentially catalytically relevant XAT species **Cr-1’** and alkyl radical precursor **Co-1’**^47^ were heated together. To our gratification, the desired product **2a** was formed in a 3:1 ratio with the internal olefin side product **1a’** (Fig. 2b, entry 1). While XAT from a catalytic M–X species was envisioned as one viable pathway to generate the product, another possible pathway for C–I bond formation is through direct XAT of *tert-*butyl iodide with an alkyl radical in a radical chain transfer pathway^46^. To test this, **Co-1’** was heated with varying amounts of *t-*BuI. While 1 equivalent of *t-*BuI gave a 1:1 ratio of product **2a** to side product **1a’** (Fig. 2b, entry 2), increasing the amount eventually resulted in only trace amount of the internal alkene side product (Fig. 2b, entry 4). These stoichiometric experiments with **Co-1’,** **Cr-1’**, and *t-*BuI suggest XAT from *t-*BuI is a selective method for C–I bond formation when *t-*BuI concentration is high, while an XAT pathway from catalytic **Cr-1’** may also be selective at low concentrations (Fig. 2b). Implementing an XAT catalyst in combination with *t-*BuI would provide two potential pathways for C–I bond formation. Using an XAT catalyst with *t-*BuI could also be beneficial in case the radical chain is prematurely terminated. Then, the radical chain process could be reinitiated by small amounts of XAT catalyst and could prevent the reaction from dying in case of chain termination. Indeed, we observed premature termination of the radical chain only when no chromium catalyst was present (condition B) as evidenced by incomplete conversion of the alkene starting materials for many substrates in Fig. 3 (*vide infra*). Lastly, we demonstrated the catalytic relevance of **Cr-1’** and **Co-1’** by using them as pre-catalysts in place of **Cr-1** and **Co-1** to yield of the desired product (see Supplementary Fig. 4).

After establishing key reactivity for C–I bond formation, we began investigating catalytic conditions using unactivated alkene **1a** and 3 equivalents of *t-*BuI with the commercial Co(II)–salen catalyst **Co-1**. We initially developed high yielding conditions with **Cr-1** as an XAT co-catalyst (Fig. 3, Condition A). Because **Cr-1** requires manipulation under inert atmosphere, we sought to develop a second set of conditions which could be more accessible for synthetic practitioners. Using a catalytic amount of initiators to form the Co–H in situ from **Co-1**, we reoptimized the reaction conditions to be advantageous from an accessibility point of view, because all of the principal components of the reaction are commercially available, air stable, and inexpensive (Fig. 3, condition B). Other known reversible XAT catalysts that require visible light irradiation, [Fe(Cp)(CO)_2_]_2_^48^ and Mn_2_(CO)_10_^49^, were both competent in the reaction, although accompanied by formation of diiodinated product which cannot be easily separated from the hydroiodination product (See Supplementary Table 1)^48^. Other Cr(II) salts that are known to react with alkyl iodides proved inferior in terms of conversion and yield (See Supplementary Table 1)^50^. Although the reaction could be carried out at room temperature, mild heating to 65 °C drastically improved the yield, likely due to increased volatility of the byproduct isobutene which is more reactive towards the Co–H species than **1a**^5^. Electron-donating substituents on the salen backbone performed slightly worse than **Co-1**, while electron-withdrawing groups completely shut down reactivity. This was anticipated based on previous reports which support electron-rich Co-salen complexes undergoing radical cage escape faster than electron-poor ones^51,52^.

## Discussion

Using these two catalytic protocols we evaluated the generality of the reaction scope (Fig. 3). Protected amines (Phthalimide, **2b**; Tosyl, **2c**; Boc, **2d**) performed with quantitative to high yields and **2c** or **2d** did not give the corresponding cyclized products. The Boc protected benzyl ester of allyl glycine (Boc-allyl-Gly-OBn) (**±-1d**), gave good yields despite offering little control over the diastereoselectivity (1.9:1 d.r.) in the product **2d**. While alkene substrates containing terminal alkynes were not compatible with the reaction, alkene substrates containing silyl-aryl alkynes could be used to chemoselectively hydroiodinate the alkene to afford **2e**. Esters and protected alcohols **2f-2i** worked well in the reaction, including the acid-sensitive oxetane in **2** **h**. Free primary alcohol **1j** also performed very well and cleanly forms **2j** using conditions A despite potential nucleophilic attack on *t*-BuI or 6-*exo-tet* cyclization of the product. Conditions B gave a drastic decrease in yield (15%) likely due to side-reactivity of the free alcohol with the silane to form a silyl ether^53^. Functional groups that are prone to oxidation from peroxides formed the desired thioether-containing product **2k** and boronic ester-containing product **2** **l** in good yields. Interestingly, conditions B significantly outperformed conditions A in forming **2k** likely due to inhibition of the chromium catalyst from the sulfur in the thioether. The aryl aldehyde **1** **m**, gave the desired product **2** **m** in moderate yield with both sets of conditions. Nozaki-Hiyama-Kishi-type reactivity was not observed^54^, suggesting the *tert-*butyl substituents on the ligand shield the chromium center from forming nucleophilic alkyl-Cr(III) species. Allyl benzenes containing electron-withdrawing (**2n**) or -donating groups (**2o**) performed equally well, giving good yields with conditions A and slightly diminished yields when using conditions B.

We next investigated a range of *N-*heterocyclic compounds. Sulfonyl tetrazole product **2p** was obtained in good yields and gave single crystals suitable for X-ray analysis to unambiguously assign the product structure. Chloropyrimidine **1q** gave the product **2q** in good yield. Two indole derivatives worked to varying degrees with *N*-allylated indole **2** **s** performing better than allyl ether **2r**. In this case, a similarly low yield was obtained for another allyl ether **2** **g**, suggesting that this particular motif was the cause of the diminished yield and not the indole itself.

Due to the exquisite chemoselectivity exhibited by **Cr-1** for the preferential activation of more highly substituted halides, we investigated substrates with primary (pseudo)halides that would otherwise react first with metals that undergo S_N_2 oxidative additions^55^. Tosylate **2t**, chloride **2** **u**, and even iodide **2** **v** gave great to quantitative yields showing no undesired side reactions with the other halides. Due to the instability of aryl radicals, single-electron activation by **Cr-1** occurs selectively on alkyl halides allowing aromatic iodide product **2w** to be obtained in high yield. Lastly, the kinetic preference for **Co-1** to undergo HAT to terminal or 1,1-disubstituted alkenes allowed us to chemoselectively functionalize a terminal alkene in good yield (**2x**)^29^, even in the presence of internal di- or tri-substituted alkenes (**±-1x**).

Similar to the HAT hydroiodination protocol of Ohmiya and Nagao^17^, styrenes, 1,1-disubstituted alkenes, and tri-substituted alkenes were among the low yielding and unsuccessful coupling partners. 1,1-disubstituted alkenes formed isomerized tri-substituted alkene side products as the major species showing that HAA outcompetes XAT for this type of substrate. We attribute the instability of benzyl iodides and tertiary iodides, and slower iodine atom transfer to the more stabilized benzylic or tertiary radicals as reasons for their poor reactivity. Such species are typically prepared and used in situ as they decompose in the absence of stabilizers, e.g. the tertiary iodide **2ad’ **decomposed within 24 h, even at –40 °C (*vide infra*).

Next, we sought to demonstrate the broad synthetic applicability of this strategy in the late-stage functionalization of a structurally diverse set of FDA approved drugs. We challenged the method in the context of a highly functionalized and complex natural product, FK506. This natural product has multiple potential alkenes for functionalization, and numerous potentially reactive functional groups, but performed well to give the desired product **2** **y** in 48% yield. Methodologies which can chemoselectively modify the terminal alkene of FK506 are desirable for medicinal chemists as several known analogs contain modifications in this area of the molecule^56,57^. Using the progesterone receptor agonist allylestrenol, the desired product **2z** was obtained in 23% yield with complete chemoselectivity for the terminal alkene to give the product without detectable amounts of cyclized oxirane. Lastly, the alkaloid natural product and malaria treatment drug quinine was hydroiodinated to give **2aa** in 45% yield, showing the method can tolerate unprotected amines and pyridine-type heterocycles.

To expand the applications of this methodology, we envisioned cyclizations through either 2- or 1-electron pathways could give complementary approaches to compounds of different ring size. To form the small 4-membered ring, hydroiodination of an alkenyl secondary amine gave rise to a transiently formed alkyl iodide **2ab** which underwent ring closure to form the azetidine **2ab’** as a single diastereomer. The possible reversibility of the ring closure, and C–I bond forming step appear to be crucial for achieving the high diastereoselectivity. Overall, this approach for intramolecular hydroamination provides complementary access to N-heterocycles with electron-rich amines compared to oxidative^58^ or reductive^59^ MHAT protocols which utilize electron-poor amines and have comparatively poor diastereoselectivity. Next, we were able to synthesize a 5-membered pyrrolidine ring through 5-*exo-trig* radical cyclization and trapping of the resulting methyl radical to give the primary iodinated product **2ac’**. The d.r. in this case is close to the expected kinetic distribution for the cyclization step (~2:1, *cis:trans*)^60^ suggesting that formation of primary iodides occurs irreversibly as the thermodynamically favored *trans* product would be expected under fully reversible conditions. Interestingly, in both cases only conditions A were able to give the desired product while condition B failed to give any reaction.

The reversible nature of this catalytic process could allow us to run the reverse reaction for a single-electron variation of the E1 elimination reaction and selectively dehydroiodinate alkyl iodides to give alkene products^31,61^. Using the diene (±)-citronellene, an unselective global hydroiodination could first be done using MsOH and LiI to give **2ad’**. Then, using norbornadiene as an HI acceptor, condition B gave clean formation of the secondary iodide product **2ad**, accompanied by formation of iodonorbornene. Formation of the 1,1-disubstituted alkene regioisomer of **2ad** was also observed as a minor (8:1) byproduct.

**Fig. 4:**
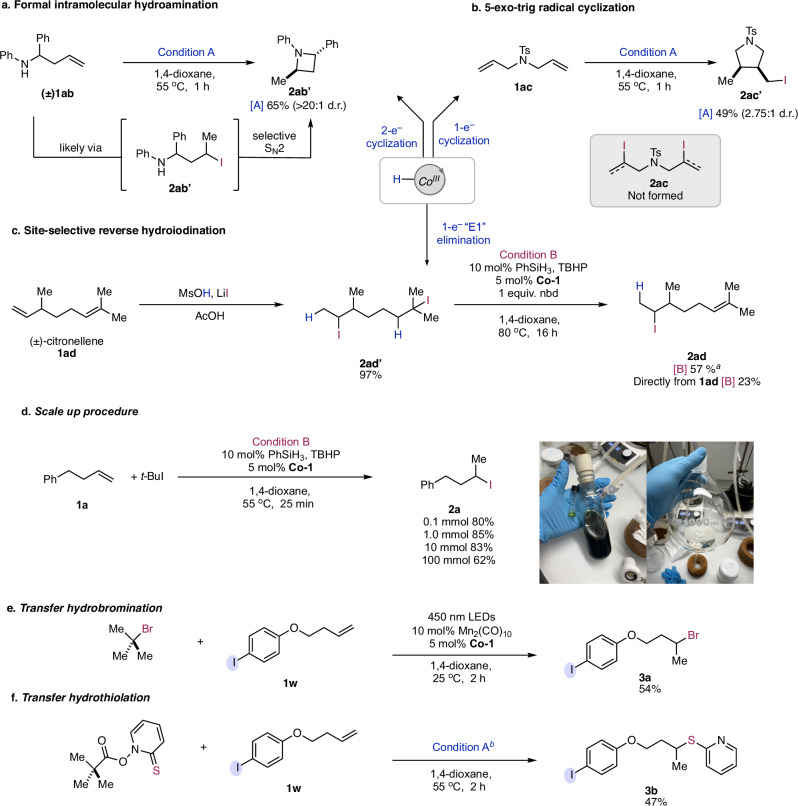
Applications of shuttle HAT. **a** Hydroiodination followed by ring closure to form azetidine. **b** 5-exo-trig radical cyclization to form pyrrolidines. No ring open hydroiodinated products were observed. **c** An unselective acid-promoted global hydroiodination followed by selective transfer hydroiodination of the tertiary iodide to norbornadiene (nbd) as an acceptor. Alternatively, **2ad** can be accessed from **1ad** via condition B in lower yield. **d** Scalability of the procedure. The picture on the left shows 0.1 mmol reaction (vial) and 100 mmol reaction (Schlenk tube). The right picture shows isolated product from the 100 mmol reaction. **e** Applying this method to transfer hydrobromination using tert-butyl bromide. **f** Applying this method to transfer hydrothiolation using the *tert*-butyl Barton ester. ^*a*^ The yield was determined by ^1^H NMR using CH_2_Br_2_ as an internal standard. ^*b*^ The reaction conditions were modified using 10 mol% of **Co-1**, and 20 mol% of **Cr-1**. Normal catalyst loading does not provide full conversion of the starting material which cannot be separated from the product, so high loadings of catalyst were required.

From the onset of this investigation, we envisioned that one of the main advantages of using shuttle HAT compared to classical methods is the ease of scalability. Traditional methods employ stoichiometric amounts of reductant and oxidant together in the same pot which is challenging to scale and causes excess waste as the two reagents inevitably quench each other to varying degrees^62^. Indeed, our protocol proved to be scalable as we were able to run the reaction from 0.1 mmol scale to 100 mmol scale, without changing the reaction conditions (Fig. 4d). One possible reason for the slight decrease in yield on 100 mmol scale is due to the decomposition of *t-*BuI as evidenced by the reagent progressively turning into a dark brown color during the longer time required for freeze-pump thaw on this scale. Using the scaled up **2a**, we demonstrated a series of derivatizations that leverage a photoredox XAT strategy developed by Leonori^63,64^, as well as polar disconnections where the alkyl iodide is an electrophile for an S_N_2 reaction (see Supplementary Information, Section 5). The S_N_2 reaction can even be run with a mixture of **1a** (40%) and **2a** (60%) without significant change in yield, which obviates the need for separation of unreacted starting material in subsequent reactions.

To showcase the generality of the transfer HAT strategy for the installation of other functional groups, a hydrobromination and hydrothiolation were carried out using slightly modified conditions from the hydroiodination reaction (Fig. 4e, f). This illustrates the generality and possibility of combining the shuttle HAT reaction with other functional group transfer reactions to enable a broad range of hydrofunctionalization reactions. Both catalytic XAT and radical chain transfer pathways were shown to be operable in the installation of other functional groups (**3a** and **3b** respectively), suggesting that new shuttle HAT reactions could be rationally designed based on both dual catalytic atom transfer cycles as well as through radical chain transfer pathways.

Leveraging the concept of shuttle catalysis, a strategy for generating HAT-active Co–H has been developed. This shuttle HAT protocol allows the most direct access to catalytic Co–H directly from Co(II) sources by abstraction of hydrogen atom from a C–H bond. In doing so, this approach avoids the oxidation–transmetalation sequence^6^, or reduction–protonation sequence of the cobalt center to generate catalytic Co–H^14–18^. The mildness of the shuttle HAT platform is exemplified in the ability to produce highly reactive alkyl iodides in a catalytic fashion using substrates that feature a range of unprotected functional groups as well as medicinally active scaffolds. The reaction proved to be scalable up to 100 mmol without modification to the procedure, and could be used in several different HAT reaction manifolds beyond hydroiodination. This lays the foundation for new HAT methodologies and radical trap reagents to be designed around this reversible catalytic process.

## Methods

### General procedure for catalytic condition A

Inside an argon-filled glovebox, to a 2-dram vial equipped with a magnetic stirring bar, **Co-1** (3.0 mg, 5.0 µmol), **Cr-1** (4.2 mg, 10 µmol), and the given substrate (0.100 mmol) were dissolved in 1,4-dioxane (1 mL). *t*-Butyl iodide (37 µL, 0.300 mmol) was added and the vial was sealed and removed from the glovebox. The reaction mixture was stirred at 55 °C for 0.25–1 h, then the solution was filtered through a silica plug and washed with pentanes (4 mL) and Et_2_O (4 mL). The solvent was removed in vacuo and the crude product was dissolved in CDCl_3_ and internal standard (CH_2_Br_2_) and the crude yield was calculated. The material was purified by flash silica column chromatography or preparative TLC.

### General procedure for catalytic condition B

Inside an argon-filled glovebox, to a 2-dram vial equipped with a magnetic stirring bar, **Co-1** (3.0 mg, 5.0 µmol), and the given substrate (0.100 mmol) were dissolved in 1,4-dioxane (0.1 mL). *t*-Butyl iodide (36.8 µL, 0.300 mmol) was added and the vial was sealed and removed from the glovebox. TBHP (1.8 µL, 0.0100 mmol, 6.6 M in hexane) was added by Hamilton syringe directly through a puncturable cap and the vial was shaken. This results immediately in a color change from a red to brown solution. ~ 30 s after adding TBHP, phenylsilane (1.2 µL, 0.0100 mmol) was added by Hamilton syringe directly through a puncturable cap. The reaction mixture was stirred at 55 °C for 0.25–1 h, then the solution was filtered through silica and washed with pentanes (4 mL) and diethyl Et_2_O (4 mL). The solvent was removed in vacuo and the crude product was dissolved in CDCl_3_ and internal standard (CH_2_Br_2_) and the crude yield was calculated. The material was purified by flash silica column chromatography or preparative TLC.

### General procedure for catalytic condition B (large scale)

Using a modified procedure from General Procedure B, alkene (1.0 equivalent), tert-butyl iodide (3.0 equivalents), 1,4-dioxane [1 M] and **Co-1** (5 mol%) were added under air to an appropriately sized Schlenk tube (>1/2 the volume of the reaction). The tube was closed and freeze-pumped-thawed under N_2_ three times. Then, through a septum, non-degassed t-BuOOH (6.6 M in hexanes) (10 mol%) was added resulting in a brown solution followed ~ 30 s later by non-degassed PhSiH_3_ (10 mol%). The reaction was heated to 55 °C under a continuous stream of N_2_ for 25 min then cooled to room temperature. The solvent was removed in vaccuo and the reaction diluted with pentanes and a saturated solution of sodium thiosulfate. The aqueous phase was extracted with pentanes (3x), dried over MgSO_4_ then solvent evaporated. The crude oil was purified by flash silica column chromatography.

## Supplementary information

Supplementary Information

Peer Review File

## Data Availability

Details about the materials and methods, experimental procedures, mechanistic studies, characterization data, and NMR spectra are available in the Supplementary Information. The X-ray crystallographic coordinates for structures reported in this study have been deposited at the Cambridge Crystallographic Data Centre (CCDC), under deposition numbers 2306574, 2306575. These data can be obtained free of charge from The Cambridge Crystallographic Data Centre via www.ccdc.cam.ac.uk/data_request/cif. All data are available from the corresponding author upon request.
