# Supplementary material for: Shuttle HAT for mild alkene transfer hydrofunctionalization
Source: Nat Commun. 2024 Oct 30;15:9397. doi: 10.1038/s41467-024-53281-7 (PMC11525564; doi:10.1038/s41467-024-53281-7)
Supplement: Supplementary file 1 — Supplementary Information [file 41467_2024_53281_MOESM1_ESM.pdf]

Supplementary Information for

Shuttle HAT for mild alkene transfer hydrofunctionalization

Tanner C. Jankins[1], Philip M. Blank[1], Andrea Brugnetti[1], Philip Boehm[1], Françoise A. Aouane[1], Bill Morandi[1]*

[1]*Laboratorium für Organische Chemie, ETH Zürich, 8093 Zürich, Switzerland.*

Table of contents

Supplementary Methods

1 General information 2

2 Optimization of reaction conditions 4

3 Starting material synthesis 5

4 Substrate scope 10

5 Product derivatization 35

6 Preparation of catalysts 38

Supplementary Discussion

7 Mechanistic considerations 40

Supplementary Notes

8 NMR spectra 41

9 X-ray crystallographic data 95

10 Supplementary References 117

1 General information

Materials: Unless otherwise stated, reagents were used as supplied from commercial sources without any further purification. Extra-dry THF, DCM, ethyl acetate, dioxane, diethyl ether, and benzene were purchased from Acros in 100-mL sure seal bottles and used directly without further purification. Various alkene starting materials that were used in previous studies were used as received.[1, 2] All screening reactions were set up in a glovebox in 2-dram vials unless stated otherwise.

NMR: ^{1}H- and ^{13}C-NMR spectra were recorded on a Bruker AVIII 400 MHz, a Bruker Neo 400 MHz or a Bruker Neo 500 MHz spectrometer at 298 K and are reported in parts per million (ppm). ^{1}H-NMR spectra are calibrated with respect to the corresponding solvent residual peak ($CHCl_3$: 7.26 ppm). ^{13}C-NMR spectra are calibrated with respect to the corresponding solvent residual peak ($CHCl_3$: 77.16 ppm). Multiplet signals are reported as follows: s = singlet, d = doublet, t = triplet, q = quartet, m = multiplet, or combinations thereof. ^{13}C signals are acquired with proton decoupling and are singlets unless otherwise stated. NMR yields were determined using CH_2Br_2 as an internal standard.

Analytical thin-layer chromatography (TLC) was performed using silica gel 60 F254 coated glass plates (Merck). Visualization was achieved by ultraviolet fluorescence (λ = 254 nm) and/or staining with potassium permanganate ($KMnO_4$).

Preparative thin-layer chromatography (TLC) was performed using silica gel 60 F254 coated glass plates (Merck). Visualization was achieved by ultraviolet fluorescence (λ = 254 nm).

Flash column chromatography was performed using silica gel 60 (pore size = 60 Å, mesh: 40–63 μm from Sigma-Aldrich or SiliCycle).

High resolution mass spectrometry (HRMS): HRMS data were obtained by the mass spectrometry service in the Laboratory of Organic Chemistry at ETH Zürich on VG-TRIBRIB for electron impact ionization (EI), a Varian IonSpec Spectrometer for electrospray ionization ESI or an IonSpec Ultima Fourier Transform Mass Spectrometer for matrix-assisted laser desorption/ionization (MALDI) and are reported as (m/z).

X-Ray analysis: Single crystalline samples were measured on a Riguka Oxford Diffraction XtaLAB Synergy-S Dualflex kappa diffractometer equipped with a Dectris Pilatus 300 HPAD detector and using microfocus sealed tube Cu-Kα radiation with mirror optics (λ = 1.54178 Å). All measurements were carried out at 100 K (unless otherwise noted) using an Oxford Cryosystems Cryostream 800 sample cryostat. Data collected on the Rigaku instrument were integrated using CrysAlisPro and corrected for absorption effects using a combination of empirical (ABSPACK) and numerical corrections. The structures were solved using SHELXT or SHELXS and refined by full-matrix least-squares analysis (SHELXL), using the program package OLEX2. Unless otherwise indicated below, all non-hydrogen atoms were refined anisotropically and hydrogen atoms were constrained to ideal geometries and refined with fixed isotropic displacement parameters (in terms of a riding model).

Optical Rotation: Optical Rotation were recorded on a Jasco P-2000 polarimeter using a 1.5-mL cell with 100 mm length (l). Values are reported in deg mL g-1 dm-1. Concentrations I are quoted in g/100mL (i.e. 10 mg/mL corresponds to c = 1.00)

2 Optimization of reaction conditions

Table S1. Screening of different catalysts and iodine sources.

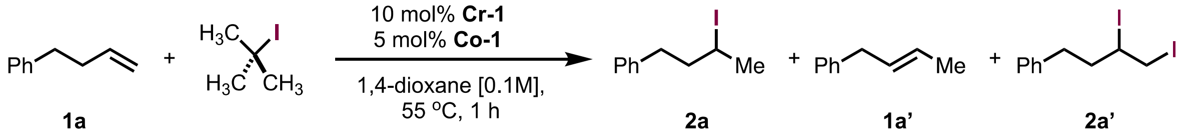

1a **2a** **1a’** **2a’**

entry	***deviation from standard conditions***	**2a (yield %)**	**1a’ (yield %)**	**2a’ (yield %)**
1	**none**	**91%**	**trace**	**0%**
2	450 nm light, 10 mol% $[FeCp(CO)_2]_2$ instead of **Cr-1**	15%	5%	4%
3	450 nm light, 10 mol%$Mn_2(CO)_{10}$ instead of **Cr-1**	82%	5%	7%
4	**Cr-2** instead of **Cr-1**	29%	trace	nd
5	$CrCl_2$ instead of **Cr-1**	trace	trace	nd
6	**Co-2** instead of **Co-1**	55%	10%	nd
7	**Co-3** instead of **Co-1**	trace	trace	nd
8	*i*-Pr iodide instead of *t*-Bu iodide	12%	trace	nd
9	ethyl iodide instead of *t*-Bu iodide	5%	0%	nd
10	no **Co-1**	7%	0%	nd
11	no **Cr-1**	0%	0%	nd
12	10 mol% of $PhSiH_3$ and *t*-BuOOH instead of **Cr-1**	27%	0%	nd
13	[1M], 10 mol% of $PhSiH_3$ and *t*-BuOOH instead of **Cr-1**	84%	0%	nd

Co-1: R = *t*-Bu
Co-2: R = OMe
Co-2: R = NO_2

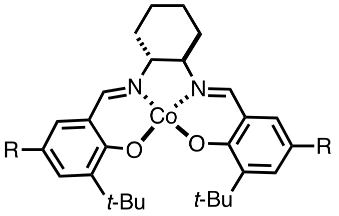

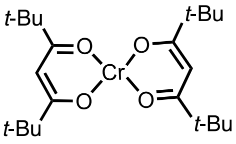

Cr-1

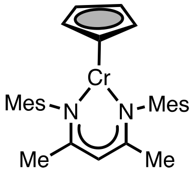

Cr-2

Table S2: Solvent screening with optimized catalysts

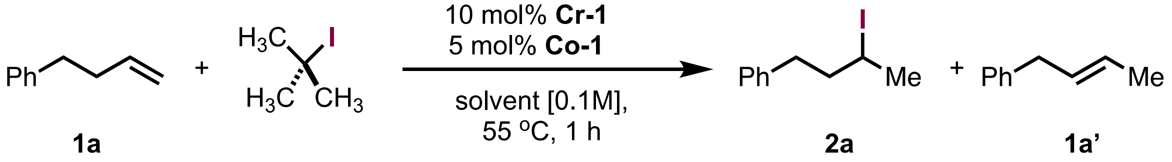

entry	*solvent*	**1a** [%]	**1a'** [%]	**2a** [%]
1	THF	2%	11%	72%
2	MeCN	40%	trace	8%
3	1,4-dioxane	0%	trace	91%
4	benzene	58%	trace	32%

3 Starting material synthesis

1-(But-3-en-1-yloxy)-4-iodobenzene (1w)

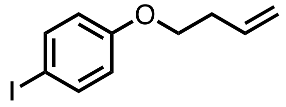

4-Iodophenol (2.000 g, 9.0 mmol), 4-bromo-1-butene (1.2 mL, 11.8 mmol), and K_2CO_3 (3.769 g, 27.3 mmol) were added to 30 mL of acetone and heated to 55 °C for 16 h. The reaction was cooled to room temperature and solvent was evaporated in vacuo. The crude material was purified by flash silica column chromatography using 15% Et_2O:pentanes to afford the product as a colorless oil (75% yield, 1.874 g).

^{1}H NMR (400 MHz, $CDCl_3$) δ 7.65–7.43 (m, 2H), 6.72–6.57 (m, 2H), 5.89 (ddt, *J* = 17.0, 10.3, 6.7 Hz, 1H), 5.34–4.71 (m, 2H), 3.97 (t, *J* = 6.7 Hz, 2H), 2.66–2.33 (m, 2H).

^{13}C NMR (101 MHz, $CDCl_3$) δ 158.9, 138.3, 134.3, 117.3, 117.1, 82.8, 67.4, 33.6.

HRMS ESI: MF $C_{10}H_{11}IO$ $[M+H]^+$ Calculated: 273.9849; Found: 273.9844.

Hex-5-en-1-yl 4-methylbenzenesulfonate (1t)

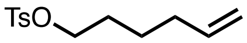

Hex-5-en-1-ol (2.000 g, 20 mmol) was dissolved in pyridine (6.5 mL, 80 mmol) and cooled to 0 °C. *p*-Toluenesulfonyl chloride (5.710 g, 30 mmol) was added in portions over 3 minutes and the reaction was stirred for 1 h at 0 °C. The crude material was directly loaded onto the column and purified by flash silica chromatography using 5% Et_2O:pentanes to afford the product as a colorless oil (70% yield, 3.565 g).

^{1}H NMR (400 MHz, $CDCl_3$) δ 7.94–7.61 (m, 2H), 7.43–7.29 (m, 2H), 5.72 (ddt, *J* = 17.0, 10.3, 6.7 Hz, 1H), 5.08–4.80 (m, 2H), 4.03 (t, *J* = 6.4 Hz, 2H), 2.45 (s, 3H), 2.00 (dtd, *J* = 8.6, 7.1, 6.6, 1.4 Hz, 2H), 1.65 (m, 2H), 1.51–1.31 (m, 2H).

^{13}C NMR (101 MHz, $CDCl_3$) δ 144.5, 137.7, 133.0, 129.6, 127.7, 114.9, 70.2, 32.7, 28.0, 24.4, 21.4.

HRMS ESI: MF $C_{13}H_{18}NaO_3S$ $[M+Na]^+$ Calculated: 277.0869; Found: 277.0871.

***N*-(4-((Trimethylsilyl)ethynyl)phenyl)hex-5-enamide (1e)**

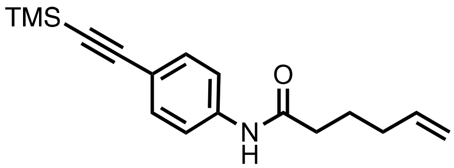

5-Hexenoic acid (362 mg, 3.17 mmol), EDC (790 mg, 4.12 mmol) and HOBt (631 mg, 4.12 mmol) were dissolved in DCM (10 mL). 4-((Trimethylsilyl)ethynyl)aniline (600 mg, 3.17 mmol) was added and the reaction was stirred for 16 h. The reaction was diluted with 10 mL of DCM and the organic layer was washed with aqueous saturated $NaHCO_3$ (2 x 10 mL), and then with brine (1 x 10 mL). The organic layer was dried with $MgSO_4$, filtered and concentrated. The residue was purified by

flash silica column chromatography in 10% EtOAc:pentane to afford a white solid (81% yield, 732 mg).

^{1}H NMR (400 MHz, $CDCl_3$) δ 7.60–7.32 (m, 4H), 7.21–7.05 (s, 1H), 5.94–5.58 (m, 1H), 5.19–4.78 (m, 2H), 2.35 (td, *J* = 7.6, 1.4 Hz, 2H), 2.15 (q, *J* = 7.2 Hz, 2H), 1.83 (dd, *J* = 8.3, 6.5 Hz, 2H), 0.24 (s, 9H).

^{13}C NMR (101 MHz, $CDCl_3$) δ 170.9, 138.7, 137.9, 132.9, 119.2, 115.7, 105.4, 39.6, 33.2, 26.6, -2.6. Alkyne carbons could not be located.

HRMS ESI: MF $C_{17}H_{24}NOSi$ $[M+H]^+$ Calculated: 286.1622; Found: 286.1624.

4-(4,4,5,5-Tetramethyl-1,3,2-dioxaborolan-2-yl)phenyl pent-4-enoate (1l)

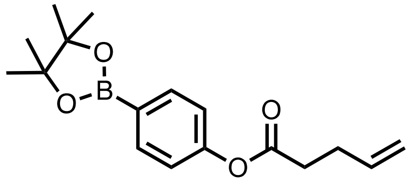

In a flask at 0 °C 4-pentenoic acid (0.72 mL, 7.0 mmol) and 4-hydroxyphenylboronicacidpinacolester (1.54 g, 7.0 mmol) were dissolved in DCM (10 mL). DCC (1.73 g, 8.40 mmol) was then added and the mixture was allowed to warm to room temperature and stirred for 16 h. After completion of the reaction the solid DCU was removed with filtration through celite with DCM and the organic layer was washed with aqueous saturated $NaHCO_3$ (2 x 10 mL) and then with brine (1 x 10 mL). The organic layer was dried with $MgSO_4$, filtered and concentrated under reduced pressure. The residue was purified through flash silica chromatography in 10% EtOAc:pentanes. The product was obtained as a white solid (62% yield, 1.32 g).

^{1}H NMR (400 MHz, $CDCl_3$): δ 7.85–7.80 (m, 2H), 7.10–7.06 (m, 2H), 5.90 (ddt, *J* = 16.8, 10.2, 6.4 Hz, 1H), 5.19 – 5.04 (m, 2H), 2.67 (ddd, *J* = 7.8, 7.1, 0.8 Hz, 2H), 2.55–2.46 (m, 2H), 1.34 (s, 12H).

^{13}C NMR (101 MHz, $CDCl_3$) δ 171.3, 153.2, 136.3, 136.2, 120.9, 115.9, 83.9, 33.7, 28.8, 24.8. The carbon attached to boron could not be found.

HRMS ESI MF: $[M+H]^+$ $C_{17}H_{24}BO_4$Calculated: 303.1762, Found: 303.1765.

Benzyl 2-((*tert*-butoxycarbonyl)amino)pent-4-enoate (1d)

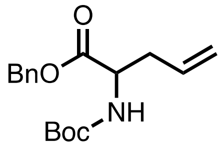

In a round-bottom flask cooled to 0 °C was added benzylic alcohol (193 µL, 1.86 mmol) and (±)-N-Boc allyl glycine (400 mg, 1.86 mmol) and DCM (10 mL). DCC (460 mg, 2.23 mmol) was then added and the mixture was stirred overnight at 25 °C. After completion of the reaction the solid DCU was removed with filtration through Celite with DCM and the organic layer was washed with aqueous saturated $NaHCO_3$ (2 x 10 mL) and then with brine (1 x 10 mL). The organic layer was dried over $MgSO_4$ and concentrated under reduced pressure. The residue was purified by column chromatography 10% EtOAc:pentane and product was obtained as a yellowish oil (41% yield, 233 mg).

^{1}H NMR (400 MHz, $CDCl_3$): δ 7.41–7.30 (m, 5H), 5.65 (ddt, *J* = 16.0, 11.1, 7.2 Hz, 1H), 5.24–4.99 (m, 5H), 4.42 (d, *J* = 7.3 Hz, 1H), 2.62–2.43 (m, 2H), 1.43 (s, 9H).

^{13}C NMR (101 MHz, $CDCl_3$) δ 172.0, 155.3, 135.5, 132.3, 128.7, 128.5, 128.5, 119.3, 80.0, 67.2, 53.1, 36.9, 28.4.

HRMS ESI: MF $[M+Na]^+$ $C_{17}H_{23}NNaO_4$ Calculated: 328.1519, Found: 328.1513.

4-Methylthiophenyl pent-4-enoate (1k)

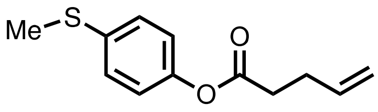

In a round-bottom flask cooled to 0 °C, 4-pentenoic acid (1.00 mL, 10.0 mmol) and 4-methylthiophenol (1.40 g, 10.0 mmol) were added in 15 mL of DCM. DCC (2.28 g, 12.0 mmol) was then added and the mixture was stirred for 16 h at 25 °C. After completion of the reaction the solid DCU was removed with filtration through Celite with DCM and the organic layer was washed with aqueous saturated $NaHCO_3$ (2 x 15 mL) and then with brine (1 x 15 mL). The organic layer was dried with $MgSO_4$ filtered and concentrated under reduced pressure. The residue was purified through flash silica column chromatography with 10% EtOAc:pentane. The product was obtained as yellowish oil (89% yield, 1.98 g).

^{1}H NMR (400 MHz, $CDCl_3$): δ 7.29–7.24 (m, 2H), 7.03–6.98 (m, 2H), 5.90 (ddt, *J* = 17.1, 10.2, 6.4 Hz, 1H), 5.18–5.04 (m, 2H), 2.66 (ddd, *J* = 7.8, 7.1, 0.8 Hz, 2H), 2.55–2.48 (m, 2H), 2.48 (s, 3H).

^{13}C NMR (101 MHz, $CDCl_3$): δ 171.5, 148.4, 136.3, 135.6, 128.1, 122.0, 115.9, 33.6, 28.9, 16.6.

HRMS ESI: MF $C_{12}H_{15}O_2S$ $[M+H]^+$ Calculated: 223.0787; Found: 223.0786.

3-(But-3-en-1-yloxy)benzaldehyde (1m)

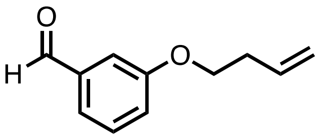

In a round-bottom flask cooled to 0 °C was added 3-hydroxybenzaldehyde (1.221 g, 10.0 mmol), DEAD (6.8 mL, 15 mmol, 2.2M in toluene), PPh_3 (3.934 g, 15.0 mmol) 3-buten-1-ol (1.29 mL, 15.0 mmol) in 30 ml of THF. After addition of all reagents, the reaction was allowed to warm to room temperature and stirred for 16 h. The reaction was concentrated under reduced pressure and the

residue was purified by flash silica column chromatography with 10% EtOAc:pentane, and the product was obtained as a colorless oil (65% yield, 1.142 g).

^{1}H NMR (400 MHz, $CDCl_3$) δ 9.97 (d, *J* = 1.9 Hz, 1H), 7.56–7.34 (m, 3H), 7.18 (dt, *J* = 6.5, 2.7 Hz, 1H), 5.90 (ddtd, *J* = 17.0, 10.3, 6.7, 1.7 Hz, 1H), 5.33–4.96 (m, 2H), 4.08 (td, *J* = 6.7, 2.0 Hz, 2H), 2.57 (qt, *J* = 6.7, 1.4 Hz, 2H).

^{13}C NMR (101 MHz, $CDCl_3$) δ 192.3, 159.6, 137.9, 134.2, 130.1, 123.6, 122.1, 117.2, 113.0, 67.6, 33.6.

HRMS ESI Calculated: m/z: $C_{12}H_{15}O_2$ $[M+H]^+$ Calculated 191.1067; Found 191.1072.

4 Substrate scope

General procedure A

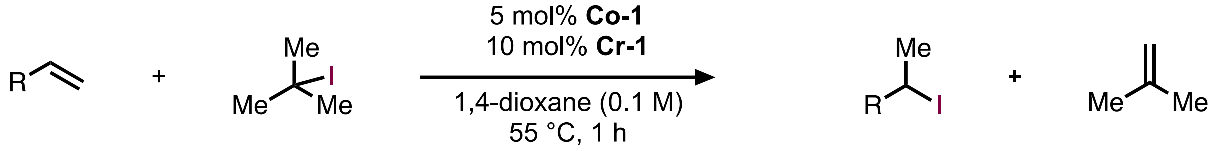

1

2

Inside an argon-filled glovebox, to a 2-dram vial equipped with a magnetic stirring bar, **Co-1** (3.0 mg, 5.0 µmol), **Cr-1** (4.2 mg, 10 µmol), and the given substrate (0.100 mmol) were dissolved in 1,4-dioxane (1 mL). *t*-Butyl iodide (37 µL, 0.300 mmol) was added and the vial was sealed and removed from the glovebox. The reaction mixture was stirred at 55 °C for 0.25–1 h, then the solution was filtered through a silica plug and washed with pentanes (4 mL) and Et_2O (4 mL). The solvent was removed *in vacuo* and the crude product was dissolved in $CDCl_3$ and internal standard and the crude yield was calculated. The material was purified by flash silica column chromatography or preparative TLC.

General procedure B

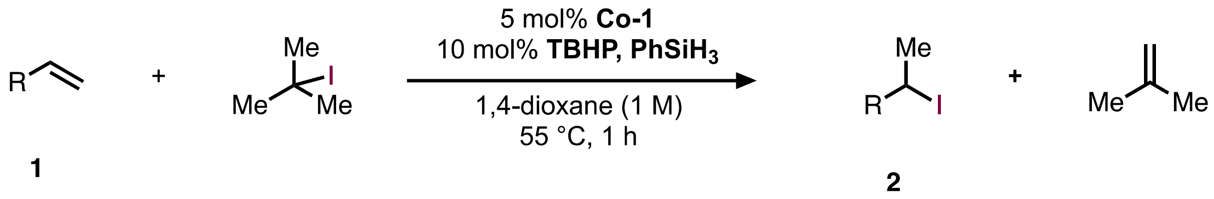

Inside an argon-filled glovebox, to a 2-dram vial equipped with a magnetic stirring bar, **Co-1** (3.0 mg, 5.0 µmol), and the given substrate (0.100 mmol) were dissolved in 1,4-dioxane (0.1 mL). *t*-Butyl iodide (36.8 µL, 0.300 mmol) was added and the vial was sealed and removed from the glovebox. Non-degassed TBHP (1.8 µL, 0.0100 mmol, 6.6 M in hexane) was added by Hamilton syringe directly through a puncturable cap and the vial was shaken. This results immediately in a color change from a red to brown solution. ~30 seconds after adding TBHP, non-degassed phenylsilane (1.2 µL, 0.0100 mmol) was added by Hamilton syringe directly through a puncturable cap. The reaction mixture was stirred at 55 °C for 0.25–1 h, then solution was filtered through silica and washed with pentanes (4 mL) and diethyl Et_2O (4 mL). The solvent was removed *in vacuo* and the crude product was dissolved in $CDCl_3$ and internal standard and the crude yield was calculated. The material was purified by flash silica column chromatography or preparative TLC.

General Procedure B2 (No glovebox)

Using a modified procedure from General Procedure B, alkene (1.0 equivalent), tert-butyl iodide (3.0 equivalents), 1,4-dioxane [1 M] and Co-1 (5 mol%) were added under air to an appropriately sized Schlenk tube (>1/2 the volume of the reaction). The tube was closed and freeze-pumped-thawed under N_2 three times. Then, through a septum, non-degassed t-BuOOH (6.6M in hexanes) (10 mol%) was added resulting in a brown solution followed ~30 seconds later by non-degassed $PhSiH_3$ (10 mol%). The reaction was heated to 55 °C under a continuous stream of N_2 for the allotted time, then cooled to room temperature. The solvent was removed in vaccuo and the reaction diluted with pentanes and a saturated solution of sodium thiosulfate. The aqueous phase was extracted with pentanes (3x), dried over $MgSO_4$ then solvent evaporated. The crude oil was purified by flash silica column chromatography using 100% pentanes.

General note: we found general procedure B simpler for reaction setup on small scale and thus the results from Table 1 reflects using this protocol. General Procedure B2 was used for all applications >1 mmol and did not appear to give different results than General Procedure B.

(3-iodobutyl)benzene (2a)

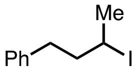

2a was prepared according to the general procedures using 4-phenyl-1-butene (15.0 µL, 0.100 mmol). Using the reaction from procedure A, flash silica column chromatography in 100% pentanes yielded the product as colorless oil (21 mg, 81%).

^{1}H NMR (400 MHz, $CDCl_3$) δ = 7.34–7.25 (m, 2H), 7.25–7.16 (m, 3H), 4.12 (dqd, J = 9.1, 6.8, 4.5 Hz, 1H), 2.85 (ddd, J = 14.0, 9.1, 5.2 Hz, 1H), 2.70 (ddd, J = 13.8, 9.0, 6.9 Hz, 1H), 2.25–2.09 (m, 1H), 1.95 (d, J = 6.9 Hz, 3H), 1.89 (m, 1H).

^{13}C NMR (101 MHz, $CDCl_3$) δ = 140.8, 128.53, 128.50, 126.1, 44.4, 35.8, 29.6, 29.00.

HRMS ESI: MF $[M+H]^+$ $C_{10}H_{13}I$ Calculated: 260.0056, Found: 260.0055.

Optical Rotation: [α] = 0.00° (c = 0.5, $CHCl_3$)

2-(4-iodopentyl)isoindoline-1,3-dione (2b)

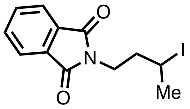

2b was prepared according to the general procedure using 2-(3-iodobutyl)isoindoline-1,3-dione (20.0 mg, 0.100 mmol). Using the reaction from procedure A, flash silica column chromatography in 100% pentanes yielding the product as a colorless oil (32.5 mg, 99%).

^{1}H NMR (400 MHz, $CDCl_3$) δ = 7.36–7.27 (m, 2H), 7.25–7.16 (m, 2H), 4.19–4.03 (m, 1H), 2.85 (ddd, J = 14.0, 9.0, 5.2 Hz, 1H), 2.70 (ddd, J = 13.8, 9.0, 6.9 Hz, 1H), 2.16 (dtd, J = 14.3, 9.0, 5.2 Hz, 1H), 1.96 (d, J = 6.8 Hz, 3H), 1.89 (m, 1H).

^{13}C NMR (126 MHz, $CDCl_3$) δ 168.6, 134.1, 132.2, 123.4, 39.9, 37.1, 29.1, 28.9.

HRMS ESI: MF $[M+H]^+$ $C_{12}H_{15}O_2NI$ Calculated: 344.0142, Found: 344.0144.

_N_-(4-iodopentyl)-4-methylbenzenesulfonamide (2c)

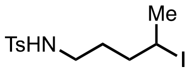

2c was prepared according to the general procedure using 4-methyl-*N*-(pent-4-en-1-yl)benzenesulfonamide (23.9 mg, 0.100 mmol). Using the reaction from procedure A, flash silica column chromatography in 30% Et_2O:pentanes yielded the product as colorless oil (20.0 mg, 54%).

^{1}H NMR (400 MHz, $CDCl_3$) δ = 7.78–7.71 (m, 2H), 7.35–7.28 (m, 2H), 4.48 (t, J = 6.3 Hz, 1H), 4.15–4.02 (m, 1H), 2.97 (q, J = 6.5 Hz, 2H), 2.43 (s, 3H), 1.93–1.44 (m, 6H).

^{13}C NMR (101 MHz, $CDCl_3$) δ = 143.7, 136.9, 129.9, 127.2, 42.4, 39.6, 30.0, 29.0, 29.0, 21.7.

HRMS ESI: MF [M+H]+ $C_{12}H_{19}NO_2SI$ Calculated: 368.0176, Found: 368.0181.

Benzyl 2-((*tert*-butoxycarbonyl)amino)-4-iodopentanoate (2d)

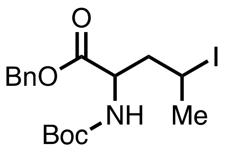

2d was prepared according to the general procedure using (±)-benzyl 2-((*tert*-butoxycarbonyl)amino)pent-4-enoate (30.5 mg, 0.100 mmol). Using the reaction from procedure A, flash silica column chromatography in 15% EtOAc:pentanes yielded the product as a colorless oil (26.0 mg, 60%, 1.9:1 d.r.). The analytic data is reported for the diastereomer mixture with assignments for major and minor diastereomers when possible.

^{1}H NMR (500 MHz, $CDCl_3$) δ 7.45–7.27 (m, 5H), 5.36–5.15 (m, 2H), 5.15–5.01 (m, 1H), 4.43 (s, 1H), 4.17 (q, *J* = 5.4 Hz, 1H), 2.40 (m, 1H, minor), 2.31 (m, 1H, major), 2.01–1.83 (m, 3H), 1.44 (s, 9H).

^{13}C NMR (126 MHz, $CDCl_3$) δ 171.9 (minor), 171.5 (major), 155.3, 128.67 (major or minor), 128.65 (major or minor), 128.60 (major or minor), 128.56 (major or minor), 128.5 (major or minor), 128.4 (major or minor), 128.3 (major or minor), 80.30, 67.4 (major), 67.3 (minor), 54.4 (minor), 53.2 (major), 46.0 (major), 45.2 (minor), 28.9 (major), 28.3, 28.2 (minor), 23.6 (minor), 21.3 (major).

HRMS ESI: MF $[M+Na]^+$ $C_{17}H_{24}IO_4NNa$ Calculated: 456.0642, Found: 456.0636.

5-Iodo-*N*-(4-((trimethylsilyl)ethynyl)phenyl)hexanamide (2e)

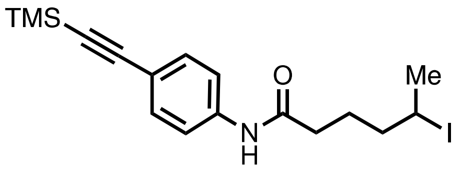

2e was prepared according to the general procedure using *N*-(4-((trimethylsilyl)ethynyl)phenyl)hex-5-enamide (28.2 mg, 0.100 mmol). Using the reaction from procedure A, Prepartive TLC in 40% Et_2O:pentanes yielded the product as a colorless oil (15.1 mg, 36%).

^{1}H NMR (500 MHz, $CDCl_3$) δ 7.52–7.36 (m, 4H), 7.13 (s, 1H), 4.20 (m, 1H), 2.39 (t, *J* = 7.0 Hz, 2H), 1.94 (d, *J* = 6.8 Hz, 3H), 1.91–1.66 (m, 4H), 0.24 (d, *J* = 0.6 Hz, 9H).

^{13}C NMR (126 MHz, $CDCl_3$) δ 170.5, 138.1, 133.0, 119.2, 104.9, 93.9, 42.1, 36.7, 29.5, 29.0, 25.7, 0.1. One of the alkynyl carbons could not be located.

HRMS ESI: MF $[M+H]^+$ $C_{17}H_{26}NOSiI$ Calculated: 414.0745, Found: 414.0741.

Ethyl 4-iodopentanoate (2f)

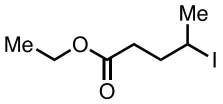

2f was prepared according to the general procedure using ethyl pent-4-enoate (14.2 µL, 0.100 mmol). Using the reaction from procedure A, flash silica column chromatography in 100% pentanes yielded the product as colorless oil (15.0 mg, 60%). The analytical data matches the reported data.[3]

^{1}H NMR (400 MHz, $CDCl_3$) δ = 4.20 (m, 1H), 4.14 (d, J = 7.1 Hz, 2H), 2.60–2.37 (m, 2H), 2.13–1.97 (m, 2H), 1.94 (d, J = 6.9 Hz, 3H), 1.26 (t, J = 7.1 Hz, 3H).

^{13}C NMR (101 MHz, $CDCl_3$) δ = 172.7, 60.7, 37.7, 34.7, 29.0, 28.7, 14.3.

2-Iodopropyl pivalate (2g)

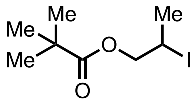

2-iodopropyl pivalate was prepared according to the general procedure using allyl pivalate (14.2 mg, 0.100 mmol). Using the reaction from procedure A, flash silica column chromatography in 100% pentanes yielded the product as colorless oil (13.5 mg, 50%).

^{1}H NMR (500 MHz, $CDCl_3$) δ 4.30–4.18 (m, 2H), 4.18–4.10 (m, 1H), 1.88 (d, J = 6.4 Hz, 3H), 1.23 (d, J = 2.4 Hz, 9H).

^{13}C NMR (126 MHz, $CDCl_3$) δ 177.9, 70.4, 29.8, 29.7, 27.4, 27.3.

HRMS ESI MF [M+ I–] $C_8H_{16}O_2$ Calculated: 143.1067, Found: 143.1066.

3-((4-Iodopentyl)oxy)oxetane (2h)

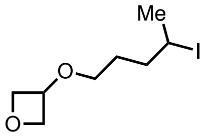

2h was prepared according to the general procedures using 3-(pent-4-en-1-yloxy)oxetane (14.2 mg, 0.100 mmol). Using the reaction from procedure A, flash silica column chromatography in 100% pentanes yielded the product as colorless oil (10.8 mg, 40%).

^{1}H NMR (400 MHz, $CDCl_3$) δ = 4.76 (td, J = 6.3, 1.8 Hz, 2H), 4.64–4.56 (m, 3H), 4.60–4.49 (m, 1H), 3.42–3.32 (m, 1H), 1.95 (dd, J = 6.2, 1.6 Hz, 4H), 1.85–1.74 (m, 1H), 1.78–1.60 (m, 1H), 1.56 (dd, J = 3.3, 1.7 Hz, 2H).

^{13}C NMR (101 MHz, $CDCl_3$) δ = 78.9, 72.4, 67.9, 39.6, 30.2, 29.9, 29.1.

HRMS ESI: MF [M+Na]+ $C_8H_{15}O_2INa$, Calculated: 293.0009, Found: 293.0008.

***Tert*-butyl(3-iodobutoxy)dimethylsilane (2i)**

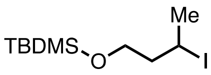

2i was prepared according to the general procedure using (but-3-en-1-yloxy)(*tert*-butyl)dimethylsilane (18.6 mg, 0.100 mmol). Using the reaction from procedure A, flash silica column chromatography in 15% Et_2O:pentanes yielded the product as colorless oil (26.6 mg, 85%). The analytical data matches the literature values.[4]

^{1}H NMR (400 MHz, $CDCl_3$) δ = 4.35 (dqd, J = 9.5, 6.9, 4.4 Hz, 1H), 3.76 (ddd, J = 10.3, 5.7, 4.6 Hz, 1H), 3.65 (ddd, J = 10.3, 8.1, 4.8 Hz, 1H), 2.06–1.93 (m, 4H), 1.79 (m, 1H), 0.90 (s, 9H), 0.07 (d, J = 3.8 Hz, 6H).

^{13}C NMR (101 MHz, $CDCl_3$) δ = 62.8, 45.6, 29.3, 26.9, 26.1, 18.4, -5.1.

5-Iodohexan-1-ol (2j)

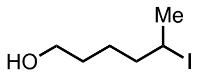

2j was prepared according to the general procedure using hex-5-en-1-ol (10.0 mg, 0.100 mmol). Using the reaction from procedure A, Preparative TLC in 50% Et_2O:pentanes yielded the product as colorless oil (11.4 mg, 47%). The analytical data matches the reported data.[5]

^{1}H NMR (400 MHz, $CDCl_3$) δ = 4.19 (dqd, J = 8.6, 6.8, 4.7 Hz, 1H), 3.66 (t, J = 6.0 Hz, 2H), 1.93 (d, J = 6.8 Hz, 3H), 1.93 – 1.79 (m, 1H), 1.75–1.15 (m, 5H).

^{13}C NMR (101 MHz, $CDCl_3$) δ = 62.8, 42.7, 31.9, 30.4, 29.0, 26.2.

4-Methylthiophenyl 4-iodopentanoate (2k)

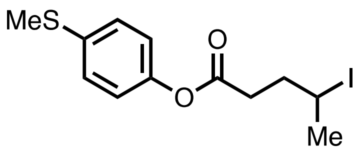

2k was prepared according to the general procedure using 4-methylthiophenyl pent-4-enoate (22.2 mg, 0.100 mmol). Using the reaction from procedure B, preparative TLC in 5% EtOAc:pentanes yielded the product as a colorless oil (20.3 mg, 60%).

^{1}H NMR (400 MHz, $CDCl_3$) δ 7.36–7.19 (m, 2H), 7.11–6.95 (m, 2H), 4.27 (dqd, J = 9.1, 6.8, 4.4 Hz, 1H), 2.90–2.62 (m, 2H), 2.48 (s, 3H), 2.24–2.03 (m, 2H), 1.99 (d, J = 6.9 Hz, 3H).

^{13}C NMR (101 MHz, $CDCl_3$) δ 171.2, 148.4, 135.9, 128.2, 122.1, 37.5, 34.8, 29.0, 28.2, 16.6.

HRMS ESI: MF $[M+Na]^+$ $C_{12}H_{15}IO_2SNa$ Calculated: 372.9730, Found: 372.9729.

4-(4,4,5,5-Tetramethyl-1,3,2-dioxaborolan-2-yl)phenyl 4-iodopentanoate (2l)

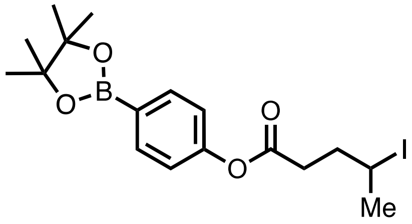

2l was prepared according to the general procedure using 4,4,5,5-tetramethyl-1,3,2-dioxaborolan-2-yl)phenyl pent-4-enoate (30.2 mg, 0.100 mmol). Using the reaction from procedure A, preparative TLC in 5% EtOAc:pentanes yielded the product as colorless oil (22.5 mg, 52%).

^{1}H NMR (400 MHz, $CDCl_3$) δ 7.84 (d, J = 8.5 Hz, 2H), 7.10 (d, J = 8.6 Hz, 2H), 4.28 (dqd, J = 9.0, 6.8, 4.4 Hz, 1H), 2.94–2.61 (m, 2H), 2.28–2.04 (m, 2H), 1.99 (d, J = 6.8 Hz, 3H), 1.34 (s 12H).

^{13}C NMR (101 MHz, $CDCl_3$) δ 170.9, 153.2, 136.3, 120.9, 84.0, 83.9, 37.5, 34.9, 29.0, 28.2, 24.9.

^{11}B NMR (160 MHz, $CDCl_3$) δ 30.4.

HRMS ESI: MF $[M+Na]^+$ $C_{17}H_{24}IO_4BNa$ Calculated: 453.0705, Found: 453.0704.

3-(But-3-en-1-yloxy)benzaldehyde (2m)

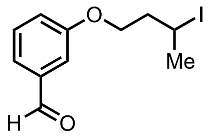

2m was prepared according to the general procedure using 3-(3-iodobutoxy)benzaldehyde (17.6 mg, 0.100 mmol). Using the reaction from procedure A, preparative TLC in 10% Et_2O:pentanes yielded the product as colorless oil (13.9 mg, 46%).

^{1}H NMR (400 MHz, $CDCl_3$) δ = 9.98 (s, 1H), 7.51–7.27 (m, 3H), 7.23–7.13 (m, 1H), 4.43 (dqd, J = 9.6, 6.9, 4.4 Hz, 1H), 4.29–4.04 (m, 2H), 2.26 (ddt, J = 14.5, 9.7, 4.9 Hz, 1H), 2.18–2.07 (m, 1H), 2.03 (d, J = 6.9 Hz, 3H).

^{13}C NMR (101 MHz, $CDCl_3$) δ = 192.2, 159.4, 137.9, 130.2, 123.8, 121.9, 113.0, 68.0, 41.9, 29.2, 25.1.

HRMS ESI: MF $[M+H]^+$ $C_{11}H_{14}IO_2$: Calculated: 305.0033, Found: 305.0035.

1,2,3,4,5-Pentafluoro-6-(2-iodopropyl)benzene (2n)

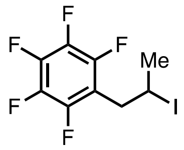

2n was prepared according to the general procedure using 1,2,3,4,5-pentafluoro-6-(2-iodopropyl)benzene (20.8 mg, 0.100 mmol). Using the reaction from procedure A, preparative TLC 1% Et_2O:pentanes yielded the product as colorless oil (20.1 mg, 60%). The perfluoroarene appears as a complex set of short multiplets in the ^{13}C NMR and their ppm and splitting values are not assigned.

^{1}H NMR (400 MHz, $CDCl_3$) δ 4.47–4.23 (m, 1H), 3.48–3.29 (m, 1H), 3.19 (dd, *J* = 14.4, 6.9 Hz, 1H), 1.95 (dt, *J* = 6.9, 0.7 Hz, 3H).

^{19}F NMR (376 MHz, CDCl3) δ –142.3 – –142.4 (m), –155.4 (t, J = 20.8 Hz), –161.8 – –162.0.

HRMS ESI: MF [M-I+H] $C_9H_7F_5$ Calculated: 209.0384, Found: 209.0384 and MF: [-M+I] Calculated: 126.9039, Found: 126.9041.

5-(2-Iodopropyl)benzo[*d*][1,3]dioxole (2o)

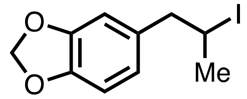

2o was prepared according to the general procedure using safrole (16.2 mg, 0.100 mmol). Using the reaction from procedure A, preparative TLC 20% Et_2O:pentanes yielded the product as colorless oil (17.2 mg, 60%).

^{1}H NMR (500 MHz, $CDCl_3$) δ 6.75 (dd, *J* = 7.9, 0.4 Hz, 1H), 6.69–6.58 (m, 2H), 5.94 (s, 2H), 4.28 (ddt, *J* = 14.2, 7.4, 6.8 Hz, 1H), 3.33–3.12 (m, 1H), 2.96 (dd, *J* = 14.2, 7.5 Hz, 1H), 1.89 (d, *J* = 6.8 Hz, 3H).

^{13}C NMR (126 MHz, $CDCl_3$) δ 147.8, 146.5, 133.7, 122.2, 109.4, 108.3, 101.1, 49.3, 29.0, 28.1.

HRMS ESI: MF [M+H] $C_{10}H_{11}O_2I$ Calculated: 289.9798, Found: 289.9793.

5-((5-Iodohexyl)sulfonyl)-1-phenyl-1*H*-tetrazole (2p)

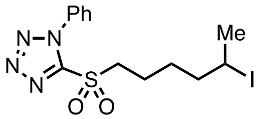

2p was prepared according to the general procedure using 5-(hex-5-en-1-ylsulfonyl)-1-phenyl-1*H*-tetrazole (29.2 mg, 0.100 mmol). Using the reaction from procedure A, preparative TLC with 40% Et_2O:pentanes yielded the product as colorless oil (26.0 mg, 62%). After prolonged storage in the freezer (~1 year) at –40 °C, single crystals suitable for X-ray diffraction were formed.

^{1}H NMR (400 MHz, $CDCl_3$) δ = 7.75–7.57 (m, 5H), 4.15 (ddd, J = 8.7, 6.8, 4.2 Hz, 1H), 3.76 (ddd, J = 8.6, 7.1, 1.4 Hz, 2H), 2.11–1.81 (m, 6H), 1.80–1.54 (m, 3H).

^{13}C NMR (101 MHz, $CDCl_3$) δ = 153.5, 133.1, 131.6, 129.9, 125.2, 55.9, 41.9, 29.0, 28.7, 28.5, 21.5.

HRMS ESI MF [M+Na]+ $C_{13}H_{17}IO_2N_4Na$: [M+H]+ Calculated: 443.0009, Found: 443.0010.

X-ray CCDC: 2306574.

4,6-dichloro-5-(2-iodopropyl)pyrimidine (2q)

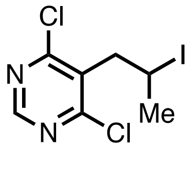

2q was prepared according to the general procedure using 5-allyl-4,6-dichloropyrimidine (18.9 mg, 0.100 mmol). Using the reaction from procedure A, preparative TLC with 15% EtOAc:pentanes yielded the product as colorless oil (18.5 mg, 58%).

^{1}H NMR (400 MHz, $CDCl_3$) δ 8.70 (s, 1H), 4.69–4.41 (m, 1H), 3.59 (dd, *J* = 14.3, 7.9 Hz, 1H), 3.43 (dd, *J* = 14.3, 7.5 Hz, 1H), 1.98 (d, *J* = 6.8 Hz, 3H).

^{13}C NMR (101 MHz, $CDCl_3$) δ 162.3, 156.4, 131.4, 42.5, 28.4, 20.1.

HRMS ESI: MF $[M+H]^+$ $C_7H_8N_2Cl_2I$ Calculated: 316.9104, Found: 316.9104.

1-(*Tert*-butyldimethylsilyl)-5-(2-iodopropoxy)-1*H*-indole (2r)

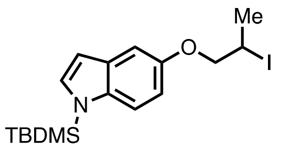

2r was prepared according to the general procedure using 5-(allyloxy)-1-(*tert*-butyldimethylsilyl)-1*H*-indole (28.8 mg, 0.100 mmol). Using the reaction from procedure A, preparative thin layer chromatograph in 50% DCM:pentanes yielded the product as colorless oil (13.0 mg, 32%).

^{1}H NMR (500 MHz, $CDCl_3$) δ = 7.39 (m, 1H), 7.16 (dd, J = 3.2, 0.5 Hz, 1H), 7.09 (dd, J = 2.5, 0.5 Hz, 1H), 6.82 (ddd, J = 8.9, 2.6, 0.5 Hz, 1H), 6.53 (dd, J = 3.1, 0.9 Hz, 1H), 4.40 (dqd, J = 7.9, 6.8, 5.6 Hz, 1H), 4.30 (dd, J = 10.2, 5.6 Hz, 1H), 4.07 (dd, J = 10.2, 8.0 Hz, 1H), 2.01 (d, J = 6.8 Hz, 3H), 0.92 (s, 9H), 0.58 (s, 6H).

^{13}C NMR (126 MHz, $CDCl_3$) δ = 152.5, 136.6, 132.1, 132.0, 114.7, 112.1, 104.7, 104.5, 76.0, 26.5, 24.9, 23.4, 19.6, -3.9.

HRMS ESI: MF $[M+Na]^+$ $C_{17}H_{26}IONSiNa$ Calculated: 438.0721, Found: 438.0719.

1-(2-Iodopropyl)-1*H*-indole (2s)

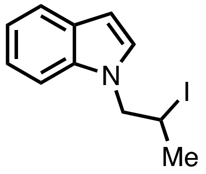

2s was prepared according to the general procedure using 1-allyl-1*H*-indole (15.7 mg, 0.100 mmol). Using the reaction from procedure A, preparative TLC in 5% Et_2O:pentanes yielded the product as colorless oil (21.3 mg, 62%).

^{1}H NMR (400 MHz, $CDCl_3$) δ 7.63 (ddd, *J* = 7.9, 1.2, 0.8 Hz, 1H), 7.35–7.30 (m, 1H), 7.25–7.20 (m, 1H), 7.16–7.09 (m, 2H), 6.52 (dd, *J* = 3.2, 0.9 Hz, 1H), 4.65 (dd, *J* = 14.5, 5.7 Hz, 1H), 4.52–4.41 (m, 1H), 4.31 (dd, *J* = 14.4, 9.1 Hz, 1H), 1.79 (d, *J* = 6.8 Hz, 3H).

^{13}C NMR (101 MHz, $CDCl_3$) δ 139.2, 134.5, 127.9, 121.9, 121.2, 119.8, 109.2, 101.9, 56.4, 25.7, 23.0.

HRMS ESI: MF $[M+H]^+$ $C_{11}H_{13}NI$ Calculated: 286.0087, Found: 286.0086.

5-Iodohexyl 4-methylbenzenesulfonate (2t)

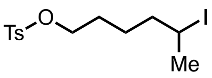

2t was prepared according to the general procedure using hex-5-en-1-yl 4-methylbenzenesulfonate (25.4 mg, 0.100 mmol). Using the reaction from procedure A, flash silica column chromatography in 5% Et_2O:pentanes yielded the product as colorless oil (32.4 mg, 85%).

^{1}**H NMR** (400 MHz, $CDCl_3$) δ = 7.83–7.75 (m, 2H), 7.39–7.31 (m, 2H), 4.09 (m, 1H), 4.03 (t, J = 6.4 Hz, 2H), 2.45 (s, 3H), 1.88 (d, J = 6.8 Hz, 3H), 1.77–1.27 (m, 6H).

^{13}**C NMR** (101 MHz, $CDCl_3$) δ = 144.9, 133.2, 130.0, 128.0, 70.3, 42.1, 29.5, 28.9, 28.1, 25.8, 21.8.

HRMS ESI: MF $[M+Na]^+$ $C_{13}H_{19}IO_3SNa$ Calculated: 404.9992, Found: 404.9994.

1-Chloro-5-iodohexane (2u)

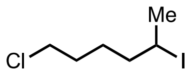

2u was prepared according to the general procedure using 6-chlorohex-1-ene (13.1 µL, 0.100 mmol). Using the reaction from procedure A, flash silica column chromatography in 100% pentanes yielded the product as colorless oil (24.4 mg, 99%). The analytical data matches the reported data.[6]

^{1}**H NMR** (400 MHz, $CDCl_3$) δ = 4.18 (dqd, J = 8.7, 6.8, 4.5 Hz, 1H), 3.55 (t, J = 6.6 Hz, 2H), 1.93 (d, J = 6.8 Hz, 3H), 1.92–1.51 (m, 6H).

^{13}**C NMR** (101 MHz, $CDCl_3$) δ = 44.7, 42.0, 31.7, 29.6, 28.9, 27.1.

1-Iodo-5-iodohexane (2v)

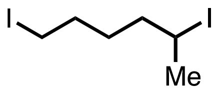

2v was prepared according to the general procedure using 6-iodohex-1-ene (21.0 mg, 0.100 mmol). Using the reaction from procedure A, flash silica column chromatography in 100% pentanes yielded the product as colorless oil (22.2 mg, 65%).

^{1}H NMR (500 MHz, $CDCl_3$) δ 4.27–4.12 (m, 1H), 3.22 (t, *J* = 7.0 Hz, 2H), 1.95 (d, *J* = 6.8 Hz, 3H), 1.90–1.79 (m, 2H), 1.73–1.46 (m, 4H).

^{13}C NMR (126 MHz, $CDCl_3$) δ 41.8, 32.7, 30.8, 29.6, 29.0, 6.4.

HRMS ESI: MF $[M+H]^+$ $C_{16}H_{13}I_2$ Calculated: 337.9023, Found: 337.9016.

1-Iodo-4-(3-iodobutoxy)benzene (2w)

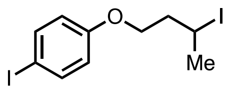

2-w was prepared according to the general procedure using 1-(but-3-en-1-yloxy)-4-iodobenzene (27.4 mg, 0.100 mmol, 1 equiv.). Using the reaction from procedure A, flash silica column chromatography in 10% Et_2O:pentanes yielded the product as colorless oil (32.1 mg, 80%).

^{1}H NMR (400 MHz, $CDCl_3$) δ = 7.60–7.51 (m, 2H), 6.73–6.64 (m, 2H), 4.47–4.33 (m, 1H), 4.13–3.96 (m, 2H), 2.29–2.14 (m, 1H), 2.14–2.01 (m, 1H), 2.01 (dd, J = 6.9, 0.9 Hz, 3H).

^{13}C NMR (101 MHz, $CDCl_3$) δ = 158.7, 138.4, 117.1, 83.1, 67.9, 41.9, 29.2, 25.3.

HRMS ESI: MF $C_8H_{13}I_2O$ $[M+H]^+$ Calculated: 401.8972, Found: 401.8972.

(*E*)-6-(3-((4-iodopentyl)oxy)but-1-en-1-yl)-1,5,5-trimethylcyclohex-1-ene (2x)

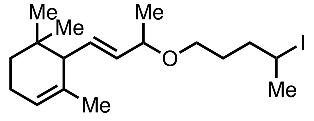

2x was prepared according to the general procedure using (±)-(*E*)-1,5,5-trimethyl-6-(3-(pent-4-en-1-yloxy)but-1-en-1-yl)cyclohex-1-ene (26.2 mg, 0.100 mmol). Using the reaction from

procedure A, preparative TLC in 5% EtOAc:pentanes yielded the product as colorless oil (19.5 mg, 50%, 1:1 d.r.). The product was isolated as diastereomeric mixture.

^{1}H NMR (500 MHz, $CDCl_3$) δ = 5.43–5.38 (m, 1H), 5.41–5.33 (m, 1H), 5.37–5.25 (m, 1H), 4.20 (m, 1H), 3.78 (m, 1H), 3.47 (dtt, J = 9.6, 7.2, 5.7 Hz, 1H), 3.33–3.23 (m, 1H), 2.10 (d, J = 9.1 Hz, 1H), 2.04–1.96 (m, 3H), 1.93 (ddd, J = 6.8, 1.5, 0.8 Hz, 3H), 1.91–1.80 (m, 1H), 1.80–1.69 (m, 1H), 1.68–1.58 (m, 2H), 1.60–1.54 (m, 2H), 1.47–1.36 (m, 1H), 1.26–1.18 (m, 4H), 0.89 (d, J = 4.9 Hz, 3H), 0.82 (d, J = 18.0 Hz, 3H).

^{13}C NMR (126 MHz, $CDCl_3$) δ = 134.2, 133.4, 121.1, 76.7, 67.2, 54.2, 39.9, 31.8, 31.7, 30.5, 30.3, 29.1, 27.9, 27.6, 27.1, 23.2, 22.9, 22.2.

HRMS ESI: MF $[M+Na]^+$ $C_{18}H_{32}IONa$ Calculated: 413.1312, Found: 413.1314.

Iodo-FK506 (2y)

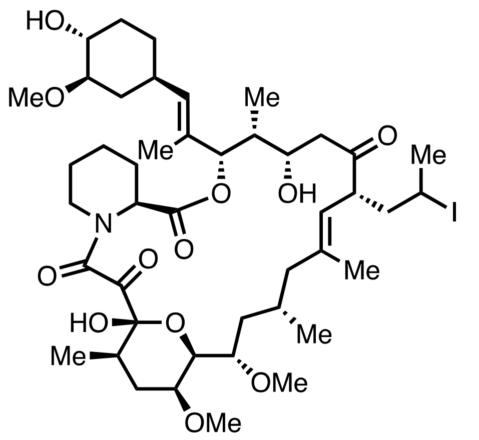

2x was prepared according to a modified general procedure A using FK506 (160.8 mg, 0.200 mmol), **Co-1** (24.1 mg, .04 mmol), **Cr-1** (25.2 mg, 0.06 mmol), *t*-Bu–I (72 μL, 0.600 mmol). Using the reaction from procedure A, flash silica column chromatography from 30–50% acteone:pentanes yielded the product as yellow foam (90.1 mg, 48%). The product was isolated as diastereomeric mixture. The rearrangement depicted below results in two different isomers in solution.[7] The two isomers and two diastereomers results in complex NMR spectra which are reported without assigning to the isomer.

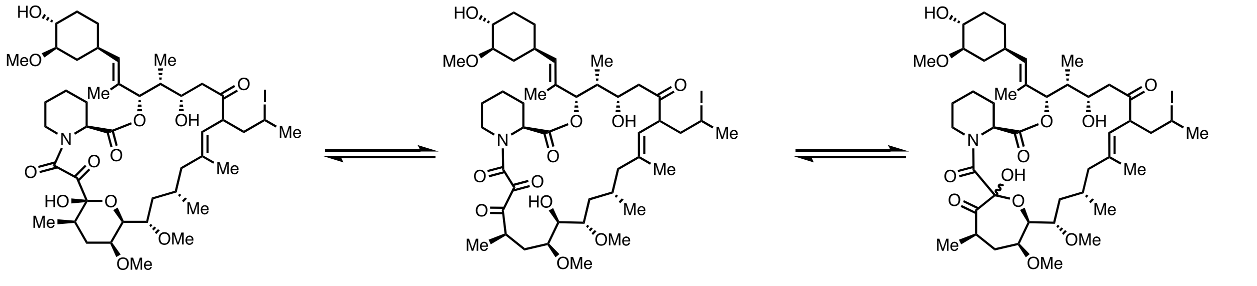

^{1}H NMR (400 MHz, $CDCl_3$) δ 5.53 (d, *J* = 6.2 Hz, 1H), 5.41 (d, *J* = 5.8 Hz, 1H), 5.30 (s, 1H), 5.17 (d, *J* = 16.9 Hz, 1H), 5.10–4.90 (m, 5H), 4.83–4.61 (m, 2H), 4.45 (dd, *J* = 31.4, 15.8 Hz, 4H), 4.15 (d, *J* = 14.3 Hz, 1H), 4.01–3.78 (m, 2H), 3.78–3.65 (m, 2H), 3.67–3.51 (m, 2H), 3.49–3.20 (m, 15H), 3.16 (s, 1H), 3.00 (m, 3H), 2.91–2.64 (m, 5H), 2.46–2.21 (m, 6H), 2.13–1.95 (m, 1H), 1.85–1.52 (5H), 1.52–1.14 (m, 5H), 1.14–0.72 (m, 7H).

^{13}C NMR (126 MHz, $CDCl_3$) δ 213.0, 211.9, 196.1, 195.6, 168.9, 168.8, 165.4, 164.3, 140.3, 139.6, 132.5, 131.7, 129.8, 129.6, 122.0, 121.8, 116.7, 98.7, 97.3, 84.2, 75.1, 73.6, 73.6, 73.5,

72.7, 72.6, 70.3, 57.0, 56.6, 56.5, 56.3, 53.5, 48.4, 45.4, 44.4, 43.7, 43.6, 43.6, 43.4, 40.0, 39.6, 35.0, 34.9, 34.8, 32.6, 31.2, 30.6, 29.3, 26.1, 20.4, 16.5, 16.3, 14.2, 9.6.

HRMS ESI: MF $[M+Na]^+$ $C_{44}H_{70}INO_{12}Na$ Calculated: 954.3835, Found: 954.3839.

Iodo-allyestrenol (2z)

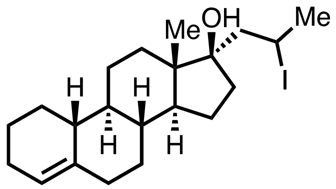

2y was prepared according to general procedure A using allyestrenol (30.2 mg, 0.100 mmol). Using the reaction from procedure A, preparative TLC yielded the product as colorless oil (9.5 mg, 22%, ~2.6:1 d.r.). The product was isolated as diastereomeric mixture, with the peaks for both diastereomers reported. Major and minor diastereomers could not be definitively assigned.

^{1}H NMR (500 MHz, $CDCl_3$) δ 5.37 (d, *J* = 15.4 Hz, 1H), 4.62–4.49 (m, 1H), 2.40–2.32 (m, 1H), 2.29–2.17 (m, 1H), 2.16–2.08 (m, 1H), 2.06 (d, *J* = 6.8 Hz, 3H), 2.01–1.89 (m, 4H), 1.84 (ddt, *J* = 11.5, 4.6, 3.3 Hz, 1H), 1.80–1.48 (m, 6H), 1.48–1.42 (m, 1H), 1.42–1.01 (m, 8H), 0.94–0.80 (m, 3H), 0.63 (qd, *J* = 10.6, 4.3 Hz, 1H).

^{13}C NMR (126 MHz, $CDCl_3$) δ 140.3, 120.2, 85.3, 50.2, 50.2, 49.7, 49.3, 47.3, 47.2, 45.9, 44.1, 42.1, 42.0, 37.6, 35.7, 35.5, 33.7, 33.6, 33.4, 31.9, 31.8, 31.1, 30.9, 30.5, 28.9, 28.9, 27.7, 26.7, 26.6, 26.0, 25.9, 25.8, 25.6, 25.6, 23.5, 22.2, 14.0, 13.9.

HRMS ESI: MF $[M+Na]^+$ $C_{21}H_{33}INaO$ Calculated: 451.1468, Found: 451.1459.

(1*R*)-((1*S*,2*S*,4*S*,5*R*)-5-(1-iodoethyl)quinuclidin-2-yl)(6-methoxyquinolin-4-yl)methanol (2z)

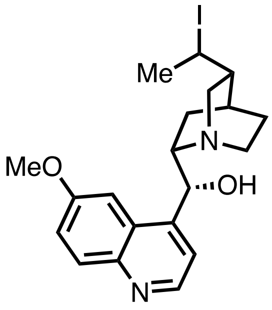

2z was prepared according to general procedure A using quinine (32.4 mg, 0.100 mmol). Using the reaction from procedure A, the crude material was purified by preparative HPLC, which was extracted with EtOAc to afford a colorless oil (19.5 mg, 45%, ~3:1 d.r.). The diastereomers were isolated and the analytical data with the peaks for both diastereomers reported. Relative stereochemistry for the major and minor diastereomers could not be definitively assigned.

Minor diastereomer:

^{1}H NMR (500 MHz, MeOD) δ 8.88 (d, *J* = 5.2 Hz, 1H), 8.11 (d, *J* = 9.3 Hz, 1H), 8.06 (dd, *J* = 5.2, 0.8 Hz, 1H), 7.67 (dd, *J* = 9.3, 2.6 Hz, 1H), 7.57 (d, *J* = 2.6 Hz, 1H), 6.15 (s, 1H), 4.27–4.13 (m, 2H), 4.07 (s, 3H), 3.84–3.74 (m, 1H), 3.65 (dd, *J* = 13.1, 10.2 Hz, 1H), 3.31 (p, *J* = 1.6 Hz, 3H), 3.10 (ddd, *J* = 13.1, 6.3, 2.8 Hz, 1H), 2.56 (q, *J* = 10.2 Hz, 1H), 2.43 (q, *J* = 3.1 Hz, 1H), 2.27 – 2.15 (m, 2H), 1.88 (d, *J* = 6.7 Hz, 3H), 1.50 (ddt, *J* = 13.8, 10.7, 3.1 Hz, 1H).

^{13}C NMR (126 MHz, MeOD) δ 161.4, 152.6, 144.9, 139.8, 128.4, 127.8, 126.5, 120.9, 102.7, 68.3, 60.9, 60.4, 57.1, 45.9, 45.1, 32.6, 27.2, 25.7, 25.3, 18.1.

HRMS ESI: MF $[M+H]^+$ $C_{20}H_{26}N_2O_2I$ Calculated: 453.1034, Found: 453.1031.

Major:

^{1}H NMR (500 MHz, MeOD) δ 8.88 (d, *J* = 5.1 Hz, 1H), 8.11 (d, *J* = 9.3 Hz, 1H), 8.06 (d, *J* = 5.1 Hz, 1H), 7.66 (dd, *J* = 9.3, 2.6 Hz, 1H), 7.54 (d, *J* = 2.6 Hz, 1H), 6.13–6.09 (m, 1H), 4.27–4.18 (m, 1H), 4.18–4.10 (m, 1H), 4.06 (s, 3H), 3.76 (dd, *J* = 10.9, 6.8 Hz, 1H), 3.69 (dd, *J* = 13.2, 10.5 Hz, 1H), 3.30–3.34 (m, 2H), 3.19 (ddd, *J* = 13.2, 6.2, 2.6 Hz, 1H), 2.60–2.55 (m, 1H), 2.36–2.20 (m, 3H), 2.00–1.92 (m, 1H), 1.89 (d, *J* = 6.6 Hz, 3H), 1.64–1.52 (m, 1H).

^{13}C NMR (126 MHz, MeOD) δ 161.3, 147.8, 145.4, 140.5, 128.3, 128.2, 126.0, 120.9, 102.7, 68.2, 60.9, 57.0, 54.3, 44.9, 44.4, 30.5, 29.7, 26.7, 25.4, 18.3.

HRMS ESI: MF $[M+H]^+$ $C_{20}H_{26}N_2O_2I$ Calculated: 453.1034, Found: 453.1029.

2-Methyl-1,4-diphenylazetidine (2ab')

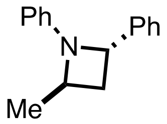

2-methyl-1,4-diphenylazetidine was prepared according to the general procedure using (±)-*N*-(1-phenylbut-3-en-1-yl)aniline (22.3 mg, 0.100 mmol). Using the reaction from procedure A, preparative TLC in 10% EtOAc:pentanes yielded the product as colorless oil (14.5 mg, 65%, >20:1 d.r.).

^{1}H NMR (400 MHz, $CDCl_3$) δ = 7.45–7.26 (m, 5H), 7.17–7.07 (m, 2H), 6.72–6.64 (m, 1H), 6.64–6.56 (m, 2H), 4.57 (dd, J = 8.6, 5.6 Hz, 1H), 3.83 (dqd, J = 9.6, 6.8, 4.5 Hz, 1H), 2.51 (ddd, J = 14.3, 9.8, 5.6 Hz, 1H), 2.02–1.88 (m, 4H).

^{13}C NMR (126 MHz, $CDCl_3$) δ = 146.9, 142.4, 129.3, 129.1, 127.8, 126.7, 117.8, 113.6, 58.5, 50.6, 29.2, 25.4.

HRMS ESI: MF $[M+H]^+$ $C_{16}H_{17}N$: Calculated: 224.1434, Found: 224.1436.

3-(Iodomethyl)-4-methyl-1-tosylpyrrolidine (2ac')

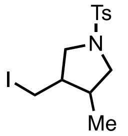

2ac' was prepared according to the general procedure using *N*,*N*-diallyl-4-methylbenzenesulfonamide (18.6 mg, 0.100 mmol). Using the reaction from procedure A, preparative TLC 10% EtOAc:pentanes yielded the product as colorless oil (18.6 mg, 49%, 2.75:1 d.r.). The product was isolated as diastereomeric mixture, with the peaks for both diastereomers reported. Major and minor are noted when chemical shifts are distinct and not overlapping with other signals.

^{1}H NMR (500 MHz, $CDCl_3$) δ = 7.72 (dd, J = 8.2, 1.5 Hz, 3H), 7.36–7.29 (m, 3H), 3.61–3.46 (m, 2H), 3.39 (dd, J = 9.8, 6.4 Hz, 1H), 3.19 (dd, J = 10.2, 3.9 Hz, 1H, minor), 3.13–3.06 (m, 2H), 3.02 (dt, J = 9.9, 7.3 Hz, 1H), 2.93 (dd, J = 10.1, 8.1 Hz, 1H, minor), 2.85 (ddd, J = 9.9, 8.6, 3.4 Hz, 2H), 2.51 – 2.38 (m, 1H), 2.44 (s, 5H), 2.35 – 2.24 (m, 1H), 1.81 (ddd, J = 12.0, 10.4, 5.7 Hz, 1H), 1.35 – 1.24 (m, 2H), 0.94 (d, J = 6.2 Hz, 3H, minor), 0.79 (d, J = 7.1 Hz, 3H, major).

^{13}C NMR (126 MHz, $CDCl_3$) δ = 143.7, 143.7, 133.9, 133.7, 129.9, 127.7, 127.6, 55.2, 54.6, 54.5, 51.9, 47.8, 45.4, 39.5, 36.3, 21.7, 16.4, 12.6, 6.3, 2.8.

HRMS ESI: $[M+H]^+$ MF $C_{13}H_{18}NO_2SI$ Calculated: 379.0097, Found: 379. 0096.

2,7-Diiodo-2,6-dimethyloctane (2ad')

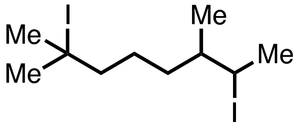

To a solution of (±)-β-citronellene (**1ad**) (0.91 mL, 5.0 mmol) in acetic acid (25 mL) were added LiI (3.3 g, 25 mmol, 5.0 equiv) then MsOH (1.7 mL, 25 mmol, 5.0 equiv). The mixture was stirred at room temperature for 4 h, then extracted with hexanes (3 x 25 mL). The combined organic layers were washed with sat. aq. $Na_2S_2O_3$, dried with $MgSO_4$, filtered, and concentrated under

reduced pressure to obtain an orange oil (crude mass 1.9 g, 4.8 mmol, 97%), which was filtered through silica with pentanes and the crude material was used without further purification. The product decomposes within 24 h, even at –40 °C, so the material was used in subsequent steps immediately.

^{1}H NMR (400 MHz, $CDCl_3$, mixture of diastereomers) *δ* 4.43–4.30 (m, 1H), 1.96–0.79 (m, 19H)

HRMS ESI: MF $C_{10}H_{20}I$ $[M-I]^+$ calculated: 267.0604, found: 267.0602.

7-Iodo-2,6-dimethyloct-2-ene (2ad)

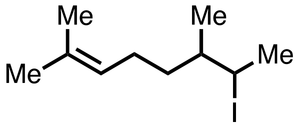

2ad was formed according to a modified general procedure B (80 °C, 16 h) using **2ad'** (39.4 mg, 0.100 mmol) and norbornadiene (10 μL, 0.100 mmol). The product was detected by crude ^{1}H NMR in 57% yield, in 8:1 r.r. with the minor regioisomer being the 1,1-disubstituted alkene from the elimination. **2ad** was prepared according to general procedure B using **1ad** (18 μL, 0.100 mmol). Using the reaction from **1ad**, flash silica column chromatography 100% hexanes yielded the product as a colorless oil (6.1mg, 23% yield, 1:1 d.r.). The product was isolated as a diastereomeric mixture, with the peaks for both diastereomers reported.

^{1}H NMR (500 MHz, $CDCl_3$) δ 5.12–5.06 (m, 1H), 4.36 (dqd, *J* = 13.2, 7.0, 3.3 Hz, 1H), 2.14–1.93 (m, 1H), 1.87 (dd, *J* = 25.9, 7.0 Hz, 3H), 1.69 (s, 3H), 1.61 (s, 3H), 1.39–1.08 (m, 2H), 0.99 (dd, *J* = 19.3, 6.6 Hz, 2H), 0.91–0.79 (m, 3H).

^{13}C NMR (126 MHz, $CDCl_3$) δ 132.0, 132.0, 124.3, 124.2, 41.6, 41.4, 40.5, 39.9, 37.6, 34.6, 26.8, 25.9, 25.8, 25.4, 24.9, 22.8, 18.4, 17.9, 17.8, 17.3.

HRMS EI: MF $C_{10}H_{19}I$ [M] Calculated: 266.0526, Found: 266.0527.

Scale-up procedure (General Procedure C): Using a modified procedure from General Procedure B, **1a** (1.0 equivalent), *tert*-butyl iodide (3.0 equivalents), 1,4-dioxane [1 M] and **Co-1** (0.05 equivalents) were added under air to an appropriately sized Schlenk tube (>1/2 the volume of the reaction). The tube was closed and freeze-pumped-thawed under N_2 three times. Then, through a septum, *t*-BuOOH (0.1 equivalent) was added resulting in a brown solution followed ~30 seconds later by $PhSiH_3$ (0.1 equivalent). The reaction was heated to 55 °C under a continuous stream of N_2 for 25 minutes then cooled to room temperature. The solvent was removed in vaccuo and the reaction diluted with pentanes and a saturated solution of sodium thiosulfate. The aqueous phase was extracted with pentanes (3x), dried over $MgSO_4$ then solvent evaporated. The crude oil was purified by flash silica column chromatography using 100% pentanes. The products were afforded in the following amounts according to scale. The analytical data matches that of the smaller scale reaction of **2a** reported above. [1 mmol; 85% yield, 221 mg. 10 mmol; 83% yield, 2.161 g. 100 mmol 62%, 16.179 g].

We attribute the decreased yield for the 100 mmol reaction due to the prolonged time required for the freeze-pump-thaw sequence which resulted in considerable decomposition of the *tert*-butyl iodide. This could be visually monitored by the solution going from colorless to a dark brown color before the end of the third cycle.

1-(3-Bromobutoxy)-4-iodobenzene (3a)

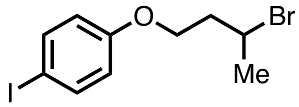

3a was prepared according to following procedure. Inside an argon-filled glovebox, to a 2-dram vial equipped with a magnetic stirring bar, **Co-1** (3.0 mg, 5.0 µmol), $Mn_2(CO)_{10}$ (4.7 mg, 12 µmol), and the **1w** (27.4 mg, 0.100 mmol) were dissolved in 1,4-dioxane (0.3 mL). *Tert*-butyl bromide (79 µL, 0.700 mmol) was added and the vial was sealed and removed from the glovebox. The reaction mixture was placed in a photochemical reactor and irradiated with a 450

nm LED lamp for 2 h at 25 °C. The solution was filtered through silica and washed with pentanes (4 mL) and Et_2O (4 mL). The solvent was removed in vacuo and the material was purified by flash silica column chromatography with 20% DCM:pentanes to afford the product as a colorless oil (19.1 mg, 54%).

^{1}H NMR (400 MHz, $CDCl_3$) δ 7.71–7.46 (m, 2H), 6.78–6.59 (m, 2H), 4.37 (dqd, *J* = 9.2, 6.7, 4.3 Hz, 1H), 4.13–4.01 (m, 2H), 2.35–2.11 (m, 2H), 1.79 (d, *J* = 6.7 Hz, 3H).

^{13}C NMR (101 MHz, $CDCl_3$) δ 158.7, 138.4, 117.1, 83.1, 66.1, 47.5, 40.5, 26.8.

HRMS ESI: $C_{10}H_{12}IOBr$ $[M+H]^+$ Calculated: 353.9111, Found: 353.9105.

2-((4-(4-Iodophenoxy)butan-2-yl)thio)pyridine (3b)

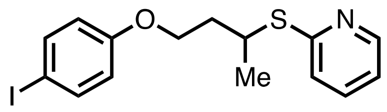

3b was prepared according to a modified general procedure A. Inside an argon-filled glovebox, to a 2-dram vial equipped with a magnetic stirring bar, **Co-1** (3.0 mg, 5.0 µmol), **Cr-1** (6.3 mg, 15 µmol), and the **1w** (27.4 mg, 0.100 mmol) were dissolved in 1,4-dioxane (1 mL). *Tert*-butyl Barton ester[8] (42.3 mg, 0.200 mmol) was added and the vial was sealed and removed from the glovebox. The reaction was heated for 2 h at 55 °C. The solution was filtered through silica and washed with pentanes (4 mL) and ethyl acetate (4 mL). The solvent was removed in vacuo and the material was purified by flash silica column chromatography with 15% EtOAc:pentanes to afford the product as a colorless oil (18.2 mg, 47%).

^{1}H NMR (400 MHz, $CDCl_3$) δ 8.38 (ddd, *J* = 5.0, 1.9, 0.9 Hz, 1H), 7.60–7.49 (m, 2H), 7.45 (ddd, *J* = 8.0, 7.4, 1.9 Hz, 1H), 7.16 (dt, *J* = 8.0, 1.0 Hz, 1H), 6.95 (ddd, *J* = 7.4, 4.9, 1.0 Hz, 1H), 6.77–6.47 (m, 2H), 4.22–3.91 (m, 3H), 2.36–2.04 (m, 2H), 1.48 (d, *J* = 6.9 Hz, 3H).

^{13}C NMR (101 MHz, $CDCl_3$) δ 159.0, 158.9, 149.6, 138.3, 136.1, 123.0, 119.6, 117.1, 82.7, 65.9, 37.0, 36.3, 21.8.

HRMS ESI: MF $C_{15}H_{17}IONS$ $[M+H]^+$ Calculated: 386.0070, Found: 386.0062.

5 Product derivatization

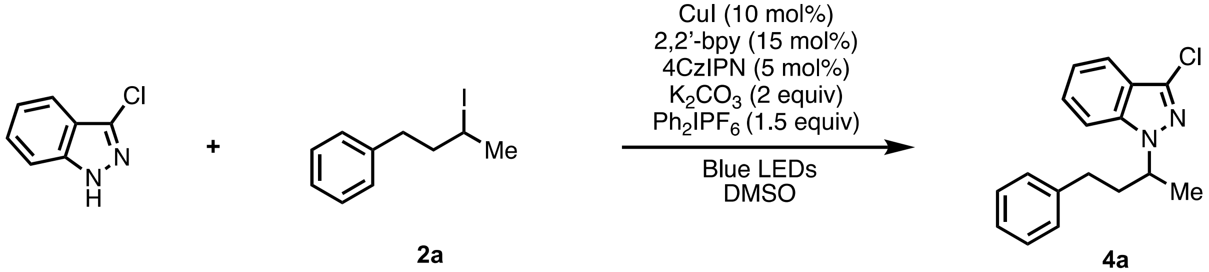

2a **4a**

3-Chloro-1-(4-phenylbutan-2-yl)-1H-indazole (4a): An oven-dry tube equipped with a stirring bar was charged with 4CzIPN (1.6 mg, 2 μmol, 2 mol%), CuI (1.9 mg, 0.01 mmol, 10 mol%), 2,2'-bipyridine (2.3 mg, 0.015 mmol, 15 mol%), K_2CO_3 (28 mg, 0.20 mmol, 2.0 equiv.), Ph_2IPF_6 (64 mg, 0.15 mmol, 1.5 equiv.), **2a** (26 mg, 0.10 mmol, 1.0 equiv) and 3-chloro-1*H*-indazole (23 mg, 0.15 mmol, 1.5 equiv). Dry and degassed DMSO (1.0 mL, 0.1 M) was added. The vial was sonicated for 2 mins, then placed approximately 8 cm from blue LEDs. The blue LEDs were switched on and the mixture was stirred under continuous irradiation at room temperature for 2 hours under fan cooling. The LEDs were switched off, the tube was opened, and the mixture was diluted with NH_4Cl sat. (2 mL) and EtOAc (5 mL). The layers were separated, and the aqueous layer was extracted with EtOAc (2 × 5 mL). The combined organic layers were washed with brine (2 mL), dried (Na_2SO_4), filtered and evaporated under reduced pressure. The crude was purified by flash chromatographic purification (10% EtOAc in hexanes to obtain the product as a colorless oil (23.0 mg, 81% yield).

^{1}H NMR (400 MHz, $CDCl_3$) δ 7.68 (dt, *J* = 8.2, 1.0 Hz, 1H), 7.41–7.36 (m, 1H), 7.27–7.17 (m, 5H), 7.08–7.03 (m, 2H), 4.55 (dqd, *J* = 8.9, 6.6, 4.7 Hz, 1H), 2.58–2.41 (m, 3H), 2.19–2.10 (m, 1H), 1.54 (d, *J* = 6.7 Hz, 3H).

^{13}C NMR (101 MHz, $CDCl_3$) δ 141.2, 140.8, 132.9, 128.6, 128.5, 127.3, 126.1, 121.2, 121.1, 120.0, 109.5, 54.3, 37.7, 32.6, 21.1.

HRMS ESI: MF $C_{17}H_{18}ClN_2$ $[M+H]^+$ 285.1153, found 285.1150.

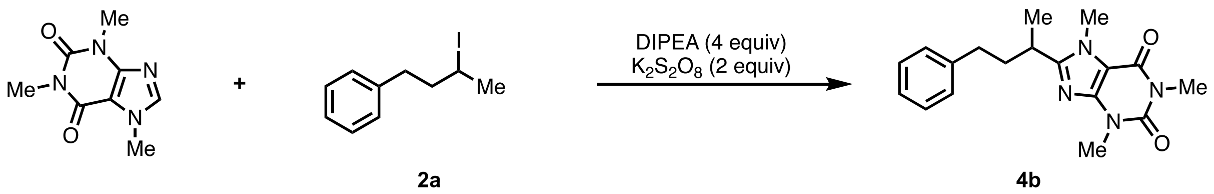

1,3,7-Trimethyl-8-(4-phenylbutan-2-yl)-3,7-dihydro-1H-purine-2,6-dione (4b): A tube equipped with a stirring bar was charged with caffeine (19 mg, 0.1 mmol, 1.0 equiv.), the **2a** (52 mg, 0.200 mmol, 2.0 equiv.), and potassium persulfate (54 mg, 0.2 mmol, 2.0 equiv.). DMSO (0.75 mL), water (0.25 mL), and DIPEA (70 µL, 0.4 mmol, 4.0 equiv.) were then added. The mixture was immediately transferred to a preheated aluminum block at 70 °C and stirred at this temperature for 2 h. The mixture was allowed to cool to room temperature and was diluted with H_2O (15 mL) and EtOAc (15 mL). The layers were separated and the aqueous layer was extracted with EtOAc (x 2). The combined organic layers are washed with brine (x 2), dried ($MgSO_4$), filtered and evaporated. The crude was purified by flash column chromatography on silica gel (15-30% acetone in pentanes) and obtained as a colorless oil (12.3 mg, 38% yield).

^{1}H NMR (400 MHz, CDCl3) δ 7.30–7.24 (m, 2H), 7.21–7.16 (m, 1H), 7.12–7.08 (m, 2H), 3.77 (s, 3H), 3.58 (s, 3H), 3.40 (s, 3H), 2.92–2.81 (m, 1H), 2.67–2.53 (m, 2H), 2.23 (dtd, J = 13.6, 8.1, 6.5 Hz, 1H), 1.99 (m, 1H), 1.32 (d, J = 6.9 Hz, 3H).

^{13}C NMR (101 MHz, CDCl3) δ 158.0, 155.6, 151.9, 148.4, 141.3, 128.6, 128.4, 126.2, 107.1, 36.8, 33.5, 31.4, 30.5, 29.9, 28.0, 19.5.

HRMS ESI: MF $C_{18}H_{22}N_4NaO_2$ $[M+Na]^+$ Calculated: 349.1635; Found: 349.1630.

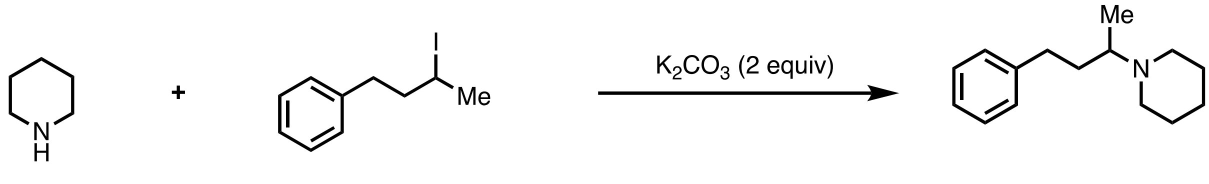

2a **4c**

1-(4-phenylbutan-2-yl)piperidine (4c): The title compound was prepared according to the following procedure. **2a** (130 mg, 0.5 mmol), piperdine (59μL, 0.6 mmol), and K_2CO_3 (83 mg, 0.6 mmol) were dissolved in 2 mL MeCN. The reaction was heated to 80 °C for 16 h. The mixture was allowed to cool to room temperature and was diluted with H_2O (15 mL) and EtOAc (15 mL). The layers were separated and the aqueous layer was extracted with EtOAc (x 2). The combined organic layers are washed with brine (x 2), dried ($MgSO_4$), filtered and evaporated. The crude was purified by flash column chromatography on silica gel (50% acetone in pentanes) and obtained as a colorless oil (54.6 mg, 50% yield).

The conditions above could be replicated with the addition of **1a** (50μL, 0.33 mmol) to give a yield of 48% (^{1}H NMR).

^{1}H NMR (500 MHz, $CDCl_3$) δ 7.32–7.27 (m, 2H), 7.25–7.15 (m, 3H), 2.79–2.59 (m, 2H), 2.59–2.47 (m, 3H), 2.43 (d, *J* = 6.5 Hz, 2H), 1.94–1.81 (m, 1H), 1.68–1.51 (m, 5H), 1.45 (p, *J* = 5.9 Hz, 2H), 1.02 (d, *J* = 6.5 Hz, 3H).

^{13}C NMR (126 MHz, $CDCl_3$) δ 142.9, 128.5, 128.2, 125.6, 58.9, 49.3, 35.5, 33.3, 26.5, 25.0, 13.8.

HRMS ESI: MF $C_{15}H_{24}N$ $[M+H]^+$ Calculated: 218.1903, Found: 218.1904.

6 Preparation of catalysts

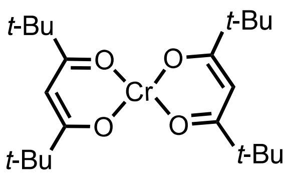

Cr-1

Cr(2,2,6,6-tetramethyl-5-oxohept-3-en-3-olate)$_2$ (**Cr-1**): To a stirred solution of 2,2,6,6-tetramethyl heptanedione (3.77 mL, 3.32 g, 18.0 mmol, 2 equiv.) in THF (10 mL) was added *n*-BuLi (11.3 mL, 1.6 M, 18.0 mmol, 2 equiv.) at -78 °C over a period of 30 min. After stirring for 2 h and warming up to room temperature, the reaction mixture was transferred to a glove box and added to a solution of $CrCl_2$ (1.11 g, 9.00 mmol, 1 equiv.) in THF (60 mL). The mixture was stirred at room temperature overnight. The solvent was evaporated *in vacuo*, then the resulting solids were extracted with hexanes (4 x 5 mL), and the resulting hexane solution was stored at –40 °C overnight, yielding the desired paramagnetic complex **Cr-1** as yellow-brownish crystalline solid (1.80 g, 48%). Analysis by X-ray revealed the desired structure matched known structure.[9]

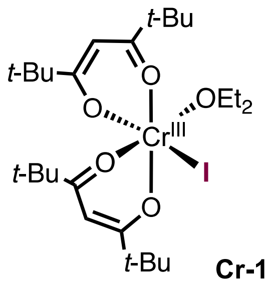

Cr-1'

Cr(2,2,6,6-tetramethyl-5-oxohept-3-en-3-olate)$_2$(I)(OEt_2) (**Cr-1'**): To a stirred solution of **Cr-1** (100 mg, 0.238 mmol) in Et_2O (5 mL) was added I_2 (30 mg, 0.118 mmol) and stirred at room temperature overnight. The solvent was allowed to slowly evaporate inside the glovebox by sitting open overnight. This afforded **Cr-1'** as a green solid with single crystals suitable for X-ray analysis directly from this crop (145 mg, 98%). This solid is extremely sensitive to air outside the

glovebox, and decomposes within seconds. Therefore, the sample had to be mounted inside the glovebox for X-ray analysis.

X-ray CCDC: 2306575

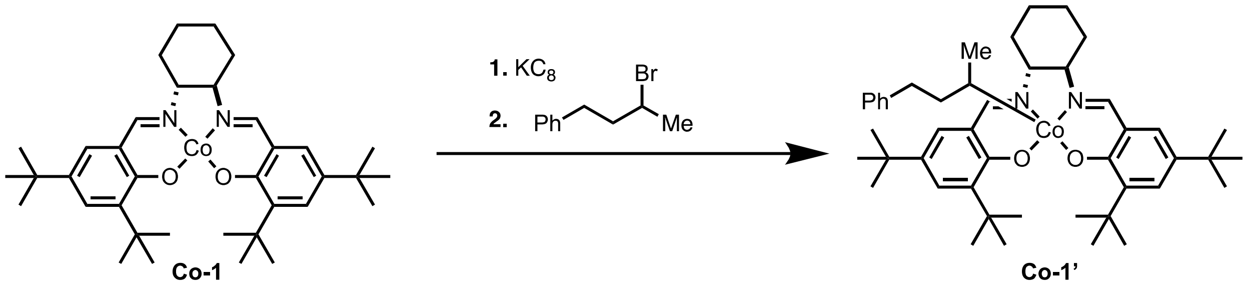

In an Ar filled glovebox, **Co-1** (90 mg, 0.15 mmol) was dissolved in THF (3 mL) and cooled in the freezer at –40 °C for 2 h. To this red solution, KC_8 (20 mg, 0.15 mmol) was added with an additional amount of THF (1 mL). While stirring, the reaction was allowed to warm up to rt for 1 h. The reaction was filtered through a Celite pad (2 cm) packed in a pipette. The filtered green solution was placed in the glovebox freezer for 2 h, then alkyl bromide (35 mg, 0.165 mmol) in pre-cooled THF (0.3 mL) was added. The reaction was stirred and allowed to warm up to room temperature for 3 h. The reaction was dried under reduced pressure to give a brown solid. The product was washed with pentane (2 x 5 mL), then extracted with Et_2O. The extract was filtered and dried under vacuum to yield **Co-1'** as a brown solid (69% yield, 76 mg, 1:1 d.r.). The analytical data matches the literature values.[10]

^{1}H NMR (400 MHz, THF) δ 8.15–7.81 (m, 2H), 7.41 (ddd, *J* = 14.6, 5.3, 2.3 Hz, 1H), 7.31–7.23 (m, 2H), 7.22–7.12 (m, 2H), 7.10–6.93 (m, 3H), 6.73–6.62 (m, 1H), 5.22–5.06 (m, 1H), 4.13 (q, *J* = 6.6 Hz, 1H), 3.43 (q, *J* = 7.0 Hz, 1H), 3.32 (d, *J* = 6.3 Hz, 1H), 3.03 (d, *J* = 60.1 Hz, 1H), 2.91–2.58 (m, 4H), 2.13–1.87 (m, 4H), 1.68 (d, *J* = 3.1 Hz, 9H), 1.64 (d, *J* = 4.6 Hz, 9H), 1.38–1.30 (m, 18H), 0.94 (t, *J* = 6.5 Hz, 2H), 0.18 (s, 9H), -0.36 (d, *J* = 6.8 Hz, 3H, diastereomer 1), -0.46 (d, *J* = 8.8 Hz, 3H, diastereomer 2).

7 Mechanistic considerations

Pathway 1: Alkyl–Co^{IV} are electrophilic at carbon and can undergo substitution by nucleophiles.[10, 11] The oxidation of alkyl–Co^{III} to alkyl–Co^{IV} could potentially occur by I_2 or **I•** which could be formed by decomposition of *tert*-butyl iodide. Then, I^- could attack the electrophilic carbon to form the product and reform the starting catalyst **Co-1**. There is no evidence for this pathway and internal or external nucleophiles were never found to attack the alkyl-Co^{IV} which would likely occur if this were the pathway. Also, this pathway is unlikely based on the required microscopic reversibility of the C–I bond forming step which would imply that **Co-1** can undergo direct S_N2-type oxidative addition to alkyl iodides which does not occur.

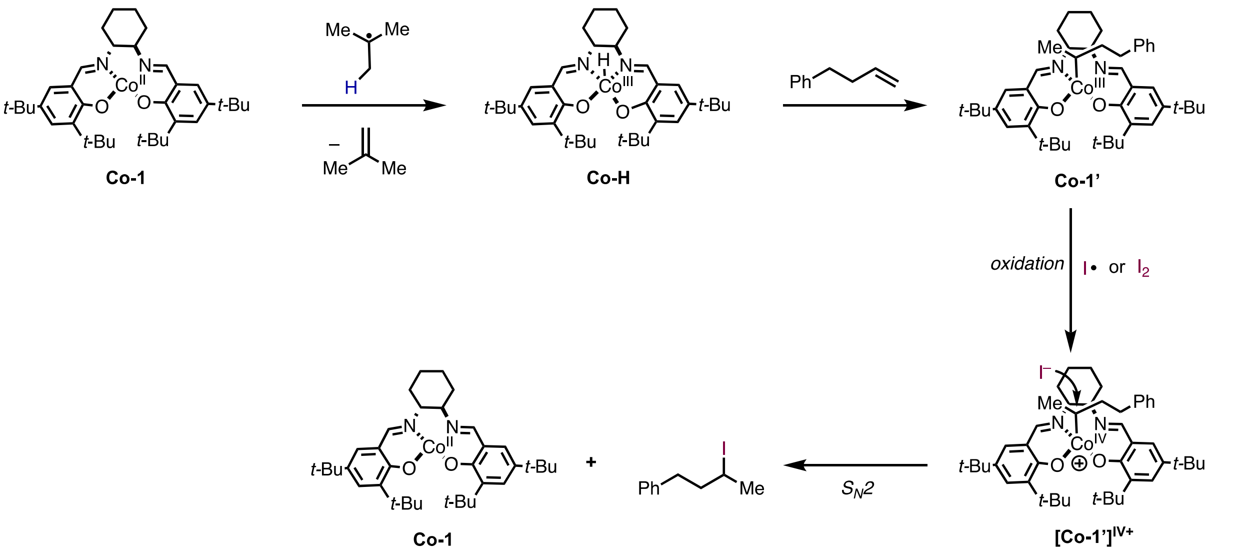

Supplementary Figure 1: **Alternative oxidative MHAT mechanistic pathway. Co-1** abstracts a hydrogen atom from a *tert*-butyl radical to form **Co-H**. **Co-H** undergoes HAT with **1a** to form **Co-1'**. This is oxidized by in situ generated I_2 or **I•** and followed by rebound attack of the I^- on the electrophilic carbon to form the product and regenerate the starting complex.

Pathway 2 (Only possible for condition A): **Co-1** undergoes single electron transfer with **Cr-1**[12] to form a nucleophilic **[Co-1]⁻** which can attack the alkyl halide to form an alkyl Co^{III} species[13]. β–H elimination or Co–C homolysis and HAA would afford **Co-H** which could undergo MHAT and trapping of the alkyl radical with *tert*-butyl iodide or **Cr–1**'. This pathway can be ruled out as more highly substituted alkyl iodides react faster than less substituted which would be the opposite trend if there were nucleophilic attack on the alkyl iodide.

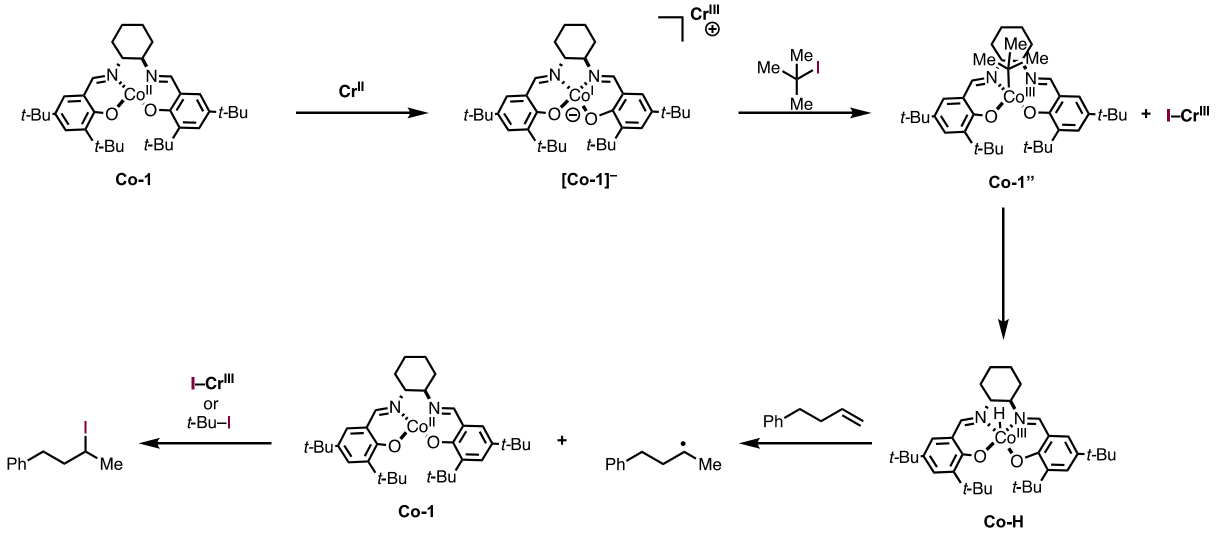

Supplementary Figure 2: **Cr to Co electron transfer pathway. Co-1** and **Cr-1** undergo SET to form Co^{I} and Cr^{III}. The $[Co\text{-}1]^{-}$ is nucleophilic and attacks *tert*-butyl iodide to form an alkyl-Co^{III} and **Cr-1'** . β–H elimination or Co–C homolysis and HAA would afford **Co-H** which could undergo MHAT and trapping of the alkyl radical with *tert*-butyl iodide or **Cr–1.** This would regenerate **Co-1** but in order to fully turnover both cycles **Cr-1'** must undergo XAT to reform **Cr-1**.

Pathway 3: The reaction is initiated by XAA with **Cr-1** and *tert*-butyl iodide to form a *tert*-butyl radical and **Cr-1'**. **Co-1** undergoes HAA from the *tert*-butyl radical to form **Co-H** which undergoes MHAT to the alkene and radical cage escape of the alkyl radical. The product can then be formed by either XAT from **Cr-1'** or *tert*-butyl iodide to produce **Cr-1** or a *tert*-butyl radical.

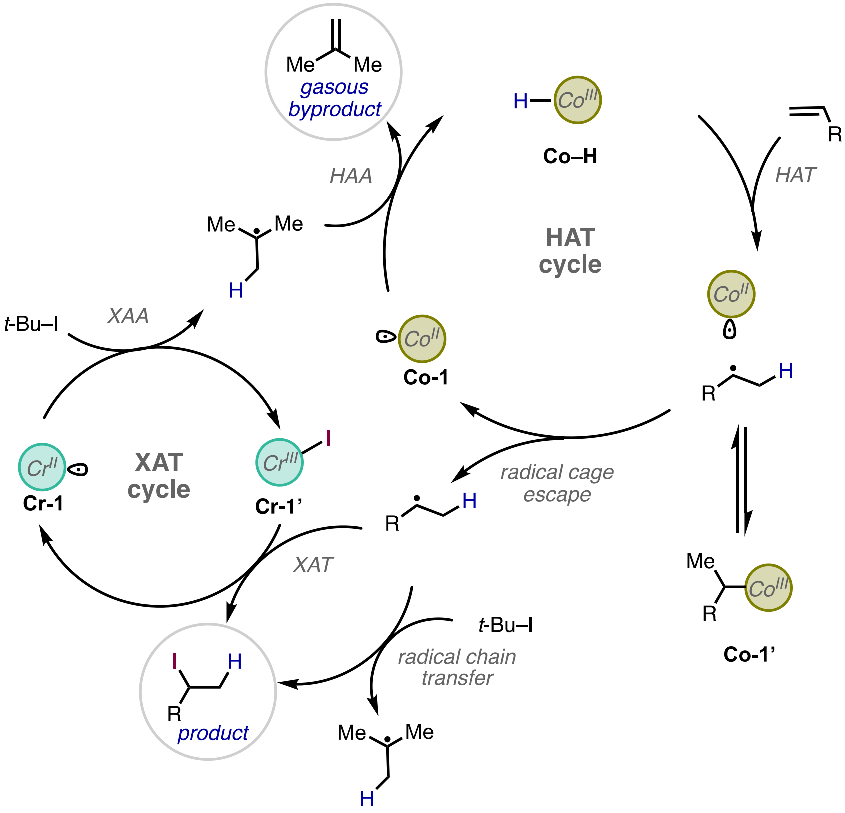

Supplementary Figure 3: **Proposed catalytic cycle. Co-1** and **Cr-1** undergo SET to form Co^{I} and Cr^{III}. The $[Co\text{-}1]^{-}$ is nucleophilic and attacks *tert*-butyl iodide to form an alkyl-Co^{III} and **Cr-1'** . β–H elimination or Co–C homolysis and HAA would afford **Co-H** which could undergo MHAT and trapping of the alkyl radical with *tert*-butyl iodide or **Cr–1.** This would regenerate **Co-1** but in order to fully turnover both cycles **Cr-1'** must undergo XAT to reform **Cr-1**.

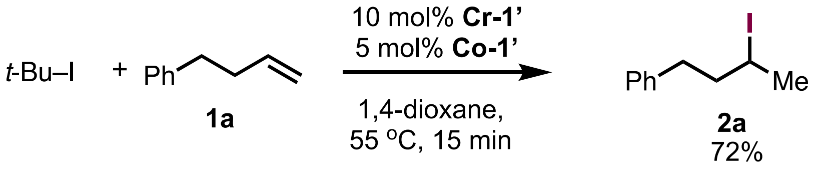

Supplementary Figure 4: **Catalytic competency of model complexes.** Control reaction showing the catalytic competency of **Co-1'** and **Cr-1'**.

8 NMR spectra

1d ^{1}H NMR (400 MHz, $CDCl_3$)

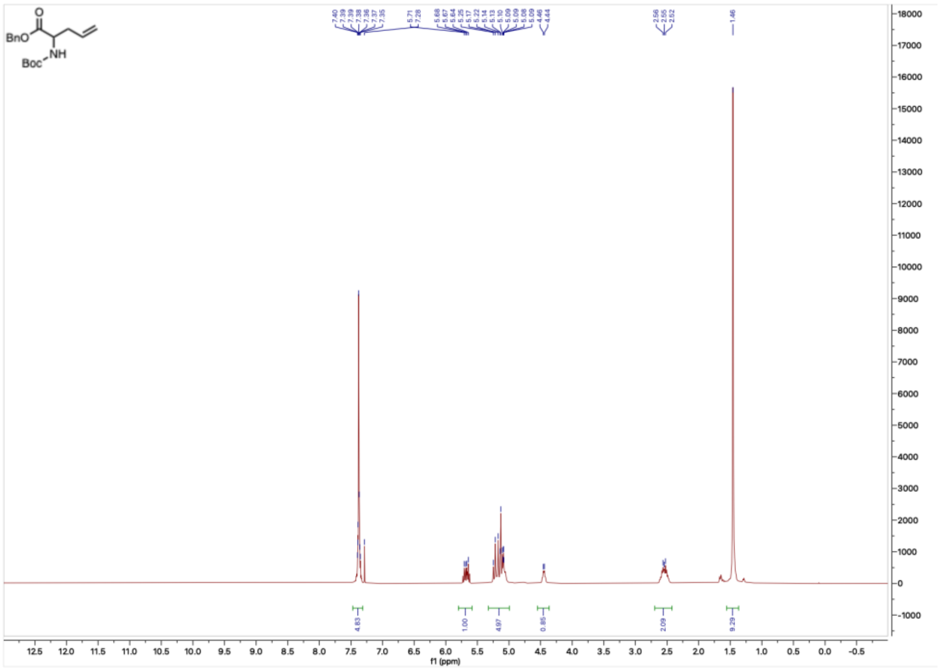

1d ^{13}C NMR (101 MHz, $CDCl_3$)

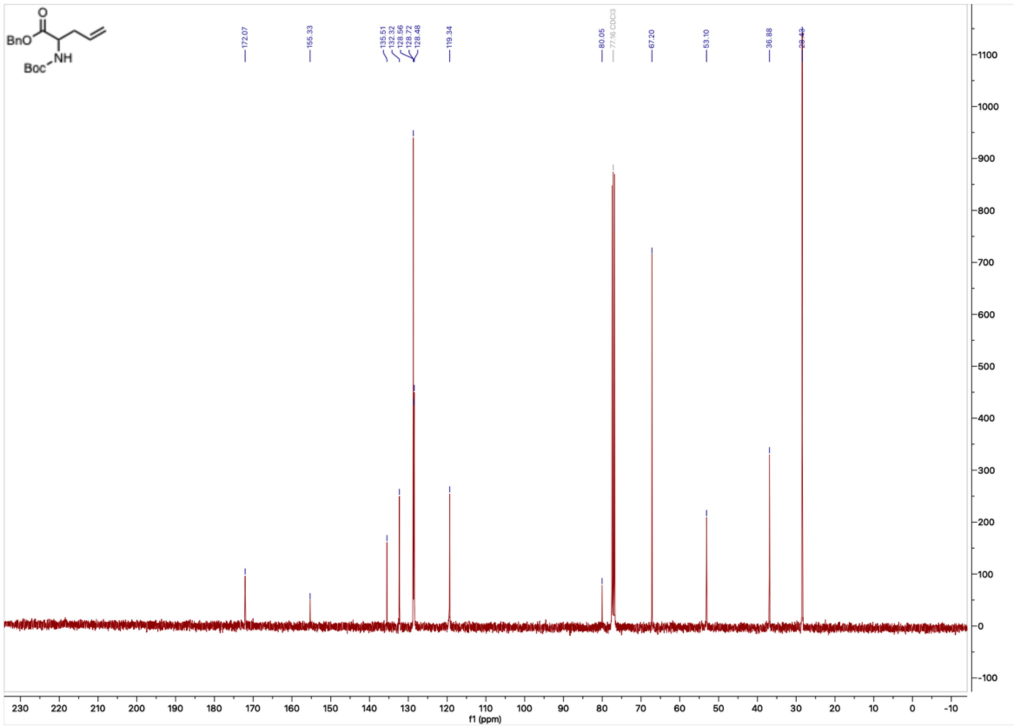

1e ^{1}H NMR (400 MHz, $CDCl_3$)

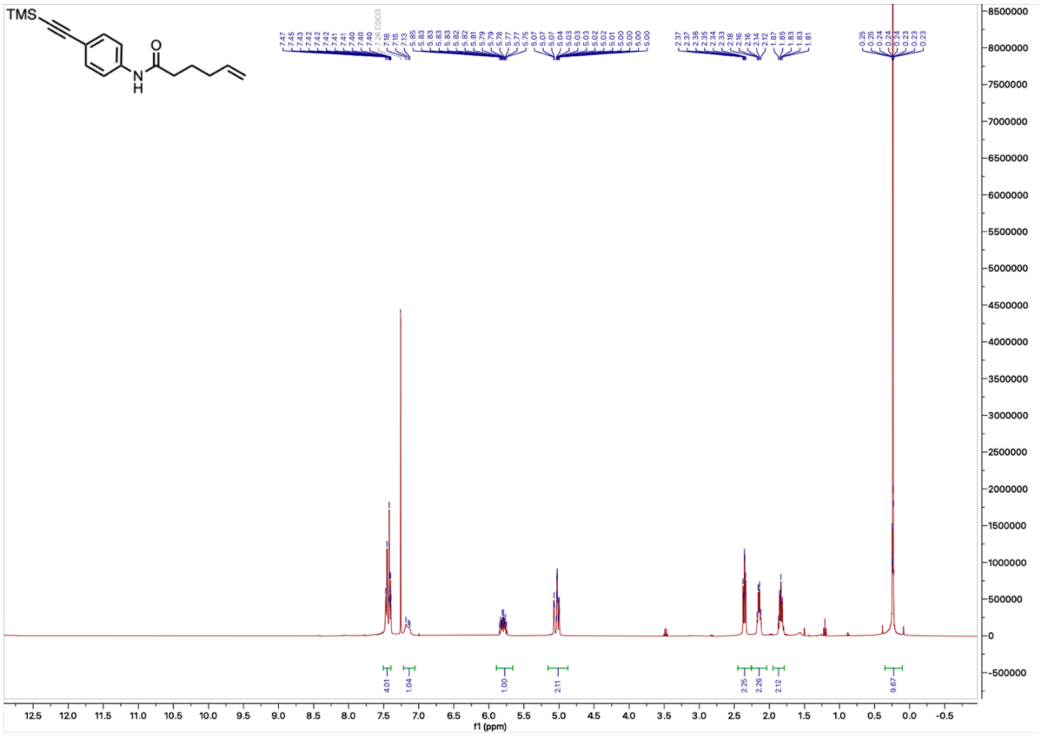

1e ^{13}C NMR (101 MHz, $CDCl_3$)

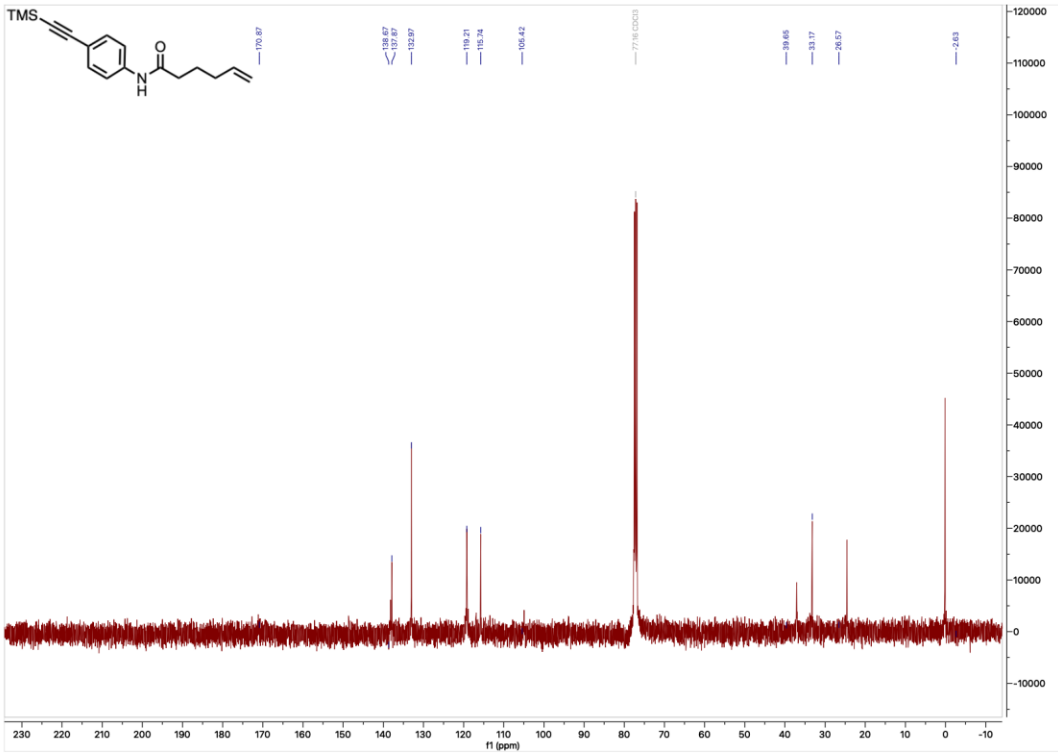

1l ^{1}H NMR (400 MHz, $CDCl_3$)

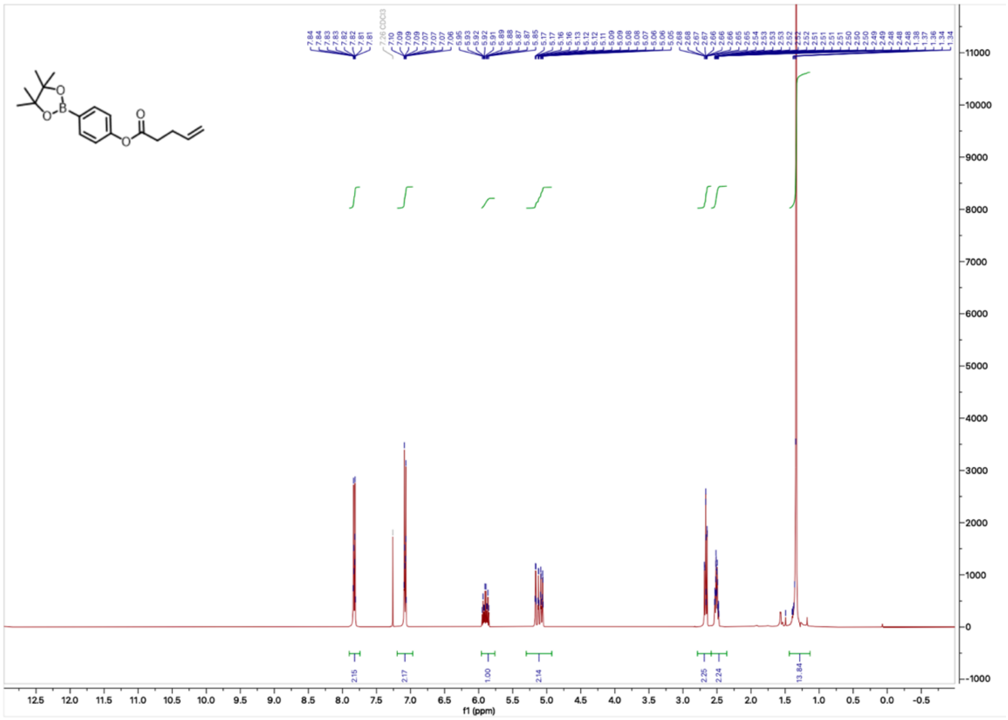

1l ^{13}C NMR (101 MHz, $CDCl_3$)

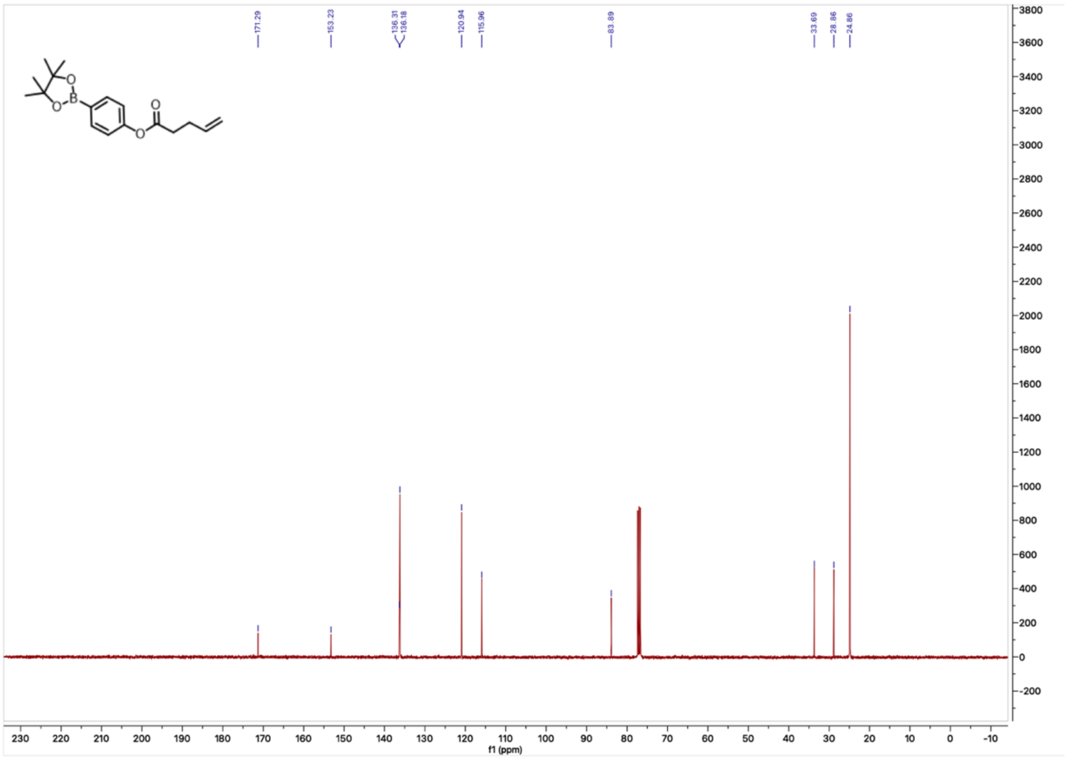

1k ^{1}H NMR (400 MHz, $CDCl_3$)

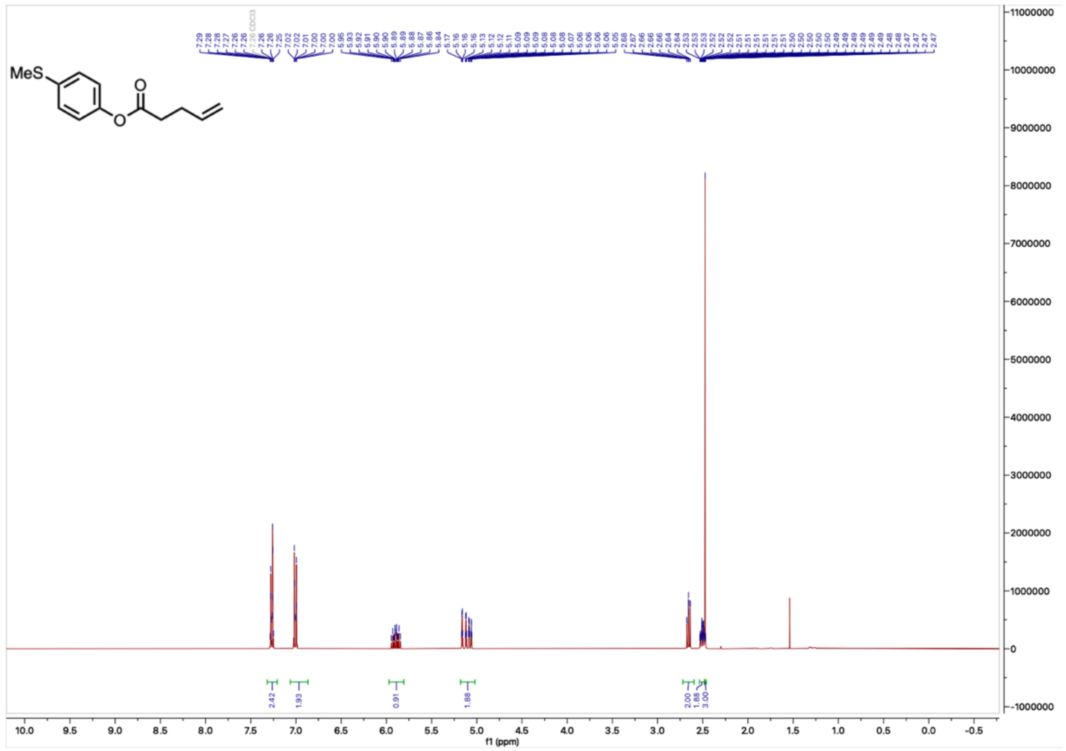

1k ^{13}C NMR (101 MHz, $CDCl_3$)

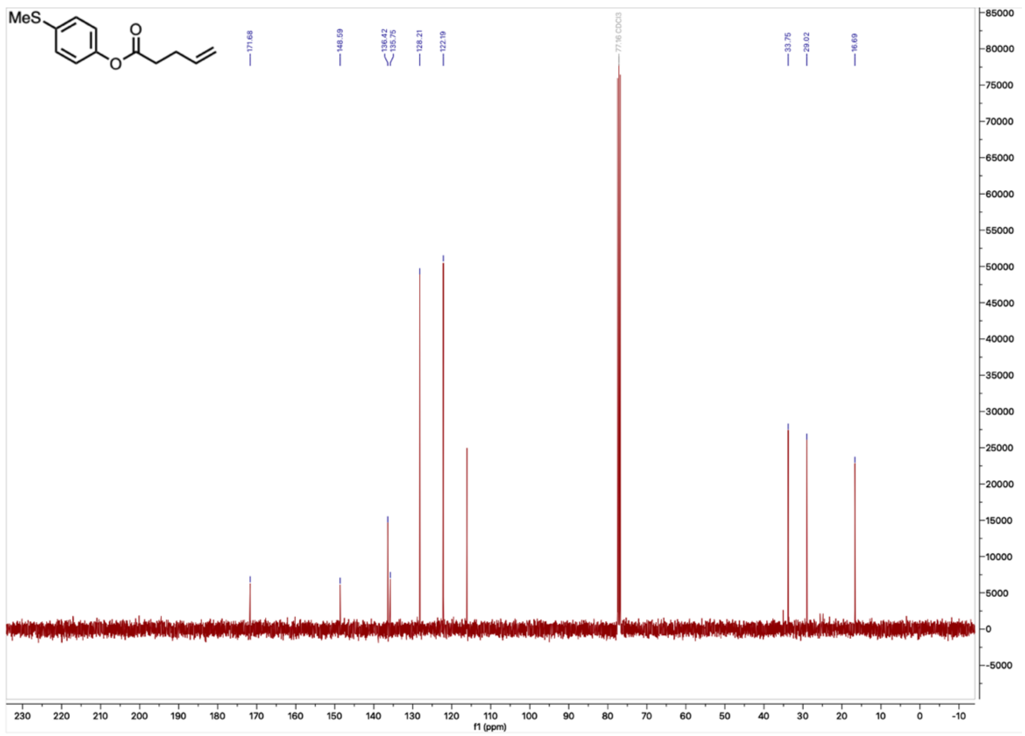

1m ^{1}H NMR (400 MHz, $CDCl_3$)

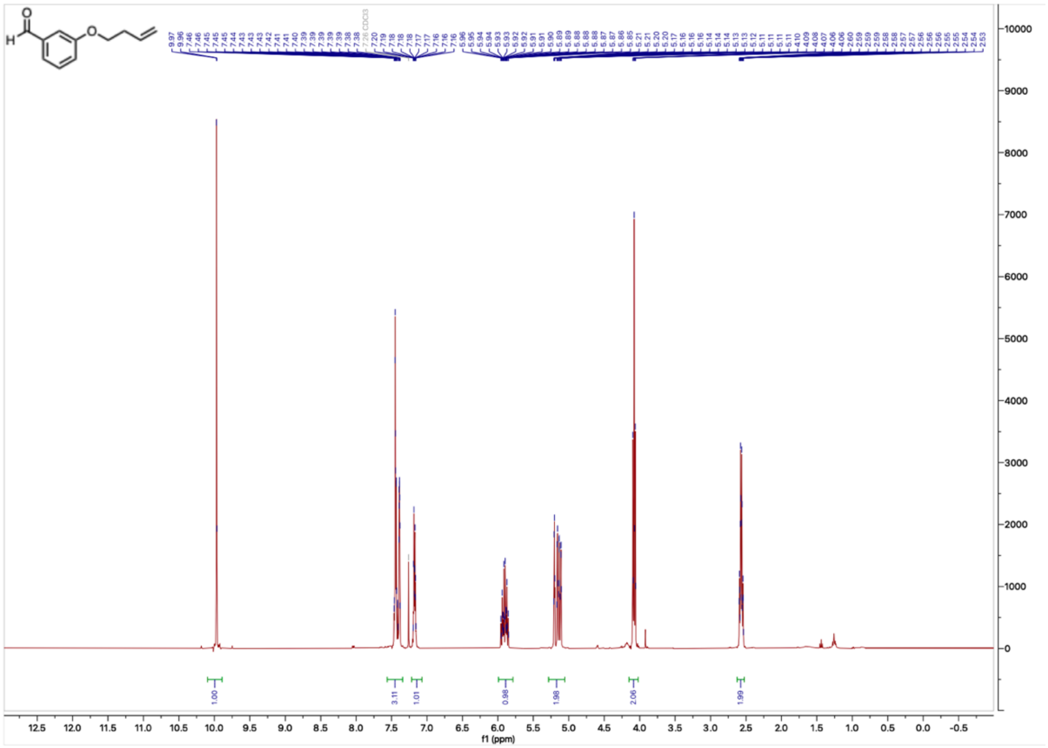

1m ^{13}C NMR (101 MHz, $CDCl_3$)

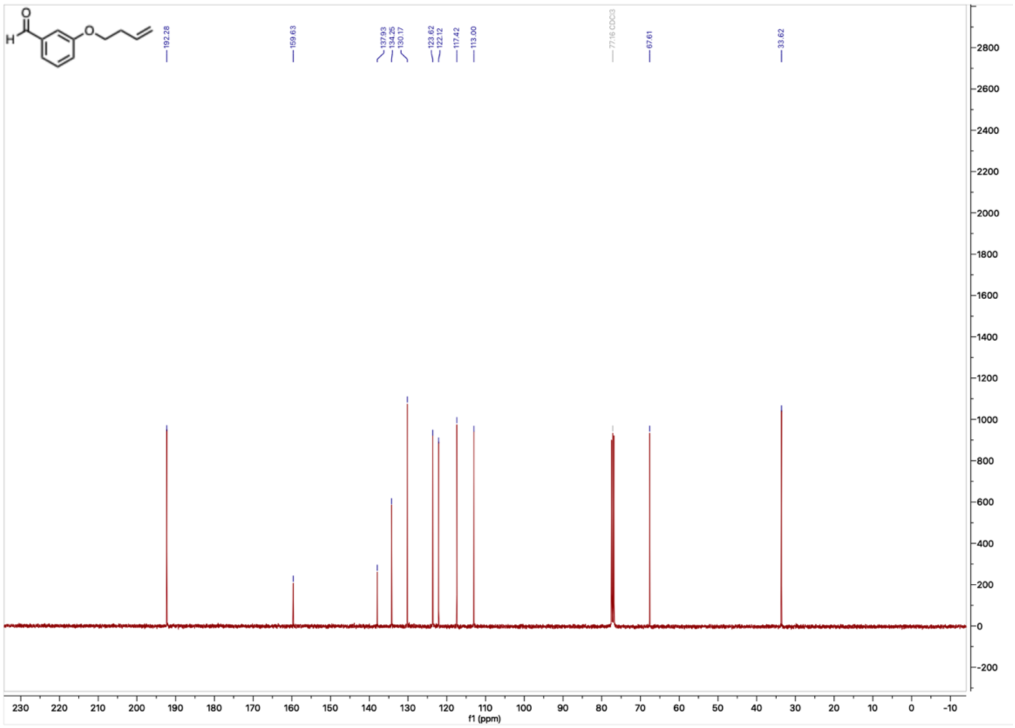

1t ^{1}H NMR (400 MHz, $CDCl_3$)

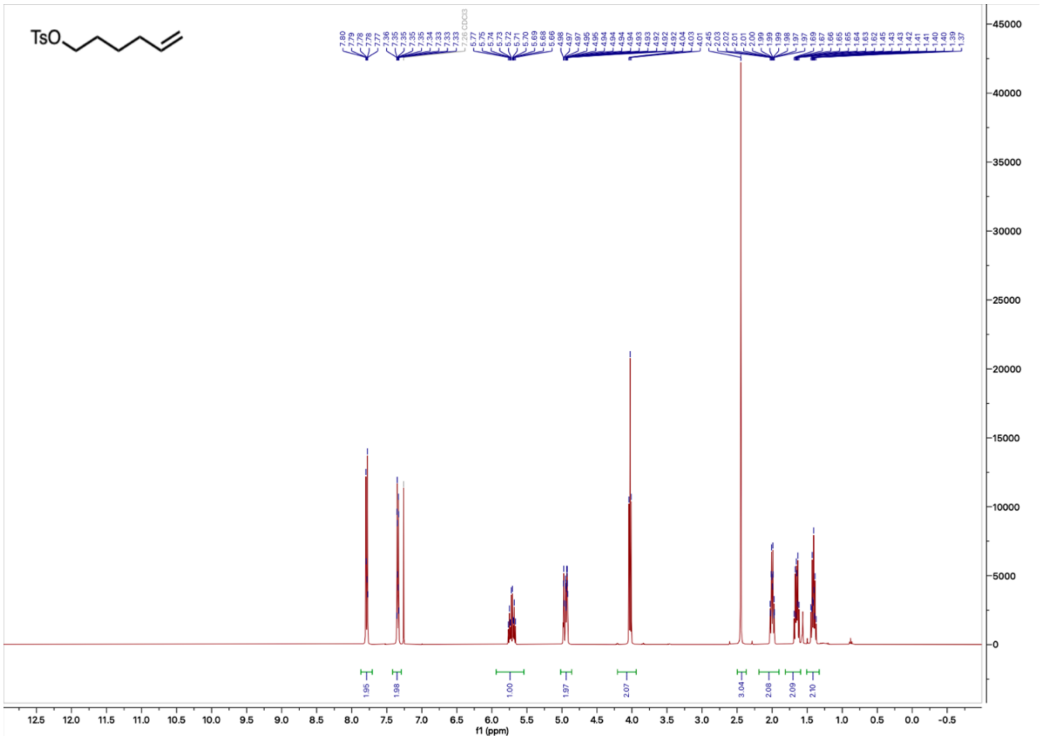

1t ^{13}C NMR (101 MHz, $CDCl_3$)

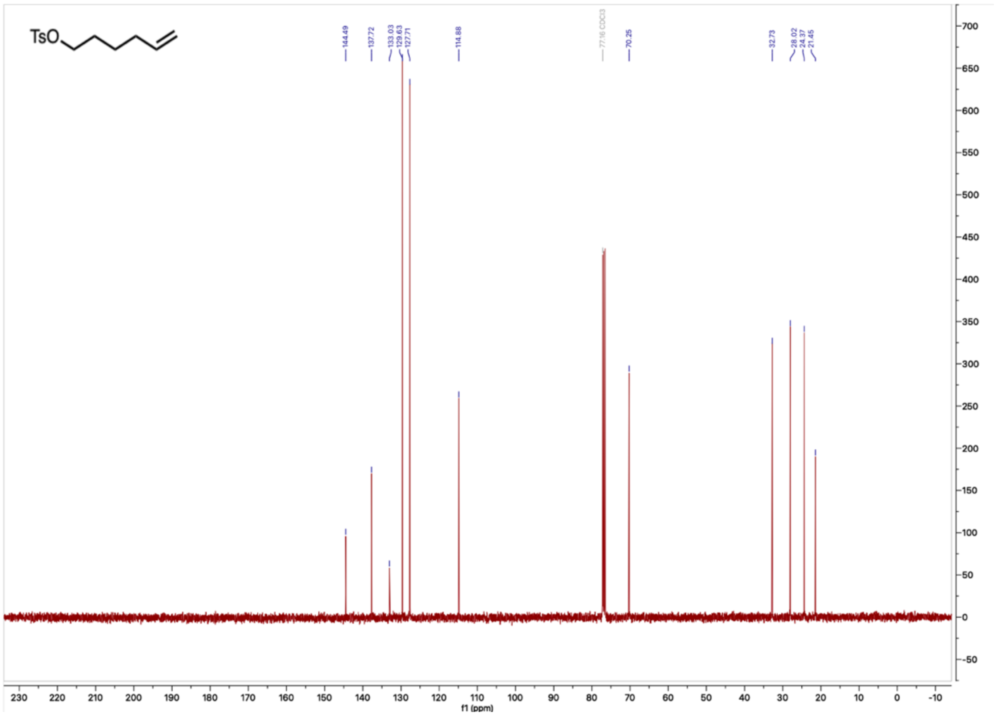

1w ^{1}H NMR (400 MHz, $CDCl_3$)

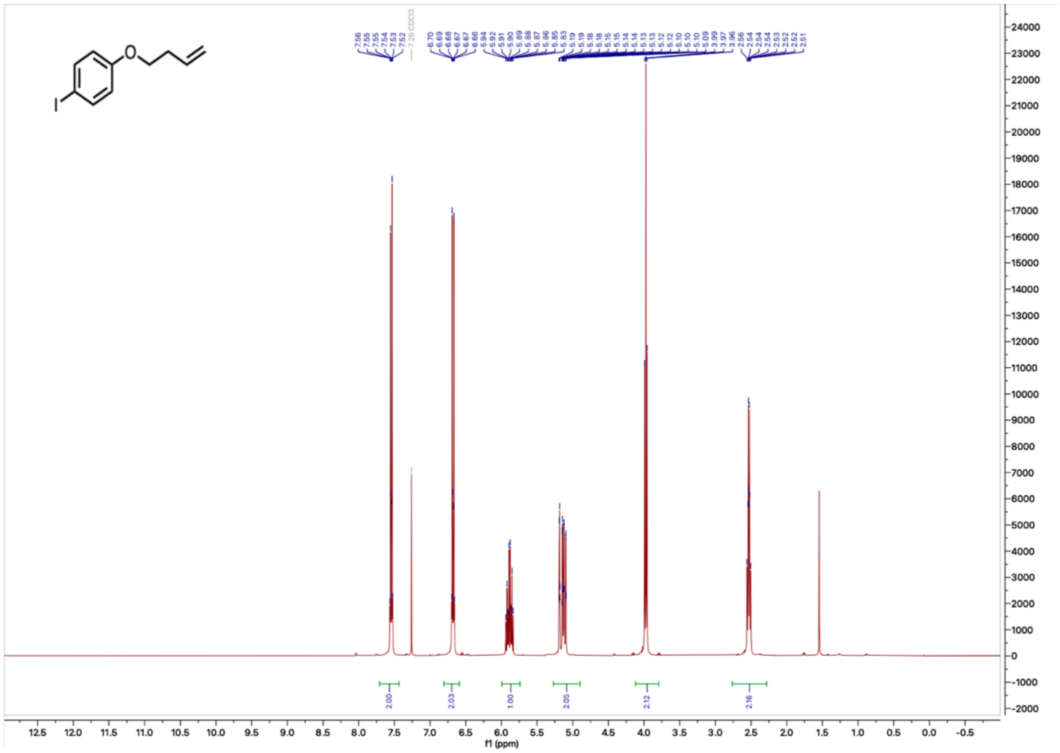

1w ^{13}C NMR (101 MHz, $CDCl_3$)

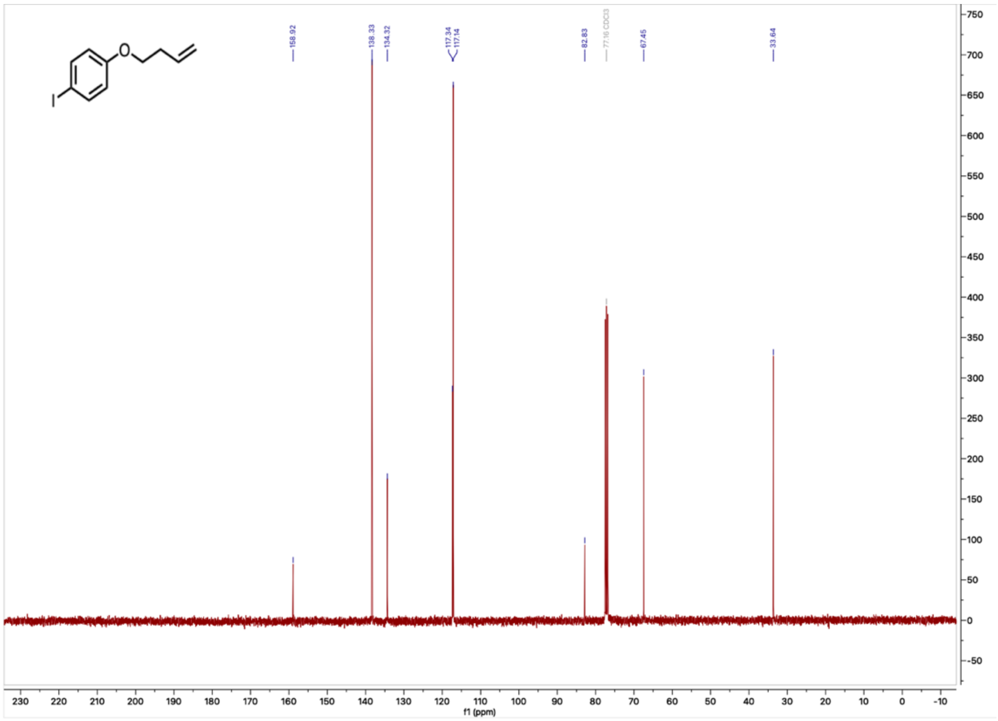

2a ^{1}H NMR (400 MHz, $CDCl_3$

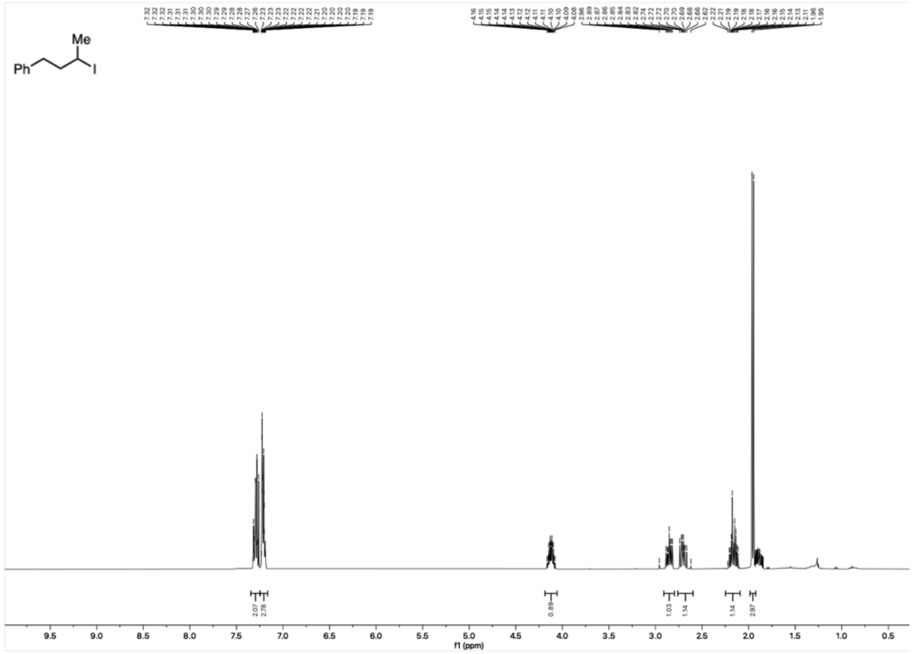

2a ^{13}C NMR (101 MHz, $CDCl_3$)

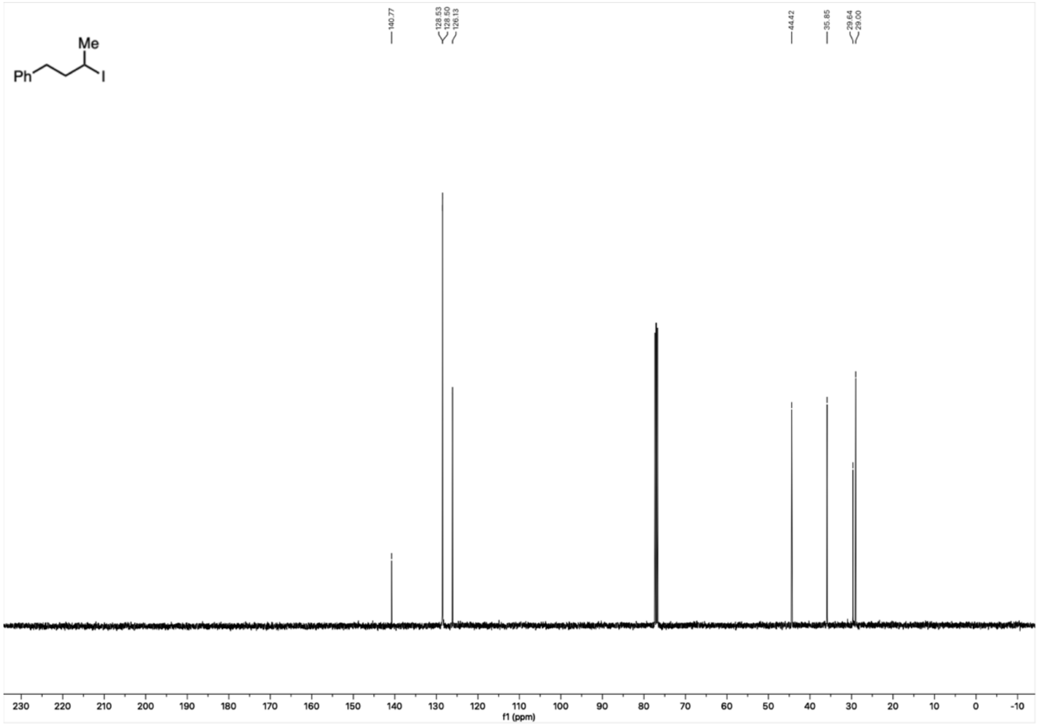

2b ^{1}H NMR (400 MHz, $CDCl_3$)

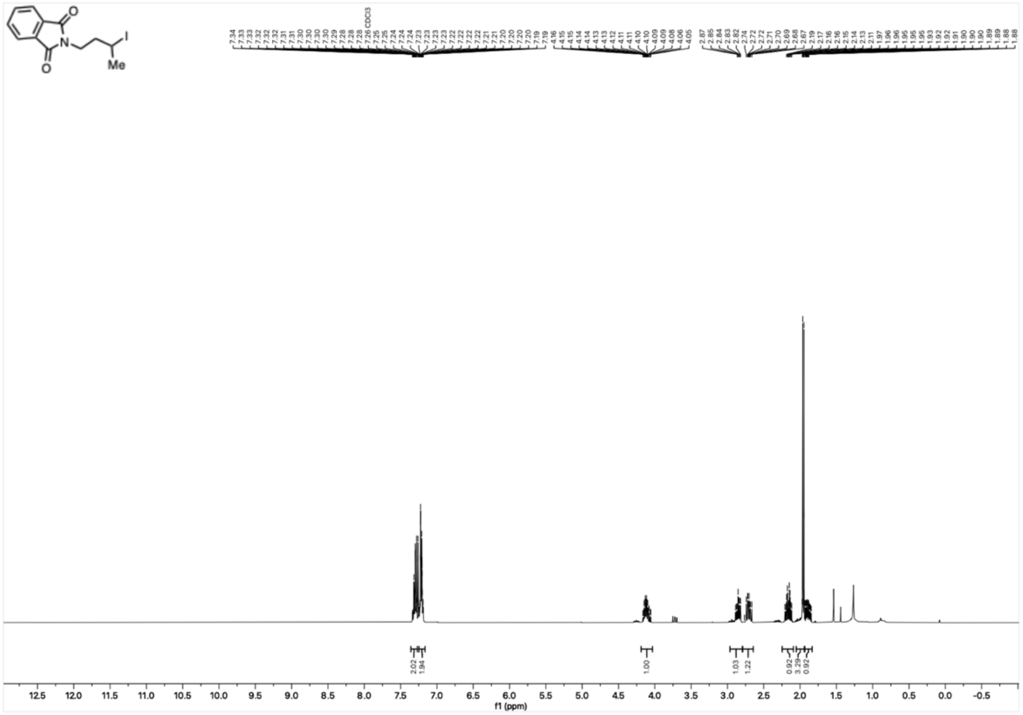

2b ^{13}C NMR (101 MHz, $CDCl_3$)

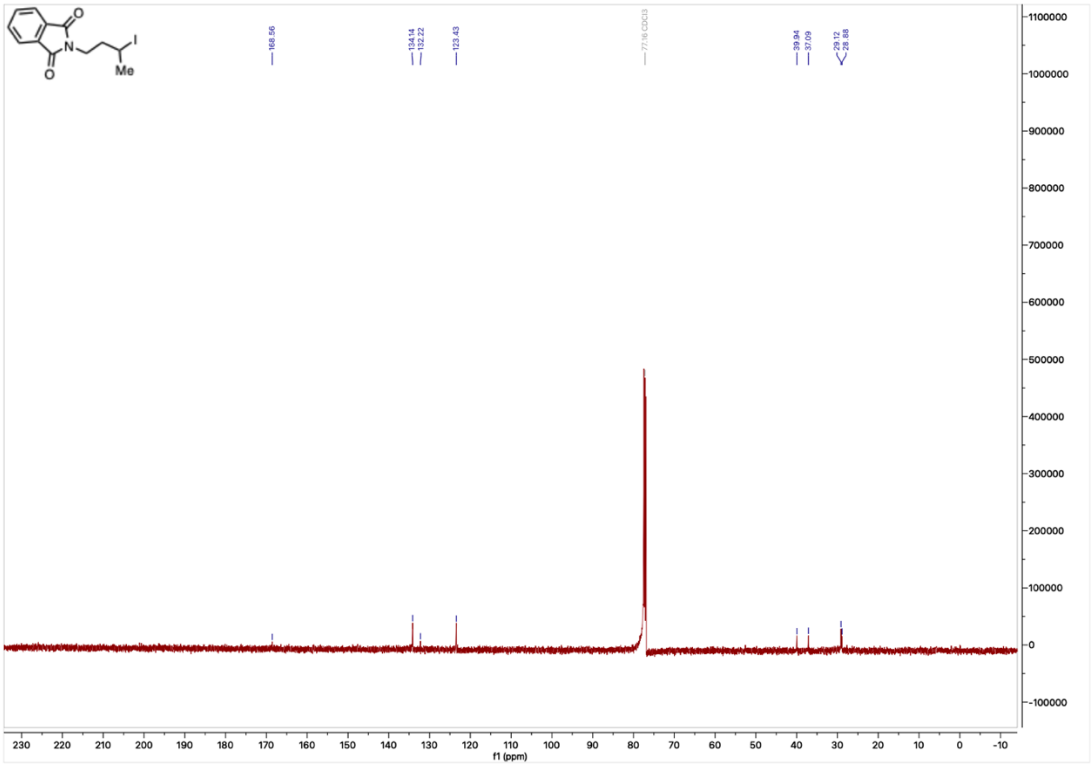

2c ^{1}H NMR (400 MHz, $CDCl_3$)

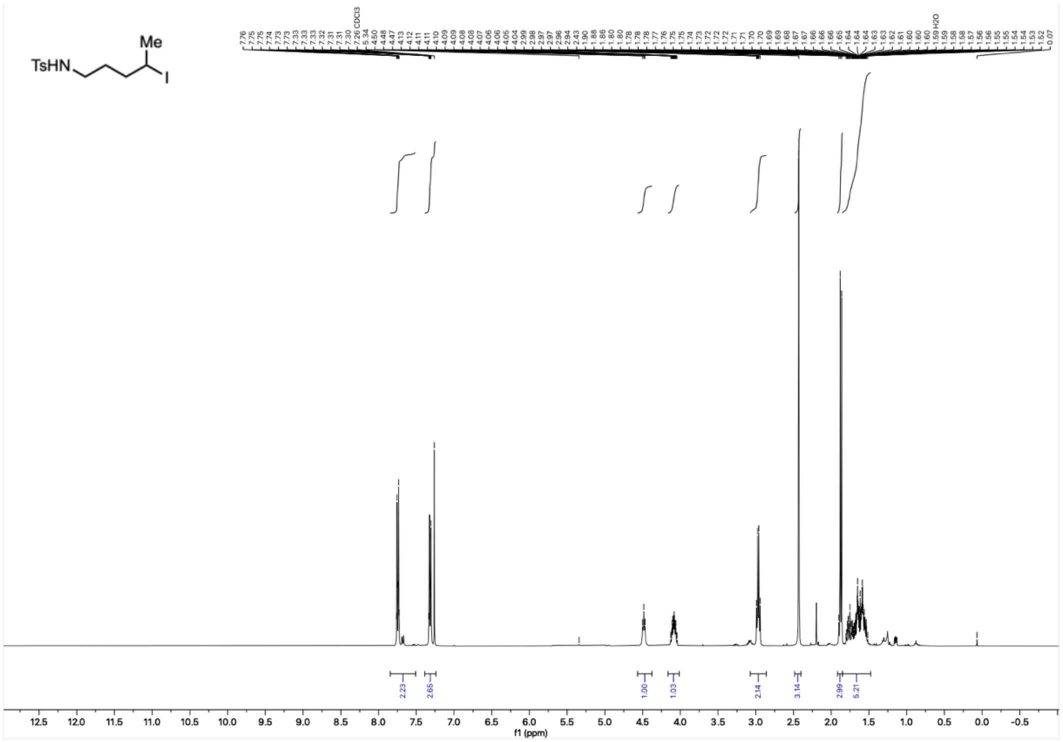

2c ^{13}C NMR (101 MHz, $CDCl_3$)

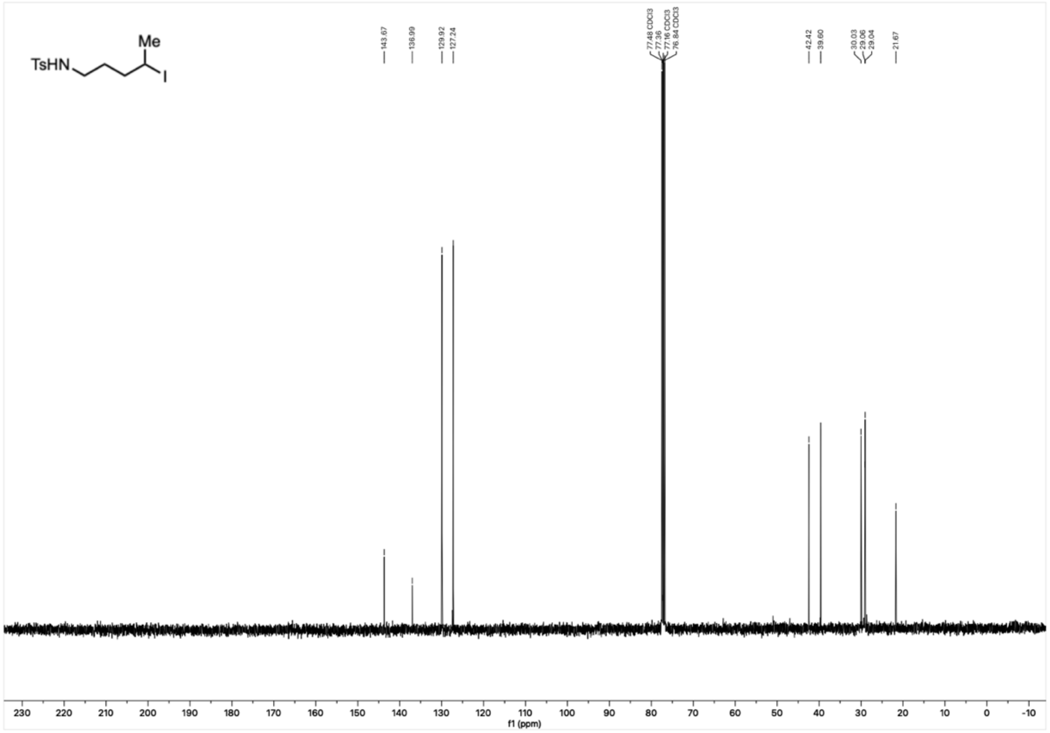

2d ^{1}H NMR (400 MHz, $CDCl_3$)

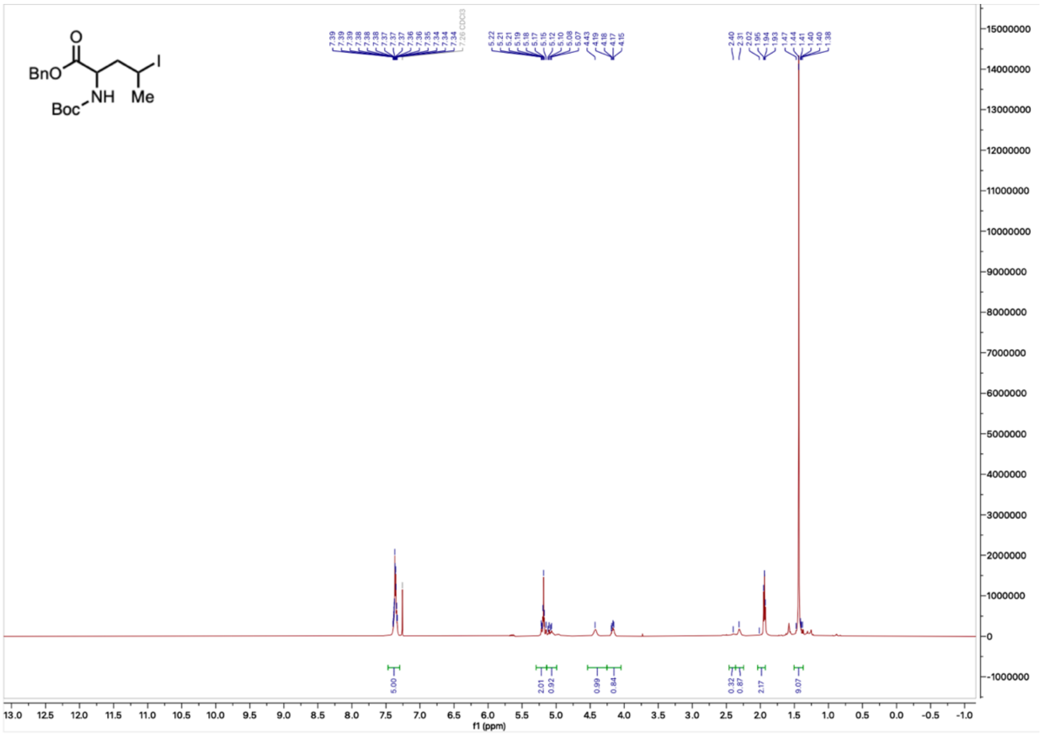

2d ^{13}C NMR (101 MHz, $CDCl_3$)

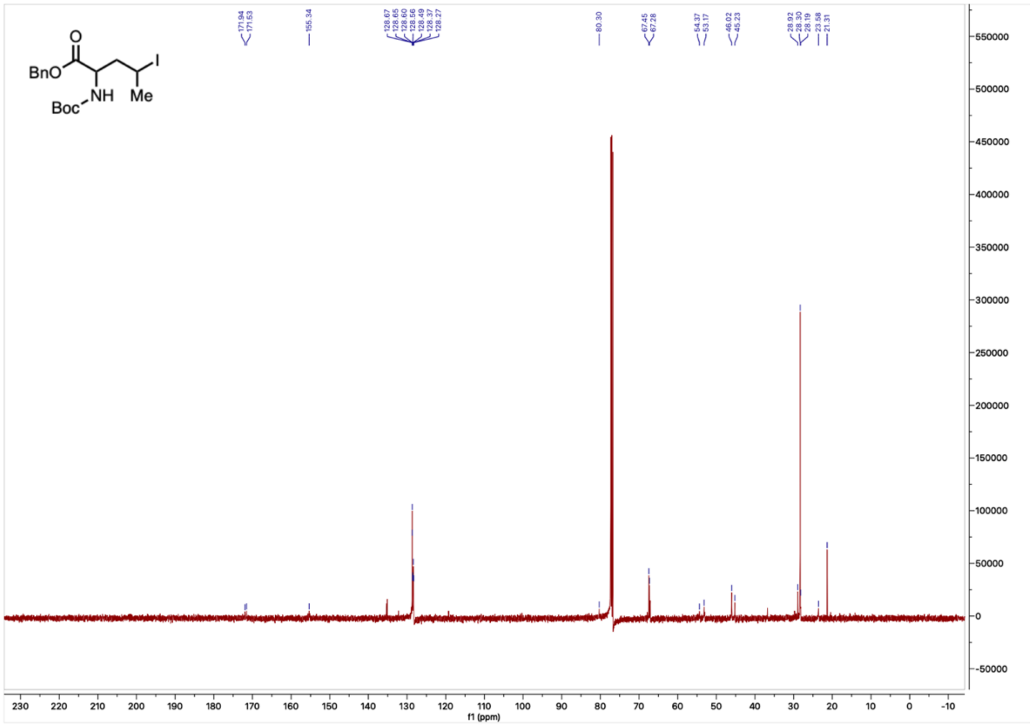

2d ^{1}H–^{13}C HSQC ($CDCl_3$)

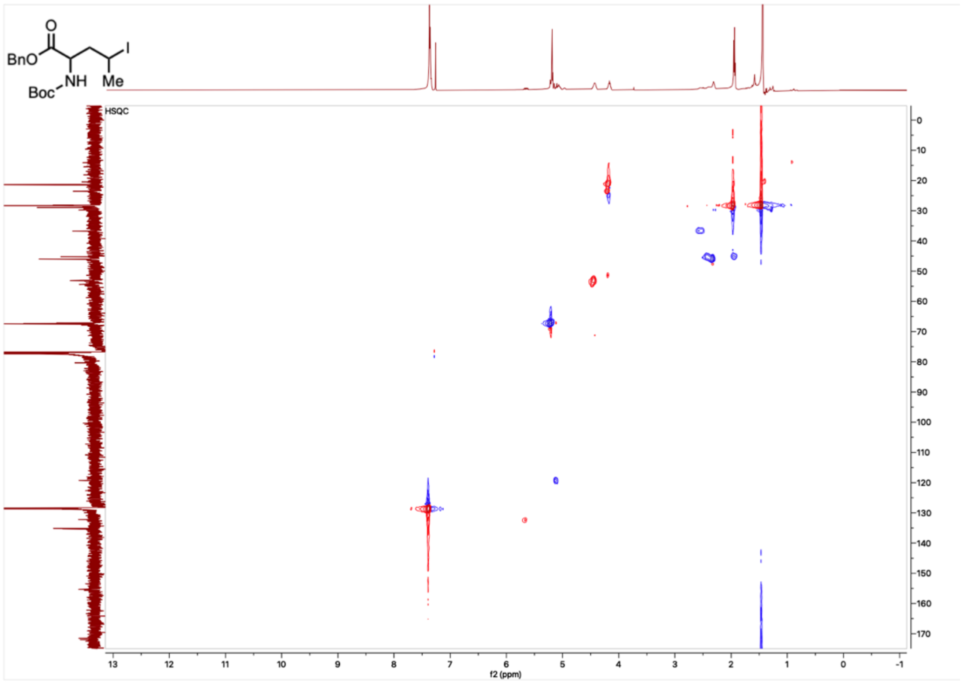

2d ^{1}H–^{1}H COSY ($CDCl_3$)

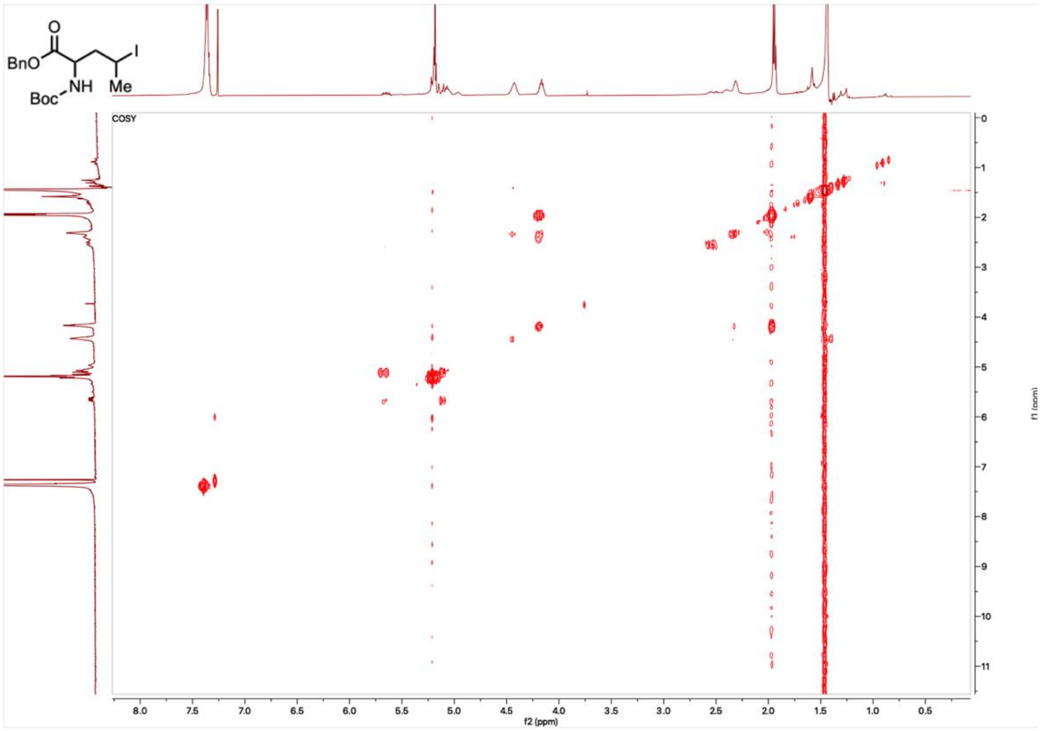

2d ^{1}H–^{13}C HMBC ($CDCl_3$)

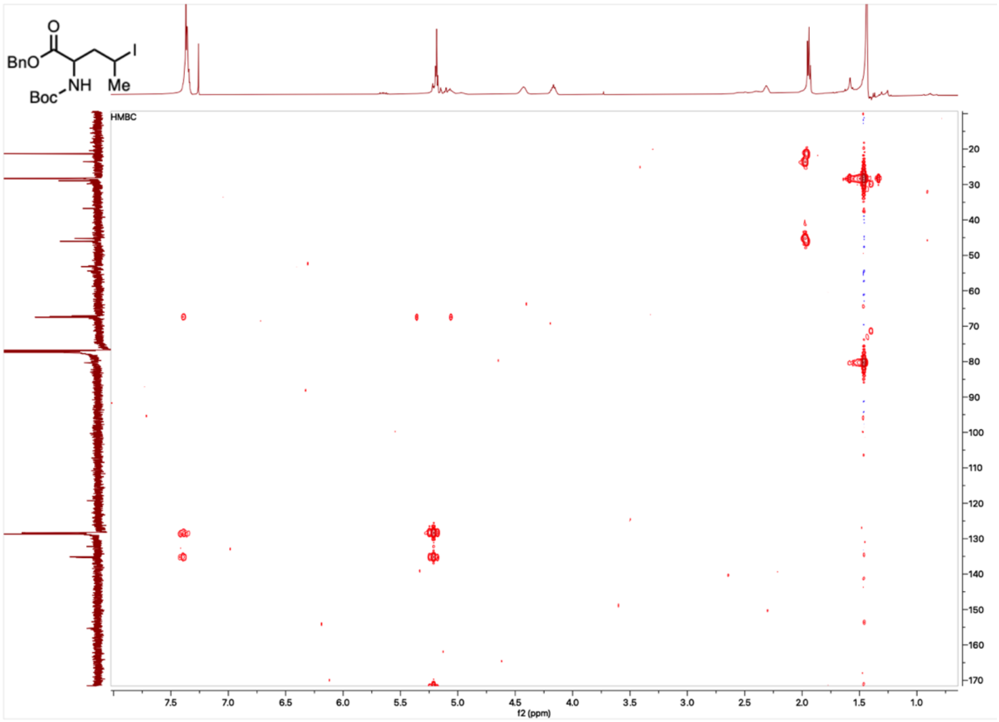

2d ^{1}H–^{1}H NOESY ($CDCl_3$)

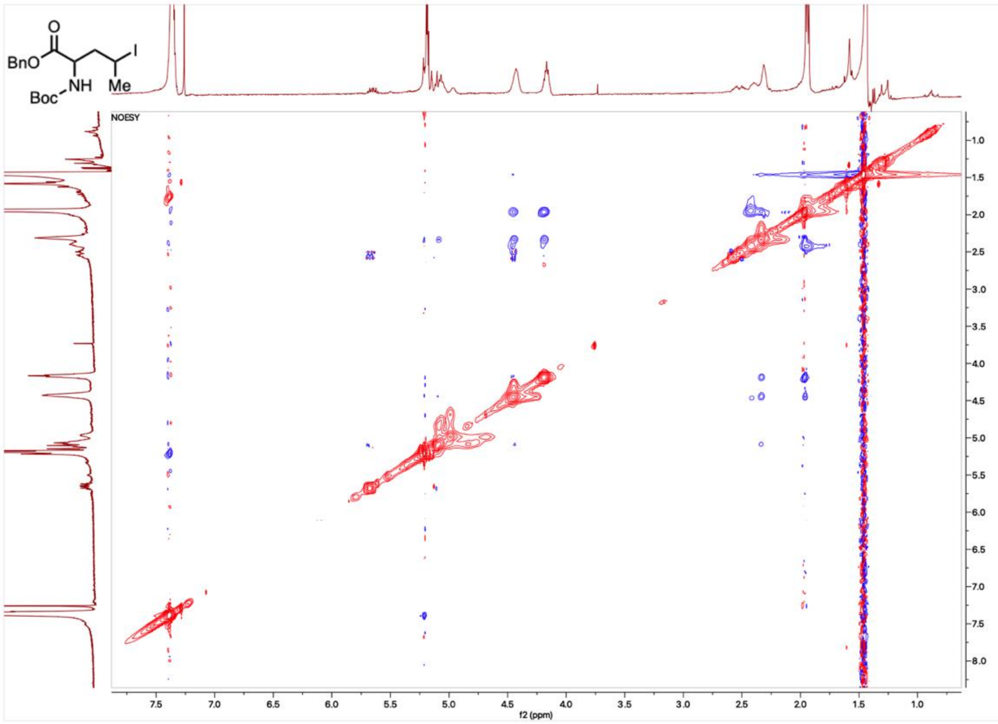

2e ^{1}H (400 MHz, $CDCl_3$)

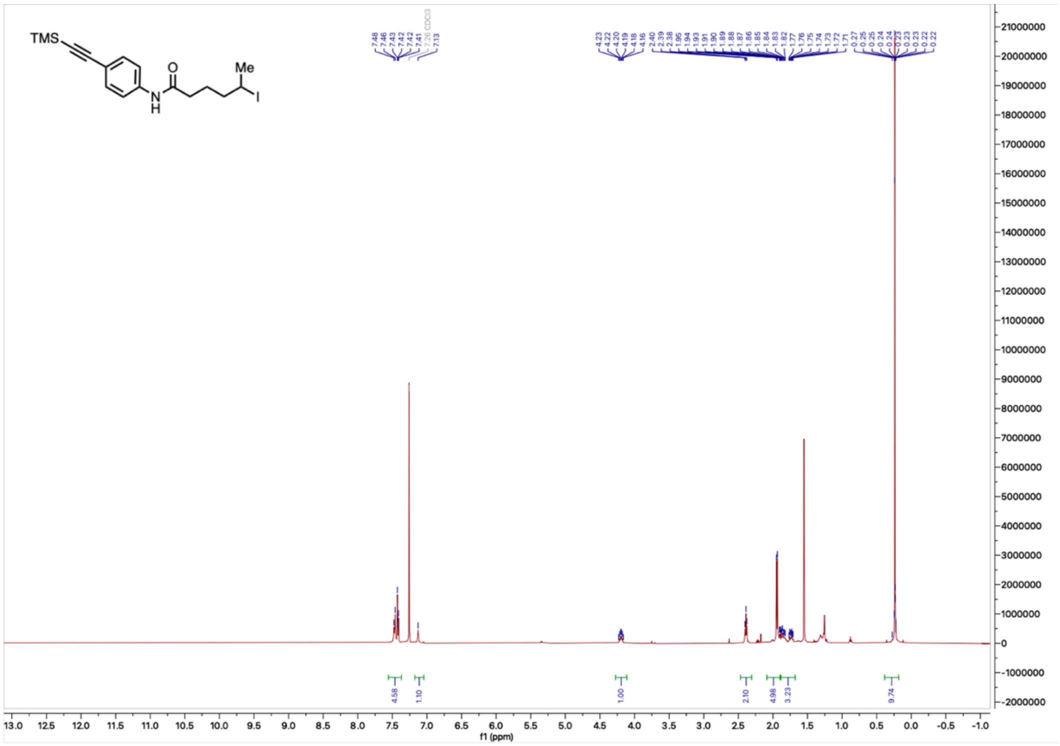

2e ^{13}C NMR (101 MHz, $CDCl_3$)

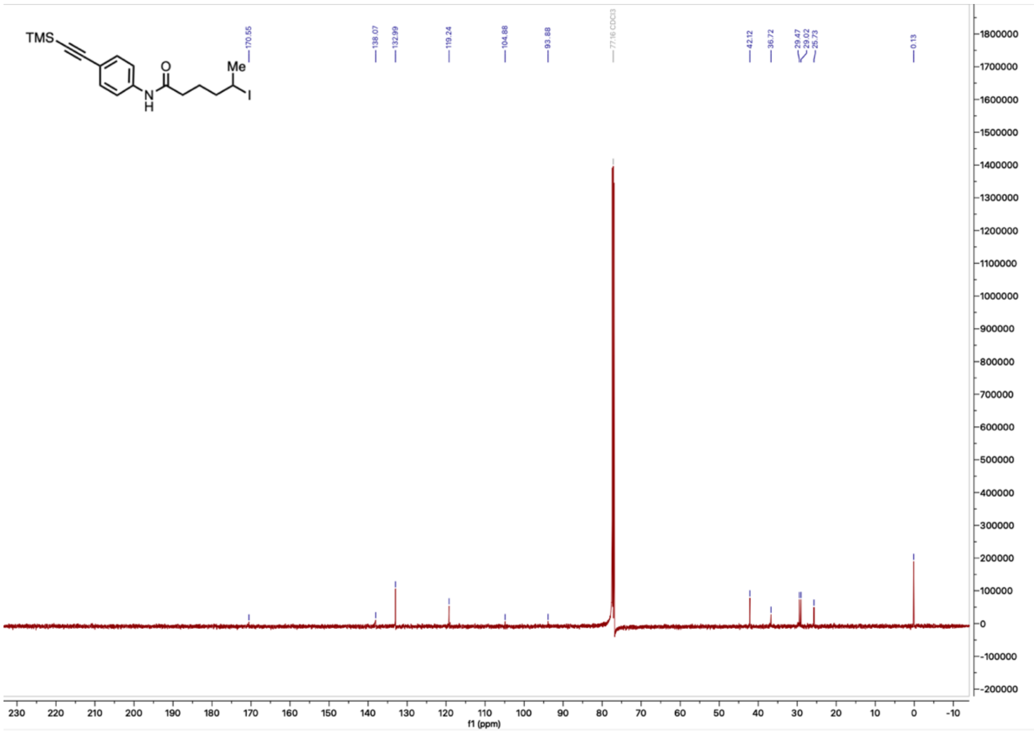

2f ^{1}H (400 MHz, $CDCl_3$)

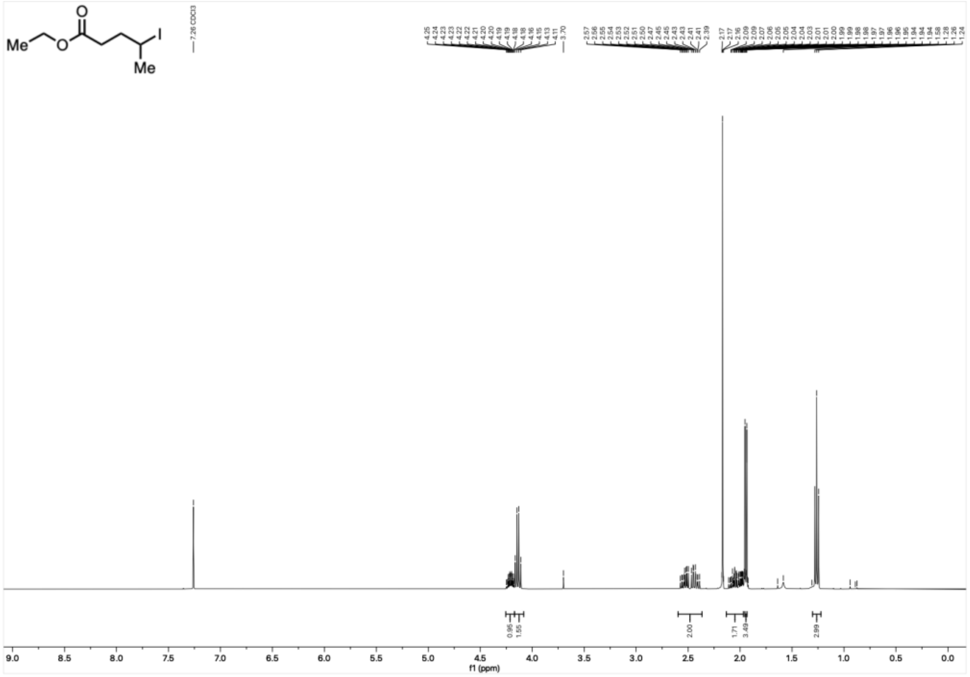

2f ^{13}C NMR (101 MHz, $CDCl_3$)

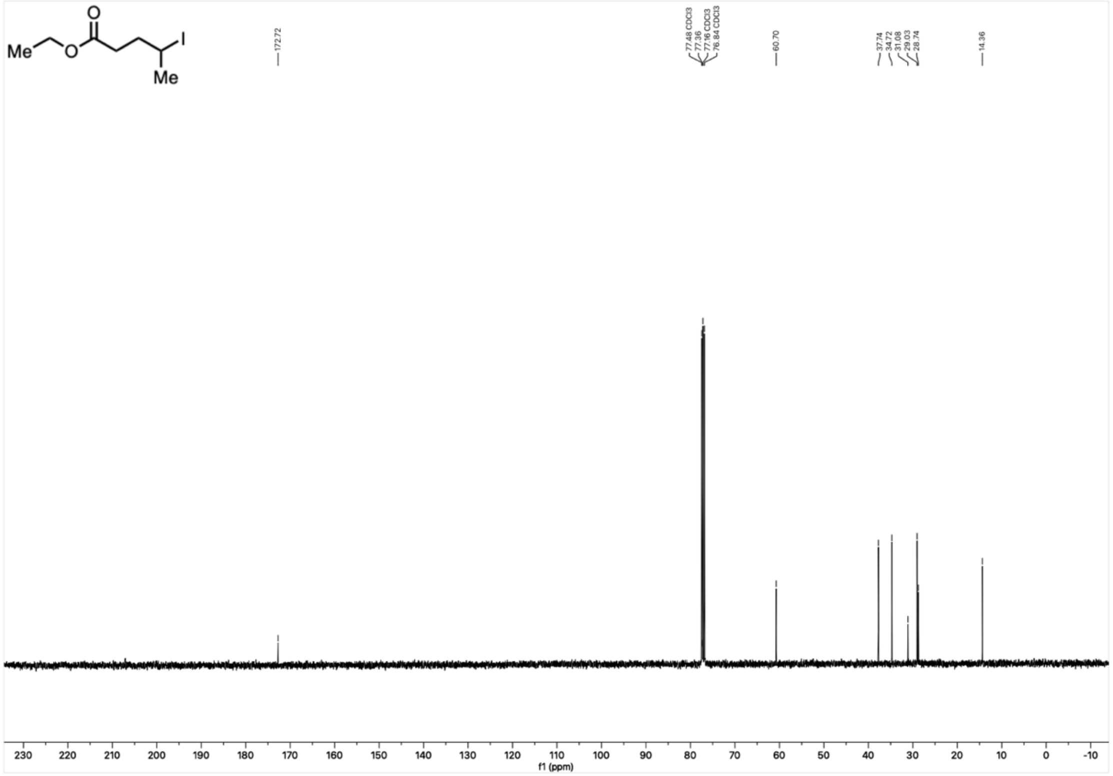

2g ^{1}H (400 MHz, $CDCl_3$)

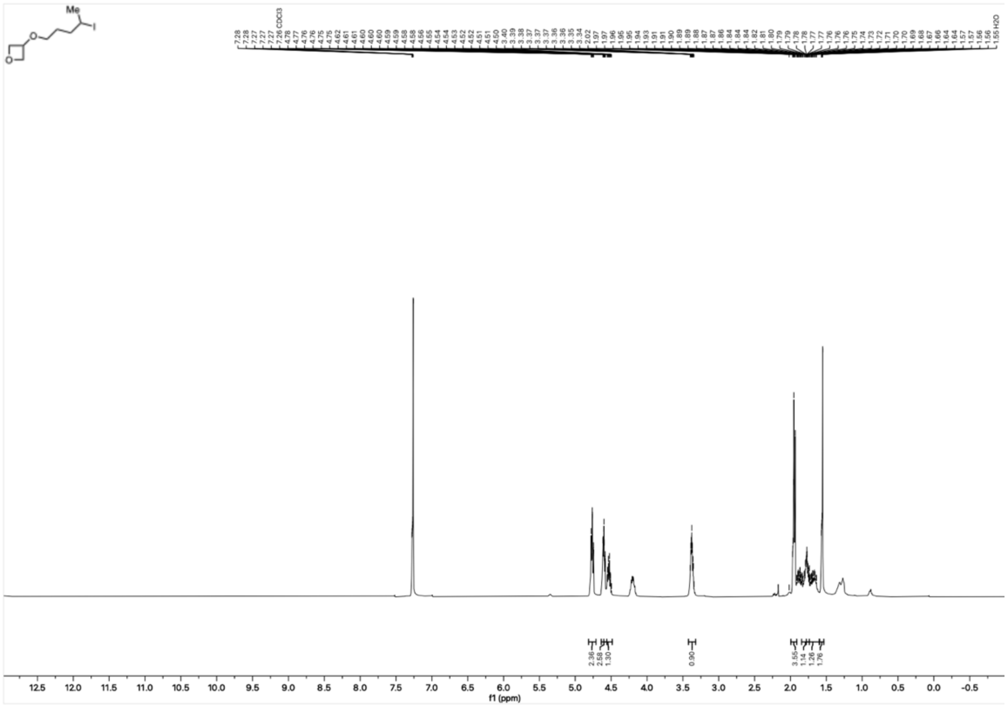

2g ^{13}C NMR (101 MHz, $CDCl_3$)

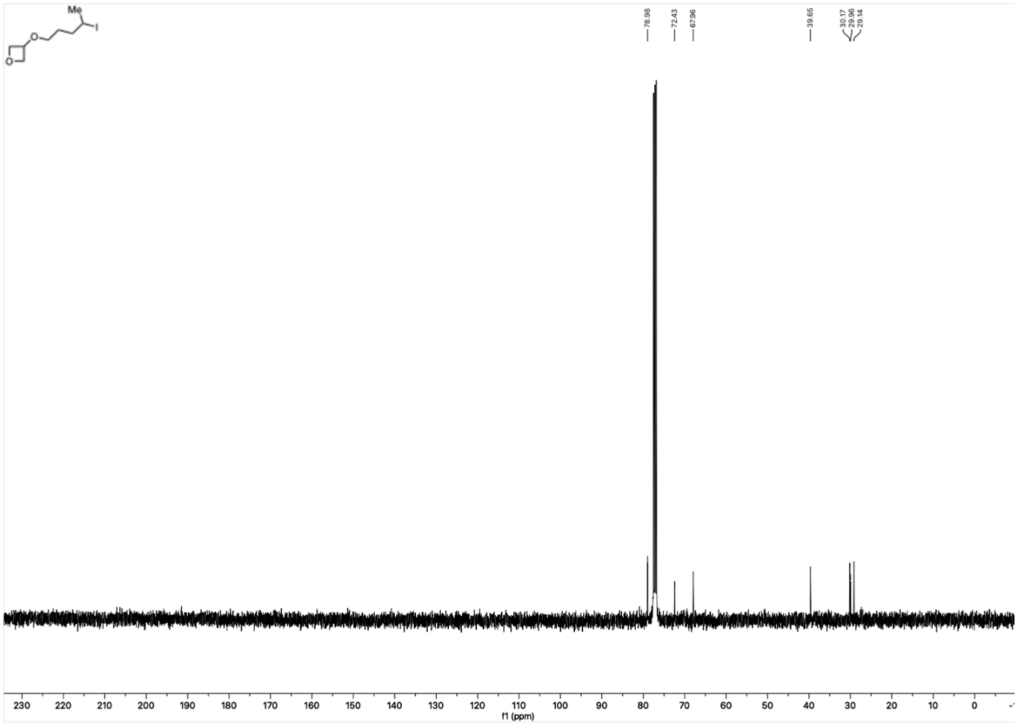

2h ^{1}H (400 MHz, $CDCl_3$)

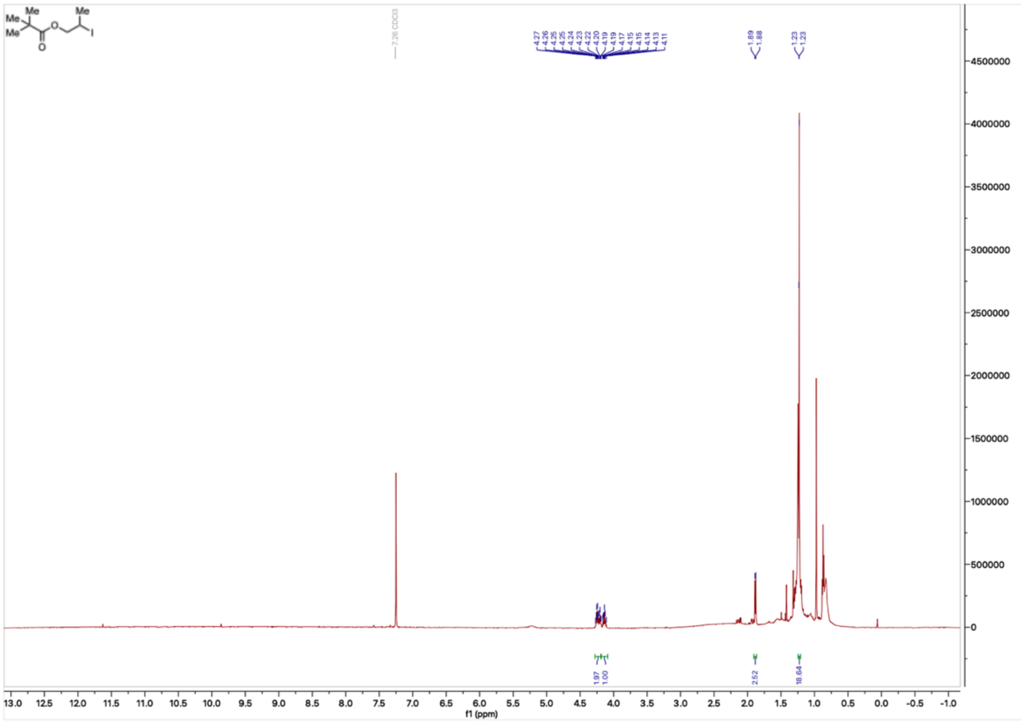

2h ^{13}C NMR (101 MHz, $CDCl_3$)

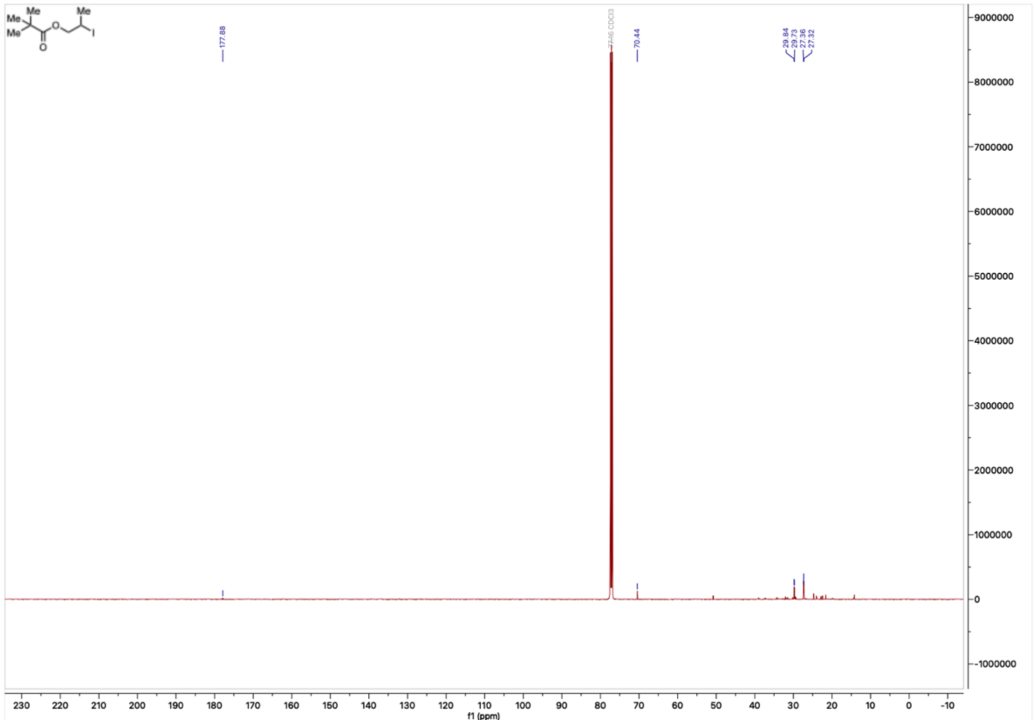

2i ^{1}H (400 MHz, $CDCl_3$)

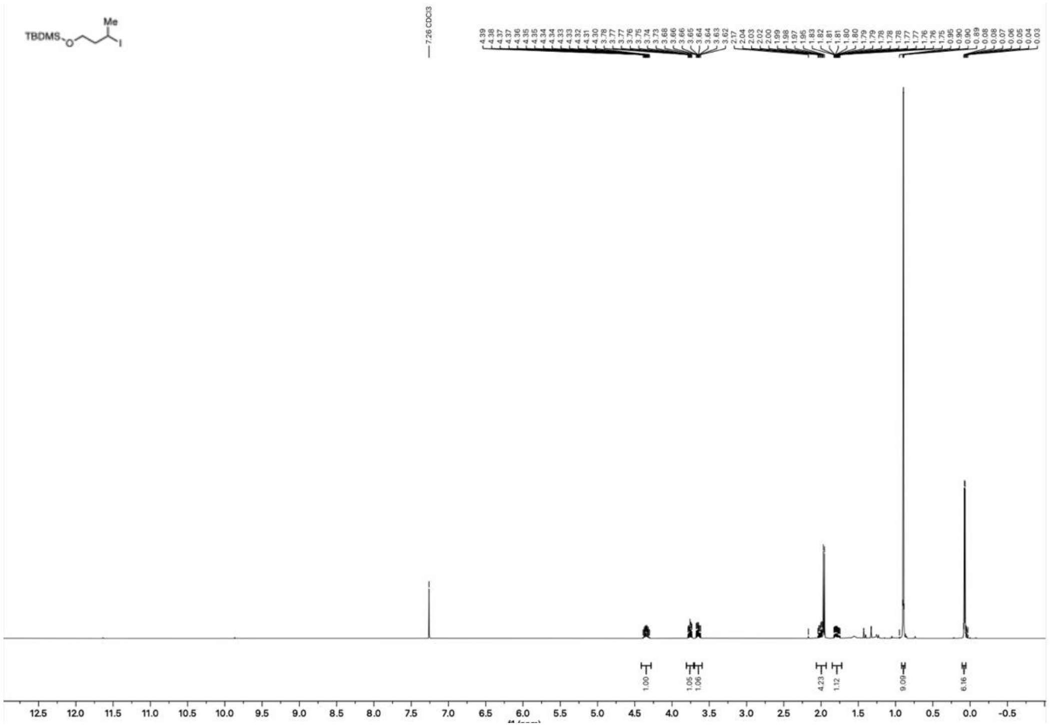

2i ^{13}C NMR (101 MHz, $CDCl_3$)

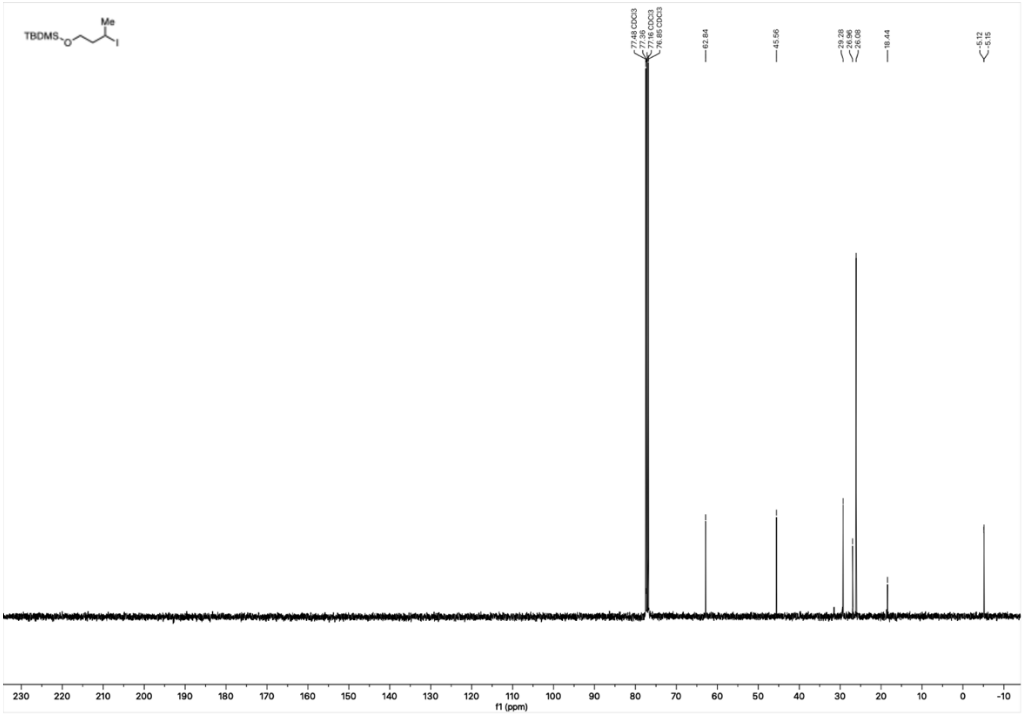

2j ^{1}H (400 MHz, $CDCl_3$)

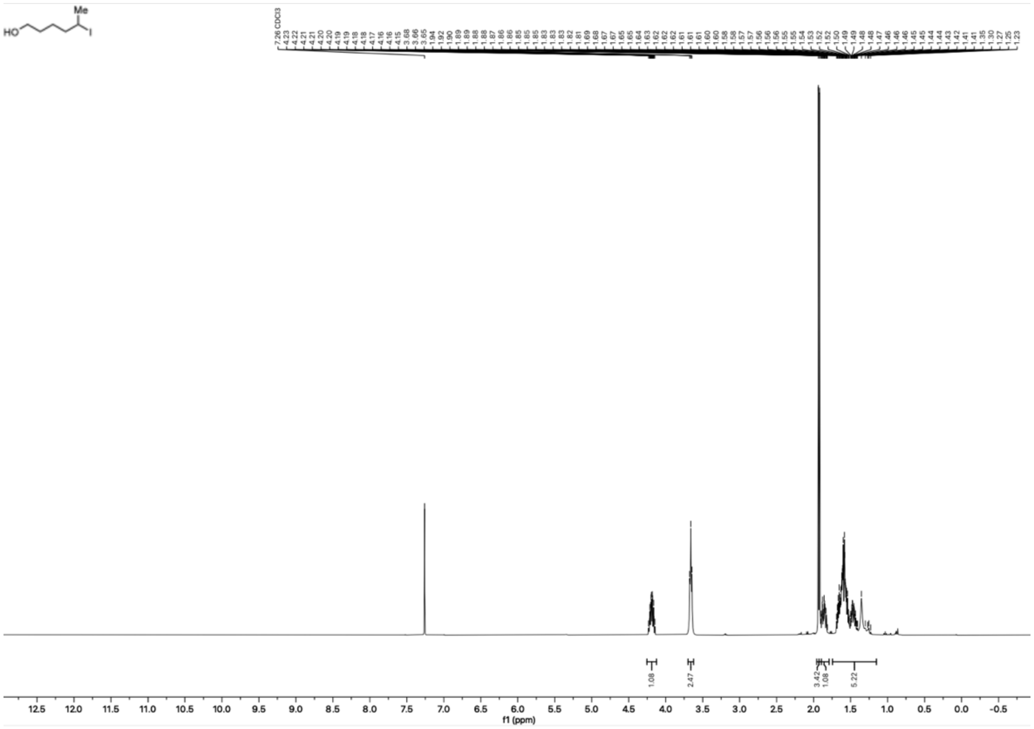

2j ^{13}C NMR (101 MHz, $CDCl_3$)

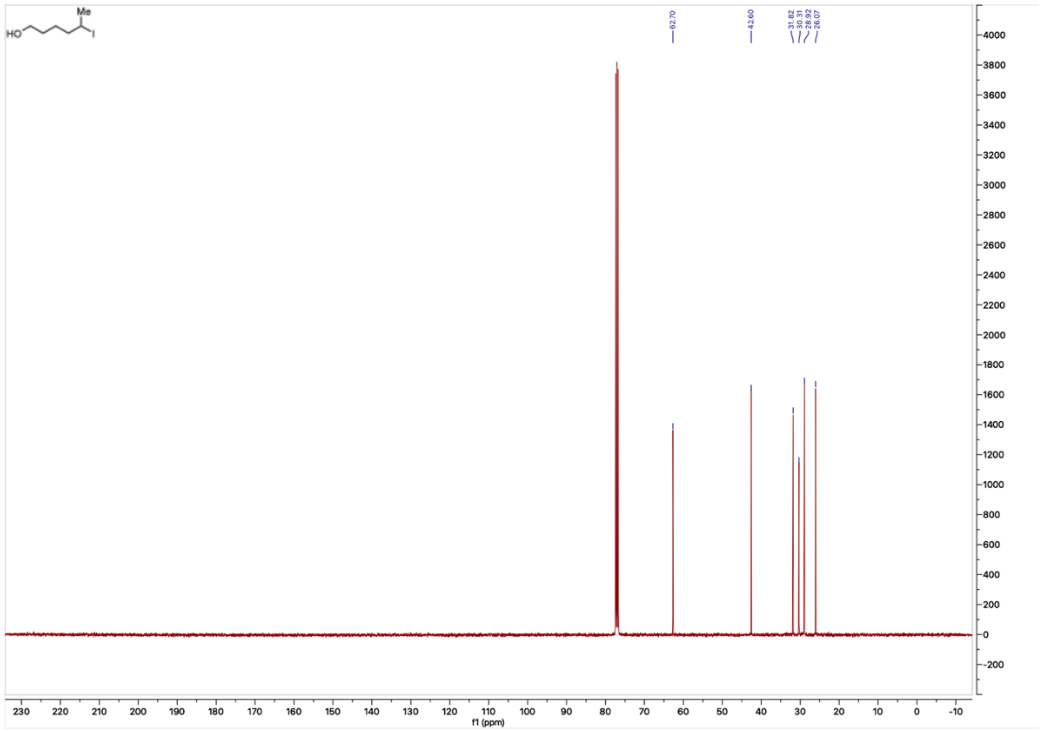

2k ^{1}H (400 MHz, $CDCl_3$)

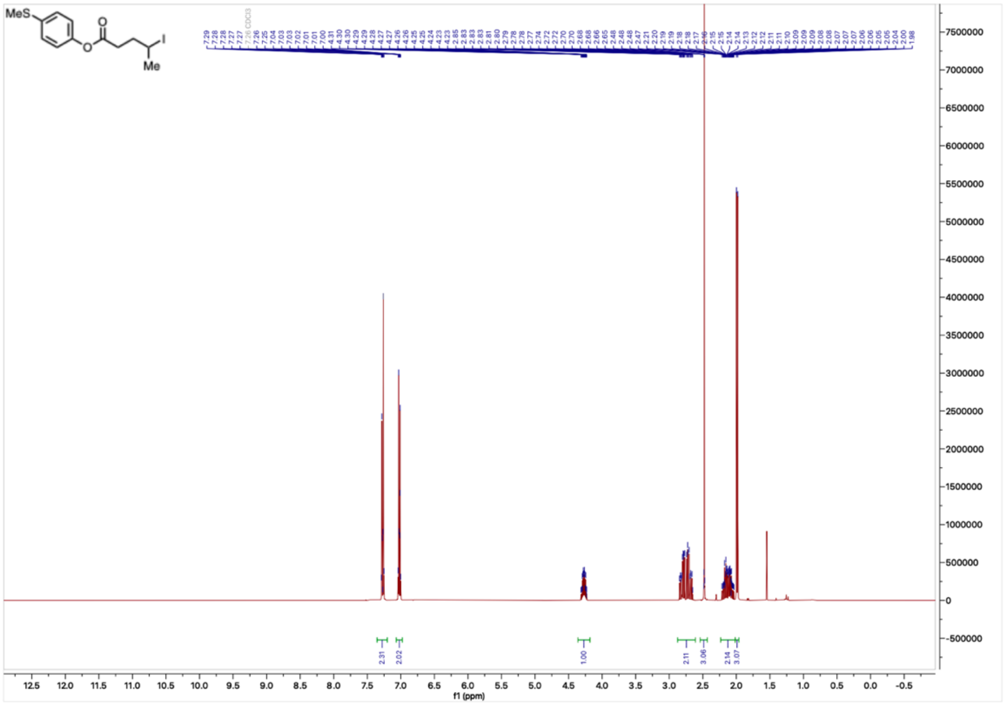

2k ^{13}C NMR (101 MHz, $CDCl_3$)

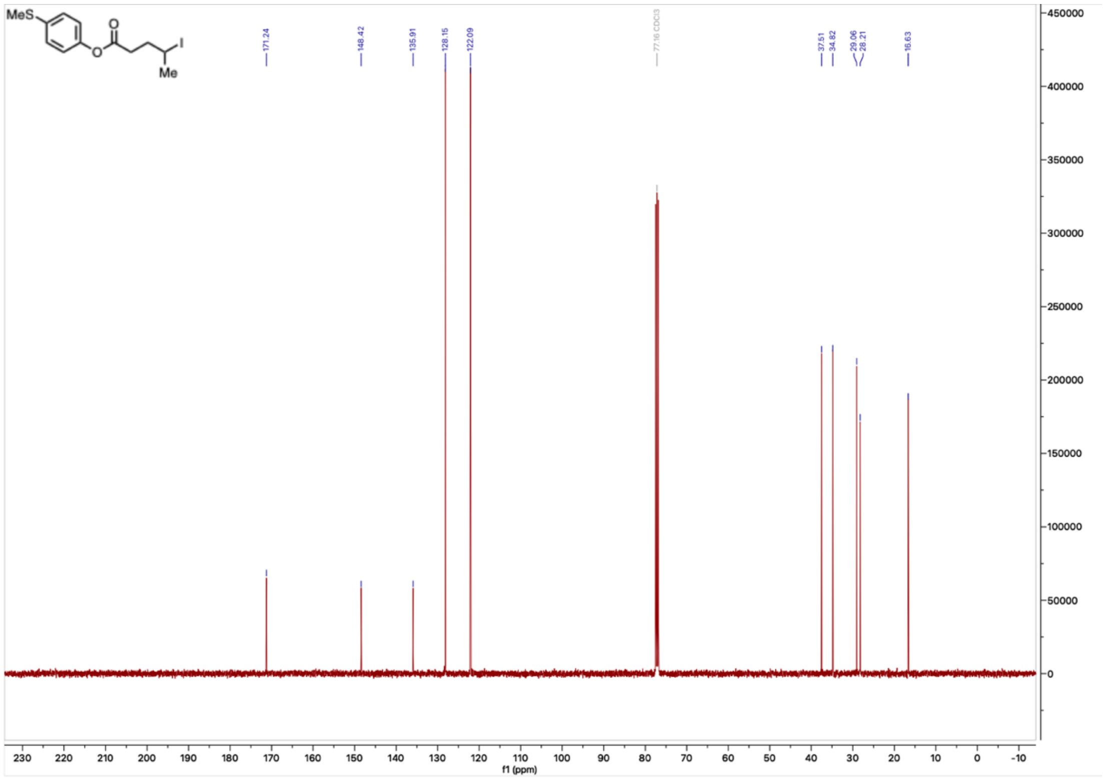

2l ^{1}H (400 MHz, $CDCl_3$)

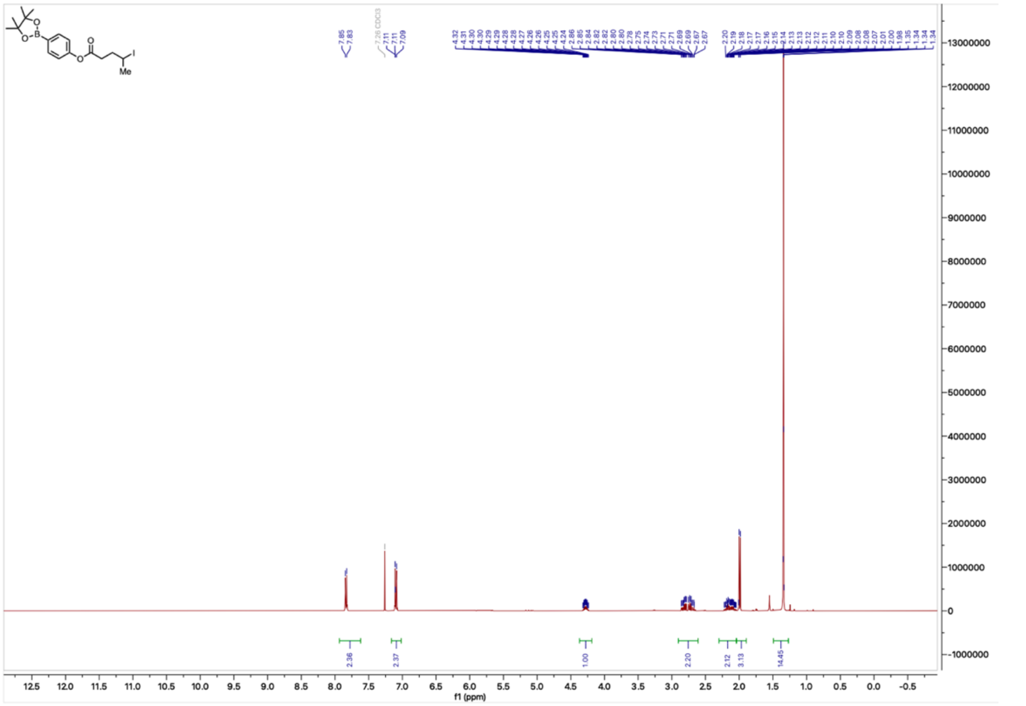

2l ^{13}C NMR (101 MHz, $CDCl_3$)

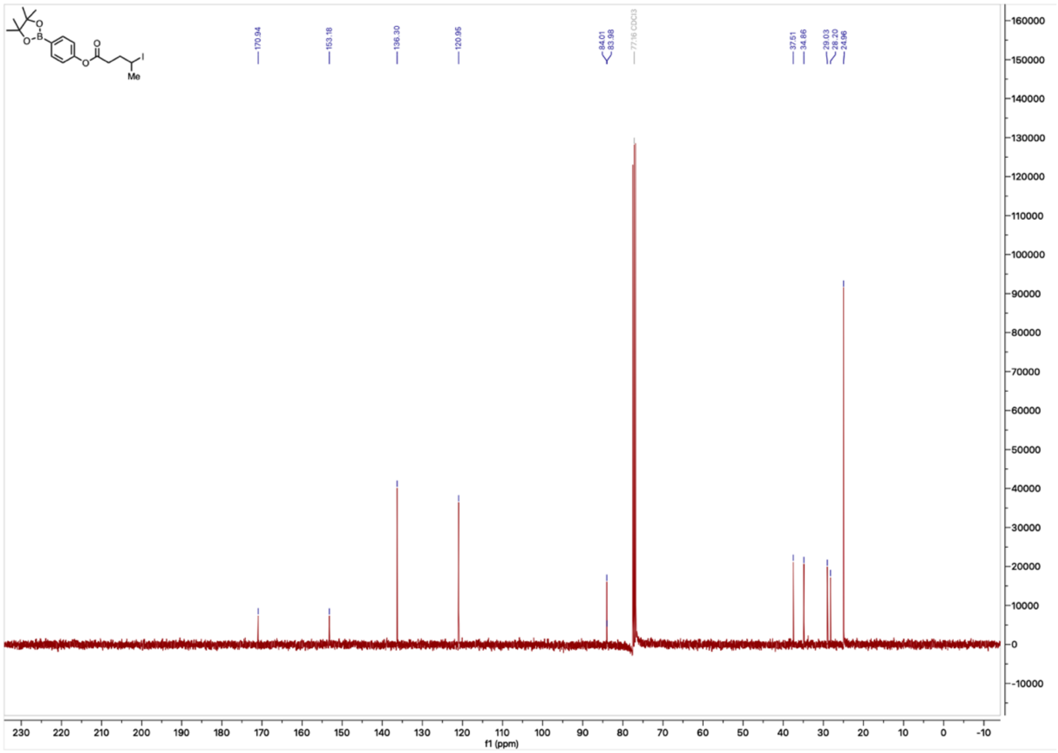

2l ^{11}B NMR (160 MHz, $CDCl_3$)

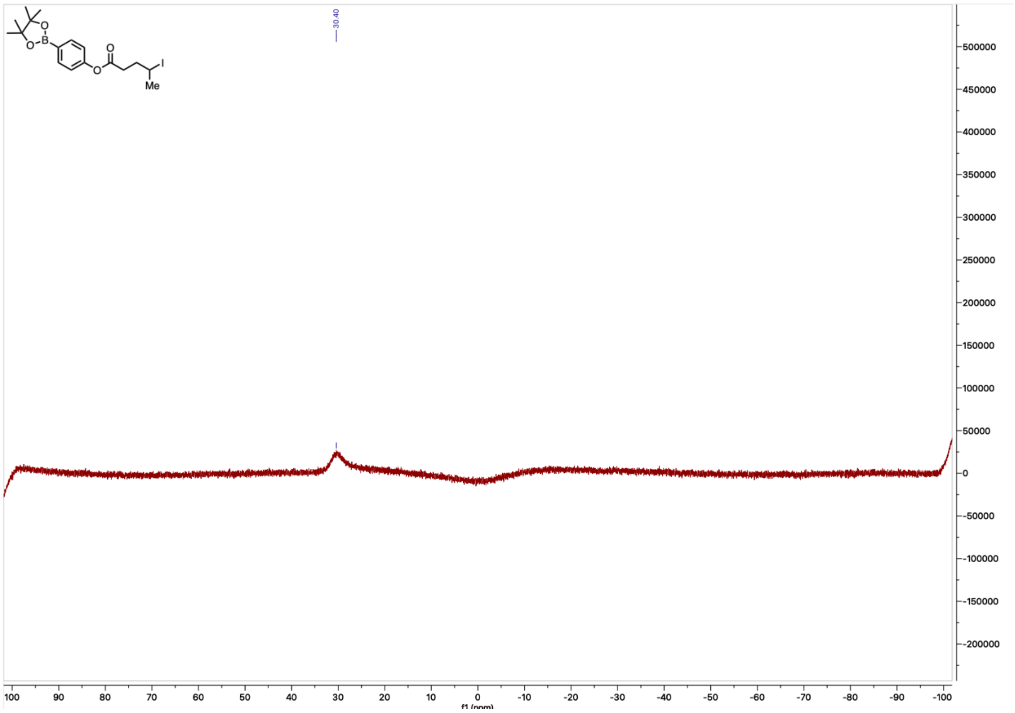

2m ^{1}H (400 MHz, $CDCl_3$)

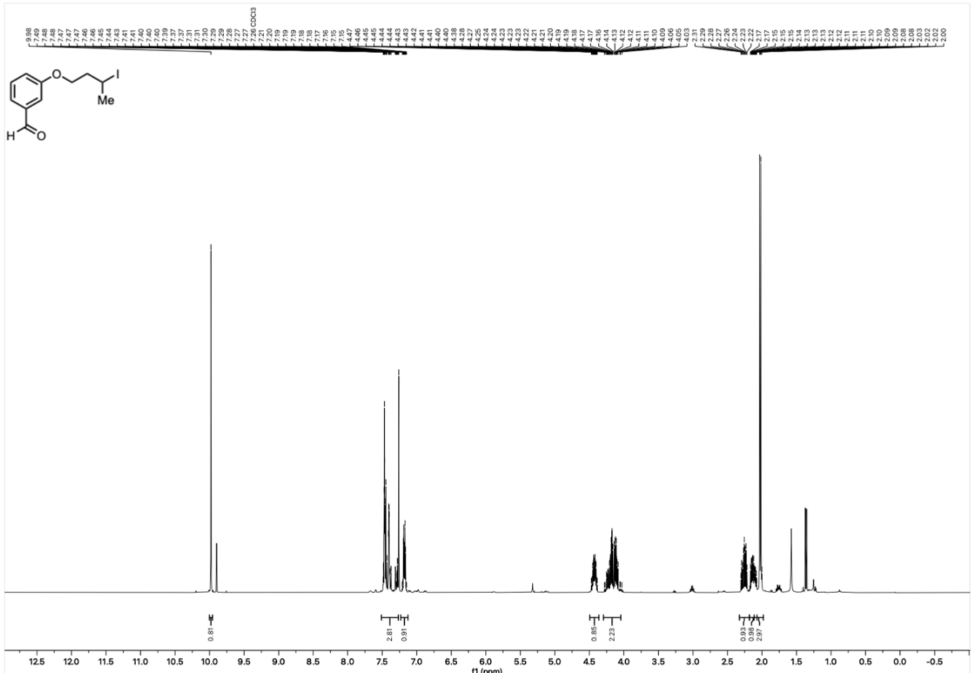

2m ^{13}C NMR (101 MHz, $CDCl_3$)

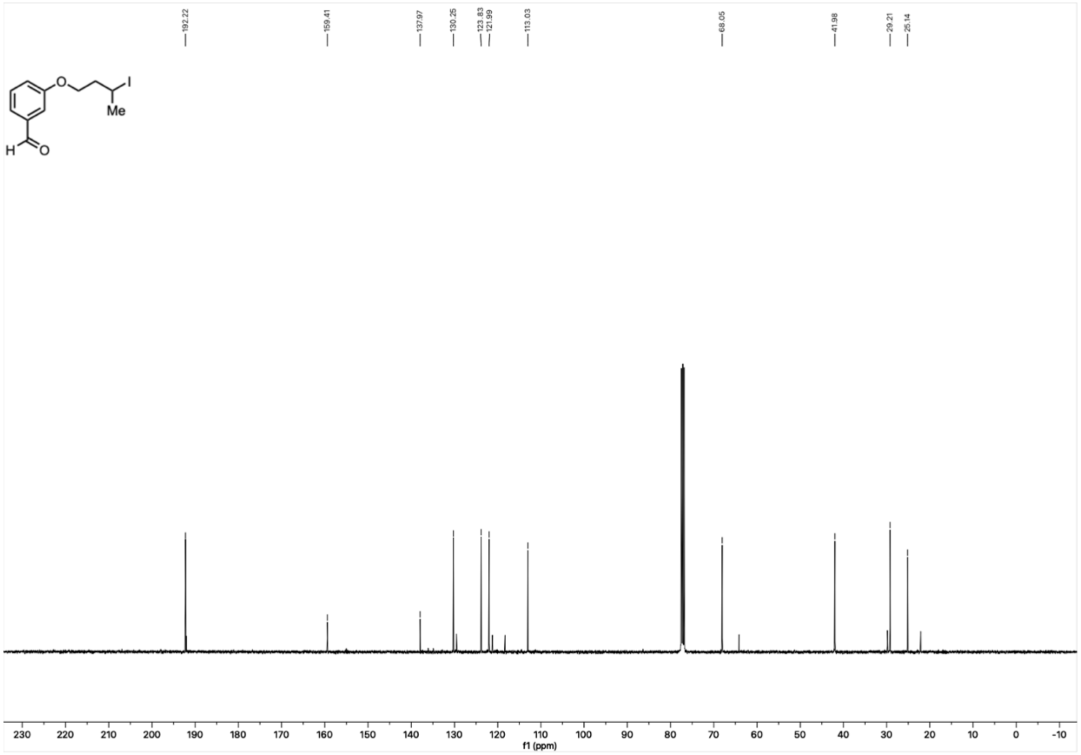

2n ^{1}H (400 MHz, $CDCl_3$)

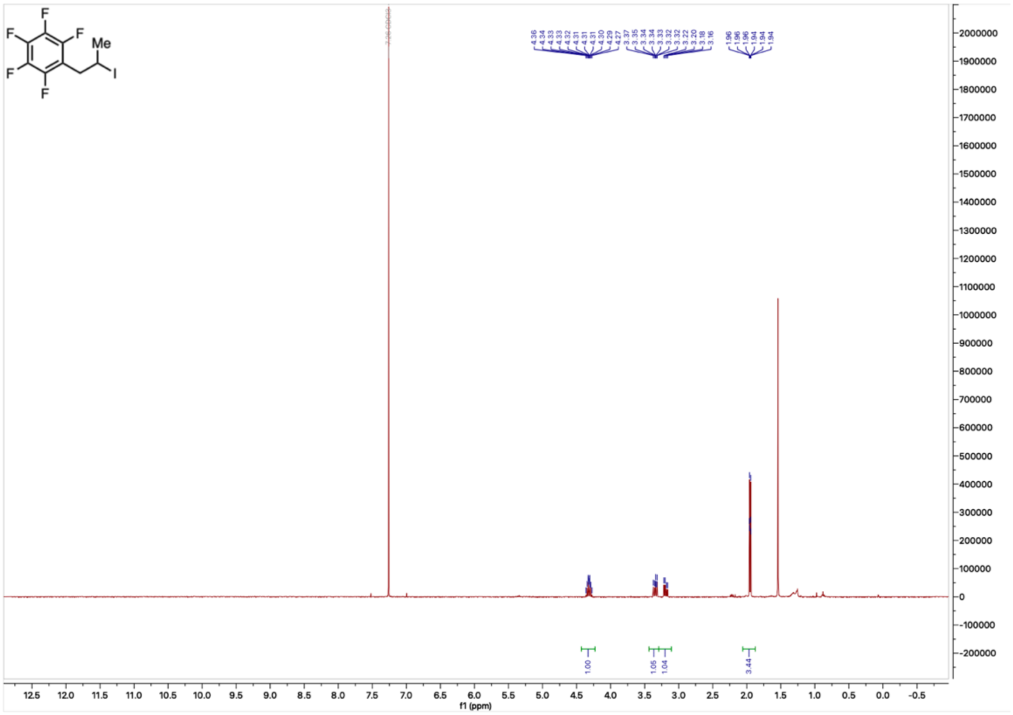

2n ^{19}F NMR (376 MHz, $CDCl_3$)

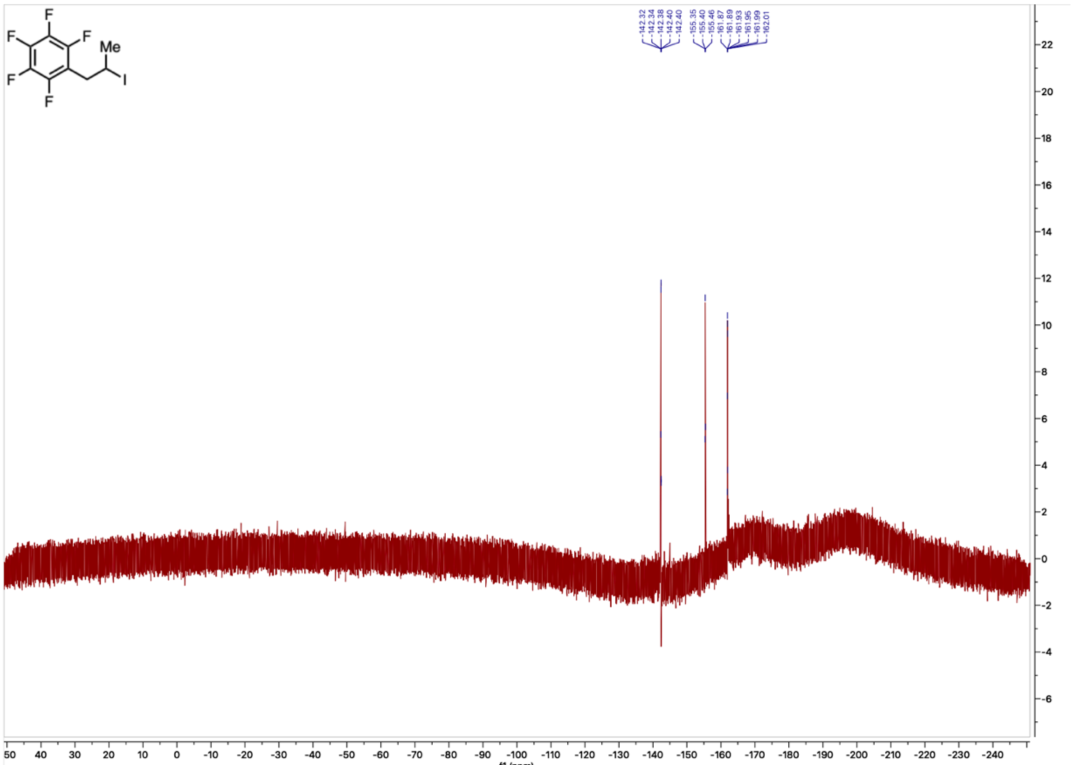

2o ^{1}H (400 MHz, $CDCl_3$)

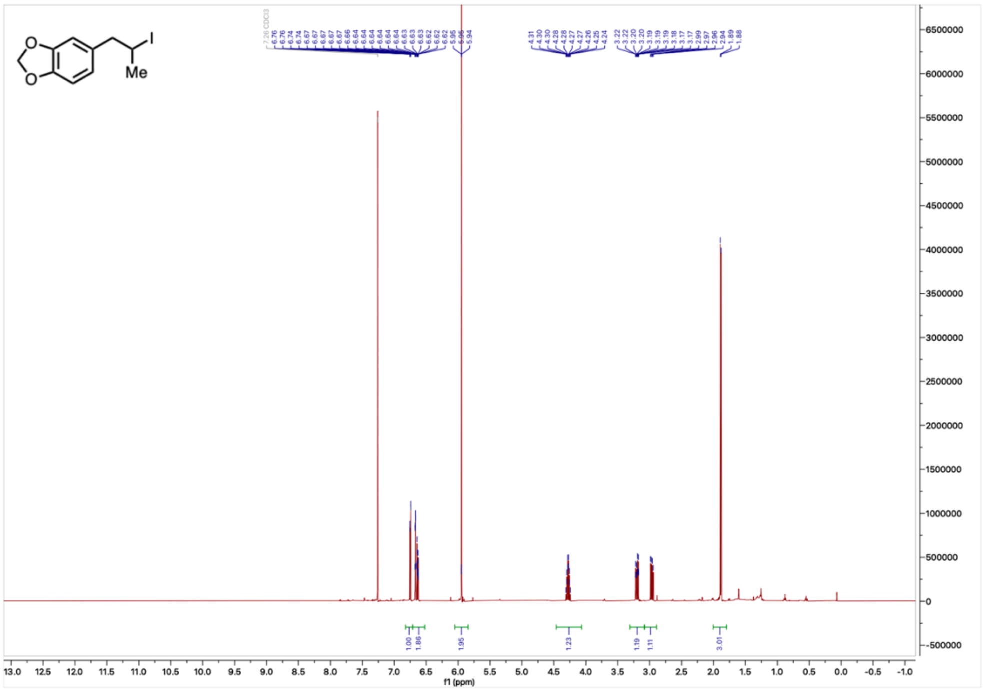

2o ^{13}C NMR (101 MHz, $CDCl_3$)

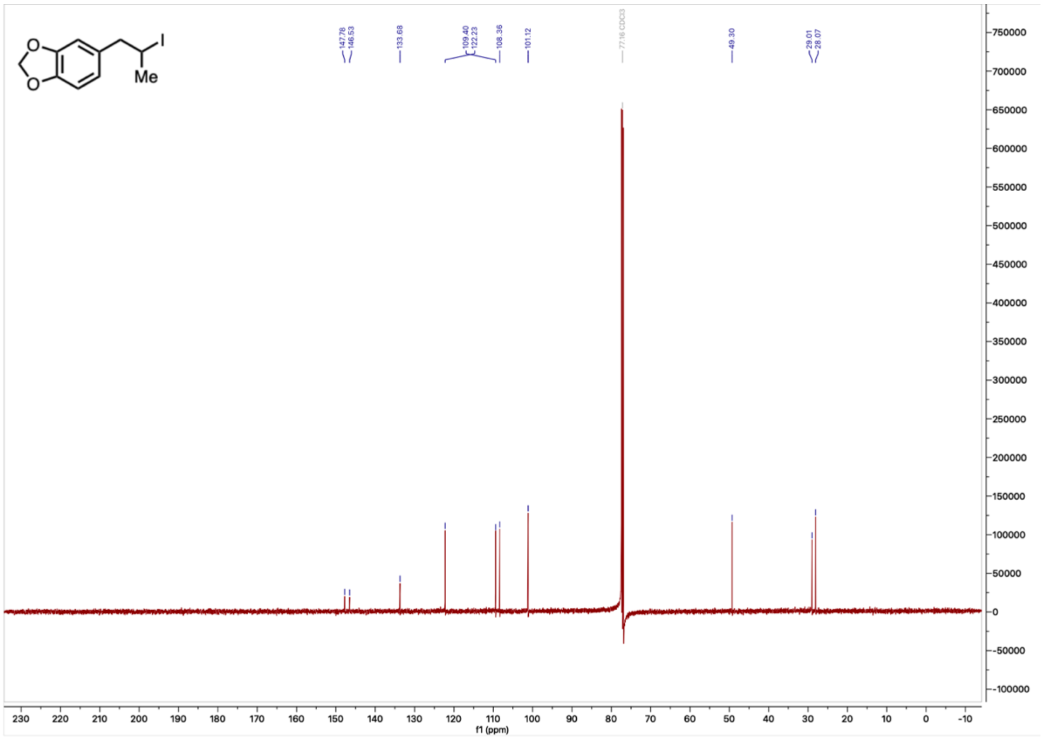

2p ^{1}H (400 MHz, $CDCl_3$)

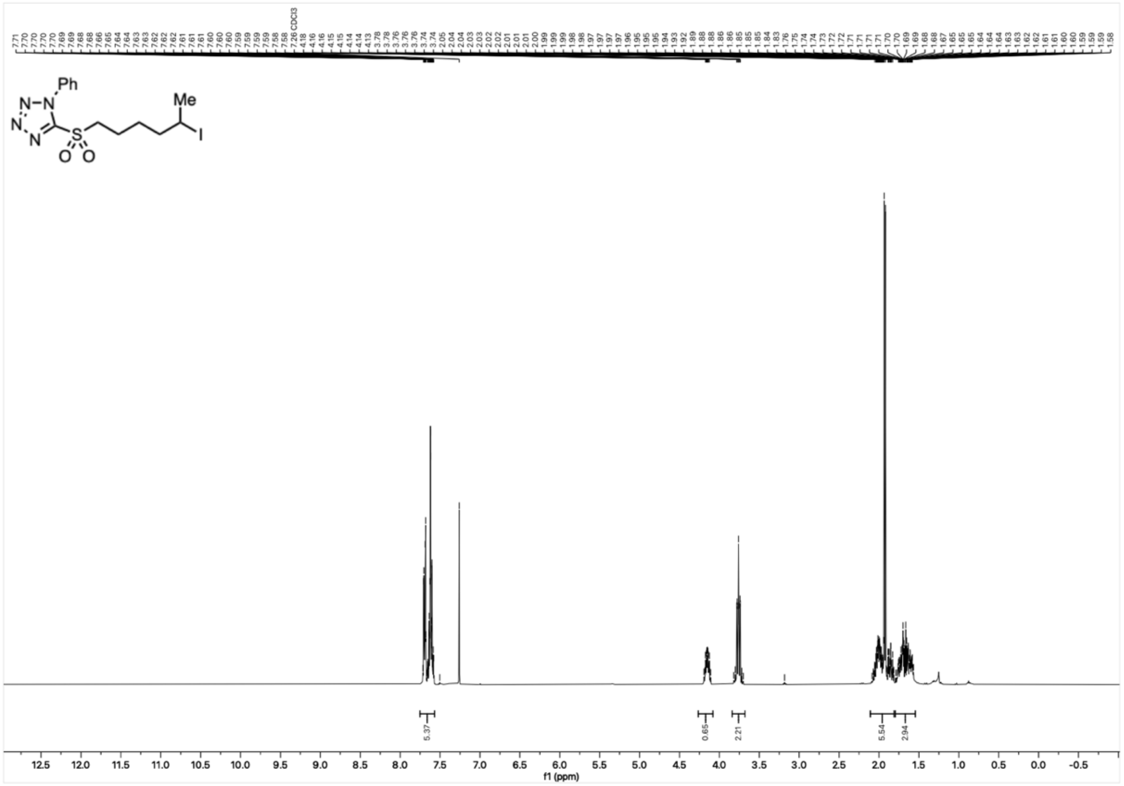

2p ^{13}C NMR (101 MHz, $CDCl_3$)

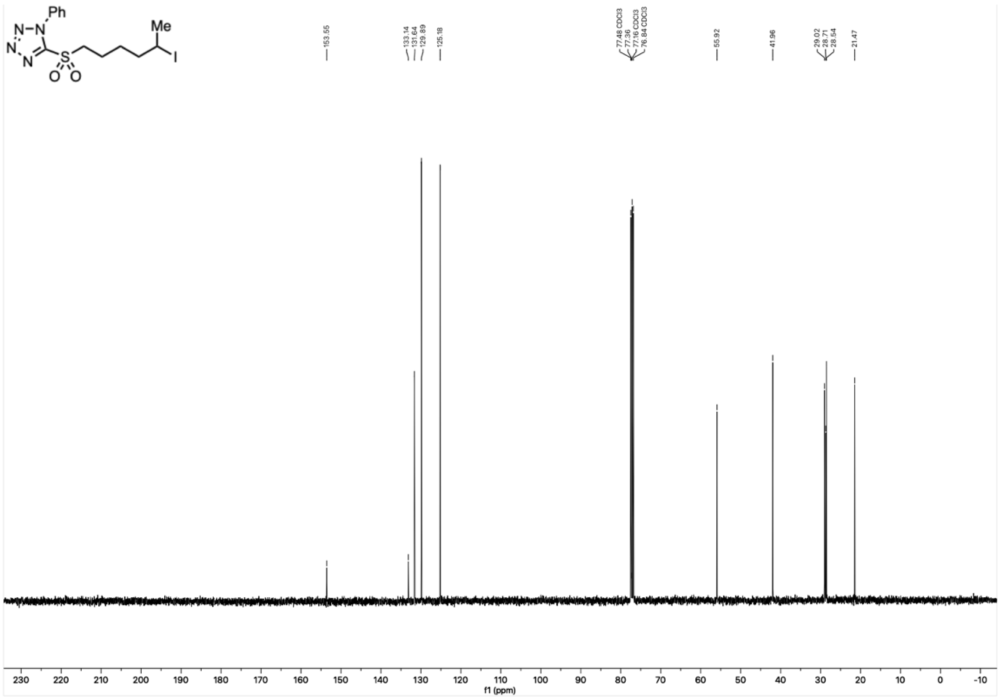

2q ^{1}H (400 MHz, $CDCl_3$)

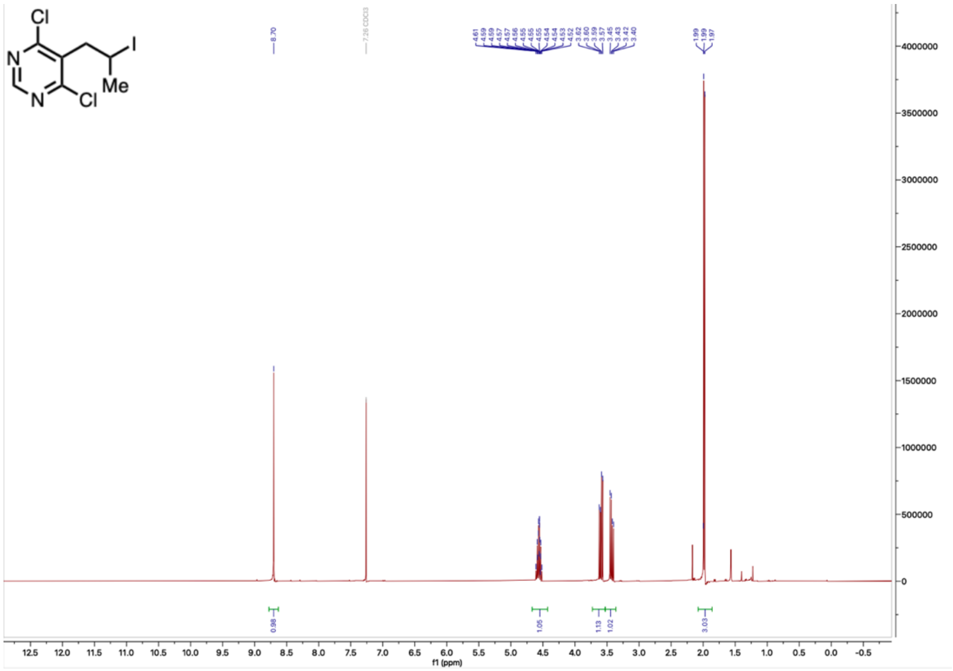

2q ^{13}C NMR (101 MHz, $CDCl_3$)

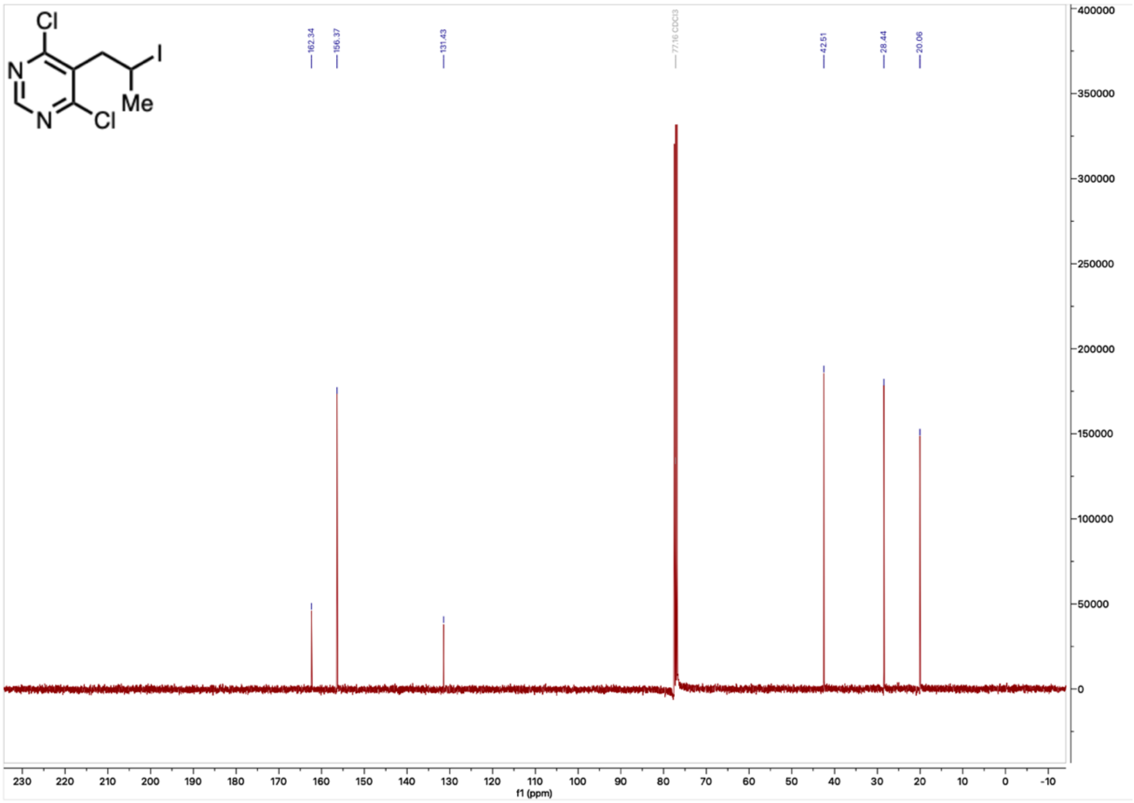

2r ^{1}H (400 MHz, $CDCl_3$)

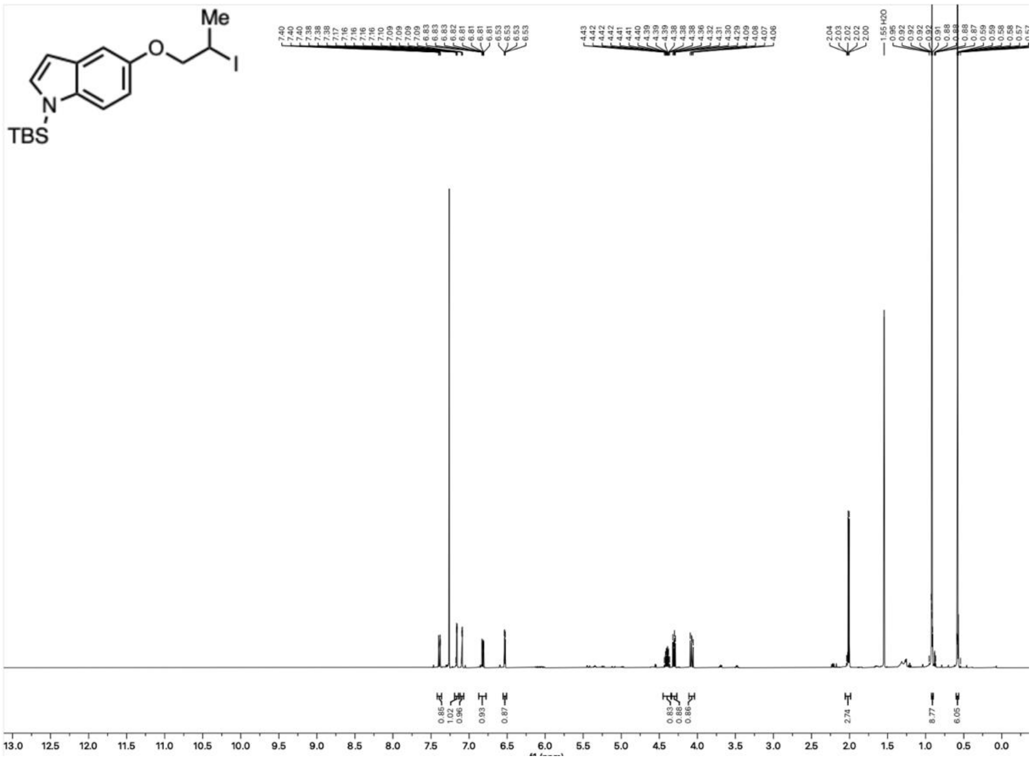

2r ^{13}C NMR (101 MHz, $CDCl_3$)

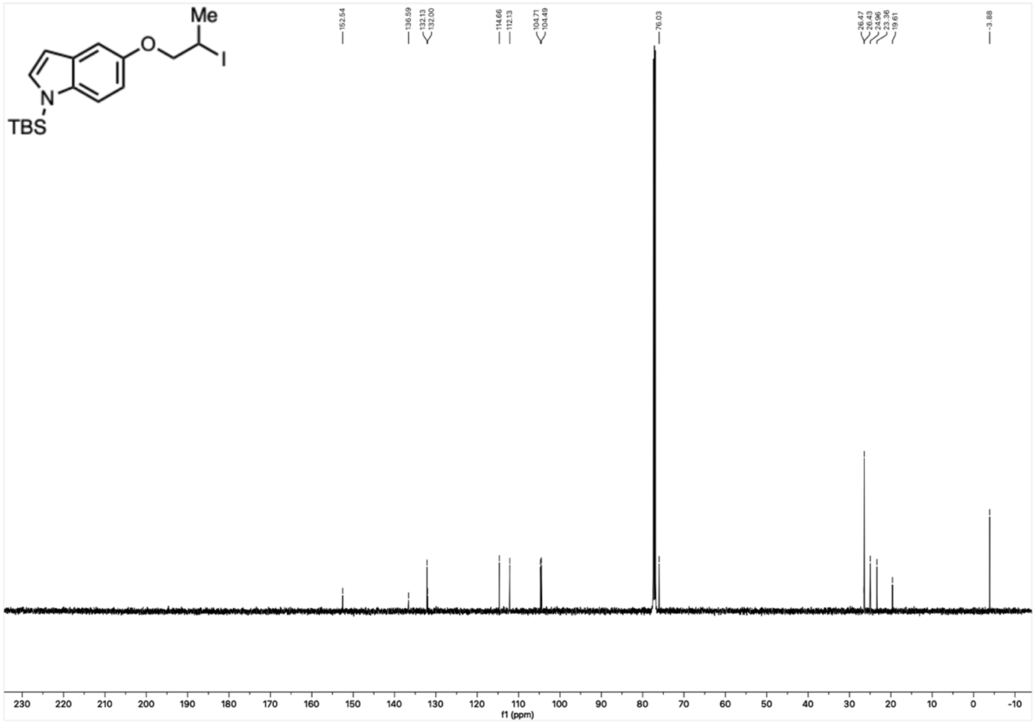

2s ^{1}H (400 MHz, $CDCl_3$)

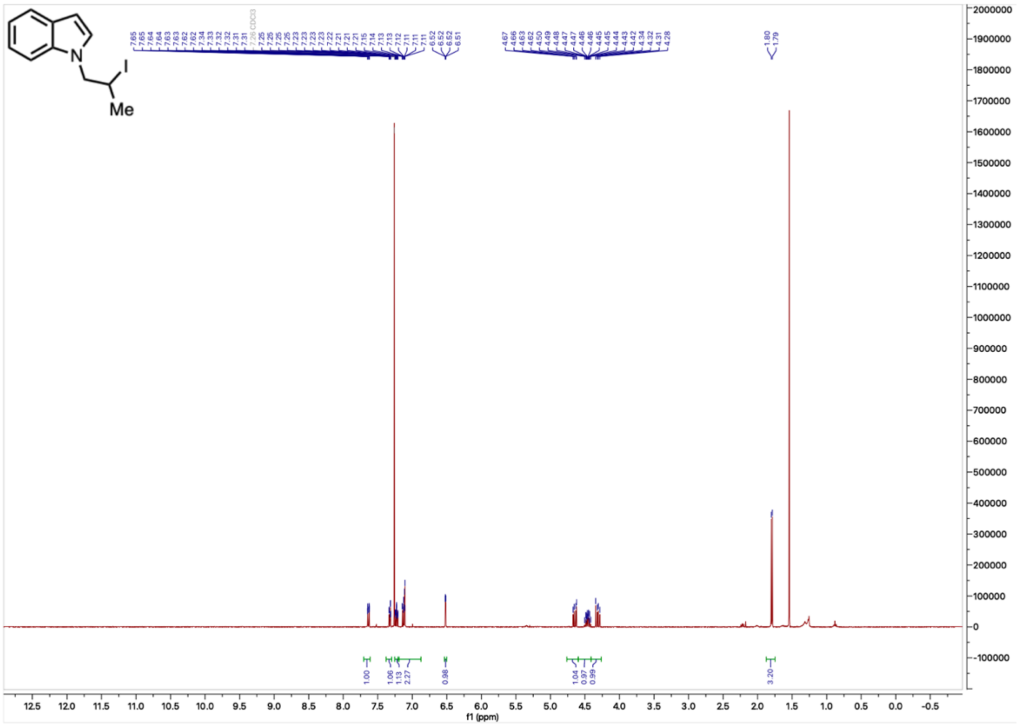

2s ^{13}C NMR (101 MHz, $CDCl_3$)

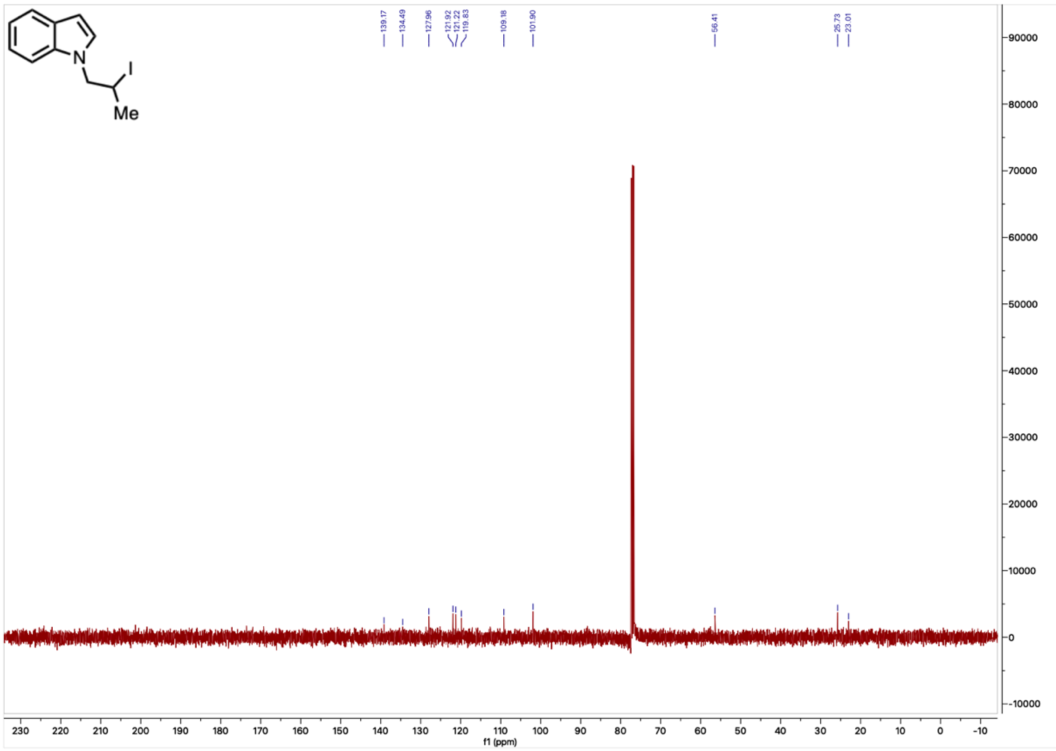

2t ^{1}H (400 MHz, $CDCl_3$)

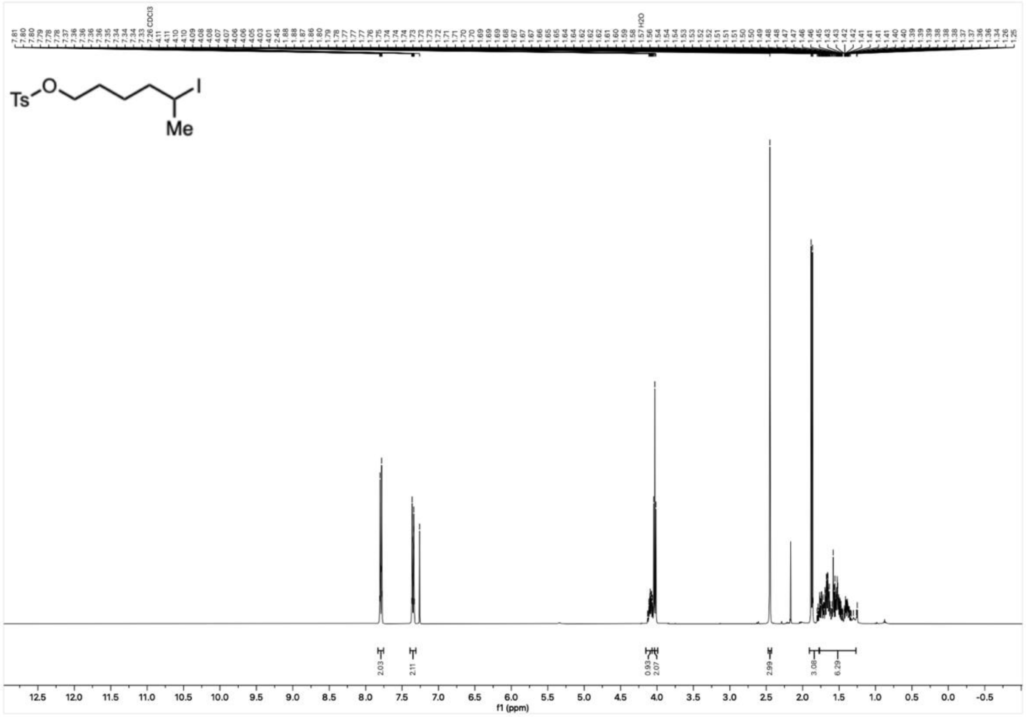

2t ^{13}C NMR (101 MHz, $CDCl_3$)

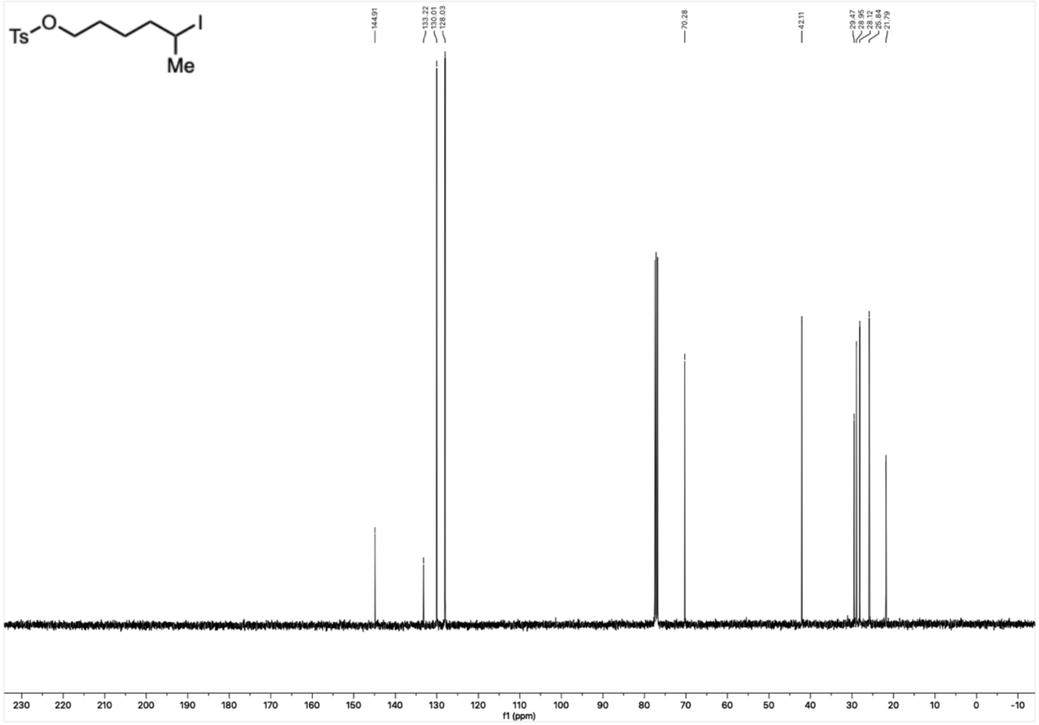

2u ^{1}H (400 MHz, $CDCl_3$)

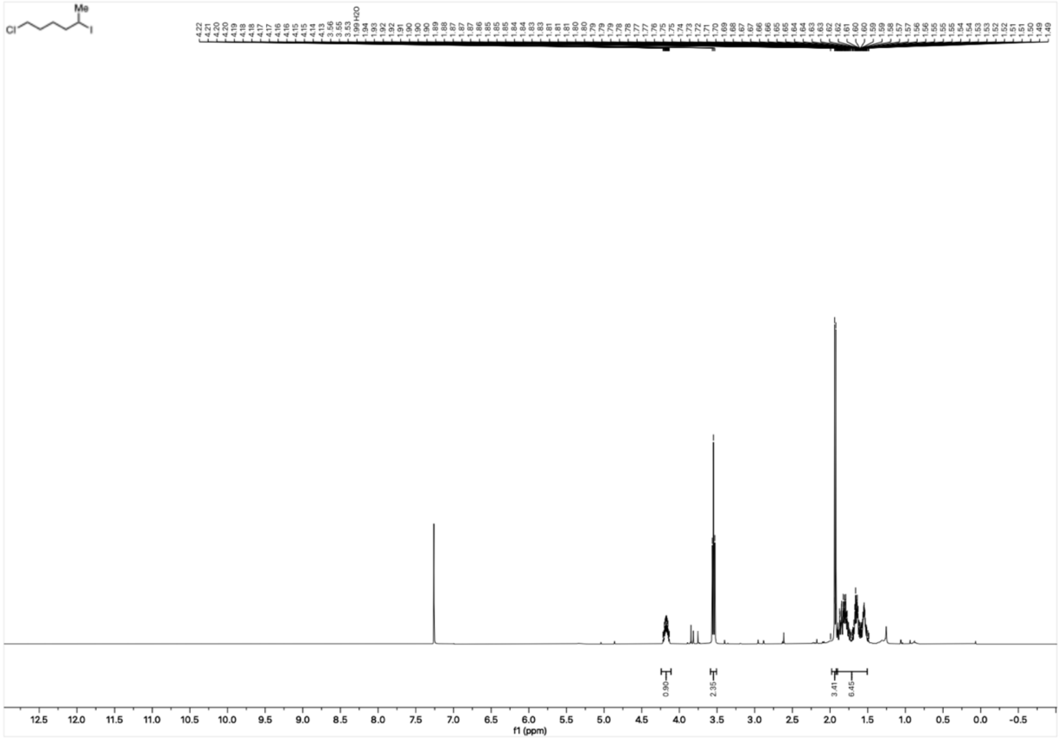

2u ^{13}C NMR (101 MHz, $CDCl_3$)

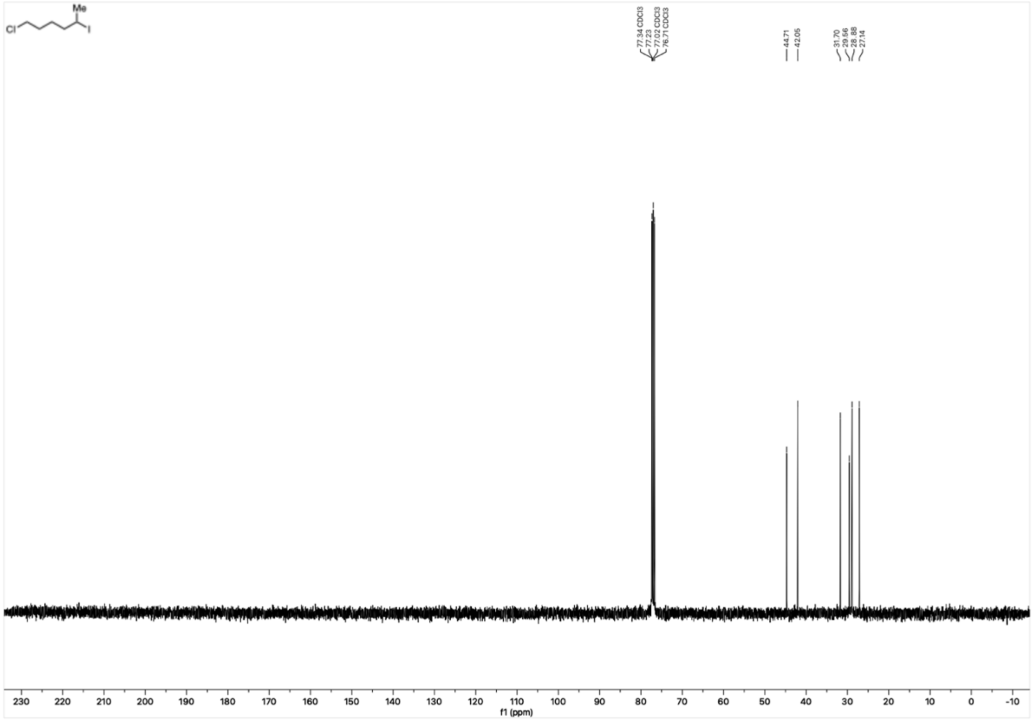

2v ^{1}H (400 MHz, $CDCl_3$)

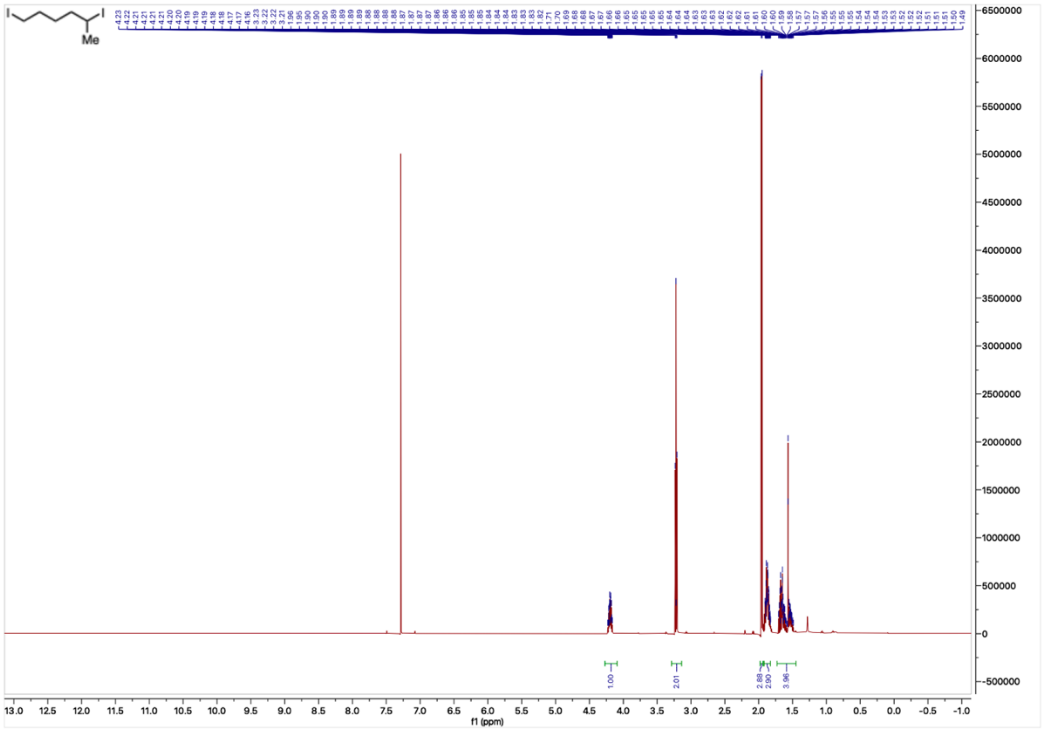

2v ^{13}C NMR (101 MHz, $CDCl_3$)

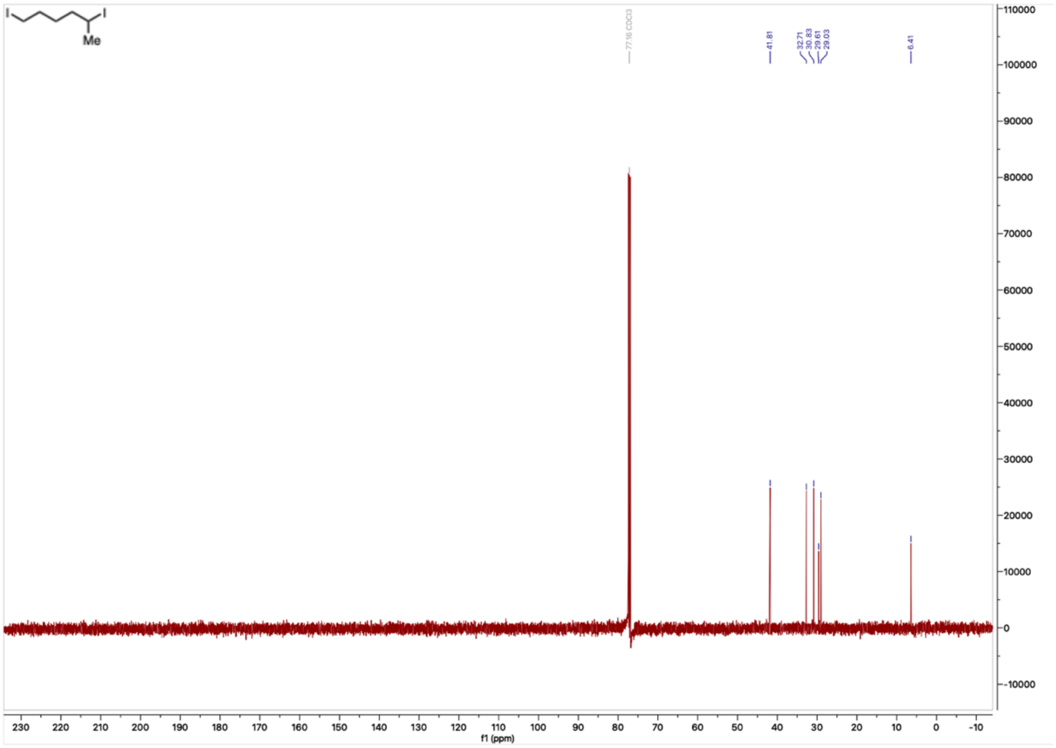

2w ^{1}H (400 MHz, $CDCl_3$)

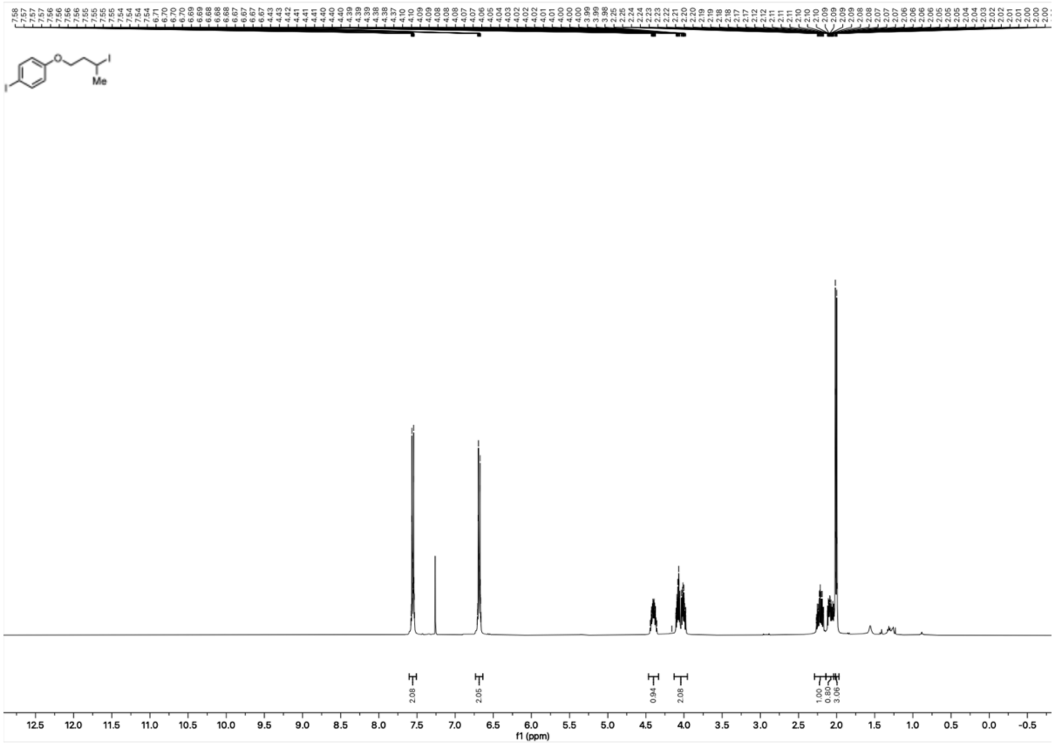

2w ^{13}C NMR (101 MHz, $CDCl_3$)

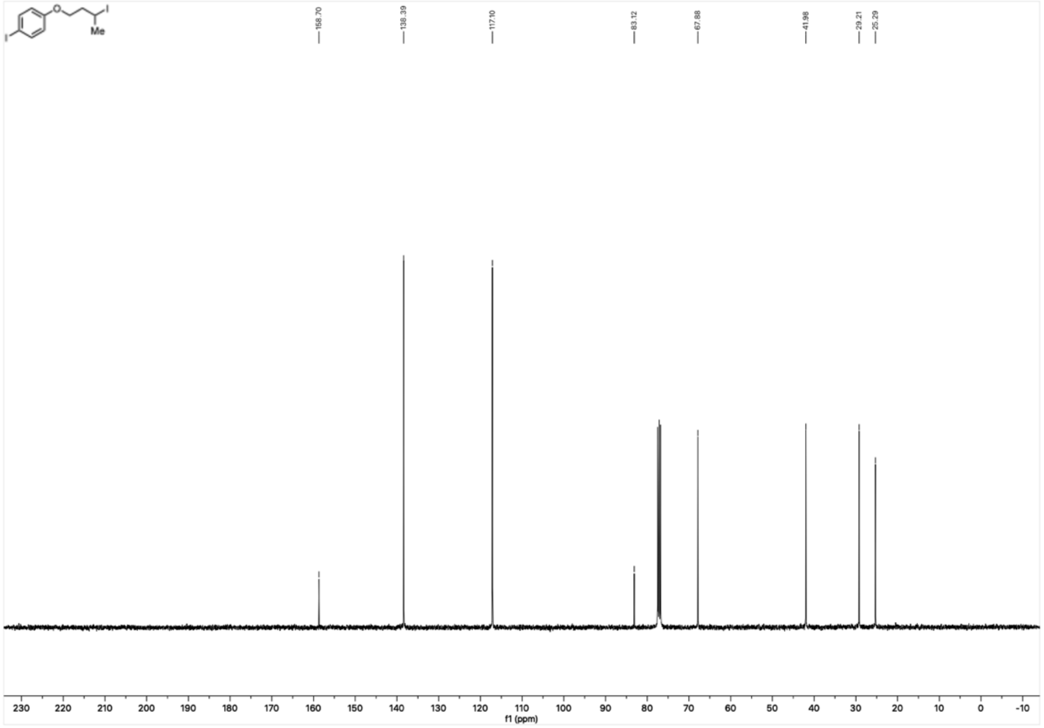

2x ^{1}H (400 MHz, $CDCl_3$)

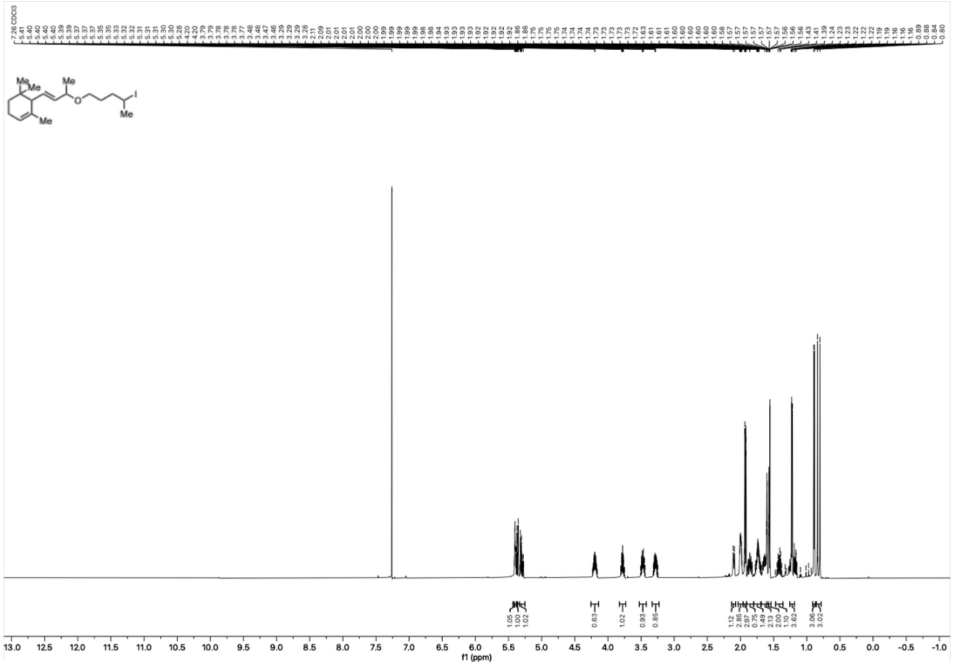

2x ^{13}C NMR (101 MHz, $CDCl_3$)

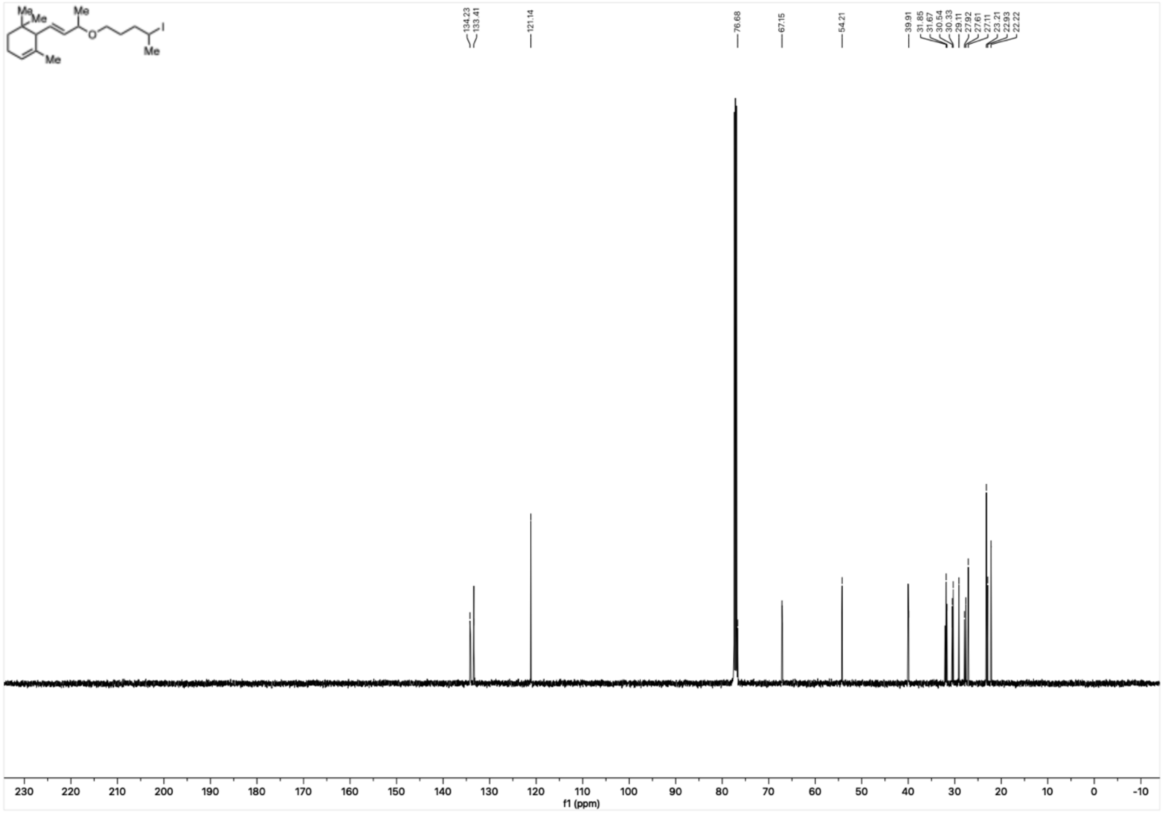

2y ^{1}H (600 MHz, $CDCl_3$)

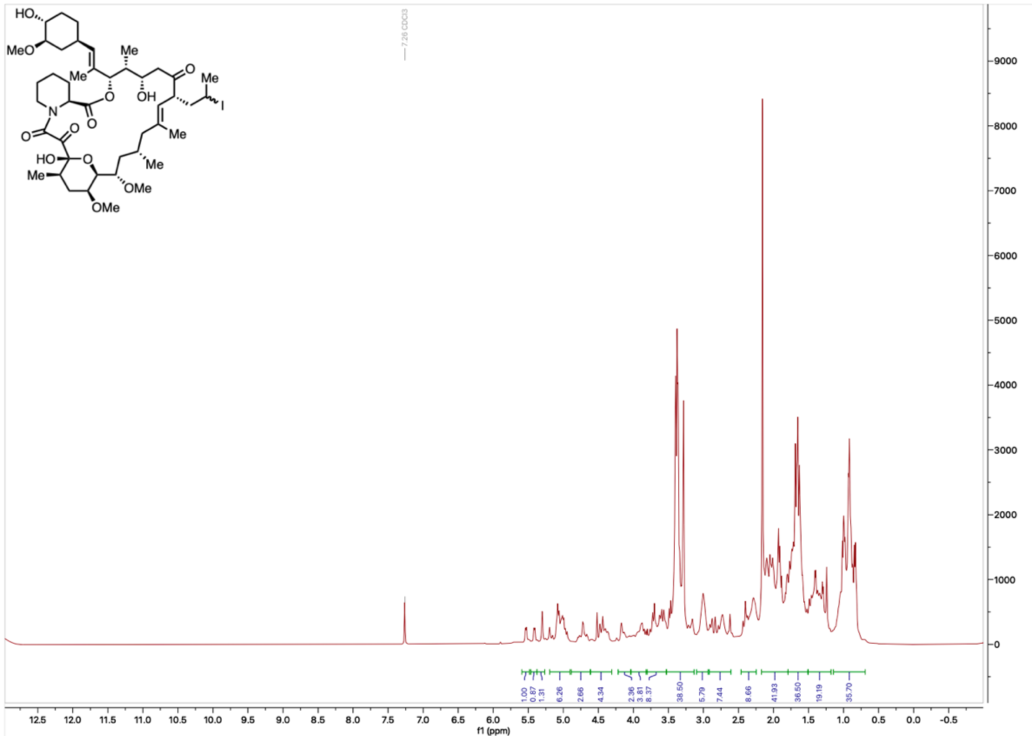

2y ^{13}C NMR (151 MHz, $CDCl_3$)

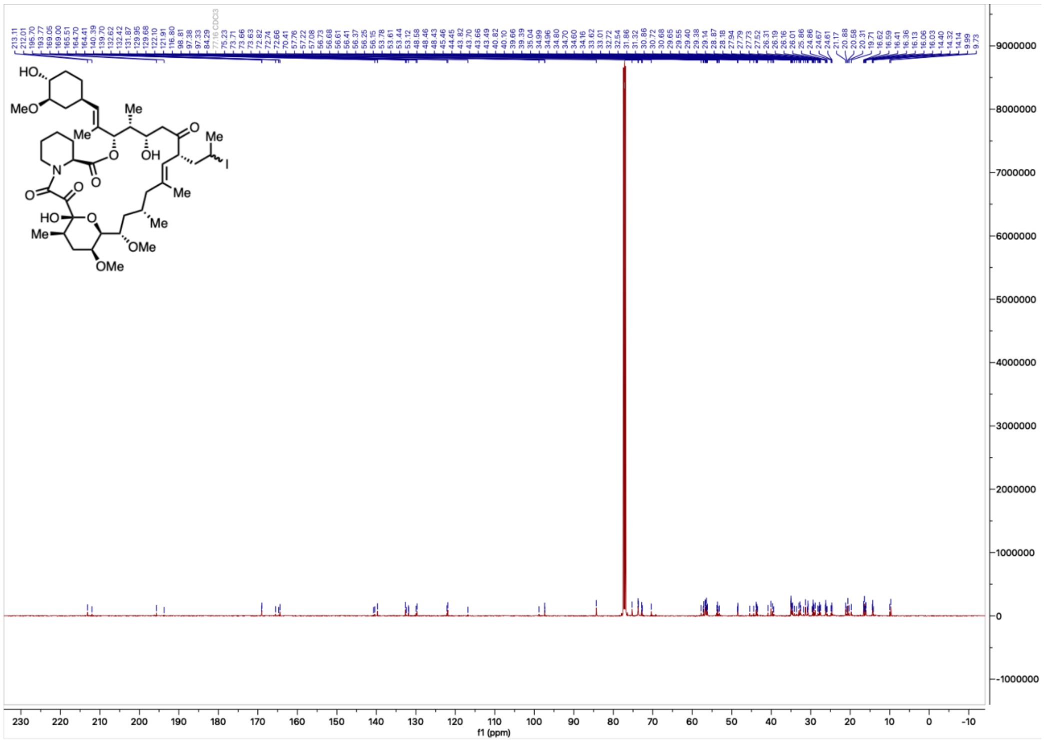

2y ^{1}H–^{13}C HSQC ($CDCl_3$)

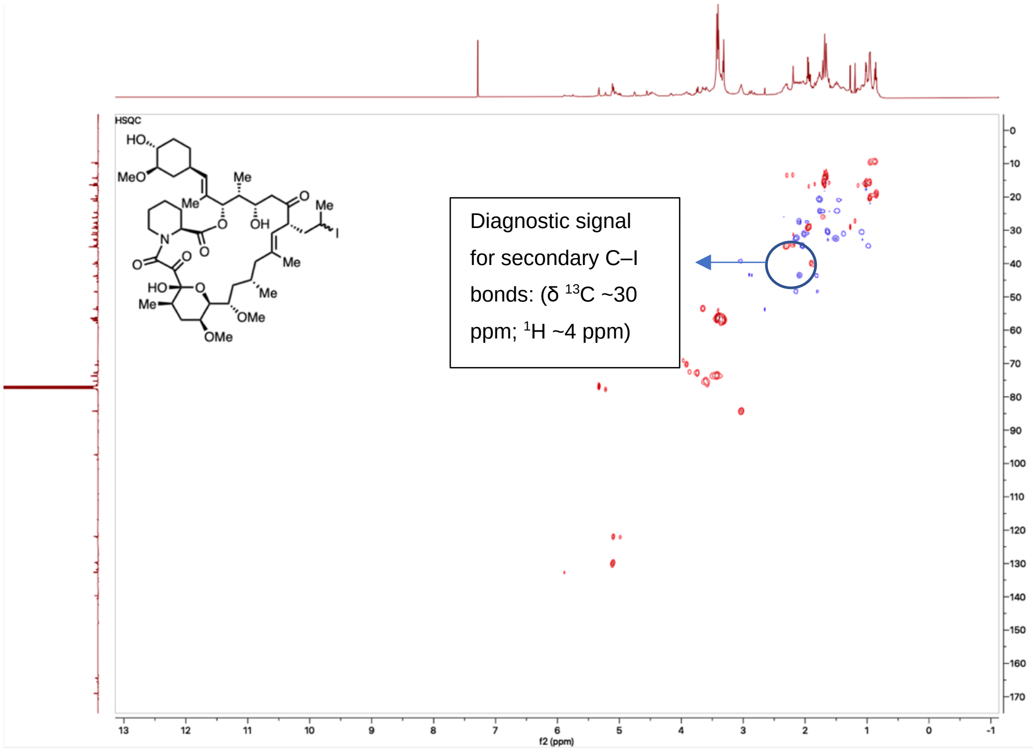

2y ^{1}H–^{13}C ($CDCl_3$)

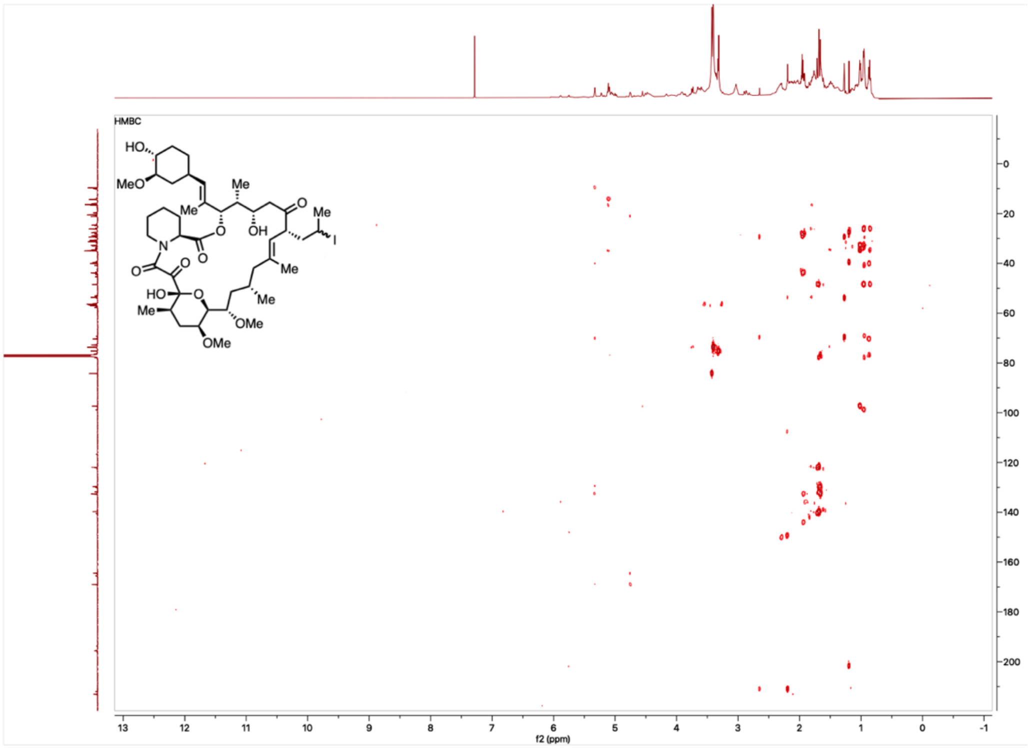

2z ^{1}H (400 MHz, $CDCl_3$)

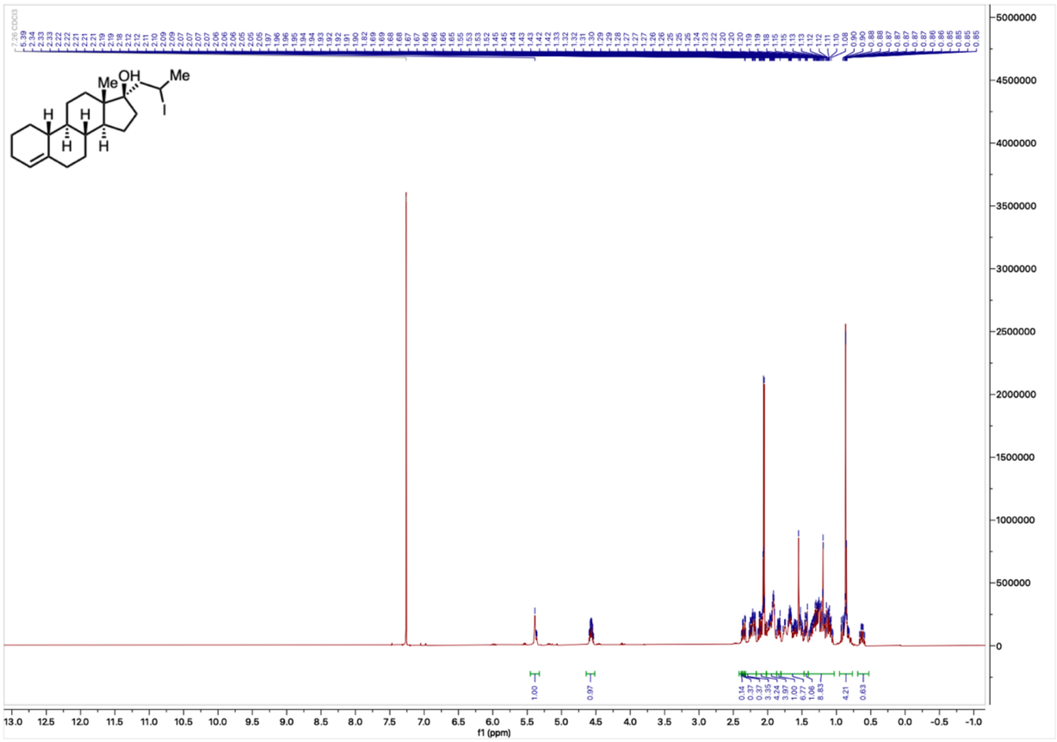

2z ^{13}C NMR (101 MHz, $CDCl_3$)

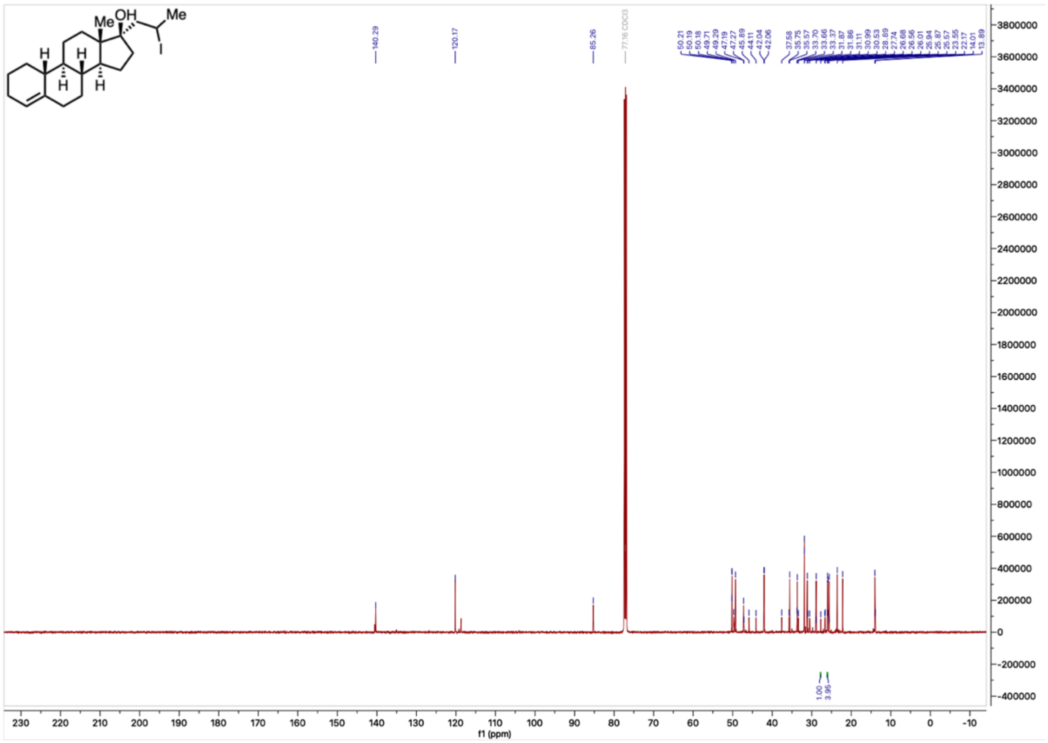

2z ^{1}H–^{13}C HSQC ($CDCl_3$)

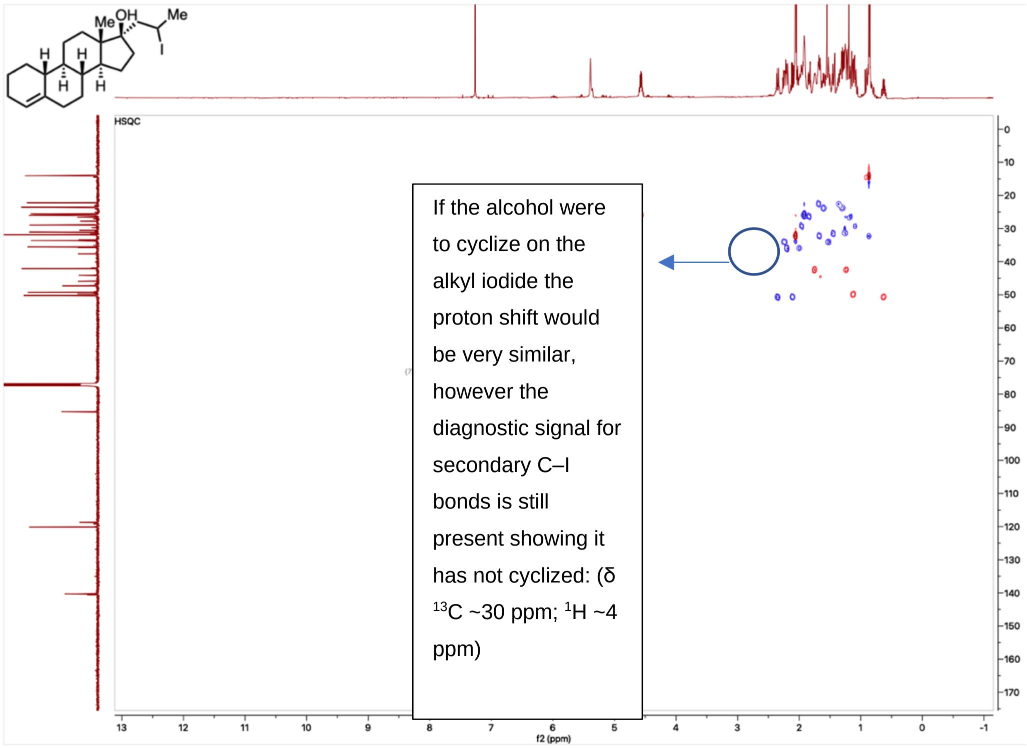

2z ^{1}H–^{13}C NOESY ($CDCl_3$)

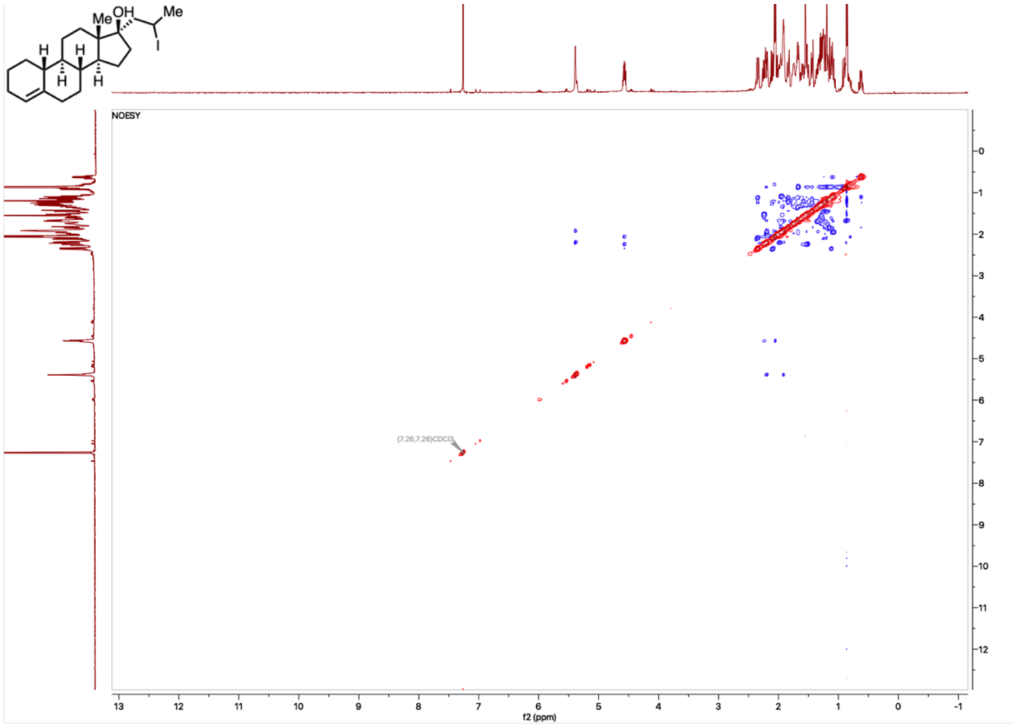

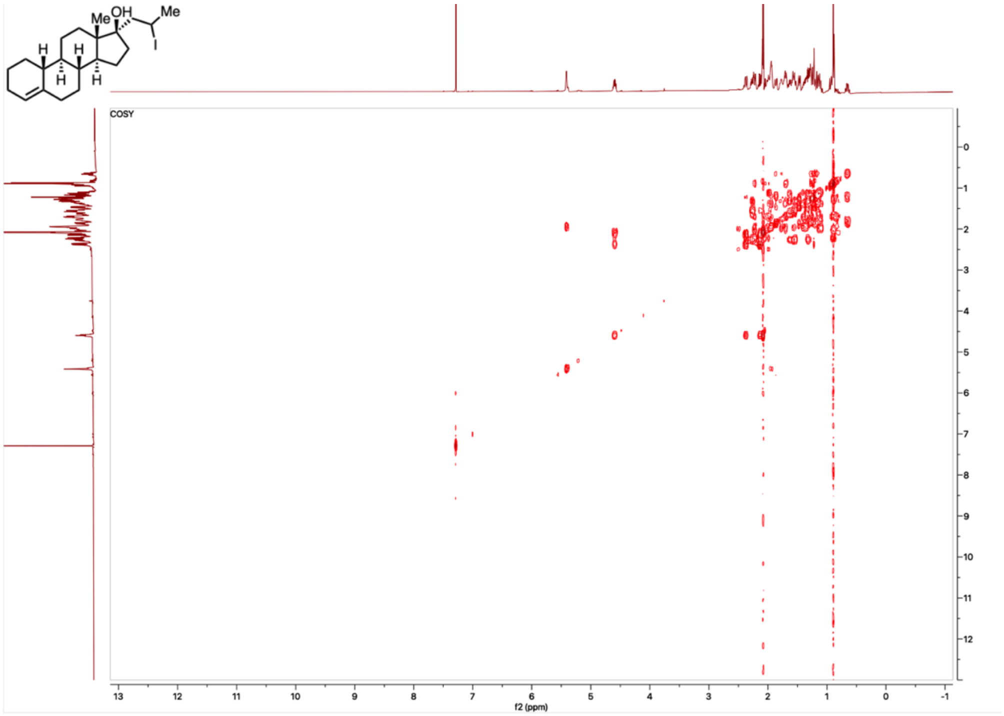

2z ^{1}H–^{13}C HMBC ($CDCl_3$)

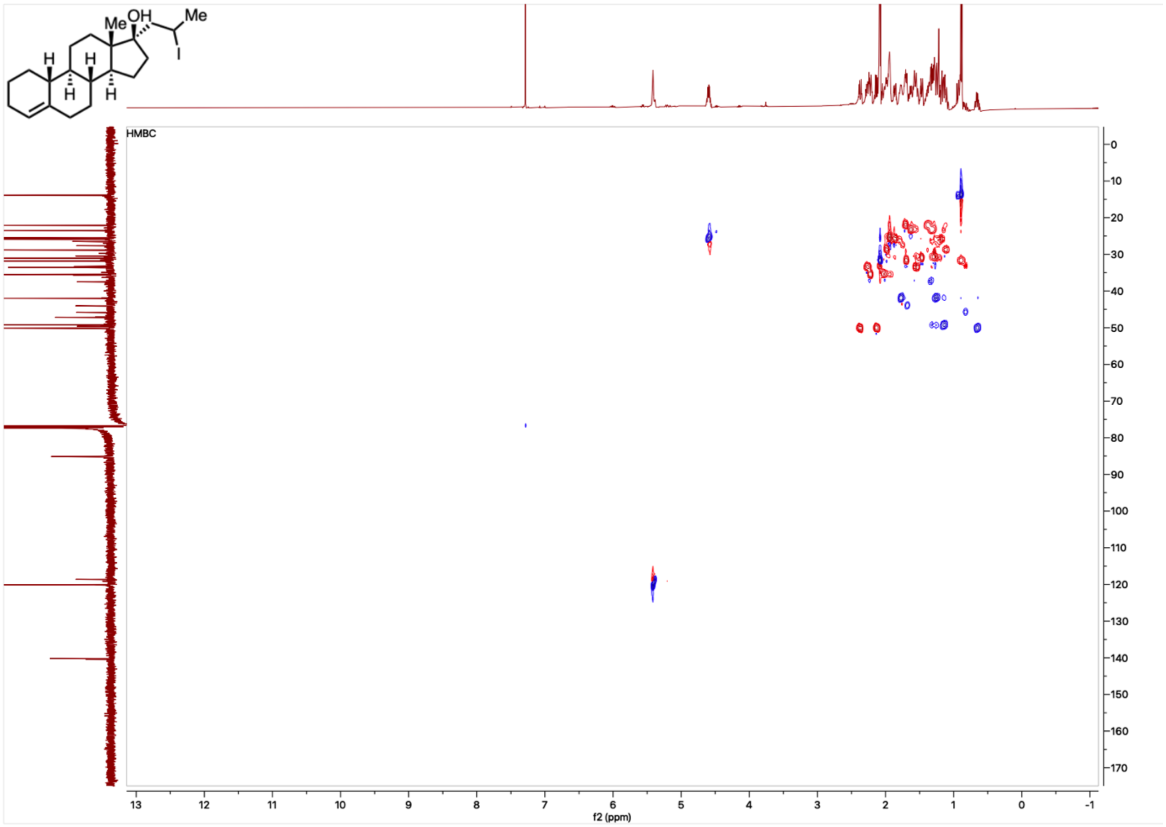

2aa (minor) ^{1}H (400 MHz, CD_3COD)

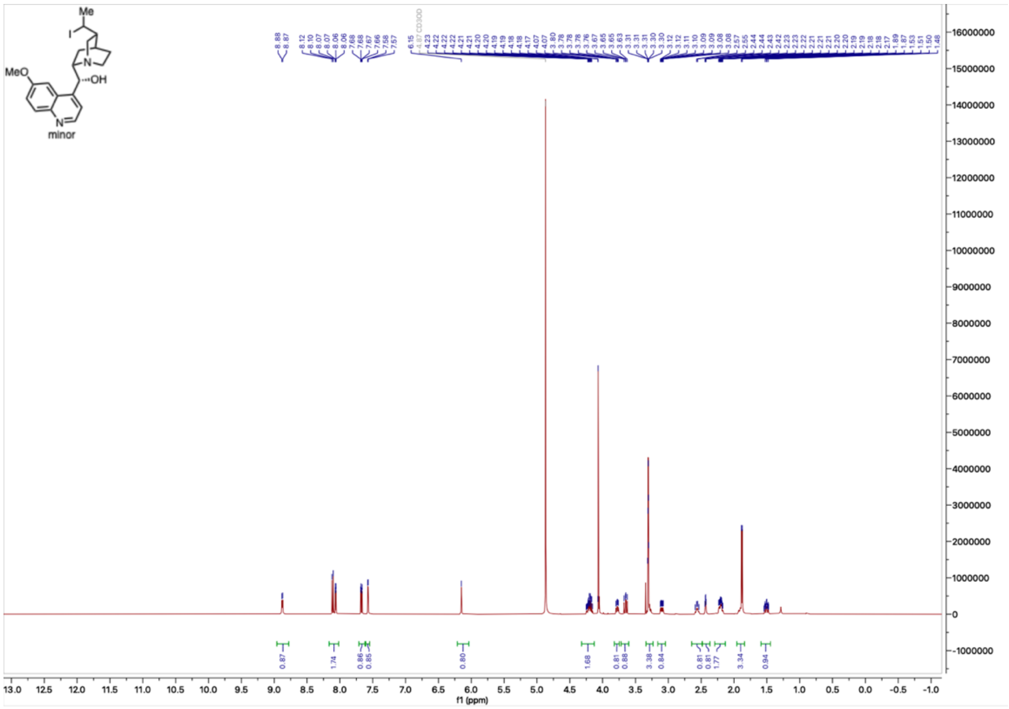

2aa (minor) ^{13}C NMR (101 MHz, CD_3COD)

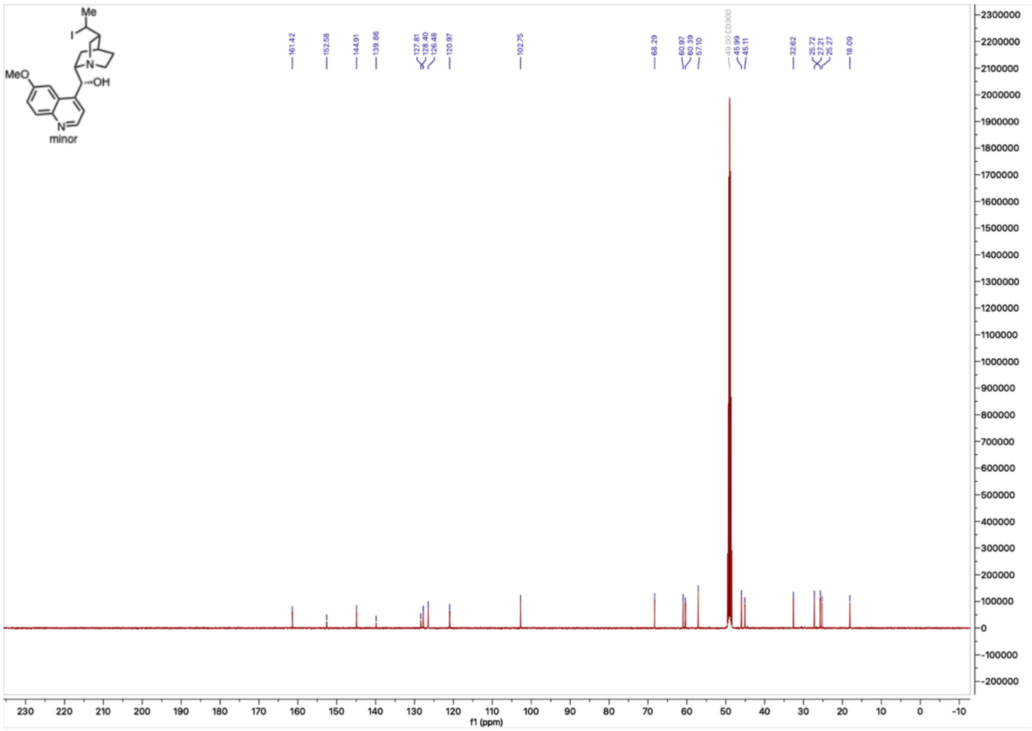

2aa (minor) ^{1}H–^{13}C HSQC (CD_3COD)

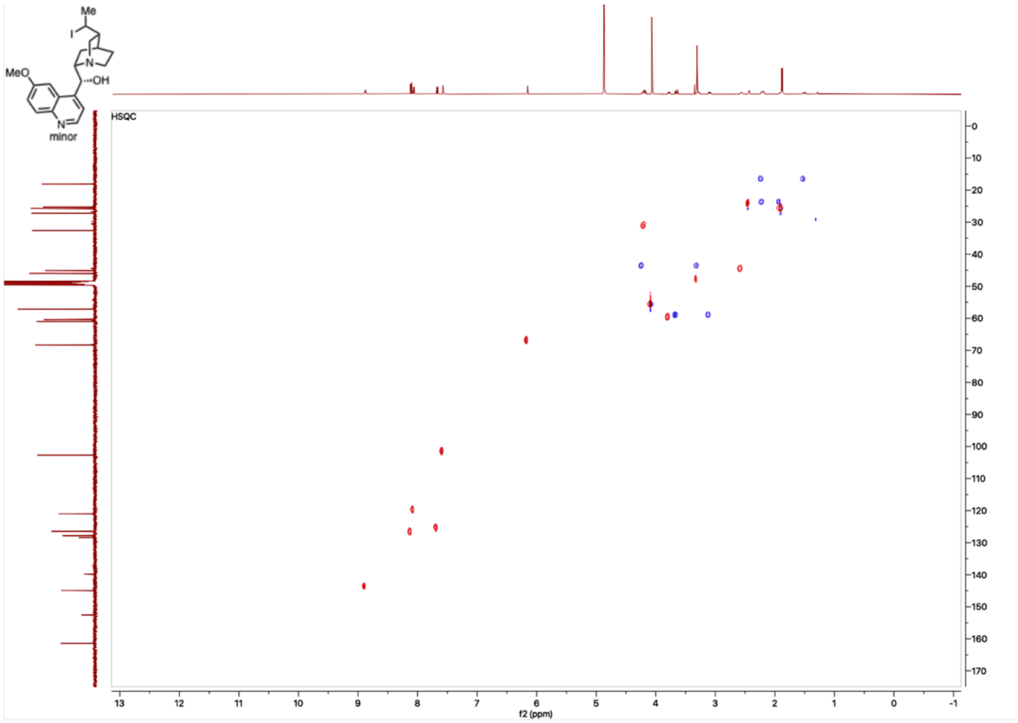

2e ^{1}H–^{1}H NOESY (CD_3COD)

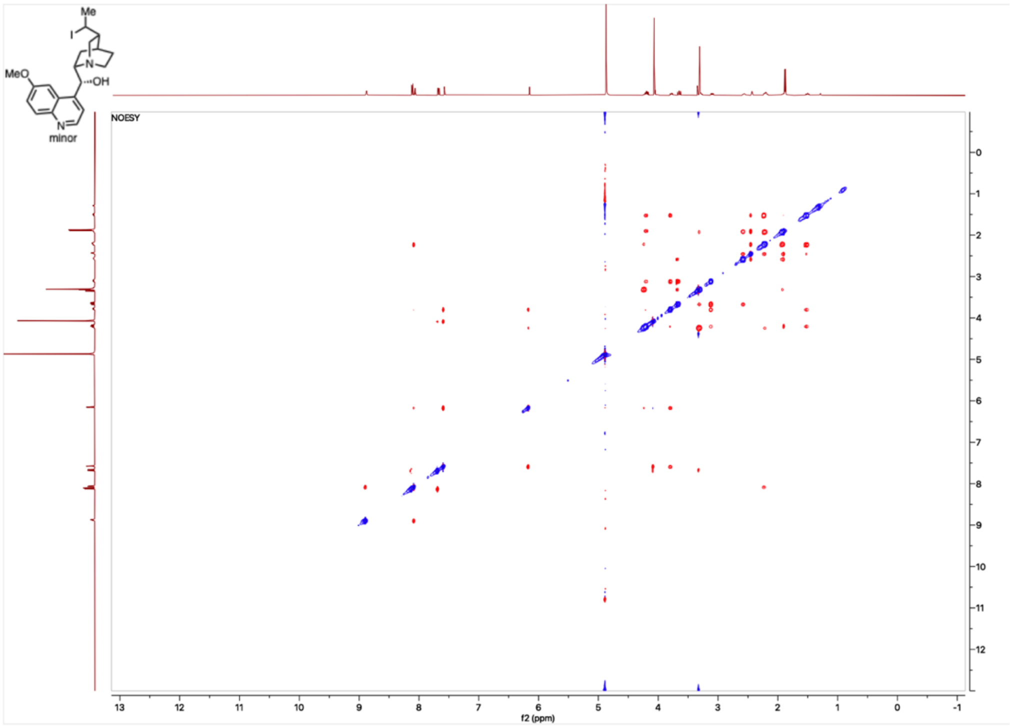

2aa (minor) ^{1}H–^{13}C HMBC (400 MHz, CD_3COD)

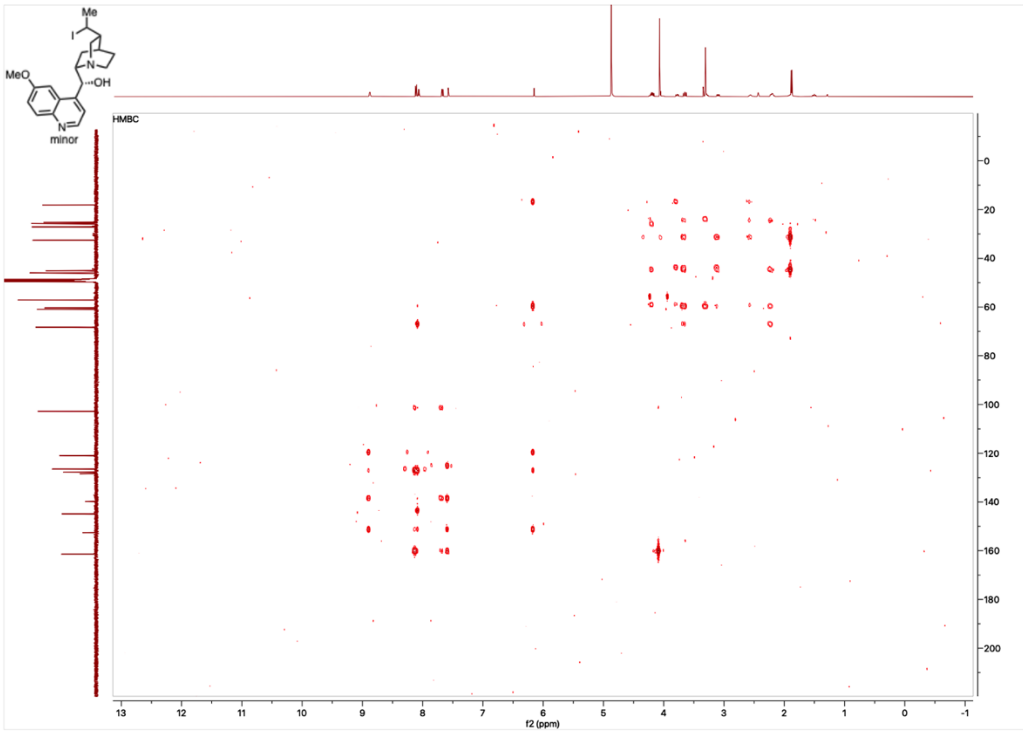

2aa (major) ^{1}H (400 MHz, CD_3COD)

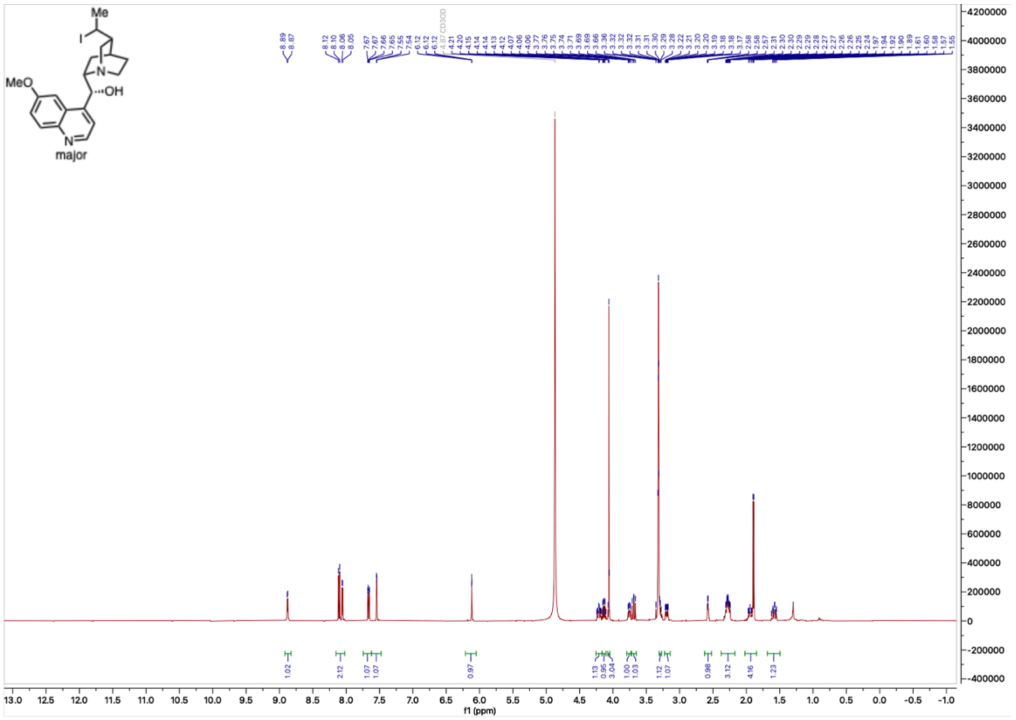

2aa (major) ^{13}C NMR (101 MHz, CD_3COD)

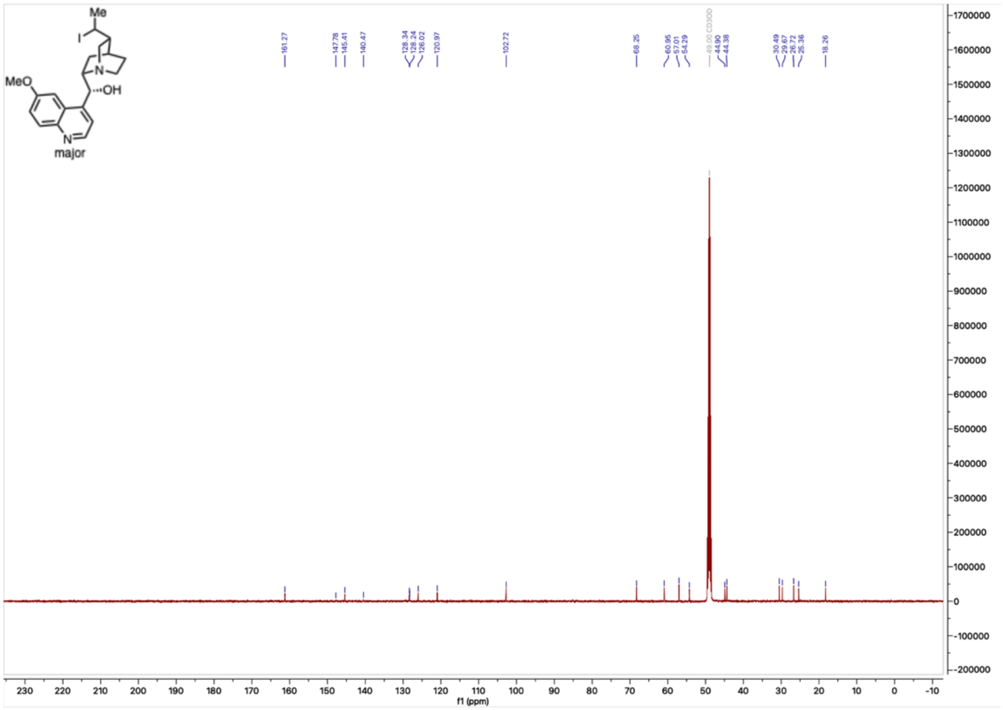

2aa (major) ^{1}H–^{13}C HSQC (CD_3COD)

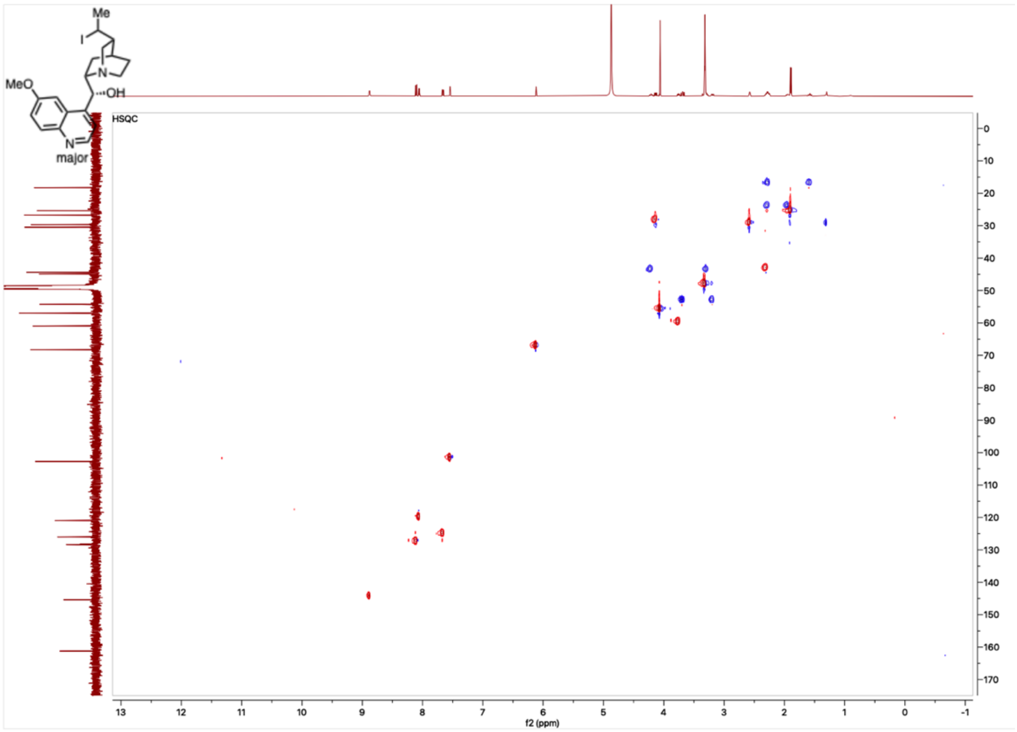

2aa (major) ^{1}H NOESY (CD_3COD)

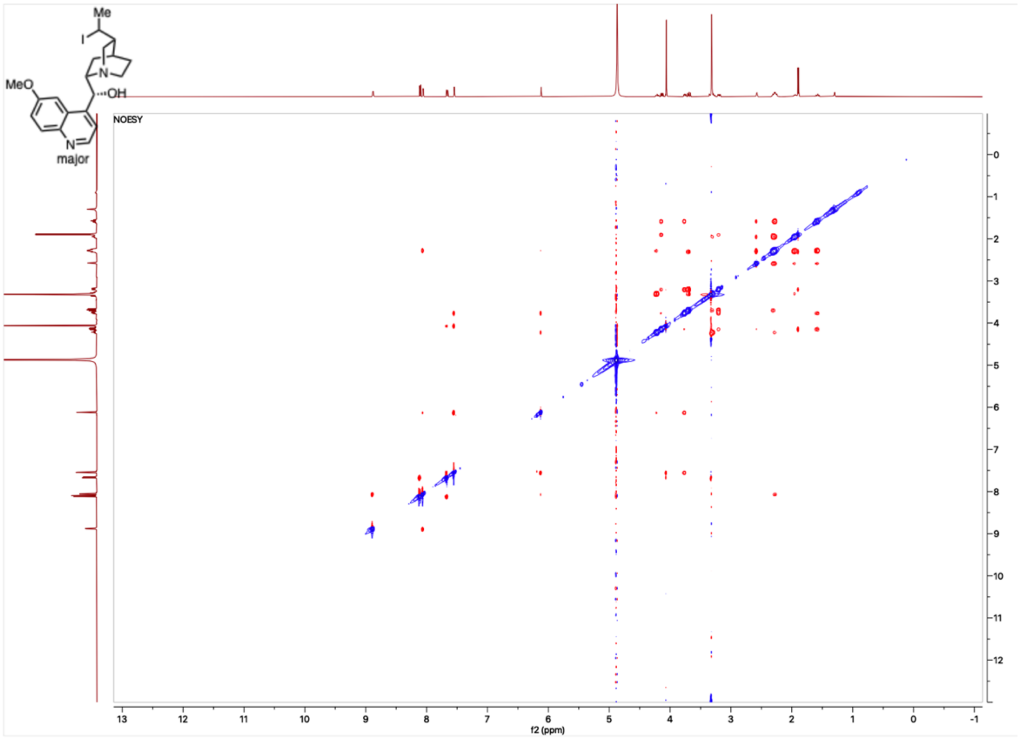

2aa (major) ^{1}H (CD_3COD)

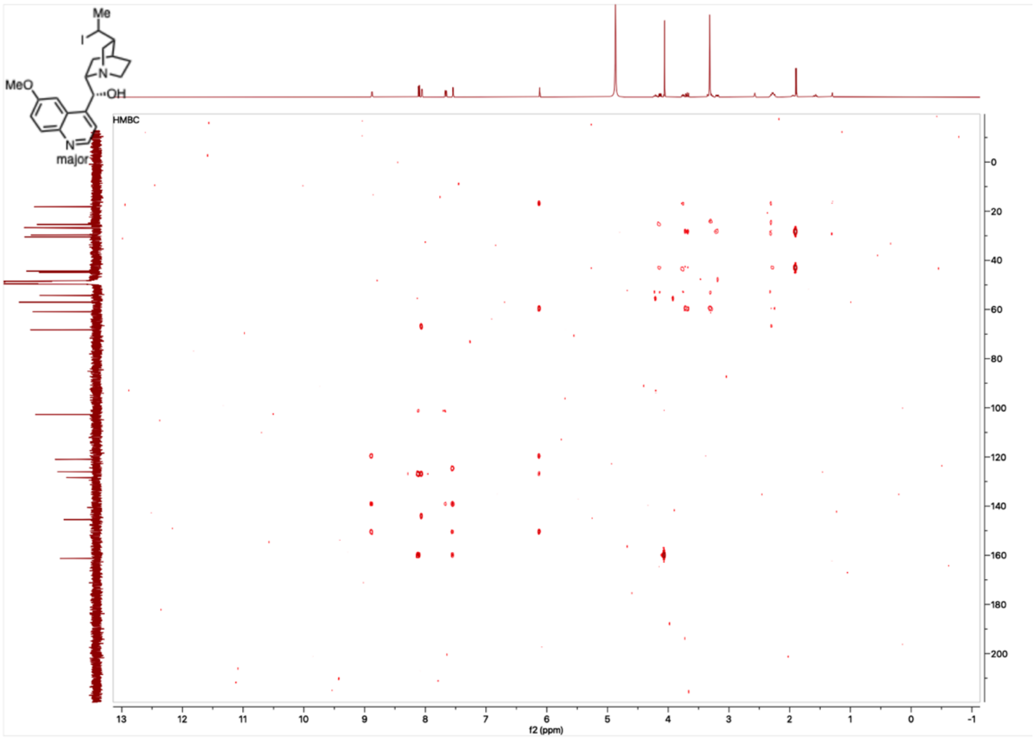

2ab ^{1}H (400 MHz, $CDCl_3$)

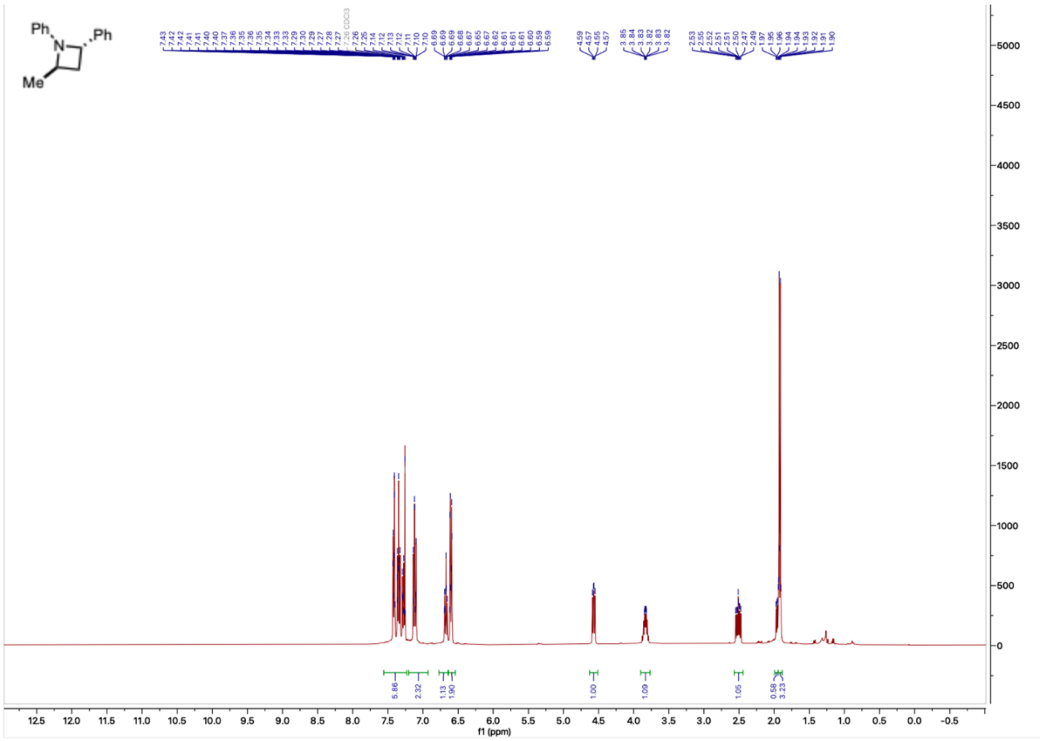

2ab ^{13}C (101 MHz, $CDCl_3$)

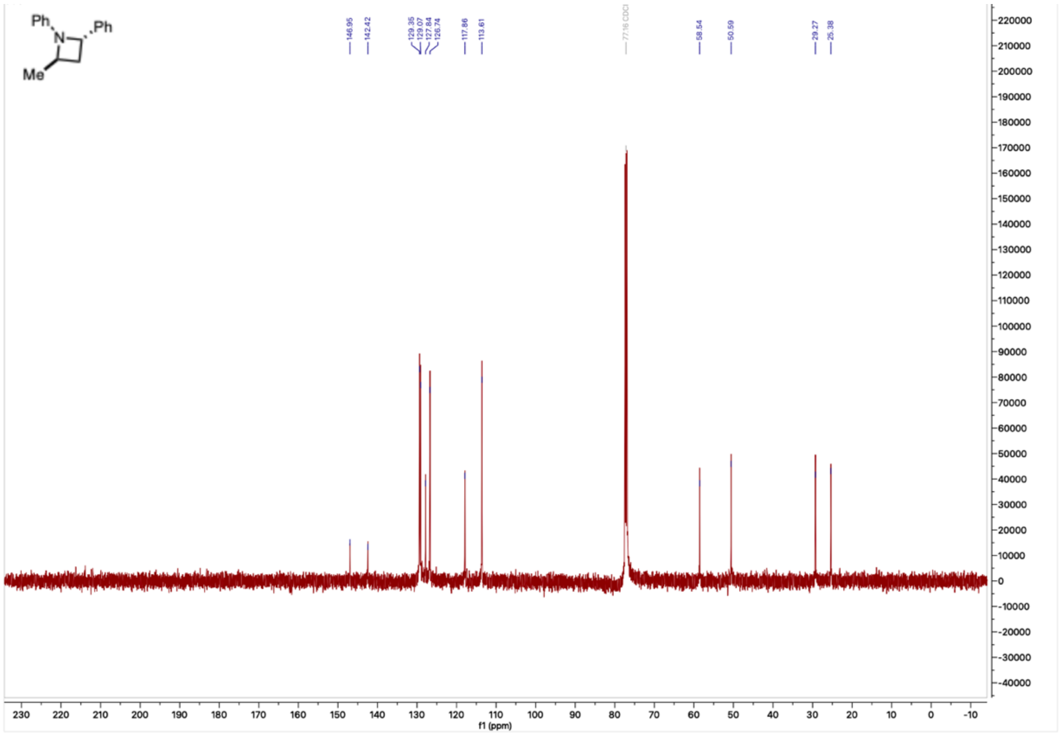

2ab ^{1}H NOESY ($CDCl_3$)

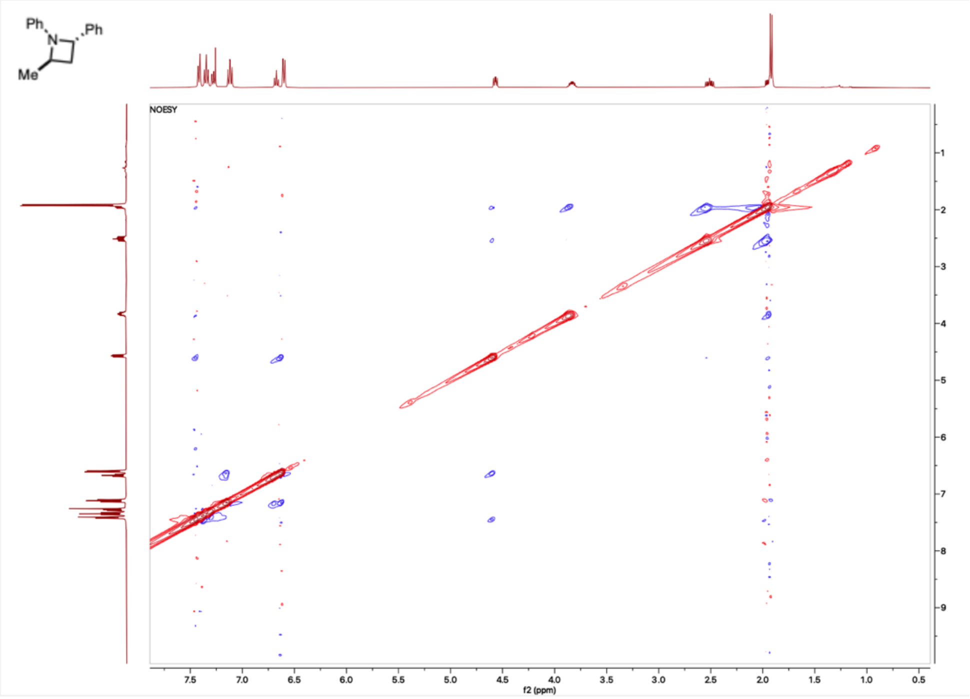

2ac ^{1}H (400 MHz, $CDCl_3$)

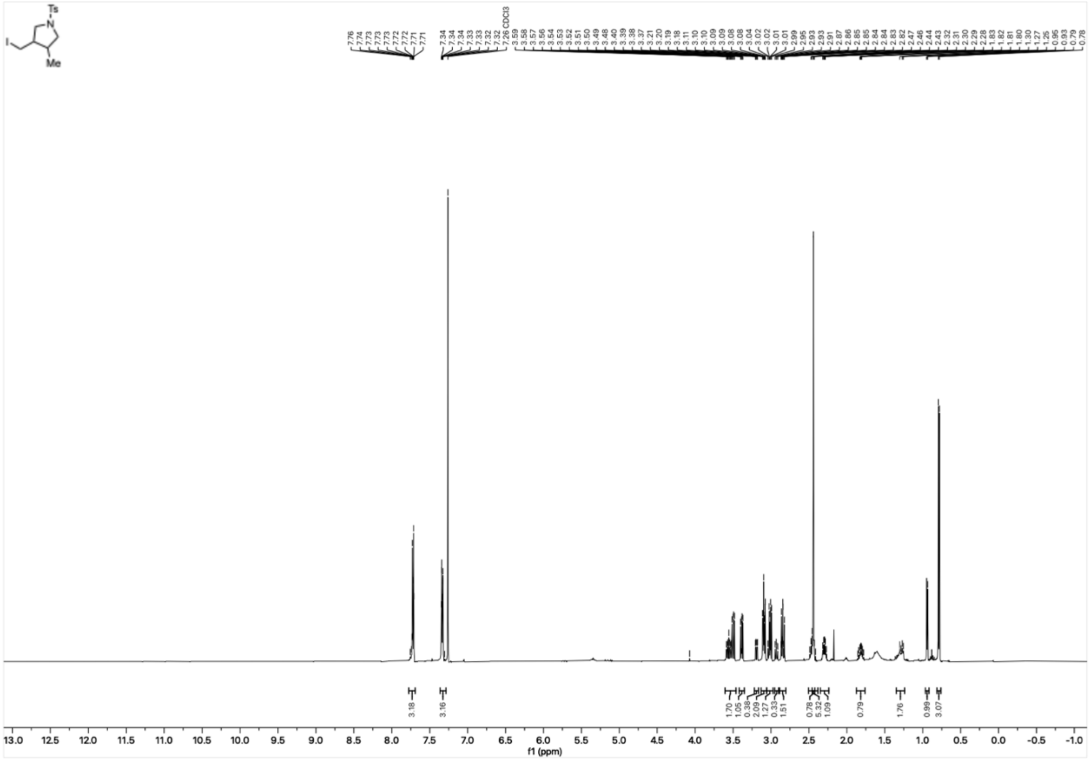

2ac ^{13}C (101 MHz, $CDCl_3$)

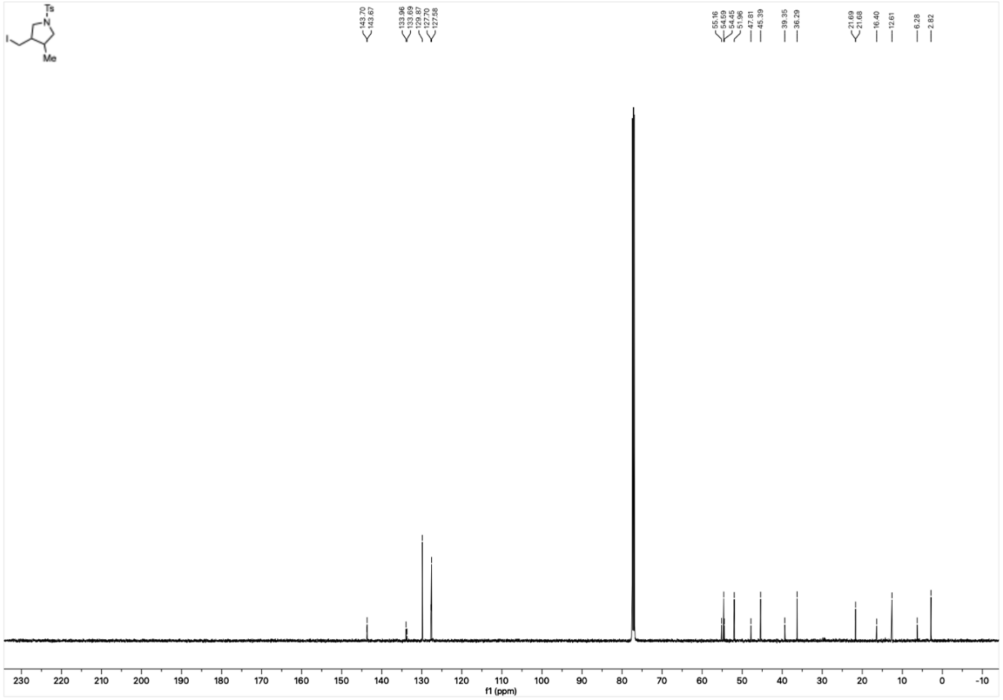

2ac ^{1}H NOESY ($CDCl_3$)

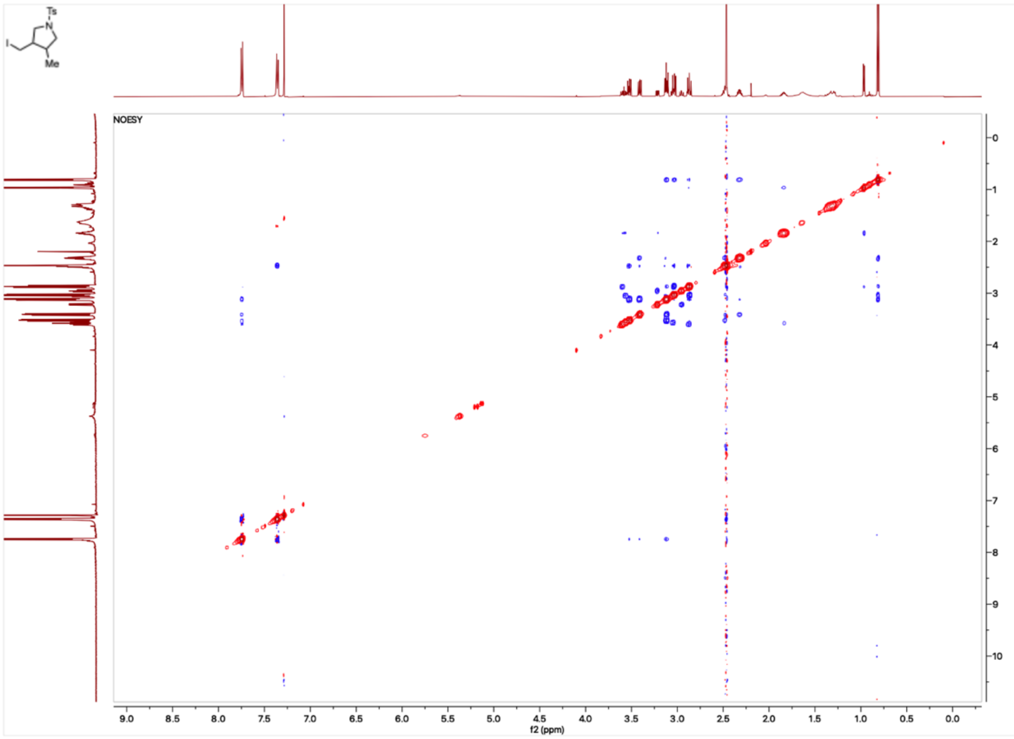

2ad’ ^{1}H (400 MHz, $CDCl_3$)

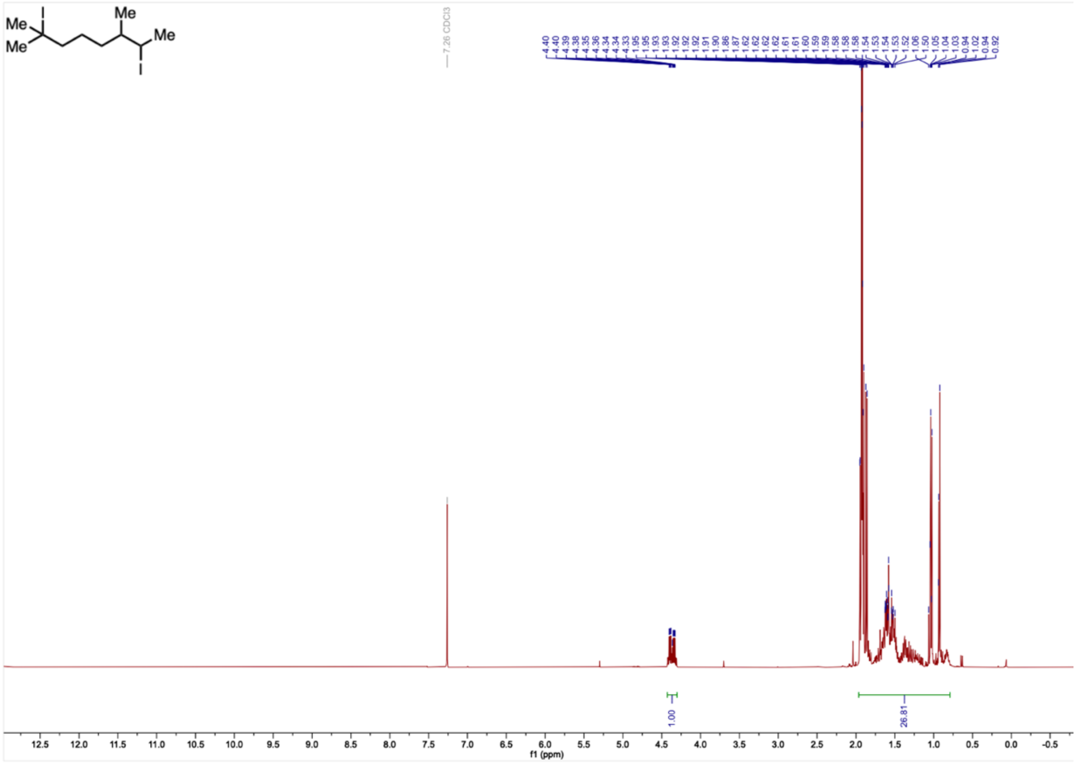

2ad ^{1}H (400 MHz, $CDCl_3$)

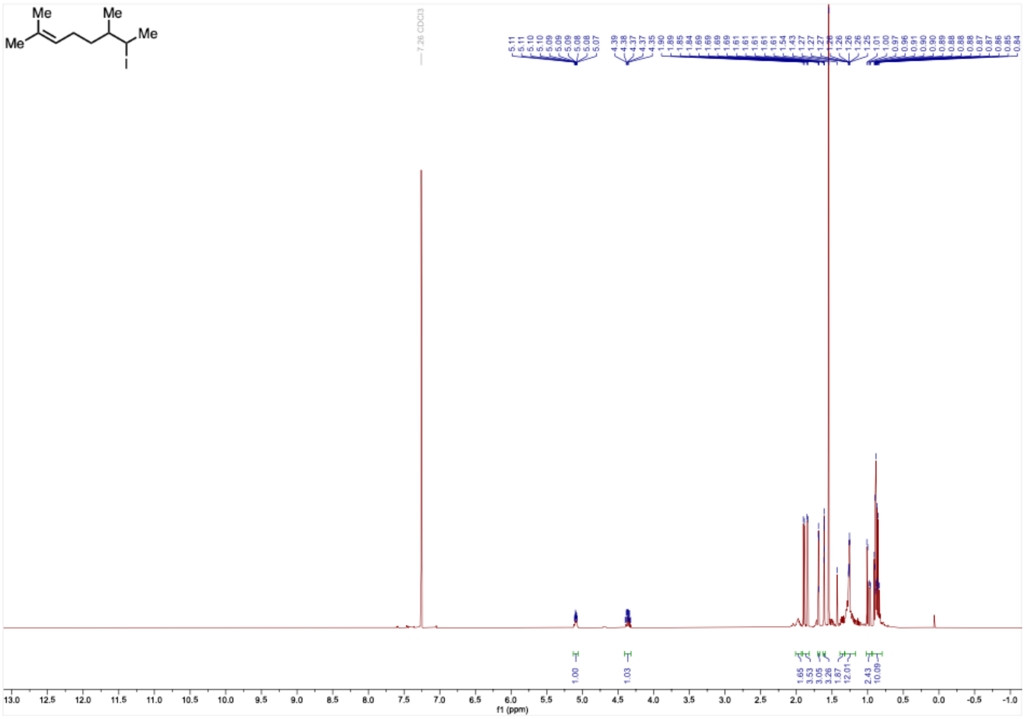

2ad ^{13}C (101 MHz, CDCl$_3$)

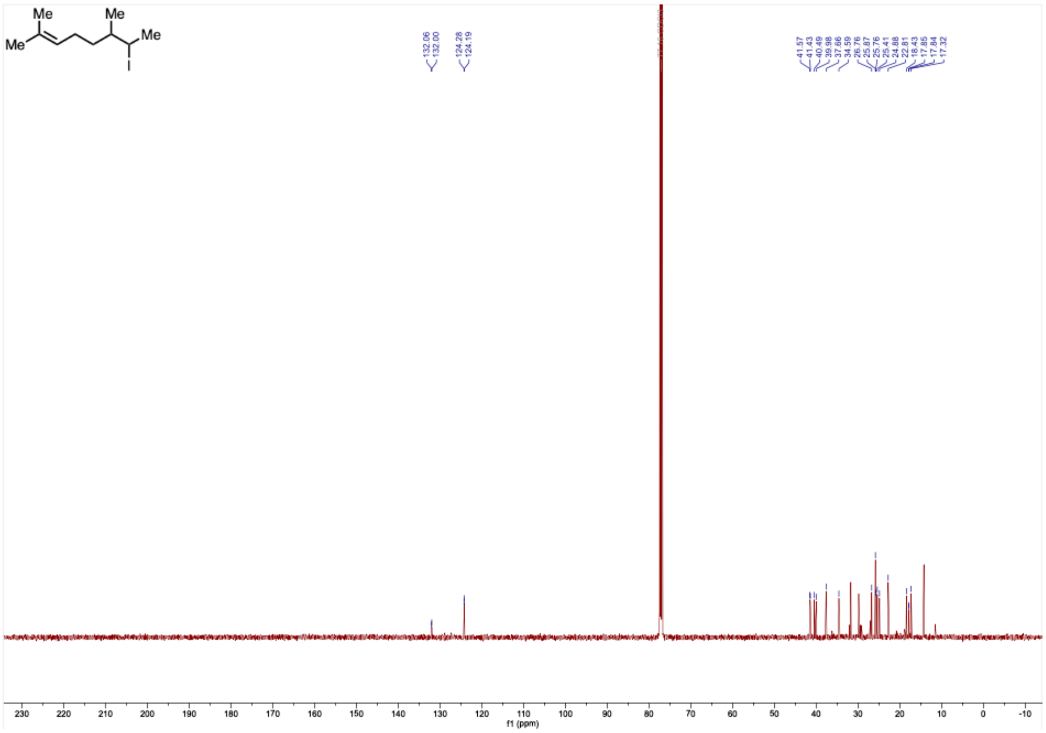

3a ^{1}H (400 MHz, CDCl$_3$)

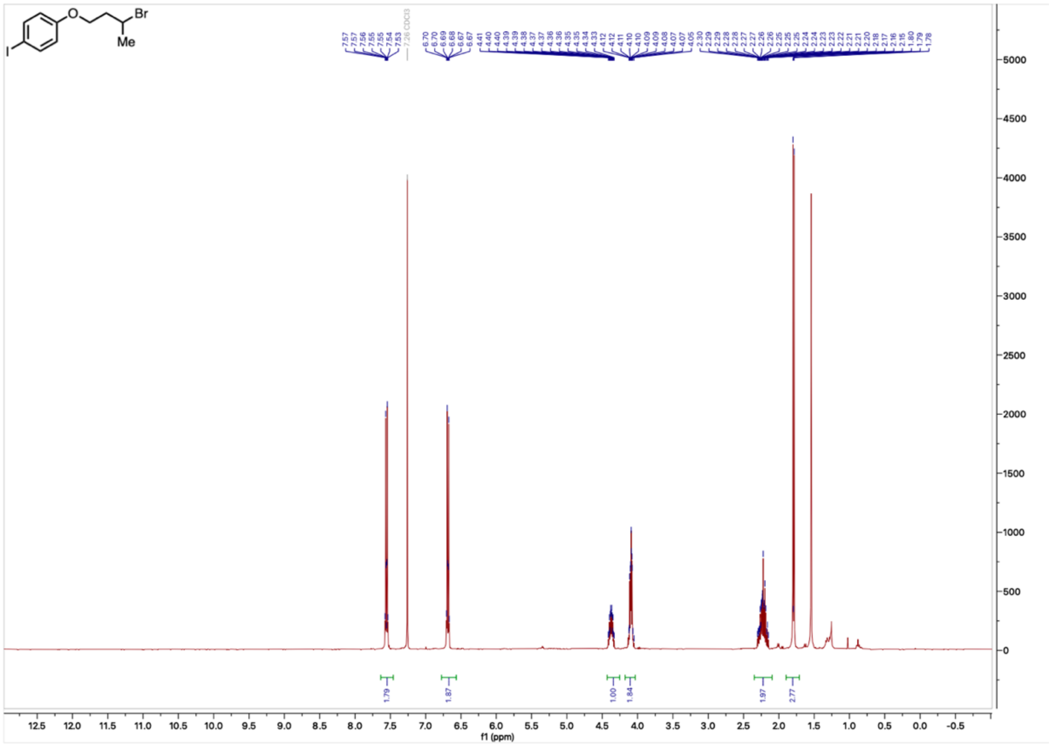

3a ^{13}C (101 MHz, $CDCl_3$)

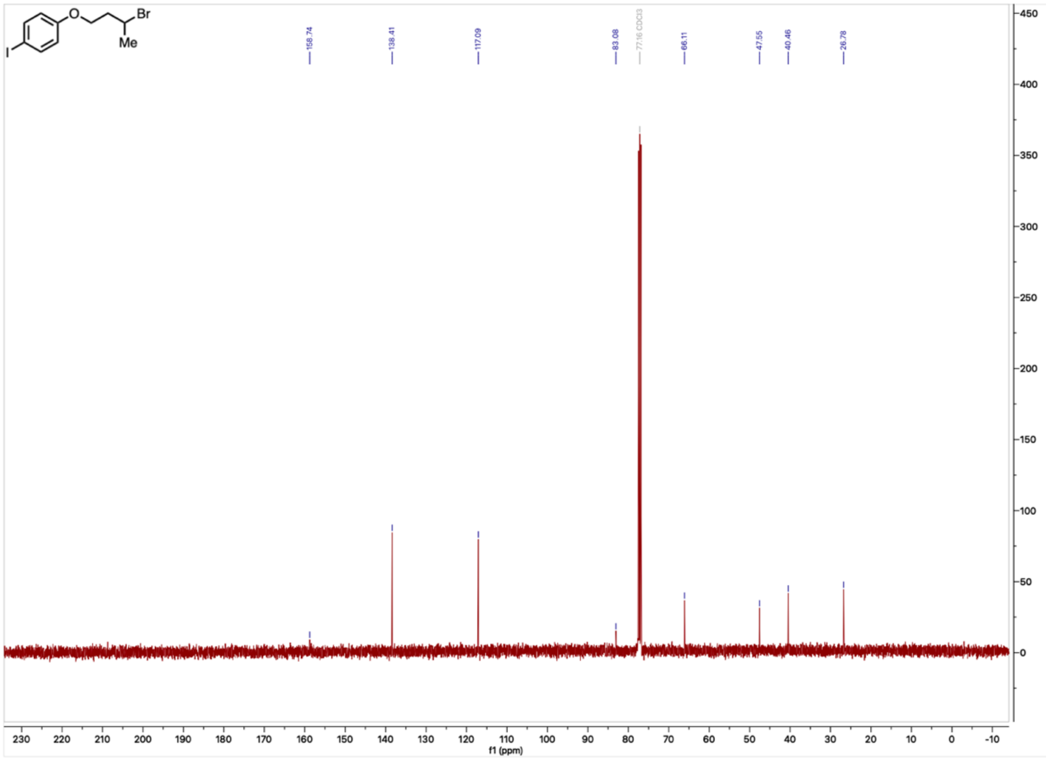

3b ^{1}H (400 MHz, $CDCl_3$)

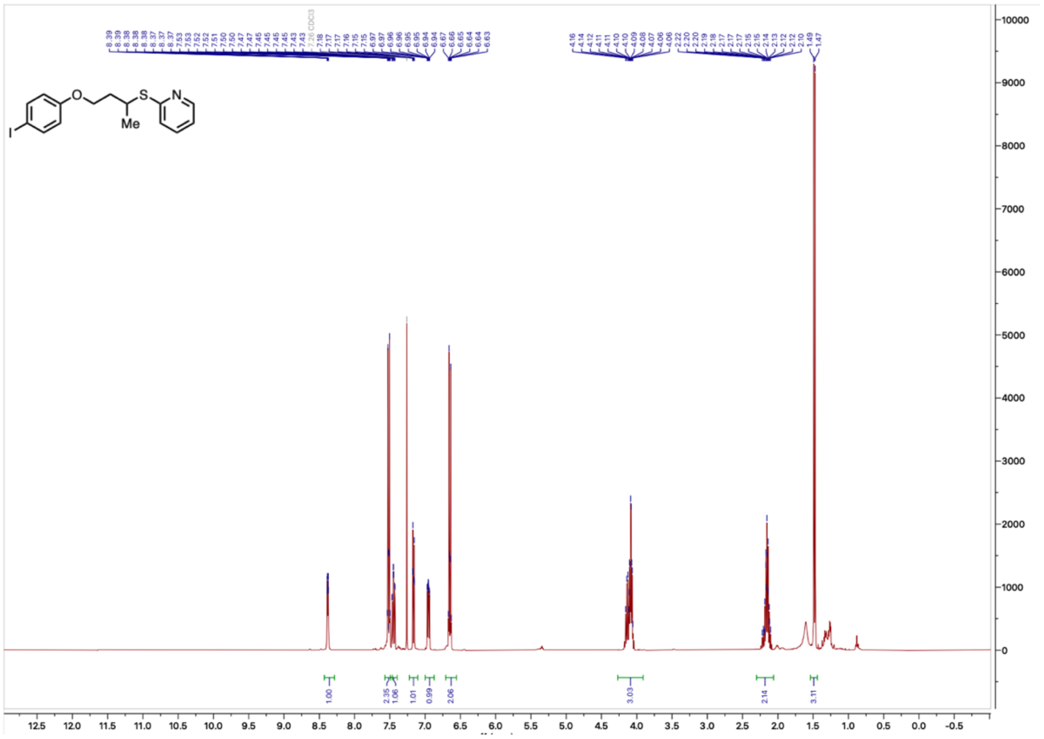

3b ^{13}C (101 MHz, $CDCl_3$)

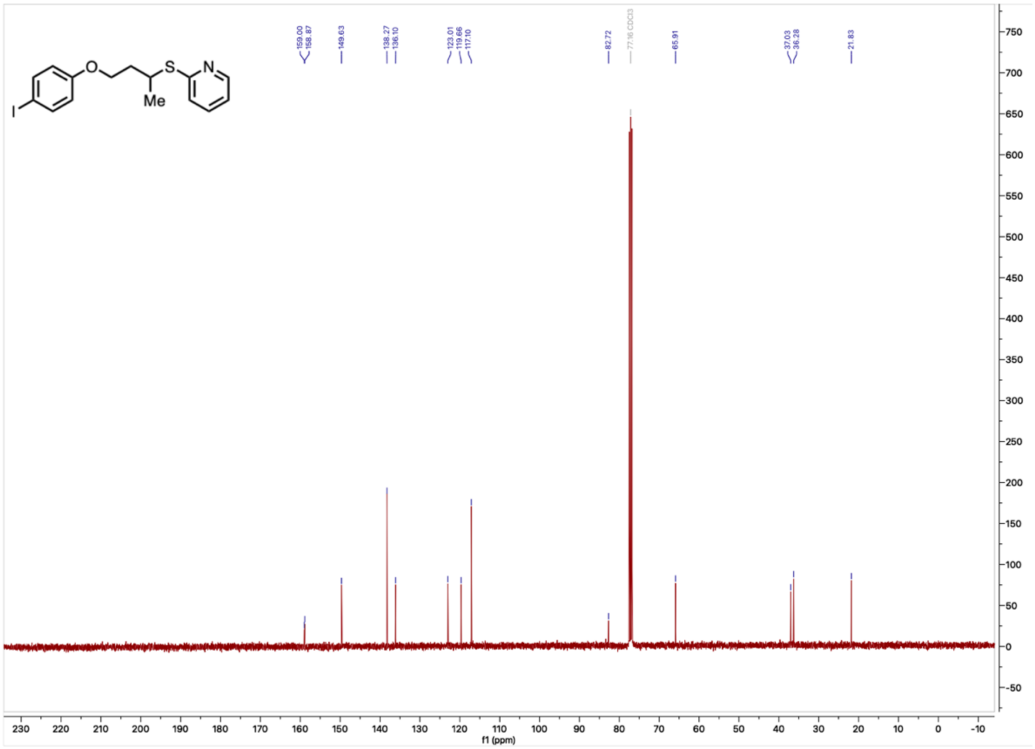

4a ^{1}H (400 MHz, $CDCl_3$)

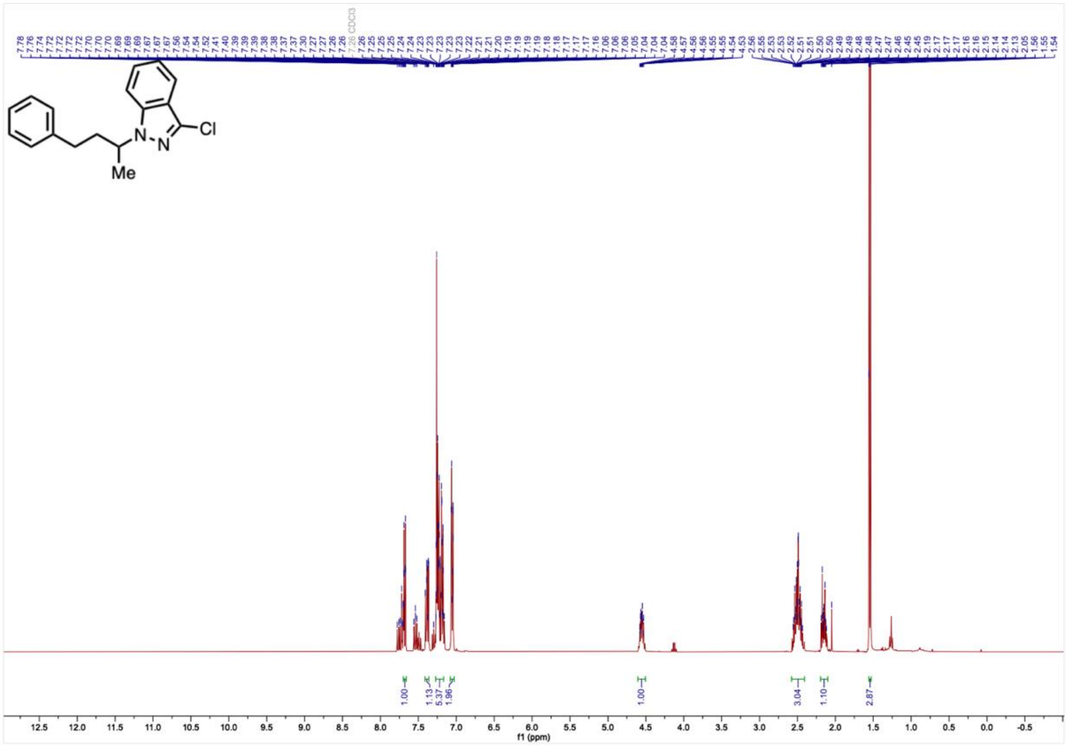

4a ^{13}C (101 MHz, $CDCl_3$)

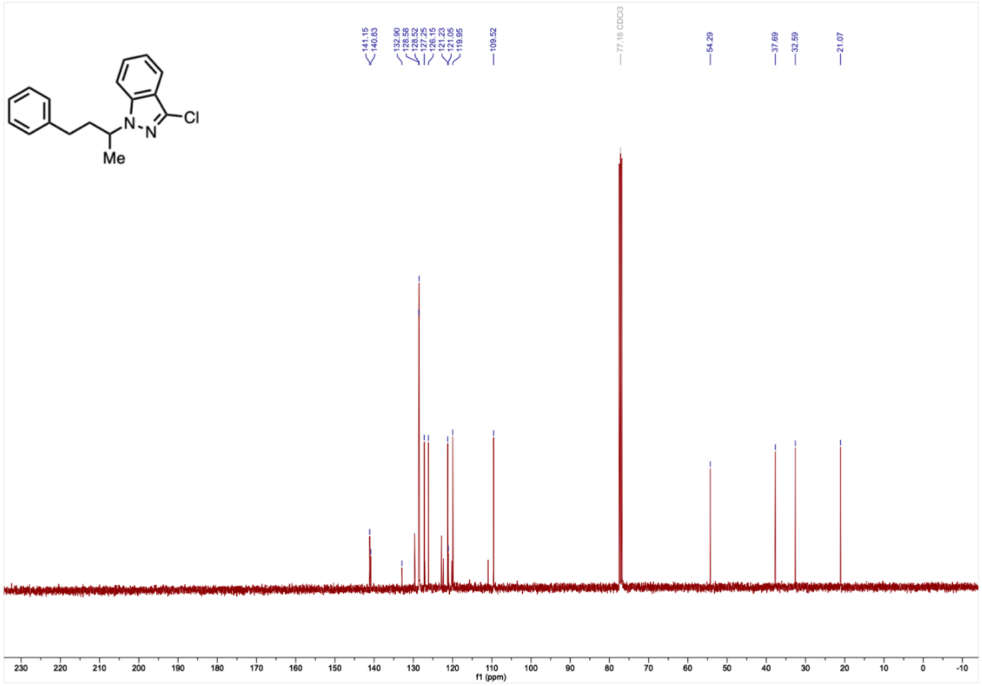

4b ^{1}H (400 MHz, $CDCl_3$)

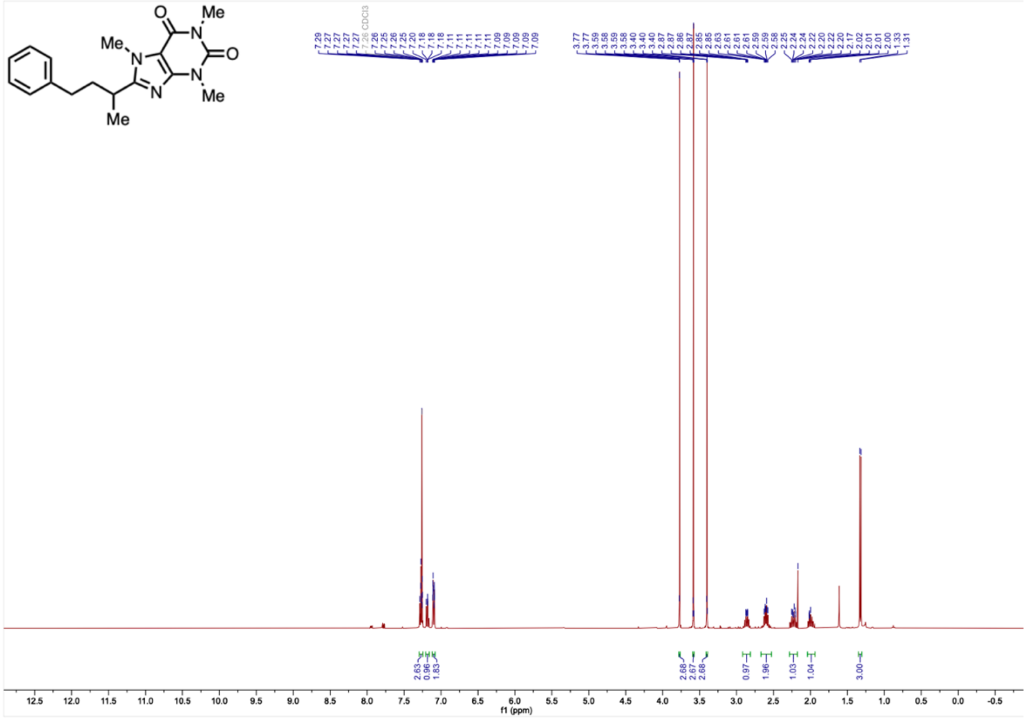

4b ^{13}C (101 MHz, $CDCl_3$)

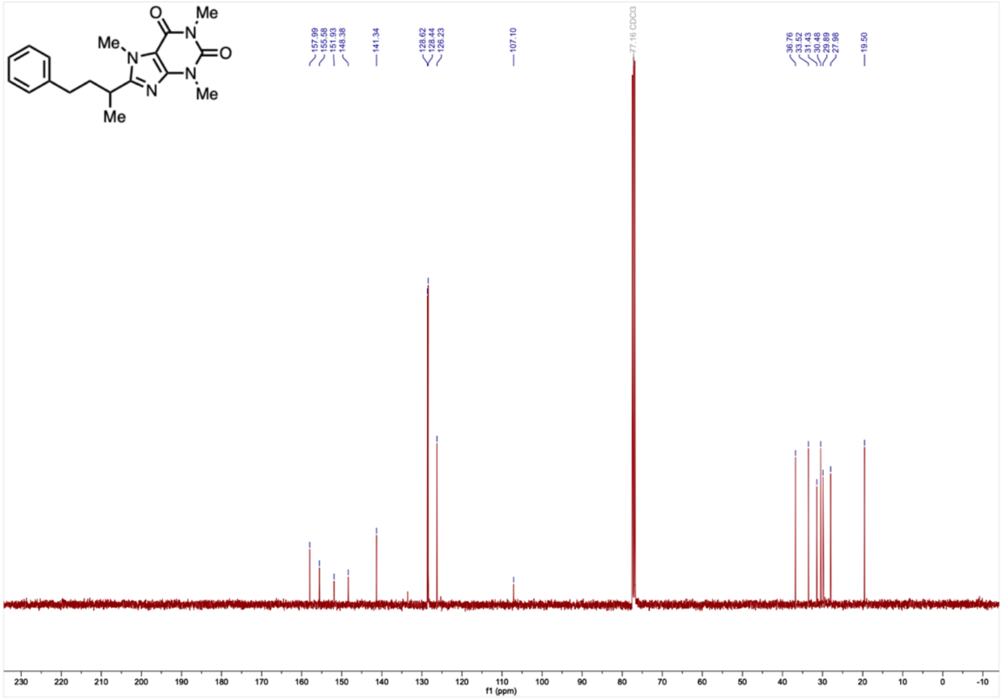

4c ^{1}H (400 MHz, $CDCl_3$)

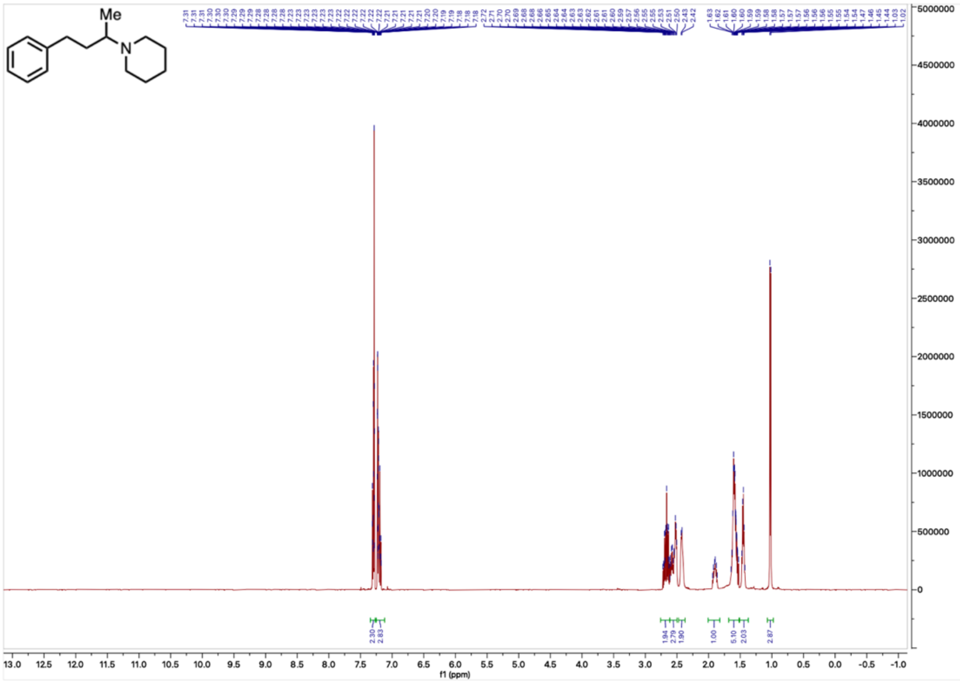

4c ^{13}C (101 MHz, $CDCl_3$)

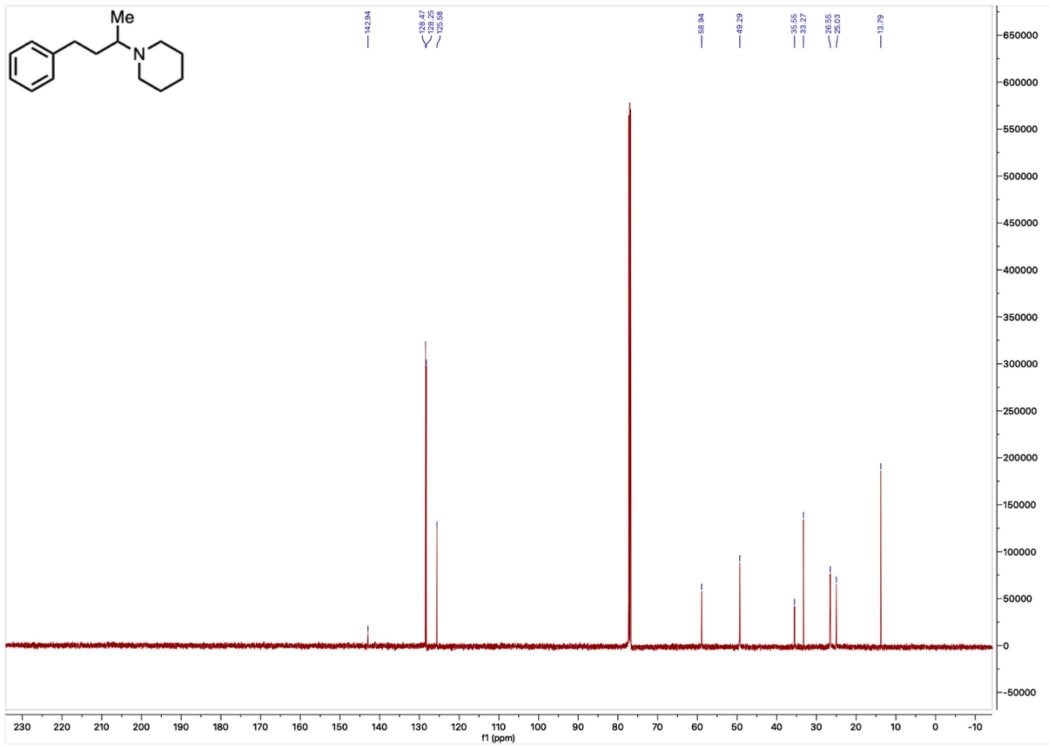

Co-1' ^{1}H (400 MHz, THF-d_8)

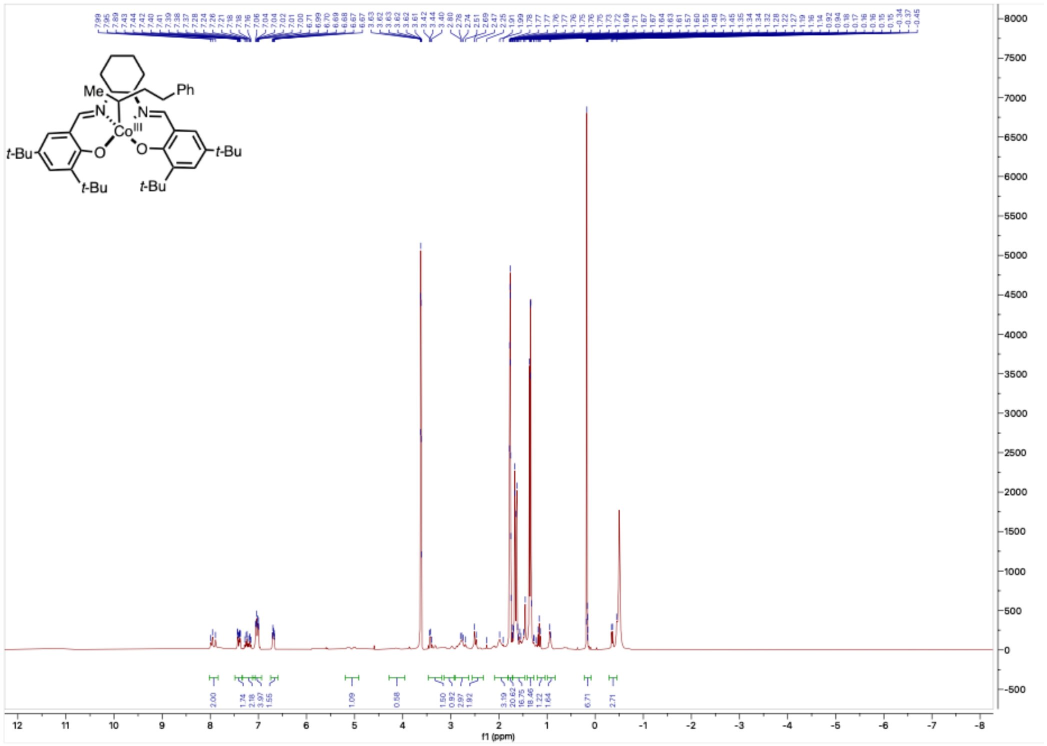

9 X ray crystallographic data

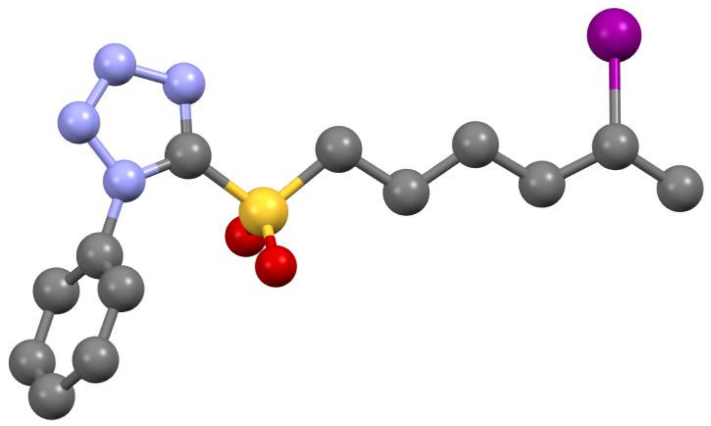

Supplementary Table 3 Crystal data and structure refinement for **2p.**

Empirical formula	$C_{13}H_{17}IN_4O_2S$
Formula weight	420.26
Temperature/K	100.0(1)
Crystal system	monoclinic
Space group	I2/a
a/Å	29.4997(5)
b/Å	5.46440(10)
c/Å	20.4182(4)
α/°	90
β/°	103.310(2)
γ/°	90
Volume/Å^3	3202.96(10)
Z	8
ρ_{calc}g/cm^3	1.743
μ/mm^{-1}	2.139
F(000)	1664.0
Crystal size/mm^3	0.204 × 0.144 × 0.11
Radiation	Mo Kα (λ = 0.71073)
2Θ range for data collection/°	5.498 to 56.562
Index ranges	-39 ≤ h ≤ 38, -7 ≤ k ≤ 7, -27 ≤ l ≤ 27
Reflections collected	47414
Independent reflections	3974 [R_{int} = 0.0428, R_{sigma} = 0.0174]
Data/restraints/parameters	3974/154/236

Goodness-of-fit on F^2	1.403
Final R indexes [I>=2σ (I)]	R_1 = 0.0433, wR_2 = 0.1025
Final R indexes [all data]	R_1 = 0.0440, wR_2 = 0.1027
Largest diff. peak/hole / e Å^{-3}	0.61/-1.26

Supplementary Table 4 Fractional Atomic Coordinates (×10^4) and Equivalent Isotropic Displacement Parameters (Å^2×10^3) for **2p**. U_{eq} is defined as 1/3 of the trace of the orthogonalised U_{IJ} tensor.

**Atom**	***x***	***y***	***z***	**U(eq)**
I1	6700.5(2)	10737.0(7)	3991.8(3)	30.78(13)
S1	4402.0(3)	5291.1(19)	3713.2(5)	19.6(2)
O1	4523.2(11)	3874(6)	4319.0(15)	27.3(7)
O2	4062.8(10)	7204(6)	3665.0(16)	27.9(7)
N1	3750.1(11)	2293(7)	2885.9(16)	20.0(7)
N2	3734.9(13)	583(7)	2404.3(18)	26.1(7)
N3	4150.9(13)	456(7)	2292.8(18)	27.2(8)
N4	4445.0(12)	2062(7)	2698.0(17)	24.0(7)
C1	3345.9(13)	2810(8)	3155(2)	21.4(8)
C2	3247.3(16)	1229(9)	3624(3)	31.5(10)
C3	2858.7(17)	1702(10)	3887(3)	37.6(12)
C4	2583.2(15)	3708(9)	3672(3)	32.5(10)
C5	2690.6(17)	5275(11)	3200(3)	41.5(13)
C6	3080.4(17)	4850(10)	2941(3)	36.4(11)
C7	4189.4(13)	3160(8)	3055.1(19)	20.2(8)
C8	4910.4(13)	6479(8)	3507(2)	22.3(8)
C9	5132.2(14)	8265(8)	4067(2)	23.5(8)
C10	5546.2(15)	9571(9)	3896(2)	27.0(9)
C11	5798.0(16)	11184(9)	4477(2)	28.2(9)
C12	6169.9(18)	12835(10)	4325(3)	29.9(10)
C13	6407(3)	14429(12)	4917(3)	34.2(12)
I1A	6682.5(17)	14286(8)	5021.1(19)	34.9(12)
C13A	6327(16)	11920(120)	3670(16)	59(13)
C12A	6118(11)	13030(60)	4229(12)	29.9(10)
I1B	6790(9)	10580(50)	4350(20)	30.78(13)
C12B	6247(8)	10060(90)	4882(14)	30(15)
C13B	6200(20)	7320(120)	5010(60)	50(30)

Supplementary Table 5 Anisotropic Displacement Parameters (Å^2×10^3) for **2p.** The Anisotropic displacement factor exponent takes the form: $-2\pi^2[h^2a^{*2}U_{11}+2hka^*b^*U_{12}+\ldots]$.

**Atom**	**U_{11}**	**U_{22}**	**U_{33}**	**U_{23}**	**U_{13}**	**U_{12}**
I1	24.01(17)	30.20(18)	42.6(3)	1.44(16)	16.89(16)	1.55(13)
S1	16.4(4)	24.4(5)	18.3(4)	-2.0(4)	4.6(3)	1.4(4)
O1	27.7(15)	32.1(17)	21.4(14)	0.1(13)	4.3(12)	-1.9(13)
O2	19.9(14)	29.3(16)	35.3(17)	-5.2(14)	7.7(12)	4.9(13)
N1	18.3(15)	23.6(17)	17.5(15)	0.2(13)	2.8(12)	1.0(13)
N2	25.5(17)	28.2(19)	24.0(17)	-3.5(15)	4.7(14)	-0.2(15)

Supplementary Table 5 Anisotropic Displacement Parameters (Å^2×10^3) for **2p.** The Anisotropic displacement factor exponent takes the form: $-2\pi^2[h^2a^{*2}U_{11}+2hka^*b^*U_{12}+\ldots]$.

**Atom**	**U_{11}**	**U_{22}**	**U_{33}**	**U_{23}**	**U_{13}**	**U_{12}**
N3	28.0(18)	30(2)	25.1(18)	-5.8(16)	8.4(14)	-1.1(16)
N4	23.3(17)	28.3(19)	20.8(16)	-2.4(15)	5.8(13)	1.6(15)
C1	13.0(16)	26(2)	26.0(19)	-3.6(16)	5.4(14)	-0.1(15)
C2	26(2)	29(2)	42(3)	10(2)	11.8(19)	7.3(18)
C3	31(2)	40(3)	48(3)	13(2)	21(2)	6(2)
C4	20(2)	34(2)	46(3)	1(2)	13.4(19)	2.4(18)
C5	28(2)	43(3)	56(3)	18(3)	15(2)	19(2)
C6	30(2)	44(3)	38(3)	15(2)	14(2)	12(2)
C7	18.5(17)	26(2)	15.6(17)	0.1(15)	3.0(14)	2.5(15)
C8	17.3(17)	26(2)	24.3(19)	-1.5(16)	7.4(15)	-0.6(16)
C9	22.2(19)	25(2)	23.8(19)	-3.9(16)	6.1(15)	-1.5(16)
C10	23(2)	31(2)	28(2)	-3.2(18)	6.8(16)	-3.7(17)
C11	28(2)	29(2)	28(2)	-2.2(18)	6.9(17)	-2.8(17)
C12	27(2)	30(2)	30(2)	6(2)	1.8(19)	-0.7(19)
C13	38(3)	35(3)	30(3)	-4(2)	10(3)	-11(3)
I1A	40(3)	37(2)	27.2(18)	-3.3(14)	7.3(16)	-13.2(18)
C13A	90(30)	50(30)	50(20)	-2(19)	39(19)	-10(20)
C12A	27(2)	30(2)	30(2)	6(2)	1.8(19)	-0.7(19)
I1B	24.01(17)	30.20(18)	42.6(3)	1.44(16)	16.89(16)	1.55(13)
C12B	28(16)	30(20)	30(30)	0(20)	8(19)	-1(17)
C13B	40(50)	30(30)	60(80)	10(30)	10(60)	0(30)

Supplementary Table 6 Bond Lengths for **2p.**

**Atom**	**Atom**	**Length/Å**	**Atom**	**Atom**	**Length/Å**
I1	C12	2.172(6)	C3	C4	1.376(7)
S1	O1	1.433(3)	C4	C5	1.379(7)
S1	O2	1.435(3)	C5	C6	1.392(7)
S1	C7	1.779(4)	C8	C9	1.530(6)
S1	C8	1.772(4)	C9	C10	1.522(6)
N1	N2	1.350(5)	C10	C11	1.526(6)
N1	C1	1.451(5)	C11	C12	1.506(7)
N1	C7	1.348(5)	C11	C12A	1.543(18)
N2	N3	1.300(5)	C11	C12B	1.52(2)
N3	N4	1.370(5)	C12	C13	1.522(8)
N4	C7	1.309(5)	I1A	C12A	2.149(19)
C1	C2	1.370(6)	C13A	C12A	1.54(2)
C1	C6	1.375(6)	I1B	C12B	2.16(2)
C2	C3	1.398(6)	C12B	C13B	1.53(2)

Supplementary Table 7 Bond Angles for **2p.**

Atom	Atom	Atom	Angle/˚	Atom	Atom	Atom	Angle/˚
O1	S1	O2	118.96(19)	C1	C6	C5	118.4(5)
O1	S1	C7	105.8(2)	N1	C7	S1	125.1(3)
O1	S1	C8	110.27(19)	N4	C7	S1	124.9(3)
O2	S1	C7	107.81(19)	N4	C7	N1	109.8(4)
O2	S1	C8	109.4(2)	C9	C8	S1	107.2(3)
C8	S1	C7	103.35(19)	C10	C9	C8	110.8(3)
N2	N1	C1	121.2(3)	C9	C10	C11	111.5(4)
C7	N1	N2	107.4(3)	C10	C11	C12A	110.4(9)
C7	N1	C1	131.3(3)	C12	C11	C10	115.7(4)
N3	N2	N1	106.9(3)	C12B	C11	C10	113.3(16)
N2	N3	N4	110.6(3)	C11	C12	I1	111.1(4)
C7	N4	N3	105.3(3)	C11	C12	C13	113.8(5)
C2	C1	N1	117.9(4)	C13	C12	I1	108.6(4)
C2	C1	C6	122.4(4)	C11	C12A	I1A	112.9(14)
C6	C1	N1	119.7(4)	C13A	C12A	C11	111.4(18)
C1	C2	C3	118.5(4)	C13A	C12A	I1A	108.1(16)
C4	C3	C2	120.0(5)	C11	C12B	I1B	108.9(18)
C3	C4	C5	120.3(4)	C11	C12B	C13B	113(2)
C4	C5	C6	120.3(5)	C13B	C12B	I1B	108.6(17)

Supplementary Table 8 Torsion Angles for **2p.**

A	B	C	D	Angle/˚	A	B	C	D	Angle/˚
S1	C8	C9	C10	-174.3(3)	C2	C1	C6	C5	-1.7(8)
O1	S1	C7	N1	83.3(4)	C2	C3	C4	C5	-0.6(9)
O1	S1	C7	N4	-91.2(4)	C3	C4	C5	C6	-0.4(9)
O1	S1	C8	C9	-64.8(3)	C4	C5	C6	C1	1.5(9)
O2	S1	C7	N1	-45.0(4)	C6	C1	C2	C3	0.7(8)
O2	S1	C7	N4	140.5(4)	C7	S1	C8	C9	-177.5(3)
O2	S1	C8	C9	67.8(3)	C7	N1	N2	N3	-0.2(5)
N1	N2	N3	N4	0.2(5)	C7	N1	C1	C2	-97.0(6)
N1	C1	C2	C3	-179.9(4)	C7	N1	C1	C6	82.4(6)
N1	C1	C6	C5	179.0(5)	C8	S1	C7	N1	-160.7(4)
N2	N1	C1	C2	78.9(5)	C8	S1	C7	N4	24.8(4)
N2	N1	C1	C6	-101.8(5)	C8	C9	C10	C11	-174.8(4)
N2	N1	C7	S1	-175.1(3)	C9	C10	C11	C12	-171.6(4)
N2	N1	C7	N4	0.1(5)	C9	C10	C11	C12A	-163.7(18)
N2	N3	N4	C7	-0.1(5)	C9	C10	C11	C12B	102.4(16)
N3	N4	C7	S1	175.2(3)	C10	C11	C12	I1	-57.8(5)
N3	N4	C7	N1	0.0(5)	C10	C11	C12	C13	179.3(5)
C1	N1	N2	N3	-176.9(4)	C10	C11	C12A	I1A	-155.9(14)
C1	N1	C7	S1	1.2(6)	C10	C11	C12A	C13A	-34(3)
C1	N1	C7	N4	176.4(4)	C10	C11	C12B	I1B	79(2)
C1	C2	C3	C4	0.4(8)	C10	C11	C12B	C13B	-41(5)

Supplementary Table 9 Hydrogen Atom Coordinates (Å×10^4) and Isotropic Displacement Parameters (Å^2×10^3) for **2p.**

**Atom**	***x***	***y***	***z***	**U(eq)**
H2	3438.69	-157.58	3766.93	38
H3	2784.93	636.24	4215.55	45
H4	2317.62	4015.94	3848.54	39
H5	2497.59	6651.84	3051.74	50
H6	3160.91	5942.41	2624.07	44
H8A	5130.11	5137.52	3475.82	27
H8B	4829.24	7336.15	3068.35	27
H9A	4897.63	9486.88	4127.78	28
H9B	5236.56	7359.42	4494.84	28
H10A	5436.45	10592.65	3490.23	32
H10B	5766.39	8342	3793.06	32
H11A	5563.67	12213.53	4623.7	34
H11B	5942.15	10113.46	4858.95	34
H11C	5986.01	10148.32	4836.28	34
H11D	5566.67	12082.32	4667.07	34
H11E	5870.93	12776.9	4293.49	34
H11F	5587.01	11502.84	4780.26	34
H12	6019.02	13945.02	3948.83	36
H13A	6563.01	13384.95	5291.31	51
H13B	6172.62	15440.82	5059.85	51
H13C	6636.74	15487.69	4780.22	51
H13D	6528.51	13125.88	3523.97	89
H13E	6510.83	10462.88	3843.32	89
H13F	6075.17	11452.02	3287.26	89
H12A	5926.45	14477.07	4038.25	36
H12B	6337.69	10911.84	5326.25	36
H13G	6498.86	6684.07	5268.37	68
H13H	5958.63	7050.07	5255.45	68
H13I	6117	6457.43	4573.57	68

Supplementary Table 10 Atomic Occupancy for **2p.**

**Atom**	***Occupancy***	**Atom**	***Occupancy***	**Atom**	***Occupancy***
I1	0.8970(18)	H11A	0.8970(18)	H11B	0.8970(18)
H11C	0.0897(14)	H11D	0.0897(14)	H11E	0.0132(13)
H11F	0.0132(13)	C12	0.8970(18)	H12	0.8970(18)
C13	0.8970(18)	H13A	0.8970(18)	H13B	0.8970(18)
H13C	0.8970(18)	I1A	0.0897(14)	C13A	0.0897(14)
H13D	0.0897(14)	H13E	0.0897(14)	H13F	0.0897(14)
C12A	0.0897(14)	H12A	0.0897(14)	I1B	0.0132(13)
C12B	0.0132(13)	H12B	0.0132(13)	C13B	0.0132(13)
H13G	0.0132(13)	H13H	0.0132(13)	H13I	0.0132(13)

Experimental. A suitable crystal was selected on a XtaLAB Synergy, Dualflex, Pilatus 300K diffractometer. The crystal was kept at 100.0(1) K during data collection. Using Olex2, the structure was solved with the SHELXT structure solution program using Intrinsic Phasing and refined with the SHELXL refinement package using Least Squares minimisation.

Crystal structure determination of **2p** Crystal Data for C13H17IN4O2S (M =420.26 g/mol): monoclinic, space group I2/a (no. 15), a = 29.4997(5) Å, b = 5.46440(10) Å, c = 20.4182(4) Å, β = 103.310(2)°, V = 3202.96(10) Å3, Z = 8, T = 100.0(1) K, μ(Mo Kα) = 2.139 mm-1, Dcalc = 1.743 g/cm3, 47414 reflections measured (5.498° ≤ 2Θ ≤ 56.562°), 3974 unique (Rint = 0.0428, Rsigma = 0.0174) which were used in all calculations. The final R1 was 0.0433 (I > 2σ(I)) and wR2 was 0.1027 (all data).

Refinement model description Number of restraints - 154, number of constraints - unknown. Details:

1. Fixed Uiso At 1.2 times of: All C(H) groups, All C(H,H) groups, All C(H,H,H,H,H,H) groups At 1.5 times of: All C(H,H,H) groups

2. Restrained distances I1B-I1 -1.5 with sigma of 0.02 I1B-C12B ≈ I1-C12 ≈ I1A-C12A with sigma of 0.02 I1-C13 ≈ I1A-C13A ≈ I1B-C13B with sigma of 0.02 C12B-C11 ≈ C12A-C11 ≈ C12-C11 with sigma of 0.02 C13-C12 ≈ C13B-C12B ≈ C13A-C12A with sigma of 0.02 C11-C13 ≈ C11-C13A ≈ C11-C13B with sigma of 0.02 C10-C12 ≈ C10-C12A ≈ C10-C12B with sigma of 0.02

3. Uiso/Uaniso restraints and constraints I1 ≈ C11 ≈ C12 ≈ C13 ≈ I1A ≈ C13A ≈ C12A ≈ I1B ≈ C12B ≈ C13B: within 2A with sigma of 0.04 and sigma for terminal atoms of 0.08 within 2A Uanis(I1) = Uanis(I1B) Uanis(C12) = Uanis(C12A)

4. Rigid body (RIGU) restrains I1, C11, C12, C13, I1A, C13A, C12A, I1B, C12B, C13B with sigma for 1-2 distances of 0.004 and sigma for 1-3 distances of 0.004

5. Others 1*[Sof(I1)+Sof(H11A)+Sof(H11B)+Sof(C12)+Sof(H12)+Sof(C13)+Sof(H13A)+Sof(H13B)+ Sof(H13C)]+1*[Sof(H11C)+Sof(H11D)+Sof(I1A)+Sof(C13A)+Sof(H13D)+Sof(H13E)+ Sof(H13F)+Sof(C12A)+Sof(H12A)]+1*[Sof(H11E)+Sof(H11F)+Sof(I1B)+Sof(C12B)+ Sof(H12B)+Sof(C13B)+Sof(H13G)+Sof(H13H)+Sof(H13I)]=1 with esd of 0.0001 Sof(I1)=Sof(H11A)=Sof(H11B)=Sof(C12)=Sof(H12)=Sof(C13)=Sof(H13A)=Sof(H13B)= Sof(H13C)=FVAR(1) Sof(H11C)=Sof(H11D)=Sof(I1A)=Sof(C13A)=Sof(H13D)=Sof(H13E)=Sof(H13F)= Sof(C12A)=Sof(H12A)=FVAR(2) Sof(H11E)=Sof(H11F)=Sof(I1B)=Sof(C12B)=Sof(H12B)=Sof(C13B)=Sof(H13G)= Sof(H13H)=Sof(H13I)=FVAR(3) 6.a Ternary CH refined with riding coordinates: C12(H12), C12A(H12A),

C12B(H12B) 6.b Secondary CH2 refined with riding coordinates: C8(H8A,H8B), C9(H9A,H9B), C10(H10A,H10B), C11(H11A,H11B), C11(H11C,H11D), C11(H11E,H11F) 6.c Me refined with riding coordinates: C13A(H13D,H13E,H13F), C13B(H13G,H13H,H13I) 6.d Aromatic/amide H refined with riding coordinates: C2(H2), C3(H3), C4(H4), C5(H5), C6(H6) 6.e Idealized Me refined as rotating group: C13(H13A,H13B,H13C).

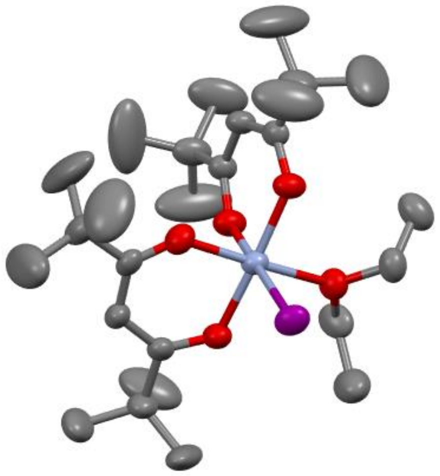

Supplementary Table 11 Crystal data and structure refinement for **Cr-1'.**

Empirical formula	$C_{26}H_{48}CrIO_5$
Formula weight	619.54
Temperature [K]	100.0(1)
Crystal system	monoclinic
Space group (number)	$P2_1/n$ (14)
a [Å]	13.6647(2)
b [Å]	16.2925(2)
c [Å]	14.2459(2)
α [°]	90
β [°]	94.2550(10)
γ [°]	90
Volume [Å^3]	3162.85(8)
Z	4
ρ_{calc} [gcm^{-3}]	1.301
μ [mm^{-1}]	10.862
F(000)	1284
Crystal size [mm^3]	0.087×0.078×0.047
Crystal colour	clear dark green
Crystal shape	block
Radiation	Cu K_α (λ=1.54184 Å)
2θ range [°]	8.26 to 159.86 (0.78 Å)
Index ranges	−17 ≤ h ≤ 17<br>−18 ≤ k ≤ 20<br>−18 ≤ l ≤ 17
Reflections collected	33148

Independent reflections	6681<br>R_{int} = 0.0452<br>R_{sigma} = 0.0329
Completeness to<br>θ = 67.684°	99.7 %
Data / Restraints / Parameters	6681/2050/537
Absorption correction	0.536/0.771
T_{min}/T_{max} (method)	(gaussian)
Goodness-of-fit on F^2	1.045
Final R indexes<br>[I≥2σ(I)]	R_1 = 0.0472<br>wR_2 = 0.1302
Final R indexes<br>[all data]	R_1 = 0.0566<br>wR_2 = 0.1378
Largest peak/hole [eÅ^{-3}]	1.31/−0.72

Supplementary Table 12. Atomic coordinates and Ueq [Å2] for **Cr-1'**.

Atom	*x*	*y*	*Z*	U_{eq}
I1	0.81697(2)	0.17437(2)	0.78310(2)	0.05273(12)
Cr1	0.76476(4)	0.27543(4)	0.63662(4)	0.03784(16)
O1	0.90151(19)	0.29610(18)	0.6134(2)	0.0439(6)
O2	0.7598(2)	0.18631(19)	0.5488(2)	0.0520(7)
O3	0.62553(19)	0.25866(19)	0.6508(2)	0.0462(6)
O4	0.7295(2)	0.3505(2)	0.5338(2)	0.0484(7)
O5	0.7661(2)	0.37504(18)	0.7239(2)	0.0478(6)
C1	0.9488(4)	0.2605(4)	0.5507(4)	0.0393(11)
C2	0.9139(4)	0.1950(4)	0.4946(4)	0.0468(13)
H2	0.956717	0.172039	0.451948	0.056
C3	0.8211(4)	0.1611(3)	0.4969(4)	0.0410(12)
C4	0.5556(4)	0.2727(3)	0.5888(4)	0.0362(11)
C5	0.5644(4)	0.3153(3)	0.5052(4)	0.0411(12)
H5	0.509680	0.316024	0.459946	0.049
C6	0.6497(4)	0.3571(4)	0.4841(4)	0.0391(11)
C7	0.7037(5)	0.3795(4)	0.8026(4)	0.0539(15)
H7A	0.741864	0.401799	0.858704	0.065
H7B	0.681197	0.323685	0.818170	0.065
C8	0.6164(7)	0.4334(7)	0.7781(9)	0.068(3)
H8A	0.588664	0.420301	0.714476	0.102
H8B	0.636768	0.491091	0.780730	0.102
H8C	0.566715	0.424052	0.823188	0.102
C9	0.8304(5)	0.4446(4)	0.7158(6)	0.0594(17)
H9A	0.793954	0.495677	0.727512	0.071
H9B	0.851693	0.447142	0.650980	0.071
C10	0.9197(6)	0.4393(7)	0.7849(7)	0.090(3)
H10A	0.966900	0.481934	0.770259	0.135
H10B	0.950230	0.385189	0.780174	0.135
H10C	0.899988	0.447459	0.848973	0.135
C11	1.0509(5)	0.2983(6)	0.5387(7)	0.058(2)
C12	1.1072(9)	0.3062(10)	0.6328(7)	0.118(6)
H12A	1.068038	0.337165	0.675677	0.176
H12B	1.121238	0.251464	0.658887	0.176
H12C	1.168931	0.335235	0.625407	0.176
C13	1.0324(7)	0.3829(6)	0.4974(11)	0.129(5)
H13A	0.995683	0.378062	0.435893	0.193
H13B	0.994378	0.415434	0.539680	0.193
H13C	1.095320	0.410152	0.489770	0.193
C14	1.1116(6)	0.2506(7)	0.4731(7)	0.095(3)
H14A	1.075135	0.245283	0.411561	0.143
H14B	1.173354	0.279693	0.465919	0.143
H14C	1.125662	0.195923	0.499399	0.143
C15	0.7876(6)	0.0871(5)	0.4378(5)	0.0601(19)
C16	0.7121(9)	0.1151(7)	0.3663(8)	0.136(5)
H16A	0.661882	0.146339	0.396775	0.204
H16B	0.742210	0.150253	0.320667	0.204
H16C	0.681630	0.067511	0.333876	0.204
C17	0.8682(7)	0.0390(8)	0.4011(10)	0.139(5)

H17A	0.914956	0.022950	0.453281	0.209
H17B	0.841152	-0.010289	0.369350	0.209
H17C	0.901732	0.072452	0.356141	0.209
C18	0.7350(12)	0.0270(7)	0.5002(9)	0.152(5)
H18A	0.781791	0.005858	0.549862	0.228
H18B	0.681528	0.055537	0.528828	0.228
H18C	0.708151	-0.018730	0.461755	0.228
C19A	0.4605(12)	0.2209(9)	0.6010(10)	0.046(4)
C20A	0.4570(9)	0.1945(8)	0.7020(8)	0.036(3)
H20A	0.485299	0.237585	0.743599	0.053
H20B	0.388648	0.184921	0.715639	0.053
H20C	0.494656	0.143697	0.712489	0.053
C21A	0.4186(13)	0.1582(8)	0.5341(12)	0.074(5)
H21A	0.455465	0.106868	0.543199	0.111
H21B	0.349678	0.148642	0.545526	0.111
H21C	0.422967	0.177657	0.469480	0.111
C22A	0.4061(14)	0.2995(7)	0.5889(9)	0.069(5)
H22A	0.434893	0.340077	0.633668	0.103
H22B	0.410420	0.319683	0.524560	0.103
H22C	0.337131	0.290669	0.600606	0.103
C23	0.6510(5)	0.4119(4)	0.3980(5)	0.0567(17)
C24	0.6568(12)	0.3540(7)	0.3158(7)	0.152(5)
H24A	0.599370	0.317755	0.311798	0.228
H24B	0.658116	0.385653	0.257398	0.228
H24C	0.716649	0.320873	0.324775	0.228
C25	0.5579(6)	0.4591(6)	0.3800(8)	0.102(3)
H25A	0.502229	0.420952	0.375429	0.154
H25B	0.550543	0.497539	0.431893	0.154
H25C	0.559653	0.489621	0.320910	0.154
C26	0.7357(8)	0.4664(9)	0.4053(10)	0.158(6)
H26A	0.795714	0.433923	0.417029	0.237
H26B	0.738761	0.497046	0.346343	0.237
H26C	0.729651	0.504964	0.457326	0.237
C1A	0.7021(10)	0.1436(11)	0.4999(12)	0.068(4)
C12A	0.8258(16)	0.0243(12)	0.4643(14)	0.083(5)
H12D	0.884991	0.051669	0.491864	0.125
H12E	0.796475	-0.008350	0.512746	0.125
H12F	0.843103	-0.011754	0.413036	0.125
C11A	0.7517(13)	0.0892(10)	0.4255(11)	0.068(4)
C13A	0.8010(11)	0.1254(11)	0.3397(11)	0.063(4)
H13D	0.859790	0.156211	0.361899	0.094
H13E	0.819380	0.080649	0.298548	0.094
H13F	0.754793	0.162159	0.304567	0.094
C14A	0.6628(13)	0.0434(16)	0.3844(18)	0.107(7)
H14D	0.613517	0.082683	0.358784	0.160
H14E	0.681868	0.007140	0.333976	0.160
H14F	0.635240	0.010545	0.433686	0.160
C2A	0.6063(13)	0.1626(13)	0.5258(15)	0.082(5)
H2A	0.556777	0.126194	0.500614	0.098
C3A	0.5702(11)	0.2279(13)	0.5839(10)	0.083(5)
C20	0.3800(8)	0.2301(10)	0.5380(8)	0.159(7)
H20D	0.368598	0.286358	0.515397	0.239

H20E	0.398783	0.195544	0.485942	0.239
H20F	0.319834	0.208536	0.562149	0.239
C19	0.4592(6)	0.2299(6)	0.6136(7)	0.064(2)
C22	0.4804(9)	0.1454(7)	0.6514(11)	0.146(5)
H22D	0.533515	0.148025	0.701456	0.219
H22E	0.421339	0.122883	0.676773	0.219
H22F	0.500288	0.109891	0.600566	0.219
C21	0.4229(9)	0.2761(10)	0.6982(9)	0.158(6)
H21D	0.474725	0.276723	0.749543	0.237
H21E	0.406055	0.332609	0.679698	0.237
H21F	0.364699	0.248532	0.719281	0.237
C6A	0.9342(11)	0.3268(12)	0.5428(10)	0.066(4)
C5A	0.8748(10)	0.3651(14)	0.4707(12)	0.071(4)
H5A	0.906708	0.386044	0.418785	0.085
C4A	0.7734(10)	0.3751(11)	0.4686(10)	0.067(4)
C15A	0.7073(13)	0.4243(10)	0.3965(11)	0.086(5)
C16A	0.7665(18)	0.4510(17)	0.3186(13)	0.127(8)
H16D	0.822416	0.483814	0.344317	0.190
H16E	0.790596	0.402678	0.286530	0.190
H16F	0.725681	0.484221	0.273738	0.190
C18A	0.6219(16)	0.3746(16)	0.362(2)	0.124(9)
H18D	0.584610	0.358065	0.414676	0.186
H18E	0.579721	0.407051	0.317070	0.186
H18F	0.644636	0.325508	0.329862	0.186
C17A	0.6727(15)	0.4999(9)	0.4456(13)	0.076(5)
H17D	0.729541	0.532792	0.468645	0.114
H17E	0.630553	0.532565	0.401156	0.114
H17F	0.635498	0.483527	0.498729	0.114
C23A	1.0454(13)	0.3156(15)	0.5370(15)	0.072(5)
C24A	1.074(3)	0.2281(13)	0.525(2)	0.121(8)
H24D	1.044801	0.207333	0.464867	0.181
H24E	1.145940	0.224171	0.525815	0.181
H24F	1.051222	0.195202	0.576631	0.181
C25A	1.079(2)	0.3654(18)	0.4569(13)	0.104(7)
H25D	1.049734	0.343316	0.397219	0.156
H25E	1.058533	0.422565	0.463680	0.156
H25F	1.150675	0.362716	0.457357	0.156
C26A	1.089(2)	0.3485(17)	0.6281(13)	0.071(6)
H26D	1.069706	0.405914	0.634868	0.107
H26E	1.066360	0.316148	0.680123	0.107
H26F	1.161078	0.345117	0.629307	0.107
C9A	0.8523(14)	0.4051(12)	0.7727(15)	0.084(5)
H9AA	0.903216	0.361913	0.773431	0.101
H9AB	0.838333	0.415957	0.838798	0.101
C10A	0.8923(15)	0.4810(12)	0.733(3)	0.116(9)
H10D	0.867509	0.486573	0.666832	0.174
H10E	0.964108	0.478091	0.736851	0.174
H10F	0.871718	0.528649	0.768560	0.174
C7A	0.6775(12)	0.4248(12)	0.7234(14)	0.072(5)
H7AA	0.637593	0.416575	0.663285	0.087
H7AB	0.695720	0.483568	0.728131	0.087
C8A	0.6181(18)	0.403(2)	0.803(2)	0.097(10)

H8AA	0.553318	0.383197	0.778775	0.145
H8AB	0.610524	0.450838	0.843185	0.145
H8AC	0.651544	0.358920	0.840723	0.145

Supplementary Table 13. Anisotropic displacement parameters [Å2] for **Cr-1'.** The anisotropic displacement factor exponent takes the form: −2π2[h2(a*)2U11 + k2(b*)2U22 + … + 2hka*b*U12].

Atom	U_{11}	U_{22}	U_{33}	U_{23}	U_{13}	U_{12}
I1	0.05843(19)	0.04306(18)	0.05450(18)	0.00733(11)	−0.01054(13)	0.00584(11)
Cr1	0.0338(3)	0.0405(4)	0.0384(3)	−0.0003(3)	−0.0027(2)	0.0001(2)
O1	0.0355(13)	0.0489(16)	0.0465(14)	−0.0050(12)	−0.0011(11)	−0.0018(11)
O2	0.0553(17)	0.0529(18)	0.0471(16)	−0.0070(13)	−0.0008(13)	−0.0077(13)
O3	0.0359(13)	0.0547(18)	0.0474(15)	0.0102(13)	−0.0011(11)	−0.0035(12)
O4	0.0392(14)	0.0598(18)	0.0456(15)	0.0109(13)	−0.0014(11)	−0.0022(13)
O5	0.0530(16)	0.0390(15)	0.0515(16)	−0.0042(12)	0.0047(12)	0.0027(12)
C1	0.032(2)	0.050(3)	0.036(2)	−0.001(2)	−0.0039(19)	0.004(2)
C2	0.039(3)	0.062(4)	0.038(3)	−0.014(2)	−0.003(2)	0.003(2)
C3	0.047(3)	0.044(3)	0.030(2)	−0.002(2)	−0.008(2)	0.004(2)
C4	0.037(2)	0.026(3)	0.045(3)	−0.002(2)	−0.001(2)	0.0065(19)
C5	0.031(2)	0.049(3)	0.042(3)	0.005(2)	−0.003(2)	0.001(2)
C6	0.035(2)	0.044(3)	0.037(2)	0.007(2)	0.0003(19)	0.002(2)
C7	0.063(4)	0.053(4)	0.047(3)	−0.003(3)	0.010(3)	0.008(3)
C8	0.061(5)	0.062(6)	0.083(6)	0.005(4)	0.022(4)	0.008(4)
C9	0.059(4)	0.032(3)	0.088(5)	−0.010(3)	0.011(3)	−0.002(3)
C10	0.064(5)	0.107(8)	0.098(6)	−0.042(6)	0.000(4)	−0.009(5)
C11	0.035(3)	0.084(5)	0.055(4)	−0.013(4)	0.002(3)	−0.009(3)
C12	0.041(6)	0.246(18)	0.064(5)	−0.048(8)	−0.006(4)	−0.020(9)
C13	0.065(6)	0.113(7)	0.211(14)	0.053(9)	0.030(7)	−0.017(5)
C14	0.050(4)	0.161(9)	0.078(5)	−0.038(6)	0.023(4)	−0.017(5)
C15	0.068(4)	0.053(4)	0.055(4)	−0.013(3)	−0.020(3)	0.006(3)
C16	0.156(9)	0.107(8)	0.130(8)	−0.054(6)	−0.089(7)	0.026(7)
C17	0.101(7)	0.127(9)	0.190(11)	−0.097(8)	0.002(7)	0.011(6)
C18	0.219(12)	0.098(8)	0.143(9)	−0.018(7)	0.034(9)	−0.069(8)
C19A	0.048(7)	0.052(8)	0.037(6)	0.010(6)	−0.006(6)	−0.004(6)
C20A	0.025(5)	0.037(7)	0.046(6)	0.016(5)	0.004(4)	0.015(5)
C21A	0.089(11)	0.043(8)	0.085(10)	−0.024(8)	−0.033(10)	0.031(7)
C22A	0.125(12)	0.038(7)	0.036(7)	−0.002(5)	−0.041(8)	0.007(8)
C23	0.045(3)	0.066(4)	0.057(3)	0.027(3)	−0.004(3)	−0.002(3)
C24	0.287(15)	0.107(7)	0.069(5)	0.036(5)	0.064(8)	0.056(8)
C25	0.069(5)	0.095(6)	0.141(8)	0.076(6)	−0.007(5)	0.001(4)
C26	0.105(7)	0.195(11)	0.166(10)	0.121(9)	−0.043(7)	−0.081(8)
C1A	0.073(7)	0.064(8)	0.066(8)	−0.005(6)	−0.002(6)	−0.014(6)
C12A	0.120(12)	0.059(10)	0.068(10)	0.013(8)	−0.010(9)	0.005(9)
C11A	0.108(8)	0.056(7)	0.038(6)	−0.005(5)	−0.013(6)	0.010(6)
C13A	0.055(8)	0.060(9)	0.067(8)	0.015(7)	−0.029(6)	−0.013(7)
C14A	0.100(11)	0.101(13)	0.121(14)	−0.006(10)	0.012(10)	−0.019(10)
C2A	0.065(7)	0.105(13)	0.073(10)	−0.009(9)	0.001(7)	−0.013(8)
C3A	0.058(7)	0.138(12)	0.054(7)	−0.019(8)	0.008(6)	−0.018(7)
C20	0.075(7)	0.274(18)	0.122(8)	0.086(10)	−0.031(6)	−0.073(9)
C19	0.034(3)	0.081(6)	0.078(6)	0.006(4)	0.001(3)	−0.012(4)
C22	0.089(8)	0.136(9)	0.211(15)	0.098(10)	−0.006(9)	−0.023(7)
C21	0.107(9)	0.233(14)	0.143(10)	−0.054(10)	0.067(8)	−0.064(10)
C6A	0.052(6)	0.087(11)	0.058(7)	0.002(6)	0.004(5)	−0.020(6)
C5A	0.064(6)	0.090(13)	0.059(8)	0.008(8)	0.015(6)	0.000(8)
C4A	0.067(6)	0.067(9)	0.067(8)	0.022(7)	0.013(5)	0.001(7)

C15A	0.107(10)	0.067(9)	0.078(8)	0.030(7)	−0.022(8)	−0.001(8)
C16A	0.122(14)	0.168(17)	0.090(12)	0.042(11)	0.008(10)	0.018(12)
C18A	0.112(13)	0.127(15)	0.131(16)	0.003(13)	−0.010(11)	−0.013(11)
C17A	0.080(11)	0.065(8)	0.084(10)	0.043(7)	0.005(8)	0.012(8)
C23A	0.051(8)	0.110(13)	0.055(10)	0.001(9)	0.012(7)	−0.005(9)
C24A	0.12(2)	0.118(13)	0.13(2)	−0.029(13)	0.006(18)	0.008(13)
C25A	0.081(14)	0.172(19)	0.060(10)	0.016(13)	0.011(10)	−0.026(15)
C26A	0.034(10)	0.117(18)	0.064(9)	−0.005(10)	0.011(7)	−0.012(11)
C9A	0.084(9)	0.068(11)	0.095(13)	−0.024(10)	−0.025(9)	0.003(8)
C10A	0.064(12)	0.057(12)	0.22(3)	−0.004(14)	−0.046(15)	−0.002(9)
C7A	0.071(8)	0.055(10)	0.094(12)	−0.007(9)	0.018(8)	0.016(7)
C8A	0.100(16)	0.12(2)	0.074(15)	−0.056(14)	0.031(13)	−0.023(16)

Supplementary Table 14. Bond lengths and angles for **Cr-1‘**.

Atom–Atom	Length [Å]
I1–Cr1	2.7118(7)
Cr1–O1	1.951(3)
Cr1–O2	1.915(3)
Cr1–O3	1.947(3)
Cr1–O4	1.940(3)
Cr1–O5	2.044(3)
O1–C1	1.279(6)
O1–C6A	1.235(12)
O2–C3	1.228(6)
O2–C1A	1.229(12)
O3–C4	1.273(6)
O3–C3A	1.274(12)
O4–C6	1.260(6)
O4–C4A	1.212(11)
O5–C7	1.460(6)
O5–C9	1.443(7)
O5–C9A	1.410(14)
O5–C7A	1.458(13)
C1–C2	1.397(7)
C1–C11	1.545(8)
C2–H2	0.9500
C2–C3	1.386(7)
C3–C15	1.520(8)
C4–C5	1.392(7)
C4–C19	1.553(8)
C5–H5	0.9500
C5–C6	1.402(7)
C6–C23	1.520(7)
C7–H7A	0.9900
C7–H7B	0.9900
C7–C8	1.502(10)
C8–H8A	0.9800
C8–H8B	0.9800
C8–H8C	0.9800
C9–H9A	0.9900
C9–H9B	0.9900
C9–C10	1.513(10)
C10–H10A	0.9800
C10–H10B	0.9800
C10–H10C	0.9800
C11–C12	1.501(9)
C11–C13	1.513(10)
C11–C14	1.510(10)
C12–H12A	0.9800
C12–H12B	0.9800
C12–H12C	0.9800
C13–H13A	0.9800
C13–H13B	0.9800
C13–H13C	0.9800
C14–H14A	0.9800
C14–H14B	0.9800
C14–H14C	0.9800
C15–C16	1.468(9)
C15–C17	1.479(9)
C15–C18	1.537(10)
C16–H16A	0.9800
C16–H16B	0.9800
C16–H16C	0.9800
C17–H17A	0.9800
C17–H17B	0.9800
C17–H17C	0.9800
C18–H18A	0.9800
C18–H18B	0.9800
C18–H18C	0.9800
C19A–C20A	1.506(13)
C19A–C21A	1.483(13)
C19A–C22A	1.484(13)
C19A–C3A	1.540(14)
C20A–H20A	0.9800
C20A–H20B	0.9800
C20A–H20C	0.9800
C21A–H21A	0.9800
C21A–H21B	0.9800
C21A–H21C	0.9800
C22A–H22A	0.9800
C22A–H22B	0.9800
C22A–H22C	0.9800
C23–C24	1.511(10)
C23–C25	1.492(8)
C23–C26	1.456(9)
C24–H24A	0.9800
C24–H24B	0.9800
C24–H24C	0.9800
C25–H25A	0.9800
C25–H25B	0.9800
C25–H25C	0.9800
C26–H26A	0.9800
C26–H26B	0.9800
C26–H26C	0.9800
C1A–C11A	1.572(14)
C1A–C2A	1.421(14)
C12A–H12D	0.9800
C12A–H12E	0.9800
C12A–H12F	0.9800
C12A–C11A	1.538(13)
C11A–C13A	1.556(13)
C11A–C14A	1.506(13)
C13A–H13D	0.9800
C13A–H13E	0.9800
C13A–H13F	0.9800
C14A–H14D	0.9800
C14A–H14E	0.9800
C14A–H14F	0.9800
C2A–H2A	0.9500
C2A–C3A	1.456(14)
C20–H20D	0.9800
C20–H20E	0.9800
C20–H20F	0.9800
C20–C19	1.470(10)
C19–C22	1.499(11)
C19–C21	1.534(11)

C22–H22D	0.9800
C22–H22E	0.9800
C22–H22F	0.9800
C21–H21D	0.9800
C21–H21E	0.9800
C21–H21F	0.9800
C6A–C5A	1.407(13)
C6A–C23A	1.538(14)
C5A–H5A	0.9500
C5A–C4A	1.393(13)
C4A–C15A	1.541(14)
C15A–C16A	1.486(13)
C15A–C18A	1.477(13)
C15A–C17A	1.509(13)
C16A–H16D	0.9800
C16A–H16E	0.9800
C16A–H16F	0.9800
C18A–H18D	0.9800
C18A–H18E	0.9800
C18A–H18F	0.9800
C17A–H17D	0.9800
C17A–H17E	0.9800
C17A–H17F	0.9800
C23A–C24A	1.493(14)
C23A–C25A	1.500(14)
C23A–C26A	1.490(14)
C24A–H24D	0.9800
C24A–H24E	0.9800
C24A–H24F	0.9800
C25A–H25D	0.9800
C25A–H25E	0.9800
C25A–H25F	0.9800
C26A–H26D	0.9800
C26A–H26E	0.9800
C26A–H26F	0.9800
C9A–H9AA	0.9900
C9A–H9AB	0.9900
C9A–C10A	1.482(16)
C10A–H10D	0.9800
C10A–H10E	0.9800
C10A–H10F	0.9800
C7A–H7AA	0.9900
C7A–H7AB	0.9900
C7A–C8A	1.491(16)
C8A–H8AA	0.9800
C8A–H8AB	0.9800
C8A–H8AC	0.9800

**Atom–Atom–Atom**	**Angle [°]**
O1–Cr1–I1	91.99(9)
O1–Cr1–O5	90.03(12)
O2–Cr1–I1	92.12(10)
O2–Cr1–O1	90.45(13)
O2–Cr1–O3	88.51(14)
O2–Cr1–O4	89.41(14)
O2–Cr1–O5	176.47(13)
O3–Cr1–I1	92.19(9)
O3–Cr1–O1	175.73(12)
O3–Cr1–O5	90.76(13)
O4–Cr1–I1	178.26(10)
O4–Cr1–O1	87.18(12)
O4–Cr1–O3	88.67(12)
O4–Cr1–O5	87.11(13)
O5–Cr1–I1	91.36(9)
C1–O1–Cr1	125.5(3)
C6A–O1–Cr1	128.1(7)
C3–O2–Cr1	130.9(3)
C1A–O2–Cr1	142.2(8)
C4–O3–Cr1	126.3(3)
C3A–O3–Cr1	120.5(8)
C6–O4–Cr1	129.4(3)
C4A–O4–Cr1	132.7(7)
C7–O5–Cr1	121.8(3)
C9–O5–Cr1	123.6(3)
C9–O5–C7	114.6(5)
C9A–O5–Cr1	123.1(10)
C9A–O5–C7A	118.2(13)
C7A–O5–Cr1	118.0(9)
O1–C1–C2	125.3(5)
O1–C1–C11	113.8(5)
C2–C1–C11	120.9(5)
C1–C2–H2	117.8
C3–C2–C1	124.4(5)
C3–C2–H2	117.8
O2–C3–C2	123.2(5)
O2–C3–C15	114.1(5)
C2–C3–C15	122.7(5)
O3–C4–C5	125.1(5)
O3–C4–C19	111.6(5)
C5–C4–C19	123.1(5)
C4–C5–H5	118.3
C4–C5–C6	123.5(5)
C6–C5–H5	118.3
O4–C6–C5	122.5(5)
O4–C6–C23	116.3(5)
C5–C6–C23	121.2(5)
O5–C7–H7A	109.6
O5–C7–H7B	109.6
O5–C7–C8	110.5(6)
H7A–C7–H7B	108.1
C8–C7–H7A	109.6
C8–C7–H7B	109.6
C7–C8–H8A	109.5
C7–C8–H8B	109.5
C7–C8–H8C	109.5
H8A–C8–H8B	109.5
H8A–C8–H8C	109.5
H8B–C8–H8C	109.5
O5–C9–H9A	109.3
O5–C9–H9B	109.3
O5–C9–C10	111.7(7)
H9A–C9–H9B	107.9

C10–C9–H9A	109.3
C10–C9–H9B	109.3
C9–C10–H10A	109.5
C9–C10–H10B	109.5
C9–C10–H10C	109.5
H10A–C10–H10B	109.5
H10A–C10–H10C	109.5
H10B–C10–H10C	109.5
C12–C11–C1	110.0(8)
C12–C11–C13	109.2(8)
C12–C11–C14	109.1(7)
C13–C11–C1	106.3(7)
C14–C11–C1	113.9(7)
C14–C11–C13	108.1(8)
C11–C12–H12A	109.5
C11–C12–H12B	109.5
C11–C12–H12C	109.5
H12A–C12–H12B	109.5
H12A–C12–H12C	109.5
H12B–C12–H12C	109.5
C11–C13–H13A	109.5
C11–C13–H13B	109.5
C11–C13–H13C	109.5
H13A–C13–H13B	109.5
H13A–C13–H13C	109.5
H13B–C13–H13C	109.5
C11–C14–H14A	109.5
C11–C14–H14B	109.5
C11–C14–H14C	109.5
H14A–C14–H14B	109.5
H14A–C14–H14C	109.5
H14B–C14–H14C	109.5
C3–C15–C18	108.7(7)
C16–C15–C3	107.7(7)
C16–C15–C17	115.0(8)
C16–C15–C18	105.3(8)
C17–C15–C3	114.5(7)
C17–C15–C18	105.0(8)
C15–C16–H16A	109.5
C15–C16–H16B	109.5
C15–C16–H16C	109.5
H16A–C16–H16B	109.5
H16A–C16–H16C	109.5
H16B–C16–H16C	109.5
C15–C17–H17A	109.5
C15–C17–H17B	109.5
C15–C17–H17C	109.5
H17A–C17–H17B	109.5
H17A–C17–H17C	109.5
H17B–C17–H17C	109.5
C15–C18–H18A	109.5
C15–C18–H18B	109.5
C15–C18–H18C	109.5
H18A–C18–H18B	109.5
H18A–C18–H18C	109.5
H18B–C18–H18C	109.5
C20A–C19A–C3A	105.8(11)
C21A–C19A–C20A	112.2(12)
C21A–C19A–C22A	110.7(11)
C21A–C19A–C3A	106.5(11)
C22A–C19A–C20A	107.8(10)
C22A–C19A–C3A	113.8(12)
C19A–C20A–H20A	109.5
C19A–C20A–H20B	109.5
C19A–C20A–H20C	109.5
H20A–C20A–H20B	109.5
H20A–C20A–H20C	109.5
H20B–C20A–H20C	109.5
C19A–C21A–H21A	109.5
C19A–C21A–H21B	109.5
C19A–C21A–H21C	109.5
H21A–C21A–H21B	109.5
H21A–C21A–H21C	109.5
H21B–C21A–H21C	109.5
C19A–C22A–H22A	109.5
C19A–C22A–H22B	109.5
C19A–C22A–H22C	109.5
H22A–C22A–H22B	109.5
H22A–C22A–H22C	109.5
H22B–C22A–H22C	109.5
C24–C23–C6	105.2(6)
C25–C23–C6	112.3(6)
C25–C23–C24	106.4(7)
C26–C23–C6	111.0(7)
C26–C23–C24	110.5(8)
C26–C23–C25	111.2(7)
C23–C24–H24A	109.5
C23–C24–H24B	109.5
C23–C24–H24C	109.5
H24A–C24–H24B	109.5
H24A–C24–H24C	109.5
H24B–C24–H24C	109.5
C23–C25–H25A	109.5
C23–C25–H25B	109.5
C23–C25–H25C	109.5
H25A–C25–H25B	109.5
H25A–C25–H25C	109.5
H25B–C25–H25C	109.5
C23–C26–H26A	109.5
C23–C26–H26B	109.5
C23–C26–H26C	109.5
H26A–C26–H26B	109.5
H26A–C26–H26C	109.5
H26B–C26–H26C	109.5
O2–C1A–C11A	114.3(12)
O2–C1A–C2A	107.1(13)
C2A–C1A–C11A	138.5(14)
H12D–C12A–H12E	109.5
H12D–C12A–H12F	109.5
H12E–C12A–H12F	109.5
C11A–C12A–H12D	109.5
C11A–C12A–H12E	109.5

C11A–C12A–H12F	109.5
C12A–C11A–C1A	116.8(16)
C12A–C11A–C13A	103.4(10)
C13A–C11A–C1A	123.3(15)
C14A–C11A–C1A	99.6(16)
C14A–C11A–C12A	106.8(11)
C14A–C11A–C13A	105.4(11)
C11A–C13A–H13D	109.5
C11A–C13A–H13E	109.5
C11A–C13A–H13F	109.5
H13D–C13A–H13E	109.5
H13D–C13A–H13F	109.5
H13E–C13A–H13F	109.5
C11A–C14A–H14D	109.5
C11A–C14A–H14E	109.5
C11A–C14A–H14F	109.5
H14D–C14A–H14E	109.5
H14D–C14A–H14F	109.5
H14E–C14A–H14F	109.5
C1A–C2A–H2A	114.2
C1A–C2A–C3A	131.7(17)
C3A–C2A–H2A	114.2
O3–C3A–C19A	116.2(11)
O3–C3A–C2A	120.3(15)
C2A–C3A–C19A	114.2(12)
H20D–C20–H20E	109.5
H20D–C20–H20F	109.5
H20E–C20–H20F	109.5
C19–C20–H20D	109.5
C19–C20–H20E	109.5
C19–C20–H20F	109.5
C20–C19–C4	115.0(8)
C20–C19–C22	112.2(9)
C20–C19–C21	108.1(8)
C22–C19–C4	110.4(7)
C22–C19–C21	103.5(8)
C21–C19–C4	106.7(7)
C19–C22–H22D	109.5
C19–C22–H22E	109.5
C19–C22–H22F	109.5
H22D–C22–H22E	109.5
H22D–C22–H22F	109.5
H22E–C22–H22F	109.5
C19–C21–H21D	109.5
C19–C21–H21E	109.5
C19–C21–H21F	109.5
H21D–C21–H21E	109.5
H21D–C21–H21F	109.5
H21E–C21–H21F	109.5
O1–C6A–C5A	123.3(14)
O1–C6A–C23A	114.5(13)
C5A–C6A–C23A	122.1(13)
C6A–C5A–H5A	117.0
C4A–C5A–C6A	126.0(15)
C4A–C5A–H5A	117.0
O4–C4A–C5A	119.5(13)
O4–C4A–C15A	112.6(11)
C5A–C4A–C15A	127.5(12)
C16A–C15A–C4A	108.9(12)
C16A–C15A–C17A	108.3(11)
C18A–C15A–C4A	110.4(12)
C18A–C15A–C16A	111.7(12)
C18A–C15A–C17A	109.7(12)
C17A–C15A–C4A	107.8(11)
C15A–C16A–H16D	109.5
C15A–C16A–H16E	109.5
C15A–C16A–H16F	109.5
H16D–C16A–H16E	109.5
H16D–C16A–H16F	109.5
H16E–C16A–H16F	109.5
C15A–C18A–H18D	109.5
C15A–C18A–H18E	109.5
C15A–C18A–H18F	109.5
H18D–C18A–H18E	109.5
H18D–C18A–H18F	109.5
H18E–C18A–H18F	109.5
C15A–C17A–H17D	109.5
C15A–C17A–H17E	109.5
C15A–C17A–H17F	109.5
H17D–C17A–H17E	109.5
H17D–C17A–H17F	109.5
H17E–C17A–H17F	109.5
C24A–C23A–C6A	113(2)
C24A–C23A–C25A	109.3(12)
C25A–C23A–C6A	109.7(17)
C26A–C23A–C6A	104.3(18)
C26A–C23A–C24A	110.5(12)
C26A–C23A–C25A	110.0(12)
C23A–C24A–H24D	109.5
C23A–C24A–H24E	109.5
C23A–C24A–H24F	109.5
H24D–C24A–H24E	109.5
H24D–C24A–H24F	109.5
H24E–C24A–H24F	109.5
C23A–C25A–H25D	109.5
C23A–C25A–H25E	109.5
C23A–C25A–H25F	109.5
H25D–C25A–H25E	109.5
H25D–C25A–H25F	109.5
H25E–C25A–H25F	109.5
C23A–C26A–H26D	109.5
C23A–C26A–H26E	109.5
C23A–C26A–H26F	109.5
H26D–C26A–H26E	109.5
H26D–C26A–H26F	109.5
H26E–C26A–H26F	109.5
O5–C9A–H9AA	108.6
O5–C9A–H9AB	108.6
O5–C9A–C10A	114.8(14)
H9AA–C9A–H9AB	107.5
C10A–C9A–H9AA	108.6
C10A–C9A–H9AB	108.6

C9A–C10A–H10D	109.5	H7AA–C7A–H7AB	108.0
C9A–C10A–H10E	109.5	C8A–C7A–H7AA	109.4
C9A–C10A–H10F	109.5	C8A–C7A–H7AB	109.4
H10D–C10A–H10E	109.5	C7A–C8A–H8AA	109.5
H10D–C10A–H10F	109.5	C7A–C8A–H8AB	109.5
H10E–C10A–H10F	109.5	C7A–C8A–H8AC	109.5
O5–C7A–H7AA	109.4	H8AA–C8A–H8AB	109.5
O5–C7A–H7AB	109.4	H8AA–C8A–H8AC	109.5
O5–C7A–C8A	111.1(14)	H8AB–C8A–H8AC	109.5

Supplementary Table 15. Torsion angles of **Cr-1‘.**

**Atom–Atom–Atom–Atom**	**Torsion Angle [°]**		
Cr1–O1–C1–C2	−6.9(8)	O3–C4–C19–C21	70.9(9)
Cr1–O1–C1–C11	171.4(4)	O4–C6–C23–C24	101.5(8)
Cr1–O1–C6A–C5A	11(3)	O4–C6–C23–C25	−143.2(7)
Cr1–O1–C6A–C23A	−166.0(12)	O4–C6–C23–C26	−18.0(10)
Cr1–O2–C3–C2	1.8(8)	O4–C4A–C15A–C16A	−179.2(16)
Cr1–O2–C3–C15	179.5(4)	O4–C4A–C15A–C18A	−56.2(18)
Cr1–O2–C1A–C11A	168.2(7)	O4–C4A–C15A–C17A	63.6(17)
Cr1–O2–C1A–C2A	−9(3)	C1–C2–C3–O2	0.5(10)
Cr1–O3–C4–C5	−11.3(8)	C1–C2–C3–C15	−177.1(6)
Cr1–O3–C4–C19	163.9(5)	C2–C1–C11–C12	−131.3(8)
Cr1–O3–C3A–C19A	−177.3(9)	C2–C1–C11–C13	110.6(9)
Cr1–O3–C3A–C2A	38(2)	C2–C1–C11–C14	−8.4(11)
Cr1–O4–C6–C5	9.1(8)	C2–C3–C15–C16	−111.5(9)
Cr1–O4–C6–C23	−169.7(4)	C2–C3–C15–C17	17.9(11)
Cr1–O4–C4A–C5A	−16(2)	C2–C3–C15–C18	134.9(8)
Cr1–O4–C4A–C15A	171.1(7)	C4–C5–C6–O4	9.6(9)
Cr1–O5–C7–C8	−102.1(7)	C4–C5–C6–C23	−171.7(6)
Cr1–O5–C9–C10	−98.7(6)	C5–C4–C19–C20	6.0(12)
Cr1–O5–C9A–C10A	104(2)	C5–C4–C19–C22	134.3(9)
Cr1–O5–C7A–C8A	98.5(19)	C5–C4–C19–C21	−113.8(9)
O1–C1–C2–C3	2.4(10)	C5–C6–C23–C24	−77.2(9)
O1–C1–C11–C12	50.4(9)	C5–C6–C23–C25	38.1(9)
O1–C1–C11–C13	−67.8(9)	C5–C6–C23–C26	163.3(9)
O1–C1–C11–C14	173.3(7)	C7–O5–C9–C10	79.0(7)
O1–C6A–C5A–C4A	1(4)	C9–O5–C7–C8	80.1(8)
O1–C6A–C23A–C24A	66(2)	C11–C1–C2–C3	−175.8(6)
O1–C6A–C23A–C25A	−171.7(16)	C20A–C19A–C3A–O3	−39.9(18)
O1–C6A–C23A–C26A	−54(2)	C20A–C19A–C3A–C2A	107.2(17)
O2–C3–C15–C16	70.8(9)	C21A–C19A–C3A–O3	−159.5(15)
O2–C3–C15–C17	−159.9(8)	C21A–C19A–C3A–C2A	−12.4(19)
O2–C3–C15–C18	−42.8(9)	C22A–C19A–C3A–O3	78.3(18)
O2–C1A–C11A–C12A	62(2)	C22A–C19A–C3A–C2A	−134.7(17)
O2–C1A–C11A–C13A	−67(2)	C1A–C2A–C3A–O3	−29(4)
O2–C1A–C11A–C14A	176.9(16)	C1A–C2A–C3A–C19A	−175(2)
O2–C1A–C2A–C3A	11(3)	C11A–C1A–C2A–C3A	−165(2)
O3–C4–C5–C6	−8.3(9)	C2A–C1A–C11A–C12A	−122(3)
O3–C4–C19–C20	−169.2(9)	C2A–C1A–C11A–C13A	108(3)
O3–C4–C19–C22	−41.0(10)	C2A–C1A–C11A–C14A	−8(3)
		C19–C4–C5–C6	177.1(6)
		C6A–C5A–C4A–O4	1(3)

C6A–C5A–C4A–C15A	173.0(19)	C5A–C4A–C15A–C18A	131(2)
C5A–C6A–C23A–C24A	−111(2)	C5A–C4A–C15A–C17A	−109(2)
C5A–C6A–C23A–C25A	12(3)	C23A–C6A–C5A–C4A	177(2)
C5A–C6A–C23A–C26A	129(2)	C9A–O5–C7A–C8A	−91(2)
C5A–C4A–C15A–C16A	8(2)	C7A–O5–C9A–C10A	−65(2)

Experimental: Cr-1' was crystallized from Et_2O by evaporation at 25 °C. A clear dark green, block-shaped crystal of **Cr-1'** was mounted on the goniometer. Data were collected from a shock-cooled single crystal at 100.0(1) K on a XtaLAB Synergy, Dualflex, Pilatus 300K four-circle diffractometer with a micro-focus sealed X-ray tube using a mirror as monochromator and a Pilatus 300K detector. The diffractometer was equipped with a low temperature device and used Cu K_α radiation (λ = 1.54184 Å). All data were integrated with Crysalispro and a gaussian absorption correction using SCALE3 ABSPACK was applied. The structure was solved by dual methods using SHELXT and refined by full-matrix least-squares methods against F^2 by SHELXL using Olex2. All non-hydrogen atoms were refined with anisotropic displacement parameters. All C-bound hydrogen atoms were refined isotropic on calculated positions using a riding model with their U_{iso} values constrained to 1.5 times the U_{eq} of their pivot atoms for terminal sp^3 carbon atoms and 1.2 times for all other carbon atoms. Disordered moieties were refined using bond lengths restraints and displacement parameter restraints.

10 References

1. Makai, S., Falk, E. & Morandi, B. Direct Synthesis of Unprotected 2-Azidoamines from Alkenes via an Iron-Catalyzed Difunctionalization Reaction. *Journal of the American Chemical Society* **142**, 21548-21555 (2020).
2. Falk, E., Makai, S., Delcaillau, T., Gürtler, L. & Morandi, B. Design and Scalable Synthesis of N-Alkylhydroxylamine Reagents for the Direct Iron-Catalyzed Installation of Medicinally Relevant Amines**. *Angewandte Chemie International Edition* **59**, 21064-21071 (2020).
3. Kraft, P. & Cadalbert, R. The Thia-Analog of Ambrettolide. Synthesis and Odor of 1,8-Oxathiacyclohexadecan-2-one. *Synlett* **1997**, 600-602 (1997).
4. Andersen, C. *et al.* Introduction of Cyclopropyl and Cyclobutyl Ring on Alkyl Iodides through Cobalt-Catalyzed Cross-Coupling. *Organic Letters* **21**, 2285-2289 (2019).
5. Liang, S. *et al.* Synthesis of Alkyl Halides from Aldehydes via Deformylative Halogenation. *Organic Letters* **21**, 3848-3854 (2019).
6. Liguori, L., Bjørsvik, H.-R., Bravo, A., Fontana, F. & Minisci, F. A new direct homolytic iodination reaction of alkanes by perfluoroalkyl iodides. *Chemical Communications*, 1501-1502 (1997).
7. Akashi, T., Nefuji, T., Yoshida, M. & Hosoda, J. Quantitative determination of tautomeric FK506 by reversed-phase liquid chromatography. *Journal of Pharmaceutical and Biomedical Analysis* **14**, 339-346 (1996).
8. Ling, T., Poupon, E., Rueden, E.J., Kim, S.H. & Theodorakis, E.A. Unified Synthesis of Quinone Sesquiterpenes Based on a Radical Decarboxylation and Quinone

Addition Reaction. *Journal of the American Chemical Society* **124**, 12261-12267 (2002).
9. Zhou, W., Desnoyer, A.N., Bailey, J.A., Patrick, B.O. & Smith, K.M. Direct Synthesis of Ligand-Based Radicals by the Addition of Bipyridine to Chromium(II) Compounds. *Inorganic Chemistry* **52**, 2271-2273 (2013).
10. Shevick, S.L., Obradors, C. & Shenvi, R.A. Mechanistic Interrogation of Co/Ni-Dual Catalyzed Hydroarylation. *Journal of the American Chemical Society* **140**, 12056-12068 (2018).
11. Wilson, C.V. *et al.* Cobalt–Carbon Bonding in a Salen-Supported Cobalt(IV) Alkyl Complex Postulated in Oxidative MHAT Catalysis. *Journal of the American Chemical Society* **144**, 10361-10367 (2022).
12. Takai, K., Nitta, K., Fujimura, O. & Utimoto, K. Preparation of alkylchromium reagents by reduction of alkyl halides with chromium(II) chloride under cobalt catalysis. *The Journal of Organic Chemistry* **54**, 4732-4734 (1989).
13. Bam, R., Pollatos, A.S., Moser, A.J. & West, J.G. Mild olefin formation via bio-inspired vitamin B12 photocatalysis. *Chemical Science* **12**, 1736-1744 (2021).
